# The Impact of Orthodontic-Related Social Media Content on Patients’ Willingness to Initiate Treatment: A Systematic Review

**DOI:** 10.3390/dj14050263

**Published:** 2026-05-01

**Authors:** Konstantinos Lappas, Efthymia Tsialta, Nefeli Katanaki, Ioanna Pouliezou, Iosif Sifakakis

**Affiliations:** 1 School of Dentistry, National and Kapodistrian University of Athens, 2 Thivon Str., 11527 Athens, Greece; konslapp@yahoo.gr (K.L.); etsialta17@gmail.com (E.T.); 2 Department of Orthodontics, School of Dentistry, Aristotle University of Thessaloniki, 54124 Thessaloniki, Greece; nkatana@dent.auth.gr; 3 Department of Orthodontics, School of Dentistry, National and Kapodistrian University of Athens, 2 Thivon Str., 11527 Athens, Greece; ipouliez@dent.uoa.gr

**Keywords:** orthodontics, orthodontic treatment, social media, willingness, influence, systematic review

## Abstract

**Background/Objectives**: Nowadays, social media is increasingly utilized in the field of orthodontics for information sharing and promotion, yet its influence on patients’ willingness to initiate orthodontic treatment remains insufficiently defined. This systematic review aims to synthesize the available evidence on the impact of orthodontic-related social media content on patients’ willingness to seek orthodontic treatment. **Methods**: An extensive literature search was performed across five electronic databases up to August 2025, complemented by manual screening of reference lists. Randomized and non-randomized studies evaluating orthodontic-related social media exposure and reported treatment-related willingness or motivation outcomes were considered for inclusion. **Results**: A total of 1243 records were identified, and eight studies met the inclusion criteria, including six cross-sectional studies, one randomized controlled trial, and one qualitative study. Given the diversity of study designs and assessment methods, the results were synthesized narratively. Visually oriented orthodontic-related social media posts, particularly outcome-focused imagery such as before–after photographs, were more frequently associated with increased willingness to seek orthodontic treatment compared with technical content. Gender-related differences were reported, with female participants appearing more responsive to orthodontic-related social media exposure. Across the included studies, Instagram was identified as the platform exerting the strongest influence. **Conclusions**: The findings of this systematic review indicate that visually oriented orthodontic-related social media content, particularly outcome-focused imagery such as before–after photographs, shows more consistent associations with willingness to seek orthodontic treatment, alongside gender-related differences and platform-specific effects.

## 1. Introduction

### 1.1. Rationale

Social media constitutes an integral part of modern society, transforming how people communicate, form communities, and access information across various domains [1]. As one of the most widely adopted technologies of the digital age, social media now shapes everyday communication and information exchange for billions of individuals worldwide. In 2025, more than 5.2 billion people, representing over 65% of the global population, actively used social media, with younger cohorts remaining the most engaged demographic group [2]. The social media landscape continues to evolve, as emerging platforms such as TikTok and Threads rapidly gain popularity, while usage patterns of older platforms vary across generations and regions [2].

This widespread adoption of social media marketing and advertising [3,4] has transformed the healthcare sector by facilitating more effective interaction between providers and patients and by positioning these platforms as a leading source of health information, particularly among younger populations [5,6]. Their accessibility and interactive features further position social media as a valuable tool for disseminating medical knowledge, strengthening patient education, fostering public engagement [7], and promoting dental services to attract and inform prospective patients [8,9,10,11]. Nevertheless, despite their advantages, social media platforms also entail potential drawbacks, including the dissemination of misinformation and risks to patient–dentist confidentiality [12].

In dentistry, this trend is reflected in both professional and patient behavior. Social media posts in the dental field help foster loyalty among existing patients and inform prospective ones about clinical procedures [13]. Moreover, as a marketing and educational tool, social media has become crucial for orthodontists’ relationships with both current and potential patients [9]. Recent data indicate that 67.5% of dental practitioners in the Philippines actively use social media to communicate with patients and share educational content, emphasizing its growing role in patient interaction and professional marketing [14]. In orthodontics specifically, 76% of practitioners report using social media as part of their professional practice, making it one of the most prevalent marketing and communication strategies among orthodontic clinics [11]. Furthermore, increased social media activity has been correlated with higher numbers of new patient starts, demonstrating its effectiveness in promoting orthodontic services and enhancing patient interaction [11].

From the patient’s perspective, social media has become a significant source of health information and a major influence in decision-making regarding dental care. Many patients report using these platforms to learn about available services, explore treatment options, and evaluate practitioners’ reputations before choosing a provider. For instance, 36% of respondents in one study in the U.K. reported searching for their dentist on social media [15], while approximately 30% of orthodontic patients indicated that they use these platforms to obtain information about orthodontic treatment [16]. Moreover, positive online reviews have been identified as key determinants influencing patients’ selection of dental clinics and practitioners’ social media profiles [17]. Beyond information seeking, exposure to esthetic dental content on social media has been shown to influence patients’ treatment preferences. Evidence indicates that in Spain approximately 29% of individuals reported social media as a factor in their decision to undergo an esthetic dental procedure, while those who had recently changed dental practices were more likely to have interacted with clinics’ social media pages [18,19].

We aim to better understand how social media influences patients’ perceptions, as these platforms also play an important role in shaping appearance-related preferences [20]. Social media content often presents idealized and selectively curated representations of appearance, which may contribute to unrealistic esthetic expectations and negatively influence individuals’ self-perception [21]. Their highly visual nature promotes frequent exposure to appearance-related content and encourages comparisons with others, which have been shown to influence users’ attitudes toward their own appearance [22]. In dentistry, this is particularly relevant, as exposure to social media content has been found to affect individuals’ perceptions of smile esthetics [23]. As dental appearance is a key component of facial attractiveness and plays an important role in the formation of first impressions [24], these influences affect how individuals perceive their own smile and dental appearance, which are important considerations in the decision to seek orthodontic treatment.

The decision to pursue orthodontic treatment is influenced by a combination of esthetic, functional, and psychosocial factors. Esthetic improvement consistently emerges as the primary motivation, with many patients seeking treatment to enhance facial appearance and achieve a more attractive smile. Other commonly reported motives include improving self-confidence, oral function, and meeting perceived social expectations regarding dental esthetics [25]. For adolescents and young adults, social and peer influences further shape the decision to initiate treatment [26]. Although these motivations operate on an individual level, they mirror a broader evolution within dentistry, wherein the discipline has increasingly shifted its focus from purely functional correction toward esthetic enhancement, with facial appearance often regarded as an indicator of social value [27].

In recent years, the overall demand for orthodontic treatment has expanded beyond adolescents to include a growing number of adult patients. According to data from the American Association of Orthodontists, the number of adult orthodontic patients in the United States has reached its highest level to date, reflecting increased awareness of dental esthetics, greater access to care, and broader social acceptance of orthodontic care [28].

However, the extent to which social media exposure affects orthodontic treatment-related decision-making remains insufficiently explored, highlighting the need for a systematic assessment of the existing evidence. Consequently, understanding how social media shapes or reinforces these motivations is essential for evaluating patients’ willingness to initiate orthodontic treatment.

### 1.2. Objectives

The objective of this systematic review is to evaluate the current scientific evidence regarding the influence of orthodontic-related social media content on patients’ willingness to initiate treatment.

## 2. Materials and Methods

### 2.1. Protocol and Registration

This systematic review was conducted following the methodological guidelines of the Cochrane Handbook for Systematic Reviews of Interventions (version 6.5) [29]. This review was reported in accordance with the PRISMA statement (Preferred Reporting Items for Systematic Reviews and Meta-Analysis) [30]. The protocol of the review was registered in PROSPERO (International Prospective Register of Systematic Reviews) under the registration number CRD420251133633 ‘’https://www.crd.york.ac.uk/PROSPERO/view/CRD420251133633 (accessed on 25 August 2025)’’. The evaluation followed the registered process strictly, without deviations from its initial design.

### 2.2. Eligibility Criteria

#### 2.2.1. Inclusion Criteria

Studies were considered eligible if they met predefined inclusion criteria based on the Population, Exposure, Comparison, Outcome, and Study Design (PECOS) framework, as described below. Only studies published in English were included.

#### 2.2.2. Types of Participants

Eligible studies included human participants of any age or gender who had been exposed to orthodontic-related content on social media platforms.

#### 2.2.3. Types of Exposures

Exposure was defined as interaction with orthodontic-related content on social media, including but not limited to before-and-after images, promotional material, patient-generated reviews, clinician-generated posts, esthetic smile photographs, and treatment advertisements across major social media platforms (Facebook, Instagram, TikTok, YouTube, Twitter/X, Snapchat).

#### 2.2.4. Comparisons

A limited number of included studies incorporated explicit comparison groups, typically contrasting different levels or forms of exposure to orthodontic-related social media content, such as variations in image-based material or differing degrees of informational exposure.

#### 2.2.5. Types of Outcome Measures

The primary outcome concerned individuals’ willingness to undergo orthodontic treatment after exposure to orthodontic-related social media content. Secondary outcomes encompassed broader psychosocial responses, including motivation to pursue treatment, tendencies related to treatment-seeking behavior, perceived need for orthodontic care, and impressions regarding the credibility of orthodontists or dental professionals presented through social media.

#### 2.2.6. Study Design

Randomized and non-randomized study designs, including cross-sectional studies, qualitative studies and randomized controlled trials, were considered eligible for inclusion. No restrictions were applied regarding study duration, data collection methods, or analytical approach, provided that the study design aligned with the predefined eligibility criteria.

#### 2.2.7. Exclusion Criteria

Studies that did not investigate outcomes reflecting the impact of orthodontic-related social media exposure on willingness to initiate treatment, motivation, treatment-seeking behavior, perceived treatment need, or credibility perceptions, were excluded. In addition, studies focusing solely on dental professionals, individuals without access to social media and general internet searches unrelated to social media platforms were excluded. This review also excluded case reports, case series, non-clinical studies, literature reviews, abstracts, systematic reviews, and opinion pieces.

### 2.3. Information Sources

Extensive research of five electronic databases was conducted without restrictions on publication year, covering records from inception up to August 2025: PubMed, Cochrane Library, ScienceDirect, Scopus and ProQuest. Additionally, the reference lists of eligible studies and relevant systematic reviews were examined to identify further potentially relevant articles.

### 2.4. Search Strategy

The electronic database search was undertaken by two reviewers (K.L, E.T) utilizing combinations of free text words tailored to each database. The detailed search strategies for all databases are presented in Table 1. Only studies published in English were considered, with no publication date restrictions. Gray literature sources, including dissertations and theses retrieved through ProQuest, were also screened. Additional studies were identified by screening the reference lists of the included studies to capture any relevant records not retrieved during the initial database search.

### 2.5. Study Selection

Duplicate records were identified and removed manually, after which the study selection process was conducted in two sequential stages. First, two reviewers (K.L, E.T) independently screened the titles and abstracts of all retrieved studies. This was followed by an independent full-text assessment by the same reviewers to determine whether each study met the eligibility criteria. Prior to screening, the reviewers aligned on the eligibility criteria to ensure consistent application. Any disagreements that emerged during either stage were resolved through discussion, and when consensus could not be reached, a third reviewer (IP) was consulted. The detailed study selection process is provided in Table S2 (Supplementary Materials).

### 2.6. Data Collection and Data Items

Data extraction was independently performed by two reviewers (K.L., E.T.) using a standardized form developed for this review. Extracted information included general study details (first author, publication year, country, study setting), study characteristics (design and data-collection methods), participant demographics and sample size, and the type of social media exposure assessed. Data relating to the predefined outcomes willingness to initiate treatment, motivation, treatment-seeking behavior, perceived treatment need, and credibility-related perceptions were also recorded, along with the main findings relevant to the impact of orthodontic-related social media content.

### 2.7. Risk of Bias Assessment in Included Studies

The risk of bias of all included studies was independently assessed by two reviewers (KL, ET). Any disagreements were resolved through discussion, and a third reviewer (IP) was consulted when necessary.

For the cross-sectional studies, methodological quality was evaluated using the Modified Newcastle–Ottawa Scale (NOS) for cross-sectional designs [31]. This tool assesses three domains, Selection, Comparability, and Outcome, using a star-based system.

For the randomized controlled trial, the Revised Cochrane Risk-of-Bias tool for Randomized Trials (RoB 2) was applied [32]. This tool evaluates five domains of potential bias: bias arising from the randomization process, deviations from intended interventions, missing outcome data, measurement of the outcome, and selection of the reported results. Each domain was rated as “low risk,” “some concerns,” or “high risk,” and the overall risk of bias for the trial was determined by the highest level of bias across any domain.

For the qualitative study, methodological quality was appraised using the ten-item Joanna Briggs Institute (JBI) Critical Appraisal Checklist for Qualitative Research [33].

### 2.8. Effect Measures and Data Synthesis

A narrative synthesis of the findings was pre-specified and applied due to the considerable heterogeneity in study designs, outcome measures, and exposure types across the included studies, which did not permit statistical pooling or meta-analysis. Disagreements were settled through discussion or, when necessary, consultation with a third author (IP) prior to making the final decision.

## 3. Results

### 3.1. Study Selection

**Figure 1:**
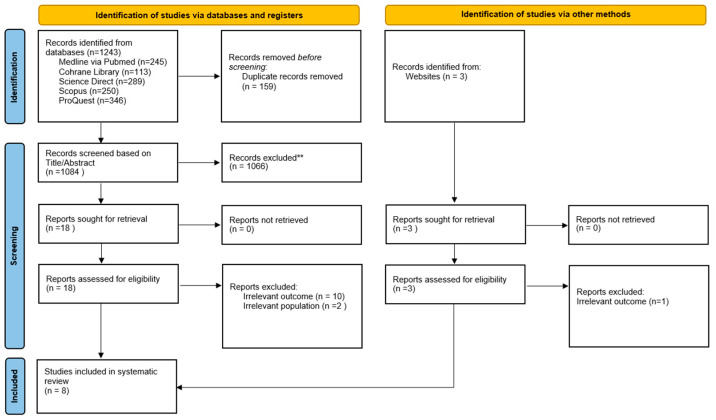
PRISMA 2020 flow diagram illustrating study selection process, including identification, screening, eligibility assessment, and final inclusion of studies.

The database search produced 1243 records, from which 159 duplicates were removed, resulting in 1084 studies entering the screening phase. Title and abstract assessment led to the exclusion of 1066 records. Eighteen full-text articles were then evaluated, and 12 were excluded for not meeting the predefined criteria. Hand-searching identified three additional studies, two of which were eligible. Ultimately, eight studies met all inclusion criteria and were incorporated into the qualitative review [34,35,36,37,38,39,40,41]. The study selection process is illustrated in the PRISMA 2020 flow diagram presented in Figure 1.

### 3.2. Study Characteristics

The characteristics of the included studies are summarized in Table 2. The eight included studies comprised six cross-sectional surveys [34,35,36,37,38,39] one randomized controlled trial [40] and one qualitative interview study [41], published between 2021 and 2026. Sample sizes ranged from 15 to 1267 participants and included laypeople, orthodontic patients, dental students, dentists, and adults seeking orthodontic treatment of various age groups.

Outcome assessment methods differed across the included studies. The cross-sectional studies relied on self-administered questionnaires with heterogeneous formats; the randomized controlled trial employed a structured questionnaire with a numerical rating scale and the qualitative study used semi-structured interviews.

Across the included studies, the social media content evaluated included before–after images, educational posts, marketing material, appliance-related photographs, clear aligner advertisements, and digitally modified smile images. The platforms assessed varied between studies and included Instagram, Facebook, Snapchat, TikTok, Twitter, YouTube, LinkedIn, WhatsApp, and Telegram. Considerable methodological and clinical heterogeneity was observed across study designs, populations, exposures, and outcome measures.

### 3.3. Risk of Bias Within Studies

Risk of bias in the cross-sectional studies was assessed using the modified Newcastle–Ottawa Scale [31], with detailed domain-level scoring reported in Appendix Table A1. Overall scores ranged from 5 to 6 stars [34,35,36,37,38,39]. The randomized controlled trial was assessed using the RoB 2 tool (Appendix Table A2) and was judged as having some concerns overall [40]. The qualitative study was appraised using the JBI Critical Appraisal Checklist for Qualitative Research (Appendix Table A3). Overall, the study demonstrated good congruence between its research question, methodological approach, data collection procedures, analytic strategy, and interpretation of findings [41].

### 3.4. Synthesis of Results

Across the included studies, the influence of social media on orthodontic-related decisions was examined. Alalola et al. [34] assessed how image-based orthodontic posts influence willingness to seek treatment, while Al-Gunaid et al. [35] examined whether social media use affects orthodontic decision-making. Diniz et al. [36] investigated the influence of social media on patients’ choice of orthodontist and acceptance of proposed orthodontic treatment. Meira et al. [37] evaluated credibility judgments toward different orthodontic image categories posted by clinicians, and Adanan et al. [41] qualitatively explored how young adults interpret orthodontic marketing on social media. Patil et al. [38] investigated the influence of social media-derived information on decisions to undergo clear aligner therapy, Karkun et al. [40] examined whether digitally corrected smile photographs of the participants, presented on Instagram, motivate and influence them to seek orthodontic treatment, and Sampson et al. [39] explored how social media exposure shapes perceptions and acceptance of temporary anchorage devices.

A quantitative synthesis could not be undertaken due to the pronounced clinical and methodological variability across the included studies. Each included study used different methodological approaches, forms of exposure, participant samples and strategies for evaluating outcomes, and they employed varied measurement instruments and rating scales to capture these outcome measures. This degree of heterogeneity prevented the derivation of a coherent pooled estimate and rendered any attempt at statistical aggregation inappropriate and potentially misleading. Consequently, in line with Cochrane recommendations, the evidence was synthesized narratively to accommodate these differences. As no meta-analysis was feasible, the GRADE framework for rating the certainty of evidence could not be applied.

### 3.5. Results of Individual Studies

#### 3.5.1. Willingness to Seek Orthodontic Treatment

The included studies reported mixed findings regarding the influence of social media exposure on willingness to seek orthodontic treatment. Adanan et al. [41] reported that social media exposure enhanced awareness and motivation among young adults. In the randomized controlled trial by Karkun et al. [40], participants completed a questionnaire using a 10-point scale. For the item assessing whether Instagram influenced the decision to seek orthodontic treatment, mean scores were higher in the experimental group compared with the control group (7.09 ± 2.28 vs. 6.17 ± 1.42; *p* < 0.05). In addition, strong influence (scores ≥ 9) was reported by 24.1% of participants in the experimental group and 0.8% in the control group (*p* < 0.05). A large proportion of participants in both groups stated that viewing ideal-smile images on Instagram and subsequently seeing their own digitally corrected smile encouraged them to consider orthodontic treatment (91.4% in the control group and 75.9% in the experimental group; *p* > 0.05). Additionally, 92.1% of the control group and 81.2% of the experimental group stated that they would have begun treatment earlier if such comparisons had been available (*p* > 0.05). However, Al-Gunaid et al. [35] found that 58.9% of participants reported that social media did not directly affect their decision to seek orthodontic treatment (*p* > 0.05).

#### 3.5.2. Social Media Platforms

Patil et al. [38] reported that Instagram was the most influential platform (66.6%) among users viewing orthodontic content. Similarly, Diniz et al. [36] identified Instagram as the most influential platform in orthodontic decision-making, being significantly associated with both the choice of orthodontist and the selection of orthodontic treatment (*p* < 0.05).

#### 3.5.3. Types of Social Media Content

Several studies evaluated the effect of specific post types on willingness. Alalola et al. [34] found that before–after images produced the highest willingness scores among all categories assessed (*p* < 0.05). Meira et al. [37] observed increased willingness for before–after posts and for posts portraying the orthodontist engaged in teaching or academic activities (*p* < 0.05). Diniz et al. [36] further reported that before–after posts on social media were considered important when choosing an orthodontist (*p* < 0.05).

#### 3.5.4. Treatment-Specific Influences

Treatment-specific influences were also reported. Patil et al. [38] reported that social media influenced the consideration of clear aligner therapy in 95% of participants, with 62.33% reporting a high level of influence (*p* < 0.05). Adanan et al. [41] noted that some individuals were specifically drawn to clear aligner promotional content. Sampson et al. [39] found that targeted exposure to mini-screw content increased acceptance of treatment involving temporary anchorage devices (*p* < 0.05).

#### 3.5.5. Demographics

Al-Gunaid et al. [35] reported that the influence of social media on treatment decision-making was significantly higher among females than males (*p* < 0.01). Patil et al. [38] reported a greater influence of social media among women compared with men when considering clear aligner therapy (42.8% vs. 31.5%; *p* < 0.05). Diniz et al. [36] also reported that women considered before–after posts more important than men when selecting an orthodontist (*p* < 0.05). In addition, Alalola et al. [34] reported higher willingness among women for selected content categories, including “being a teacher,” “participating in scientific events,” and “before and after (intraoral)” (*p* < 0.01).

#### 3.5.6. Decision-Making Aspects

Social media also affected aspects of orthodontic decision-making. Adanan et al. [41] reported that treatment decisions were influenced by online reviews, follower counts, third-party endorsements, trending content, treatment cost, clinic location, and the clarity and availability of online information. Alalola et al. [34] found that 50.7% of participants would use social media to search for an orthodontist. Al-Gunaid et al. [35] reported that 73.3% of participants considered searching social media important before seeking orthodontic treatment (*p* < 0.001).

#### 3.5.7. Credibility and Trust

Perceptions related to credibility and trust were documented across multiple studies. Meira et al. [37] found that before–after posts and teaching-related posts received the highest credibility ratings among laypeople, whereas mechanical, social, and family-related posts received lower ratings. Adanan et al. [41] reported that misinformation and unclear content sources decreased trust in orthodontic information online. Sampson et al. [39] found that 79.1% of participants would trust their clinician more if the clinician maintained an active social media profile.

## 4. Discussion

### 4.1. Summary of Evidence

Social media provides an advertising-friendly environment for dental practice, as they are widely used by dental professionals to promote services, increase practice visibility, and engage potential patients through online content [14]. From the patient perspective, exposure to dentists’ social media content has been shown to influence perceptions of dental practices and, in some cases, the selection of dentists or dental clinics [42]. Moreover, patients’ strong interest in esthetic dental treatments and their outcomes has been reported to influence marketing practices, with many clinics prioritizing the presentation of cosmetic results in their promotional content [43].

This review revealed several consistent patterns across the included studies. Before–after photographs of patients who had previously completed orthodontic treatment emerged as the most persuasive form of content, consistently associated with increased willingness to seek treatment and higher perceived credibility [34,37]. Before–after photographs likely exert greater influence because they provide an immediate and easily interpretable representation of treatment outcomes, enabling viewers to visualize their own potential transformation. This mechanism is further supported by the findings of Karkun et al. [40], where participants who viewed digitally modified versions of their own smiles reported increased motivation to pursue orthodontic treatment. Previous studies have also highlighted the influential role of before–after orthodontic images shared on social media. Al Shayea et al. [44] found that 74% of participants preferred to see before–and–after case images from orthodontists on social media, identifying them as the most desirable form of content. In contrast, technical orthodontic posts, such as those featuring screws, elastics, or mechanical procedures, were associated with the lowest willingness and credibility ratings, suggesting that such content may be less engaging for lay audiences due to its limited esthetic appeal and the need for prior clinical knowledge to interpret it meaningfully [34,37].

Clear-aligner-related content appeared particularly effective in capturing patient interest, with participants frequently reporting that social media influenced their consideration of aligner therapy and drew attention to promotional posts [37,38,41]. This effect may reflect broader esthetic and lifestyle preferences, as aligners are often perceived as more discreet, convenient, and compatible with contemporary appearance-related expectations [38,41]. These findings are consistent with previous literature in esthetic dentistry and orthodontics. Abbasi et al. [45] reported that the orthodontic treatment with clear aligners is among the most requested esthetic dental treatments, with patients often seeking it due to its popularity on social media platforms. Similarly, Demir et al. [46] found that most participants first became aware of clear aligner therapy through social media, highlighting the role of these platforms in shaping patient awareness and treatment preferences. Collectively, this evidence supports the conclusion that aligner-related content, particularly when emphasizing esthetics and lifestyle compatibility, plays a significant role in influencing patient interest and treatment consideration.

A recurring pattern across the included studies indicated that women were more strongly affected by social media exposure than men, both in terms of willingness to pursue orthodontic treatment and responsiveness to specific content types [34,35,36,38]. This finding may be explained by differences in engagement with appearance-oriented content, as well as variations in esthetic priorities and social motivations. These observations are supported by previous research. Baik et al. [47] reported greater responsiveness to esthetic and outcome-oriented dental content among female participants. In addition, Nelson et al. [11] demonstrated that social media use is more prevalent among women, suggesting that increased exposure combined with greater attentiveness to esthetic cues may contribute to the stronger influence of social media observed in this group.

Instagram consistently emerged as the most influential social media platform, exerting a strong impact on awareness of orthodontic treatment possibilities and increasing motivation to pursue care [36,37,40,41]. This influence may be attributed to its image-centric format, which exposes users to a high volume of idealized or esthetically enhanced smiles, potentially fostering appearance-based self-comparison and motivating individuals to desire similar outcomes. This finding aligns with previous research on social media use in orthodontic care. Siddiqui et al. [16] reported that Instagram is among the most commonly used platforms for accessing orthodontic-related information. Similarly, Abbasi et al. [45] noted that dental professionals actively use Instagram to promote esthetic procedures, with such content significantly increasing patient awareness of available treatments. Collectively, these observations highlight the central role of Instagram as a platform through which orthodontic expectations and motivations are shaped.

### 4.2. Limitations

Despite its strengths, this review has several limitations that should be considered when interpreting its findings. The evidence base consisted primarily of cross-sectional studies relying on convenience sampling, which limits the generalizability of the results and restricts conclusions to associative rather than causal interpretations. Although such designs are appropriate for exploring perceptions and social media behaviors, they are inherently vulnerable to selection bias and cannot account fully for temporal or contextual influences on participants’ attitudes. Substantial variability in study aims, populations, exposure types, and outcome measures contributed to considerable methodological heterogeneity, which prevented statistical pooling and reduced the comparability of results across studies. Most included studies relied on self-administered questionnaires, which, although appropriate for assessing subjective experiences, remain vulnerable to recall and social desirability bias and may have affected the accuracy of reported social media use and perceived influence. Additionally, certain outcomes, such as credibility, were assessed in only a single study, limiting the strength of inferences in these domains and highlighting gaps in the existing literature. The evidence base was also geographically concentrated, with several studies conducted within specific cultural contexts, raising questions about the transferability of findings to broader populations. Finally, this review included only English-language publications, introducing the possibility of language bias and the omission of relevant studies published in other languages.

## 5. Conclusions

Social media appears to influence patients’ willingness to seek orthodontic treatment, although this effect is not consistent across individuals or content types. Visual content, particularly before–after images, was most strongly associated with increased willingness and perceived credibility, while female users demonstrated greater responsiveness to social media exposure. However, the current evidence base is limited and consists predominantly of cross-sectional studies, which limits causal inference. Therefore, these findings should be interpreted with caution. Further well-designed studies are required to better understand the extent and mechanisms of social media influence on orthodontic treatment decisions.

## Figures and Tables

**Table 1:** Databases searched (up to August 2025), strategies used, and hits per database.

Database	Search Strategy	Results
PubMed	(Orthodontic* OR “orthodontic treatment” OR braces OR “clear aligners” OR Invisalign) AND (“Social Media” OR “social network*” OR Facebook OR Instagram OR TikTok OR YouTube OR Twitter OR X OR Snapchat) AND (willing* OR motivat* OR intent* OR perception OR attitude* OR “decision making” OR preference* OR “patient acceptance”)	245
Scopus	(Orthodontic* OR “orthodontic treatment” OR braces OR “clear aligners” OR Invisalign) AND (“Social Media” OR “social network*” OR Facebook OR Instagram OR TikTok OR YouTube OR Twitter OR X OR Snapchat) AND (willing* OR motivat* OR intent* OR perception OR attitude* OR “decision making” OR preference* OR “patient acceptance”)	250
Science Direct	(Orthodontics OR “clear aligners”) AND (“Social Media” OR “social network” OR Facebook OR YouTube) AND (willingness OR motivation OR “decision making”)	289
Cochrane:	(Orthodontic* OR “orthodontic treatment” OR braces OR “clear aligners” OR Invisalign) AND (“Social Media” OR “social network*” OR Facebook OR Instagram OR TikTok OR YouTube OR Twitter OR X OR Snapchat) AND (willing* OR motivat* OR intent* OR perception OR attitude* OR “decision making” OR preference* OR “patient acceptance”)	112
ProQuest	(Orthodontic* OR “orthodontic treatment” OR braces OR “clear aligners” OR Invisalign) AND (“Social Media” OR “social network*” OR Facebook OR Instagram OR TikTok OR YouTube OR Twitter OR X OR Snapchat) AND (willing* OR motivat* OR intent* OR perception OR attitude* OR “decision making” OR preference* OR “patient acceptance”)	346

Asterisk (*) indicates truncation used to retrieve multiple word variations.

**Table 2:** The characteristics of the included studies in the systematic review.

Authors, Publication Year, Study Setting	Study Design	Population	Sample Size	Exposure	Social Media Platforms	Outcome	Main Findings
Alalola et al., 2025 [34] Saudi Arabia	Cross-sectional survey,	General public Adults ≥ 18 (F/M): 883/384 (70% female) Mostly aged 21–30 years	1267	Image-based orthodontic posts (before–after, intraoral, extraoral, and appliance images)	Snapchat TikTok Twitter Instagram LinkedIn Facebook	Willingness to seek orthodontic treatment	Before–after images showed the highest willingness (*p* < 0.05) Mechanical images showed the lowest willingness (*p* < 0.05) Females’ higher willingness for certain images. (*p* < 0.01) 50.7% would search social media to find an orthodontist
Al-Gunaid et al., 2021 [35] Saudi Arabia	Cross- sectional questionnaire	Ortho patients (F/M): 78/61 (56.1% female) 13–40 years old	139	Orthodontics info on SM	Twitter Snapchat Facebook YouTube Instagram WhatsApp Telegram	Self-reported impact of SM on decision	58.9% reported no direct impact (*p* > 0.05) Females showed greater influence than males (*p* < 0.01) 73.3% considered searching social media important before seeking orthodontic treatment; females significantly more than males (*p* < 0.001)
Diniz et al., 2025 [36] Brazil	Cross-sectional questionnaire	Ortho patients or seeking orthodontic treatment Adults ≥ 18 (F/M): 148/58 (71.8% female) Mean Age: 37.3	206	Orthodontic social media content	Instagram WhatsApp YouTube Facebook	Influence of SM on choice of orthodontist and acceptance of orthodontic treatment	Women considered before-and-after posts more important for their choice of an orthodontist than men. (*p* < 0.05) Instagram is the most influential platform in orthodontic decision-making and the selection of orthodontic treatment (*p* < 0.05)
Meira et al., 2021 [37] Brazil	Cross- sectional	Laypeople, dental students, dentists Adults (F/M): 276/170 Mean age 31.1	446 (225 laypeople 66 dental students 155 dentists)	12 Instagram post categories (before–after, educational, social)	Instagram	Willingness to become client and perceived credibility	Among laypeople: Before–after and teaching-related posts increased willingness (*p* < 0.05) Mechanical/technical posts showed lower perceived credibility Social and family-related posts showed the lowest credibility Esthetic orthodontic images were rated as more credible Teaching and before–after posts showed the highest professional credibility
Patil et al., 2025 [38] India	Cross-sectional questionnaire	Aligner users/interested Adults & adolescents (12- >40 years) (F/M): 189/111 63% female	300	Ads, promotions and information for clear aligners	All social media	Influence from social media to undergo aligner treatment	95% reported influence 62.33% highly influenced Women more influenced than men (*p* < 0.05) Instagram most influential platform (66.6%)
Sampson et al., 2021 [39] UK, Brazil	Cross sectional questionnaire	Adults >18 (F/M):24/15 Mean age: 33.8 Mostly aged 25–42 Ortho patients	39	Orthodontic social media content	Facebook Instagram Snapchat Twitter Linked in	Influence of SM on perception and acceptance of TADs	Exposure to mini-screw (TAD) content significantly increased willingness to undergo treatment (*p*< 0.05) 79.1% reported greater trust in clinicians with a social media presence
Karkun et al., 2023 [40] India	Randomized controlled trial (RCT)	Participants with mild to moderate malocclusion (F/M): 148/128 Age: Con: 24.13 ± 2.46 Exp: 23.94 ± 3.54	256 (128 control group-128 experimental group)	Experimental group: viewed their own corrected smile + ideal images on Instagram control group: viewed only ideal smiles.	Instagram	Desire to undergo orthodontic treatment	Higher Instagram influence scores (10-point scale) in the experimental group (7.09 ± 2.28 vs. 6.17 ± 1.42) (*p* < 0.05). Strong agreement on Instagram’s influence on treatment decision was higher in the experimental group (24.1% vs. 0.8%) (*p* < 0.05) Viewing ideal smiles followed by self-image changes encouraged treatment pursuit in both groups (91.4% vs. 75.9%) (*p*> 0.05) Earlier exposure to such images would have encouraged treatment initiation in both groups (92.1% vs. 81.2%) (*p* > 0.05)
Adanan et al., 2024 [41] Malaysia	Qualitative using semi-structured interviews	Seeking orthodontic treatment Adults ≥ 18 (F/M): 8/7 22–30 years	15	Orthodontic marketing content	Instagram Facebook	Perceived influence on decision-making	Increased awareness and motivation for orthodontic treatment following social media exposure Misinformation and fake content reduced trust Interest in clear aligner content Decision-making influenced by reviews, followers, endorsements, cost, and location

## Data Availability

No new data were created or analyzed in this study.

## Supplementary Materials

The following supporting information can be downloaded at: https://www.mdpi.com/article/10.3390/dj14050263/s1, Table S1: PRISMA 2020 Checklist; Table S2: Study Selection.

## Appendix A

**Table A1:** Risk of bias assessment of the cross-sectional studies using the Newcastle–Ottawa Scale modified for cross-sectional designs.

Domain	Reference	Alalola et al. (2023) [34]	Al-Gunaid et al. (2021) [35]	Diniz et al. (2025) [36]	Meira et al. (2021) [37]	Patil et al. (2025) [38]	Sampson et al. (2021) [39]
Selection	Representativeness of the sample			✵	✵		✵
	2. Sample size	✵			✵	✵	✵
	3. Non-respondents						
	4. Ascertainment of the exposure	✵✵	✵✵	✵	✵✵	✵✵	✵
Comparability	Comparability of cohorts based on the design or analysis controlled for confounders		✵	✵		✵	✵
Outcome	Assessment of the outcome	✵	✵	✵	✵	✵	✵
	2. Statistical test	✵	✵	✵	✵	✵	✵
Overall Score (Total stars)		5	5	5	6	6	6

Asterisk (✵) indicates that the criterion was met.

**Table A2:** Risk of bias assessment of the randomized controlled trial using the Revised Cochrane risk-of-bias tool for randomized trials (RoB 2).

Study	D1	D2	D3	D4	D5	Overall
Karkun et al., (2023) [40]	Some concerns	Some concerns	Some concerns	Some concerns	Some concerns	Some concerns

D1: Bias arising from the randomization process. D2: Bias due to deviations from intended intervention. D3: Bias due to missing outcome data. D4: Bias in measurement of the outcome. D5: Bias in selection of the reported result.

**Table A3:** Critical appraisal of the included qualitative study using the JBI Critical Appraisal Checklist for Qualitative Research.

JBI Criteria for Qualitative Research	Adanan et al. (2023) [41]
Q1. Congruity between philosophical perspective and methodology	Unclear
Q2. Congruity between methodology and research question	Yes
Q3. Congruity between methodology and data collection	Yes
Q4. Congruity between methodology and data analysis	Yes
Q5. Congruity between methodology and interpretation	Yes
Q6. Researcher culturally/theoretically located	No
Q7. Influence of researcher addressed	No
Q8. Participants’ voices adequately represented	Yes
Q9. Ethical approval evident	Yes
Q10. Conclusions flow from analysis	Yes
