# Supplementary material for: The Impact of Orthodontic-Related Social Media Content on Patients’ Willingness to Initiate Treatment: A Systematic Review
Source: Dent J (Basel). 2026 May 1;14(5):263. doi: 10.3390/dj14050263 (PMC13206082; doi:10.3390/dj14050263)
Supplement: Supplementary file 1 [file dentistry-14-00263-s001.zip › dentistry-4216952-supplementary.pdf]

Table S1. PRISMA 2020 Checklist

Section and Topic	Item #	Checklist item	Location where item is reported
**TITLE**			
Title	1	Identify the report as a systematic review.	1
**ABSTRACT**			
Abstract	2	See the PRISMA 2020 for Abstracts checklist.	1
**INTRODUCTION**			
Rationale	3	Describe the rationale for the review in the context of existing knowledge.	2
Objectives	4	Provide an explicit statement of the objective(s) or question(s) the review addresses.	3
**METHODS**			
Eligibility criteria	5	Specify the inclusion and exclusion criteria for the review and how studies were grouped for the syntheses.	3-4
Information sources	6	Specify all databases, registers, websites, organisations, reference lists and other sources searched or consulted to identify studies. Specify the date when each source was last searched or consulted.	4
Search strategy	7	Present the full search strategies for all databases, registers and websites, including any filters and limits used.	4, Table S1
Selection process	8	Specify the methods used to decide whether a study met the inclusion criteria of the review, including how many reviewers screened each record and each report retrieved, whether they worked independently, and if applicable, details of automation tools used in the process.	5
Data collection process	9	Specify the methods used to collect data from reports, including how many reviewers collected data from each report, whether they worked independently, any processes for obtaining or confirming data from study investigators, and if applicable, details of automation tools used in the process.	5
Data items	10a	List and define all outcomes for which data were sought. Specify whether all results that were compatible with each outcome domain in each study were sought (e.g. for all measures, time points, analyses), and if not, the methods used to decide which results to collect.	5
	10b	List and define all other variables for which data were sought (e.g. participant and intervention characteristics, funding sources). Describe any assumptions made about any missing or unclear information.	5
Study risk of bias assessment	11	Specify the methods used to assess risk of bias in the included studies, including details of the tool(s) used, how many reviewers assessed each study and whether they worked independently, and if applicable, details of automation tools used in the process.	5, 16-21
Effect measures	12	Specify for each outcome the effect measure(s) (e.g. risk ratio, mean difference) used in the synthesis or presentation of results.	5
Synthesis methods	13a	Describe the processes used to decide which studies were eligible for each synthesis (e.g. tabulating the study intervention characteristics and comparing against the planned groups for each synthesis (item #5)).	Not applicable
	13b	Describe any methods required to prepare the data for presentation or synthesis, such as handling of missing summary statistics, or data	Not

Section and Topic	Item #	Checklist item	Location where item is reported
		conversions.	applicable
	13c	Describe any methods used to tabulate or visually display results of individual studies and syntheses.	Not applicable
	13d	Describe any methods used to synthesize results and provide a rationale for the choice(s). If meta-analysis was performed, describe the model(s), method(s) to identify the presence and extent of statistical heterogeneity, and software package(s) used.	Not applicable
	13e	Describe any methods used to explore possible causes of heterogeneity among study results (e.g. subgroup analysis, meta-regression).	Not applicable
	13f	Describe any sensitivity analyses conducted to assess robustness of the synthesized results.	Not applicable
Reporting bias assessment	14	Describe any methods used to assess risk of bias due to missing results in a synthesis (arising from reporting biases).	Not applicable
Certainty assessment	15	Describe any methods used to assess certainty (or confidence) in the body of evidence for an outcome.	Not applicable
**RESULTS**			
Study selection	16a	Describe the results of the search and selection process, from the number of records identified in the search to the number of studies included in the review, ideally using a flow diagram.	6
	16b	Cite studies that might appear to meet the inclusion criteria, but which were excluded, and explain why they were excluded.	6, Table S2
Study characteristics	17	Cite each included study and present its characteristics.	6-10
Risk of bias in studies	18	Present assessments of risk of bias for each included study.	10
Results of individual studies	19	For all outcomes, present, for each study: (a) summary statistics for each group (where appropriate) and (b) an effect estimate and its precision (e.g. confidence/credible interval), ideally using structured tables or plots.	11-12
Results of syntheses	20a	For each synthesis, briefly summarise the characteristics and risk of bias among contributing studies.	10-11
	20b	Present results of all statistical syntheses conducted. If meta-analysis was done, present for each the summary estimate and its precision (e.g. confidence/credible interval) and measures of statistical heterogeneity. If comparing groups, describe the direction of the effect.	10-11
	20c	Present results of all investigations of possible causes of heterogeneity among study results.	10-11
	20d	Present results of all sensitivity analyses conducted to assess the robustness of the synthesized results.	10-11
Reporting biases	21	Present assessments of risk of bias due to missing results (arising from reporting biases) for each synthesis assessed.	Not

Section and Topic	Item #	Checklist item	Location where item is reported
			applicable
Certainty of evidence	22	Present assessments of certainty (or confidence) in the body of evidence for each outcome assessed.	11
**DISCUSSION**			
Discussion	23a	Provide a general interpretation of the results in the context of other evidence.	12-13
	23b	Discuss any limitations of the evidence included in the review.	13-14
	23c	Discuss any limitations of the review processes used.	14
	23d	Discuss implications of the results for practice, policy, and future research.	14
**OTHER INFORMATION**			
Registration and protocol	24a	Provide registration information for the review, including register name and registration number, or state that the review was not registered.	3
	24b	Indicate where the review protocol can be accessed, or state that a protocol was not prepared.	3
	24c	Describe and explain any amendments to information provided at registration or in the protocol.	3
Support	25	Describe sources of financial or non-financial support for the review, and the role of the funders or sponsors in the review.	14
Competing interests	26	Declare any competing interests of review authors.	15
Availability of data, code and other materials	27	Report which of the following are publicly available and where they can be found: template data collection forms; data extracted from included studies; data used for all analyses; analytic code; any other materials used in the review.	15

From: Page MJ, McKenzie JE, Bossuyt PM, Boutron I, Hoffmann TC, Mulrow CD, et al. The PRISMA 2020 statement: an updated guideline for reporting systematic reviews. BMJ 2021;372:n71. doi: 10.1136/bmj.n71

For more information, visit: http://www.prisma-statement.org/

Table S2. Study Selection

N = 1243<br>[245 + 113 + 289 +250 +346]				
**Database**				
**1. Medline (via PubMed)**				
**Search date**	24/8/2025			
**Results**	245			
**Search Query**	(Orthodontic* OR "orthodontic treatment" OR braces OR "clear aligners" OR Invisalign) AND ("Social Media" OR "social network*" OR Facebook OR Instagram OR TikTok OR YouTube OR Twitter OR X OR Snapchat) AND (willing* OR motivat* OR intent* OR perception OR attitude* OR "decision making" OR preference* OR "patient acceptance")			
**Nr.**	**Authors-Title**	**Eligibility**	**Reason for exclusion**	
1	Abdelkarim A, Sullivan D. Perspectives of dental students and faculty about evidence-based dental practice. J Evid Based Dent Pract. 2014 Dec;14(4):165-73. doi: 10.1016/j.jebdp.2014.06.001. Epub 2014 Jun 16. PMID: 25488865.	Excluded	Title/Abstract	

2	Abe T, Shibao Y, Takeuchi Y, Mataki Y, Amano K, Hioki S, Miura K, Noguchi H, Funayama T, Koda M, Yamazaki M. Initial hospitalization with rigorous bed rest followed by bracing and rehabilitation as an option of conservative treatment for osteoporotic vertebral fractures in elderly patients: a pilot one arm safety and feasibility study. Arch Osteoporos. 2018 Nov 23;13(1):134. doi: 10.1007/s11657-018-0547-0. PMID: 30470939; PMCID: PMC6267386.	Excluded	Title/Abstract	
3	Abutayyem H. In-Vitro Investigation of the Shear Bond Strength of Different Orthodontic Adhesives to Enamel. J Pharm Bioallied Sci. 2024 Jul;16(Suppl 3):S2473-S2475. doi: 10.4103/jpbs.jpbs_323_24. Epub 2024 May 22. PMID: 39346208; PMCID: PMC11426852.	Excluded	Title/Abstract	
4	Adeyemo YI, Karimi M, Oziegbe E, Popoola BO, Alade M, Shamshiri AR, Murtomaa HT, Oni TO, Eigbobo JO, Onyejaka NK, Olatosi OO, Nzomiwu CL, Malami AB, Chukwumah NM, Soyoye OA, Khami MR, Foláyan MO. Factors associated with dental clinic use by clients in Nigeria during the COVID-19 pandemic. BMC Oral Health. 2024 Jul 17;24(1):811. doi: 10.1186/s12903-024-04566-2. PMID: 39020315; PMCID: PMC11256443.	Excluded	Title/Abstract	
5	Adobes Martin M, Pérez Márquez A, Meuli S, Aguilera AC, Dioguardi M, Aiuto R, Garcovich D. User Experience, Satisfaction, and Complications of Direct-to-Consumer Orthodontics in Spain: A Cross-Sectional Study. J Clin Med. 2025 Mar 30;14(7):2382. doi: 10.3390/jcm14072382. PMID: 40217832; PMCID: PMC11989835.	Excluded	Title/Abstract	
6	Aflatooni JO, Loving R, Holderread BM, Liberman SR, Harris JD. #Scoliosis: an analysis of patient perception of scoliosis on TikTok. Proc (Bayl Univ Med Cent). 2023 Aug 25;36(6):671-674. doi: 10.1080/08998280.2023.2249371. PMID: 37829238; PMCID: PMC10566427.	Excluded	Title/Abstract	

7	Ahmed HMA, Obaid DH, Kadhum HI, Nahidh M, Russo D, Herford AS, Cicciù M, Minervini G. Awareness of orthodontic patients towards smartphone orthodontic apps. Minerva Dent Oral Sci. 2024 Jun;73(3):134-141. doi: 10.23736/S2724-6329.23.04853-2. Epub 2024 May 14. PMID: 38743249.	Excluded	Title/Abstract	
8	Ajwa N, Alomran A, Alsuwaie H, Alzamil K, Alfaifi R, Almasry N, Alali Z, Alanazi M. Dental Practitioners' Perception toward Management of Physically Challenged Patient among Saudi Arabia. J Int Soc Prev Community Dent. 2018 Nov-Dec;8(6):546-552. doi: 10.4103/jispcd.JISPCD_287_18. Epub 2018 Nov 29. PMID: 30596047; PMCID: PMC6280571.	Excluded	Title/Abstract	
9	Al-Gunaid TH, Aljohani AA, Alhazmi KM, Ibrahim AM. Determining the impact of orthodontic patients' characteristics on their usage and preferences of social media. J Taibah Univ Med Sci. 2020 Oct 16;16(1):16-21. doi: 10.1016/j.jtumed.2020.08.010. PMID: 33603627; PMCID: PMC7858013.	Excluded	Full text, irrelevant outcome	
10	Al-Haj Ali SN, Farah RI, Alhariqi S. Knowledge and Attitudes of Saudi Medical Students about Emergency Management of Traumatic Dental Injuries. Int J Environ Res Public Health. 2022 Oct 31;19(21):14249. doi: 10.3390/ijerph192114249. PMID: 36361130; PMCID: PMC9654410.	Excluded	Title/Abstract	
11	Al-Moghrabi D, Johal A, Fleming PS. What are people tweeting about orthodontic retention? A cross-sectional content analysis. Am J Orthod Dentofacial Orthop. 2017 Oct;152(4):516-522. doi: 10.1016/j.ajodo.2017.04.021. PMID: 28962737.	Excluded	Title/Abstract	
12	Al-Moghrabi D, Colonio-Salazar FB, Johal A, Fleming PS. Development of 'My Retainers' mobile application: Triangulation of two qualitative	Excluded	Title/Abstract	

	methods. J Dent. 2020 Mar;94:103281. doi: 10.1016/j.jdent.2020.103281. Epub 2020 Jan 24. PMID: 31987979.			
13	Al-Silwadi FM, Gill DS, Petrie A, Cunningham SJ. Effect of social media in improving knowledge among patients having fixed appliance orthodontic treatment: A single-center randomized controlled trial. Am J Orthod Dentofacial Orthop. 2015 Aug;148(2):231-7. doi: 10.1016/j.ajodo.2015.03.029. PMID: 26232831.	Excluded	Title/Abstract	
14	Alalola B, Alghanim L, Almoneef S, Aba Numay S, Aldghim A, Abu Wathlan J. The Impact of Orthodontists' Image-Based Social Media Posts on the Public's Willingness to Seek Treatment in the Central Region of Saudi Arabia. Patient Prefer Adherence. 2025 May 3;19:1255-1261. doi: 10.2147/PPA.S521608. PMID: 40337287; PMCID: PMC12057624.	Included		
15	Aldhuwayhi S, Mallineni SK, Sakhamuri S, Thakare AA, Mallineni S, Sajja R, Sethi M, Nettam V, Mohammad AM. Covid-19 Knowledge and Perceptions Among Dental Specialists: A Cross-Sectional Online Questionnaire Survey. Risk Manag Healthc Policy. 2021 Jul 7;14:2851-2861. doi: 10.2147/RMHP.S306880. PMID: 34262372; PMCID: PMC8274826.	Excluded	Title/Abstract	
16	Alhajj MN, Al-Maweri SA, Folayan MO, Halboub E, Khader Y, Omar R, Amran AG, Al-Batayneh OB, Celebić A, Persic S, Kocaelli H, Suleyman F, Alkheraif AA, Divakar DD, Mufadhal AA, Al-Wesabi MA, Alhajj WA, Aldumaini MA, Khan S, Al-Dhelai TA, Alqahtani AS, Murad AH, Makzoumé JE, Kohli S, Ziyad TA. Knowledge, beliefs, attitude, and practices of E-cigarette use among dental students: A multinational survey. PLoS One. 2022 Oct 27;17(10):e0276191. doi: 10.1371/journal.pone.0276191. PMID: 36301839; PMCID: PMC9612543.	Excluded	Title/Abstract	

17	Alhossine RM, Ibrahim RM. Impact of the COVID-19 Pandemic on Saudi Parents' Perceptions Toward Their Children's Oral Health: A Cross-Sectional Study. Cureus. 2024 Aug 18;16(8):e67125. doi: 10.7759/cureus.67125. PMID: 39156996; PMCID: PMC11330661.	Excluded	Title/Abstract	
18	Alkadhimi A. Does the exposure to 'ideal' facial images on Instagram influence facial and bodily satisfaction? Evid Based Dent. 2021 Jan;22(1):30-31. doi: 10.1038/s41432-021-0154-6. PMID: 33772130.	Excluded	Title/Abstract	
19	Alkhozaim DA, Al-Haj Ali SN, Farah RI. Levels and correlates of knowledge of teething among Saudi Arabian families. PeerJ. 2022 Aug 18;10:e13948. doi: 10.7717/peerj.13948. PMID: 35999852; PMCID: PMC9393006.	Excluded	Title/Abstract	
20	Allanqawi T, Alkadhimi A, Fleming PS. Postgraduate orthodontic education: An international perspective on content and satisfaction levels. J World Fed Orthod. 2023 Dec;12(6):239-244. doi: 10.1016/j.ejwf.2023.08.004. Epub 2023 Sep 20. PMID: 37739847.	Excluded	Title/Abstract	
21	Almotairy N. Public perception of the advertised claims of Damon® appliance system in Saudi Arabia: a cross-sectional survey-based study. BMC Oral Health. 2025 Apr 24;25(1):622. doi: 10.1186/s12903-025-06021-2. PMID: 40275289; PMCID: PMC12023427.	Excluded	Title/Abstract	
22	Almutairi M, Alomran I, Alshahrani R, Alsania R, Ali HA, Allam R, Alessa N, Hafiz Z. Child Abuse and Neglect: Awareness among Dental Students. Healthcare (Basel). 2023 Sep 11;11(18):2510. doi: 10.3390/healthcare11182510. PMID: 37761707; PMCID: PMC10531096.	Excluded	Title/Abstract	
23	AlQabandi AK, Sadowsky C, BeGole EA. A comparison of the effects of rectangular and round arch wires in leveling the curve of Spee. Am J Orthod Dentofacial Orthop. 1999 Nov;116(5):522-9. doi: 10.1016/s0889-5406(99)70183-6. PMID: 10547511.	Excluded	Title/Abstract	

24	Alqahtani H. Management of maxillary impacted canines: A prospective study of orthodontists' preferences. Saudi Pharm J. 2021 May;29(5):384-390. doi: 10.1016/j.jsps.2021.03.010. Epub 2021 Mar 31. PMID: 34135665; PMCID: PMC8180458.	Excluded	Title/Abstract	
25	Alrubaiaan R, Nair B, Amir-Rad F, Aljanahi M, Kumar S V, Prasad S. Presurgical Infant Orthopedic Videos on YouTube™: A Thematic Analysis of Caregiver Narratives. Cleft Palate Craniofac J. 2025 Jun;62(6):921-931. doi: 10.1177/10556656241233115. Epub 2024 Feb 23. PMID: 38389436.	Excluded	Title/Abstract	
26	Andrade NN, Kshirsagar MM, Andrade T, Bandgar VV, Pimpale S, Chavan A. Assessment of Knowledge, Attitude and Practices of Health Care Professionals Working in Jumbo COVID Care Center Towards Mental Health Care Needs: A Cross Sectional Survey. Adv Biomed Res. 2023 Jan 31;12:22. doi: 10.4103/abr.abr_295_20. PMID: 36926433; PMCID: PMC10012011.	Excluded	Title/Abstract	
27	An S, Lee JY, Chung CJ, Kim KH. Comparison of different midsagittal plane configurations for evaluating craniofacial asymmetry by expert preference. Am J Orthod Dentofacial Orthop. 2017 Dec;152(6):788-797. doi: 10.1016/j.ajodo.2017.04.024. PMID: 29173858.	Excluded	Title/Abstract	
28	An SY, Lee KM, Lee JS. Korean dentists' perceptions and attitudes regarding radiation safety and protection. Dentomaxillofac Radiol. 2018 Feb;47(3):20170228. doi: 10.1259/dmfr.20170228. Epub 2018 Jan 31. PMID: 29236521; PMCID: PMC6047635.	Excluded	Title/Abstract	
29	Arslan C, Aksahin EC, Nur Yılmaz RB, Germec Cakan D. Does YouTubeTM Offer High-Quality Information About Nasoalveolar Molding? Cleft Palate Craniofac J. 2024 Jan;61(1):5-11. doi: 10.1177/10556656221115025. Epub 2022 Jul 21. PMID: 35861787.	Excluded	Title/Abstract	

30	Arunachalam R, Nathwani N, Nejatian T, Fine P, Leung A. Assessing dentists' awareness of the orthodontic-restorative interface. J Dent. 2024 Feb;141:104811. doi: 10.1016/j.jdent.2023.104811. Epub 2023 Dec 21. PMID: 38141806.	Excluded	Title/Abstract	
31	Atchison KA, Luke LS, White SC. An algorithm for ordering pretreatment orthodontic radiographs. Am J Orthod Dentofacial Orthop. 1992 Jul;102(1):29-44. doi: 10.1016/0889-5406(92)70012-Y. PMID: 1307857.	Excluded	Title/Abstract	
32	Azizi F, Zaseh MMS, Golshah A, Imani MM, Safari-Faramani R. Comparative efficacy of pharmaceutical (Ibuprofen) and non-pharmaceutical (photobiomodulation, and chewing gum) interventions for pain reduction after elastomeric separator placement in orthodontic patients: a randomized clinical trial. Lasers Med Sci. 2024 Sep 25;39(1):239. doi: 10.1007/s10103-024-04186-w. PMID: 39317795.	Excluded	Title/Abstract	
33	Barber S, Bekker H, Marti J, Pavitt S, Khambay B, Meads D. Development of a Discrete-Choice Experiment (DCE) to Elicit Adolescent and Parent Preferences for Hypodontia Treatment. Patient. 2019 Feb;12(1):137-148. doi: 10.1007/s40271-018-0338-0. PMID: 30367434; PMCID: PMC6335368.	Excluded	Title/Abstract	
34	Batista NP, Bazett-Jones DM, Waiteman MC. Confidence and knowledge of Brazilian physiotherapists and physiotherapy students in managing patellofemoral pain. Musculoskelet Sci Pract. 2025 Jun;77:103305. doi: 10.1016/j.msksp.2025.103305. Epub 2025 Mar 8. PMID: 40090119.	Excluded	Title/Abstract	
35	Berretta LM, Gasparello GG, de Souza EM, Hartmann GC, Berretta EC, Camargo ES, Ignácio SA, MotohiroTanaka O. Analyzing patient perspectives on orthodontic treatment through social media hashtags. PLoS One. 2025 Aug 21;20(8):e0330263. doi: 10.1371/journal.pone.0330263. PMID: 40839641; PMCID: PMC12370105.	Excluded	Full text, irrelevant outcome	

36	Berry L, Jones A, Barber S. Laypeople's interpretation of, and preference for, orthodontic images. J Orthod. 2022 Sep;49(3):296-303. doi: 10.1177/14653125221085983. Epub 2022 Mar 24. PMID: 35323072; PMCID: PMC9421200.	Excluded	Title/Abstract	
37	Bhamrah G, Ahmad S, NiMhurchadha S. Internet discussion forums, an information and support resource for orthognathic patients. Am J Orthod Dentofacial Orthop. 2015 Jan;147(1):89-96. doi: 10.1016/j.ajodo.2014.08.020. PMID: 25533076.	Excluded	Title/Abstract	
38	Bhattacharya S, Chatterjee P, Dutta S. Common orthopaedic problems in day to day general practice (in economically handicapped community). J Indian Med Assoc. 2007 May;105(5):263-4, 266-7, 270. PMID: 17915795.	Excluded	Title/Abstract	
39	Bjerklin K, Bondemark L. Management of ectopic maxillary canines: variations among orthodontists. Angle Orthod. 2008 Sep;78(5):852-9. doi: 10.2319/070307-306.1. PMID: 18298198.	Excluded	Title/Abstract	
40	Bjerklin K, Bondemark L. Ectopic maxillary canines and root resorption of adjacent incisors. Does computed tomography (CT) influence decision-making by orthodontists? Swed Dent J. 2008;32(4):179-85. PMID: 19172919.	Excluded	Title/Abstract	
41	Bogdanov V, Ilova D, Yordanova G. Adolescent Patient's Perceptions During Treatment With Class II Elastics. Case Rep Dent. 2024 Aug 6;2024:1489397. doi: 10.1155/2024/1489397. PMID: 39139475; PMCID: PMC11321889.	Excluded	Title/Abstract	
42	Braun S, Brenneis M, Schönnagel L, Caffard T, Diaremes P. Surgical Treatment of Spinal Deformities in Pediatric Orthopedic Patients. Life (Basel). 2023 Jun 7;13(6):1341. doi: 10.3390/life13061341. PMID: 37374124; PMCID: PMC10302009.	Excluded	Title/Abstract	

43	Bullock L, Holden MA, Jinks C, Atiah Asamane E, Herron D, Borrelli B, Callaghan MJ, Birrell F, Halliday N, Marshall M, Sowden G, Ingram C, McBeth J, Dziedzic K, Foster NE, Jowett S, Lawton S, Mallen CD, Peat G. Physiotherapists' Experiences and Perceived Acceptability of Delivering a Knee Bracing Intervention for People With Symptomatic Knee Osteoarthritis in a Randomised Trial (PROP OA): A Qualitative Study. Musculoskeletal Care. 2024 Dec;22(4):e70021. doi: 10.1002/msc.70021. PMID: 39694859; PMCID: PMC11655417.	Excluded	Title/Abstract	
44	Bungău TC, Moca AE, Ciavoi G, Romanul IM, Vaida LL, Buhaș CL. Usage and Preferences of Orthodontic Mini-Implants Among Romanian Practitioners: A Survey Study. Dent J (Basel). 2024 Dec 6;12(12):400. doi: 10.3390/dj12120400. PMID: 39727457; PMCID: PMC11675009.	Excluded	Title/Abstract	
45	Cannatà D, Galdi M, Martina S, Rongo R, D'Antò V, Valletta R, Bucci R. Preformed Elastodontic Appliances: Awareness and Attitude of Orthodontists and General Dental Practitioners. Children (Basel). 2024 Apr 1;11(4):418. doi: 10.3390/children11040418. PMID: 38671635; PMCID: PMC11048748.	Excluded	Title/Abstract	
46	Casillas Santana MÁ, Martínez Zumarán A, Patiño Marín N, Castillo Silva BE, Sámano Valencia C, Salas Orozco MF. How Dentists Face the COVID-19 in Mexico: A Nationwide Cross-Sectional Study. Int J Environ Res Public Health. 2021 Feb 11;18(4):1750. doi: 10.3390/ijerph18041750. PMID: 33670181; PMCID: PMC7916932.	Excluded	Title/Abstract	
47	Castilhos JS, Gasparello GG, Mota-Júnior SL, Hartmann GC, Miyagusuku LFI, Pithon MM, Tanaka OM. Accessories in clear aligner therapy: Laypeople's expectations for comfort and satisfaction. J Dent Res Dent Clin Dent Prospects. 2024 Spring;18(2):102-109. doi:	Excluded	Title/Abstract	

	10.34172/joddd.40793. Epub 2024 Jun 24. PMID: 39071215; PMCID: PMC11282201.			
48	Cheung T, Park J, Lee D, Kim C, Olson J, Javadi S, Lawson G, McCabe J, Moon W, Ting K, Hong C. Ability of mini-implant-facilitated micro-osteoperforations to accelerate tooth movement in rats. Am J Orthod Dentofacial Orthop. 2016 Dec;150(6):958-967. doi: 10.1016/j.ajodo.2016.04.030. PMID: 27894545; PMCID: PMC5131371.	Excluded	Title/Abstract	
49	Coêlho TG, Caracas HC. Perception of the relationship between TMD and orthodontic treatment among orthodontists. Dental Press J Orthod. 2015 Jan-Feb;20(1):45-51. doi: 10.1590/2176-9451.20.1.045-051.oar. PMID: 25741824; PMCID: PMC4373015.	Excluded	Title/Abstract	
50	Condon SB, Veitch M. The experience of pregnancy associated osteoporosis: An international survey with implications for midwifery care. Midwifery. 2022 Dec;115:103468. doi: 10.1016/j.midw.2022.103468. Epub 2022 Aug 24. PMID: 36108340.	Excluded	Title/Abstract	
51	Craven J, Davies I, Perry DC. The role of weaning in brace treatment for developmental dysplasia of the hip : time to define best practice? Bone Jt Open. 2025 Jun 11;6(6):685-690. doi: 10.1302/2633-1462.66.BJO-2025-0053.R1. PMID: 40494538; PMCID: PMC12151612.	Excluded	Title/Abstract	
52	Crerand CE, Kapa HM, Litteral J, Da Silveira AC, Markey MK. Adherence to Orthodontic Treatment in Youth With Craniofacial Conditions: A Survey of US Orthodontists. Cleft Palate Craniofac J. 2019 Nov;56(10):1322-1332. doi: 10.1177/1055665619853132. Epub 2019 Jun 3. PMID: 31159560.	Excluded	Title/Abstract	

53	Cui Y, Huang T, Yu Z, Zhou S, Zhang L, Han Y, Li S, Wang Q, Tian Y. Mechanical characterization and structural analysis of elastodontic appliances under intraoral and artificial aging conditions. BMC Oral Health. 2024 Nov 1;24(1):1337. doi: 10.1186/s12903-024-05101-z. PMID: 39487402; PMCID: PMC11529478.	Excluded	Title/Abstract	
54	Cure RJ. Experiences of the challenges of undertaking an orthodontic needs assessment within the National Health Service in England. J Orthod. 2019 Jun;46(2):143-147. doi: 10.1177/1465312519842877. Epub 2019 May 7. PMID: 31060464.	Excluded	Title/Abstract	
55	Curto A, Albaladejo A, Montero J, Alvarado A. Influence of a Lubricating Gel (Orthospeed®) on Pain and Oral Health-Related Quality of Life in Orthodontic Patients during Initial Therapy with Conventional and Low-Friction Brackets: A Prospective Randomized Clinical Trial. J Clin Med. 2020 May 14;9(5):1474. doi: 10.3390/jcm9051474. PMID: 32423007; PMCID: PMC7290469.	Excluded	Title/Abstract	
56	Curto A, Mihit F, Curto D, Albaladejo A. Assessment of Orthodontic Treatment Need and Oral Health-Related Quality of Life in Asthmatic Children Aged 11 to 14 Years Old: A Cross-Sectional Study. Children (Basel). 2023 Jan 18;10(2):176. doi: 10.3390/children10020176. PMID: 36832305; PMCID: PMC9954178.	Excluded	Title/Abstract	
57	Curto A, Albaladejo A, Alvarado-Lorenzo A, Zubizarreta-Macho Á, Curto D. Cross-Bite and Oral-Health-Related Quality of Life (OHRQoL) in Preadolescents Aged 11 to 14 Years Old: A Pilot Case-Control Study. Children (Basel). 2023 Jul 29;10(8):1311. doi: 10.3390/children10081311. PMID: 37628310; PMCID: PMC10453585.	Excluded	Title/Abstract	

58	Dămăşaru MS, Păcurar M, Mariş M, Dămăşaru E, Mariş M, Tilinca CM. Implications of type 1 diabetes mellitus in the etiology and clinic of dento-maxillary anomalies - questionnaire-based evaluation of the dentists' opinion. Med Pharm Rep. 2025 Jan;98(1):135-143. doi: 10.15386/mpr-2822. Epub 2025 Jan 31. PMID: 39949908; PMCID: PMC11817580.	Excluded	Title/Abstract	
59	de Oliveira Silva YR, Zheng D, Peters SC, Fisher OS. Stabilization of a Cu-binding site by a highly conserved tryptophan residue. J Inorg Biochem. 2024 Apr;253:112501. doi: 10.1016/j.jinorgbio.2024.112501. Epub 2024 Feb 7. PMID: 38342077.	Excluded	Title/Abstract	
60	Debelmas A, Ketoff S, Lanciaux S, Corre P, Friess M, Khonsari RH. Reproducibility assessment of Delaire cephalometric analysis using reconstructions from computed tomography. J Stomatol Oral Maxillofac Surg. 2020 Feb;121(1):35-39. doi: 10.1016/j.jormas.2019.04.008. Epub 2019 May 2. PMID: 31055092.	Excluded	Title/Abstract	
61	Değirmencioğlu E, Kılıçoğlu H. Evaluating the impact of social media marketing from the perspective of orthodontists. BMC Oral Health. 2024 Jul 11;24(1):779. doi: 10.1186/s12903-024-04558-2. PMID: 38992712; PMCID: PMC11242013.	Excluded	Full text, Sample: orthodontists, irrelevant outcome	
62	Donzelli S, Zaina F, Minnella S, Lusini M, Negrini S. Consistent and regular daily wearing improve bracing results: a case-control study. Scoliosis Spinal Disord. 2018 Jul 28;13:16. doi: 10.1186/s13013-018-0164-0. PMID: 30065972; PMCID: PMC6064121.	Excluded	Title/Abstract	
63	Drohomyretska M, Tkachenko Y. THE METHOD OF ASSESSING THE DEGREE OF GLOSSOPTOSIS ACCORDING TO CLINICAL AND X-RAY ANTHROPOMETRICAL PREDICTORS: CLINICAL GUIDELINES. Georgian Med News. 2024 May;(350):57-62. PMID: 39089272.	Excluded	Title/Abstract	

64	Dyrna F, Imhoff FB, Haller B, Braun S, Obopilwe E, Apostolakos JM, Morikawa D, Imhoff AB, Mazzocca AD, Beitzel K. Primary Stability of an Acromioclavicular Joint Repair Is Affected by the Type of Additional Reconstruction of the Acromioclavicular Capsule. Am J Sports Med. 2018 Dec;46(14):3471-3479. doi: 10.1177/0363546518807908. Epub 2018 Nov 12. PMID: 30419178.	Excluded	Title/Abstract	
65	ElKarmi R, Aljafari A, Eldali H, Hosey MT. Do expectant mothers know how early childhood caries can be prevented? A cross-sectional study. Eur Arch Paediatr Dent. 2019 Dec;20(6):595-601. doi: 10.1007/s40368-019-00442-8. Epub 2019 Apr 19. PMID: 31004321.	Excluded	Title/Abstract	
66	Ertel AE, Robinson BR, Eckman MH. Cost-effectiveness of cervical spine clearance interventions with litigation and long-term-care implications in obtunded adult patients following blunt injury. J Trauma Acute Care Surg. 2016 Nov;81(5):897-904. doi: 10.1097/TA.0000000000001243. PMID: 27602907.	Excluded	Title/Abstract	
67	Espeland LV, Ivarsson K, Stenvik A. A new Norwegian index of orthodontic treatment need related to orthodontic concern among 11-year-olds and their parents. Community Dent Oral Epidemiol. 1992 Oct;20(5):274-9. doi: 10.1111/j.1600-0528.1992.tb01698.x. PMID: 1424547.	Excluded	Title/Abstract	
68	Ey-Chmielewska H. Próba zastosowania techniki ultrasonograficznej do potwierdzenia diagnozowania, planowania i obserwacji odległych wyników leczenia dysfunkcji bólowych stawów skroniowo-zuchwowych [An attempt to use ultrasonic technique for confirming the diagnosis, planning and observation of long-term treatment results of painful temporo-mandibular joint dysfunction]. Ann Acad Med Stetin. 1998;44:223-36. Polish. PMID: 9857541.	Excluded	Title/Abstract	

69	Felizardo R, Thomas A, Foucart JM. Techniques radiographiques utiles en orthodontie [Useful radiological techniques in orthodontics]. Orthod Fr. 2012 Mar;83(1):11-22. French. doi: 10.1051/orthodfr/2012003. Epub 2012 Mar 29. PMID: 22455647.	Excluded	Title/Abstract	
70	Fischer-Brandies H, Orthuber W, Ermert M, Hussmanns A. The force module for the bending art system. Preliminary results. J Orofac Orthop. 1998;59(5):301-11. English, German. doi: 10.1007/BF01321796. PMID: 9800444.	Excluded	Title/Abstract	
71	Forkel P, Imhoff AB, Achtnich A, Willinger L. Arthroskopische Refixation tibialer knöcherner Avulsionsverletzungen des hinteren Kreuzbands mit Faden-Button-Konstrukt [All-arthroscopic fixation of tibial posterior cruciate ligament avulsion fractures with a suture-button technique]. Oper Orthop Traumatol. 2020 Jun;32(3):236-247. German. doi: 10.1007/s00064-019-00626-x. Epub 2019 Sep 6. PMID: 31492968.	Excluded	Title/Abstract	
72	Fraiwan M, Al-Kofahi N, Ibnian A, Hanatleh O. Detection of developmental dysplasia of the hip in X-ray images using deep transfer learning. BMC Med Inform Decis Mak. 2022 Aug 13;22(1):216. doi: 10.1186/s12911-022-01957-9. PMID: 35964072; PMCID: PMC9375244.	Excluded	Title/Abstract	
73	Frawley T, Parkin N, Kettle J, Longstaff S, Benson P. Young people's experiences of orthodontic retainers: A qualitative study. J Orthod. 2022 Dec;49(4):394-402. doi: 10.1177/14653125221099962. Epub 2022 May 25. PMID: 35611854; PMCID: PMC9679557.	Excluded	Title/Abstract	
74	Fukuzawa T, Oba H, Ikegami S, Uehara M, Hatakenaka T, Kurogochi D, Sasao S, Shigenobu K, Makiyama F, Koseki M, Takahashi J. Cobb angle estimation without X-ray using angle of trunk rotation and characteristics of unpredictable cases. Eur Spine J. 2025 Aug 23. doi: 10.1007/s00586-025-09263-4. Epub ahead of print. PMID: 40848157.	Excluded	Title/Abstract	

75	Gandedkar NH, Lee BP, Pangrazio-Kulbersh V, Alansari R, Castillo JL, Cruz RM, Çakan DG, Foong KW, Park JH, Park YG, Dellinger A, Perillo L, Thom AR, Vaiid N. World Federation of Orthodontists social media guidelines: Ensuring accuracy, reliability, and objectivity in online orthodontic information. J World Fed Orthod. 2025 Aug;14(4):187-193. doi: 10.1016/j.ejwf.2025.06.002. Epub 2025 Jul 14. PMID: 40664563.	Excluded	Title/Abstract	
76	Gasparello GG, Mota-Júnior SL, Hartmann GC, Berlesi AH, Acciaris F, Berretta LM, Pithon MM, Tanaka O. Orthodontics social media, perceptions of science- and non-science-based posts among orthodontists, dentists, students and laypeople. PLoS One. 2023 Sep 29;18(9):e0286927. doi: 10.1371/journal.pone.0286927. PMID: 37773974; PMCID: PMC10540967.	Excluded	Full text, irrelevant outcome	
77	Gizani S, Seremidi K, Gkourtsogianni S, Mitsea A. Awareness and practice of dentomaxillofacial imaging among paediatric dentists: a questionnaire survey of members of the European Academy of Paediatric Dentistry. Oral Radiol. 2023 Jul;39(3):576-587. doi: 10.1007/s11282-023-00675-w. Epub 2023 Mar 3. PMID: 36867317.	Excluded	Title/Abstract	
78	Glotzbecker MP, Wasser AM, Troy MJ, Proctor M, Emans JB. Neonatal C1 TO C2 osteomyelitis leading to instability and neurological decline: novel treatment with occiput-C1-C2 fusion and occiput to thorax growing rods. A case report. J Pediatr Orthop. 2015 Jun;35(4):379-84. doi: 10.1097/BPO.0000000000000309. PMID: 25171676.	Excluded	Title/Abstract	
79	Gomes JP, Costa AL, Chone CT, Altemani AM, Altemani JM, Lima CS. Three-dimensional volumetric analysis of ghost cell odontogenic carcinoma using 3-D reconstruction software: a case report. Oral Surg Oral Med Oral Pathol Oral Radiol. 2017 May;123(5):e170-e175. doi: 10.1016/j.oooo.2017.01.012. Epub 2017 Feb 6. PMID: 28407991.	Excluded	Title/Abstract	

80	González-Sáez A, Antonio-Zancajo L, Montero J, Albaladejo A, Melo M, Garcovich D, Alvarado-Lorenzo A. The Influence of Friction on Design of the Type of Bracket and Its Relation to OHRQoL in Patients Who Use Multi-Bracket Appliances: A Randomized Clinical Trial. Medicina (Kaunas). 2021 Feb 17;57(2):171. doi: 10.3390/medicina57020171. PMID: 33671217; PMCID: PMC7922052.	Excluded	Title/Abstract	
81	Gornitzky AL, Georgiadis AG, Seeley MA, Horn BD, Sankar WN. Does Perfusion MRI After Closed Reduction of Developmental Dysplasia of the Hip Reduce the Incidence of Avascular Necrosis? Clin Orthop Relat Res. 2016 May;474(5):1153-65. doi: 10.1007/s11999-015-4387-6. PMID: 26092677; PMCID: PMC4814438.	Excluded	Title/Abstract	
82	Gracco A, Cozzani M, D'Elia L, Manfrini M, Peverada C, Siciliani G. The smile buccal corridors: aesthetic value for dentists and laypersons. Prog Orthod. 2006;7(1):56-65. English, Italian. PMID: 16552456.	Excluded	Title/Abstract	
83	Graf I, Gerwing H, Hoefer K, Ehlebracht D, Christ H, Braumann B. Social media and orthodontics: A mixed-methods analysis of orthodontic-related posts on Twitter and Instagram. Am J Orthod Dentofacial Orthop. 2020 Aug;158(2):221-228. doi: 10.1016/j.ajodo.2019.08.012. Epub 2020 Jun 20. PMID: 32576427.	Excluded	Full text, irrelevant outcome	
84	Graf I, Kruse T, Braumann B, Hoefer K, Ehlebracht D. Looking good but tweeting bad? The social perception of orthodontic-related posts on Twitter and Instagram. Head Face Med. 2022 Feb 17;18(1):6. doi: 10.1186/s13005-021-00302-1. PMID: 35177104; PMCID: PMC8851782.	Excluded	Title/Abstract	
85	Gross RT, Ghaltakhchyan N, Nanney EM, Jackson TH, Wiesen CA, Mihas P, Persky AM, Frazier-Bowers SA, Jacox LA. Evaluating video-based lectures on YouTube for dental education. Orthod Craniofac Res.	Excluded	Title/Abstract	

	2023 Dec;26 Suppl 1(Suppl 1):210-220. doi: 10.1111/ocr.12669. Epub 2023 May 15. PMID: 37184946; PMCID: PMC10646151.			
86	Grünheid T, Sudit GN, Larson BE. Debonding and adhesive remnant cleanup: an in vitro comparison of bond quality, adhesive remnant cleanup, and orthodontic acceptance of a flash-free product. Eur J Orthod. 2015 Oct;37(5):497-502. doi: 10.1093/ejo/cju080. Epub 2014 Dec 29. PMID: 25548144.	Excluded	Title/Abstract	
87	Guan J, Bisson EF. Treatment of Odontoid Fractures in the Aging Population. Neurosurg Clin N Am. 2017 Jan;28(1):115-123. doi: 10.1016/j.nec.2016.07.001. PMID: 27886873.	Excluded	Title/Abstract	
88	Guo F, Tang B, Qin D, Zhao T, Su YX, McGrath C, Hua F, He H. The Impact of the COVID-19 Epidemic on Orthodontic Patients in China: An Analysis of Posts on Weibo. Front Med (Lausanne). 2020 Dec 8;7:577468. doi: 10.3389/fmed.2020.577468. PMID: 33364245; PMCID: PMC7753180.	Excluded	Title/Abstract	
89	Guo J, Yan X, Li S, Van der Walt J, Guan G, Mei L. Quantitative and qualitative analyses of orthodontic-related videos on YouTube. Angle Orthod. 2020 May 1;90(3):411-418. doi: 10.2319/082019-542.1. PMID: 33378439; PMCID: PMC8032311.	Excluded	Title/Abstract	
90	Halimi A, Zaoui F. Etude comparative des mesures d'encombrement postérieur entre la radiographie panoramique dentaire et le scanner dentaire [Comparative study of measurement of posterior tooth crowding between the dental panoramic xray and dental scanner]. Odontostomatol Trop. 2012 Mar;35(137):13-20. French. PMID: 22715639.	Excluded	Title/Abstract	
91	Haney E, Gansky SA, Lee JS, Johnson E, Maki K, Miller AJ, Huang JC. Comparative analysis of traditional radiographs and cone-beam computed tomography volumetric images in the diagnosis and treatment	Excluded	Title/Abstract	

	planning of maxillary impacted canines. Am J Orthod Dentofacial Orthop. 2010 May;137(5):590-7. doi: 10.1016/j.ajodo.2008.06.035. PMID: 20451777.			
92	Harris AB, Oni JK. Cementless, Cruciate-Retaining Primary Total Knee Arthroplasty Using Conventional Instrumentation: Technical Pearls and Intraoperative Considerations. JBJS Essent Surg Tech. 2024 Sep 13;14(3):e23.00036. doi: 10.2106/JBJS.ST.23.00036. PMID: 39280965; PMCID: PMC11392501.	Excluded	Title/Abstract	
93	Hegarty E, Campbell C, Grammatopoulos E, DiBiase AT, Sherriff M, Cobourne MT. YouTube™ as an information resource for orthognathic surgery. J Orthod. 2017 Jun;44(2):90-96. doi: 10.1080/14653125.2017.1319010. Epub 2017 May 2. PMID: 28463076.	Excluded	Title/Abstract	
94	Helms JA, Speidel TM, Denis KL. Effect of timing on long-term clinical success of alveolar cleft bone grafts. Am J Orthod Dentofacial Orthop. 1987 Sep;92(3):232-40. doi: 10.1016/0889-5406(87)90417-3. PMID: 3307380.	Excluded	Title/Abstract	
95	Hembree M, Buschang PH, Carrillo R, Spears R, Rossouw PE. Effects of intentional damage of the roots and surrounding structures with miniscrew implants. Am J Orthod Dentofacial Orthop. 2009 Mar;135(3):280.e1-9; discussion 280-1. doi: 10.1016/j.ajodo.2008.06.022. PMID: 19268823.	Excluded	Title/Abstract	
96	Henzell M, Knight A, Antoun JS, Farella M. Social media use by orthodontic patients. N Z Dent J. 2013 Dec;109(4):130-3. PMID: 24396951.	Excluded	no access to full text	
97	Hou S, Zhou T, Liu Y, Dang P, Lu H, Shi H. Teeth U-Net: A segmentation model of dental panoramic X-ray images for context semantics and contrast enhancement. Comput Biol Med. 2023 Jan;152:106296. doi: 10.1016/j.compbiomed.2022.106296. Epub 2022 Nov 12. PMID: 36462370.	Excluded	Title/Abstract	

98	Hua F, Qin D, Yan J, Zhao T, He H. COVID-19 Related Experience, Knowledge, Attitude, and Behaviors Among 2,669 Orthodontists, Orthodontic Residents, and Nurses in China: A Cross-Sectional Survey. Front Med (Lausanne). 2020 Aug 7;7:481. doi: 10.3389/fmed.2020.00481. PMID: 32850923; PMCID: PMC7427309.	Excluded	Title/Abstract	
99	Hugo B, Witzel T, Klaiber B. Comparison of in vivo visual and computer-aided tooth shade determination. Clin Oral Investig. 2005 Dec;9(4):244-50. doi: 10.1007/s00784-005-0014-3. Epub 2005 Oct 8. PMID: 16215749.	Excluded	Title/Abstract	
100	Hussain SR, Jiang SS, Bosio JA. Generational perspectives of orthodontists in the U.S. and Canada: A survey study. Am J Orthod Dentofacial Orthop. 2022 Dec;162(6):824-838. doi: 10.1016/j.ajodo.2021.07.020. Epub 2022 Aug 31. PMID: 36055883.	Excluded	Title/Abstract	
101	Hussein A, Bataineh M, Khader Y, Al-Batayneh OB. Knowledge, and treatment of molar incisor hypomineralisation (MIH) among dentists in Jordan: a cross-sectional questionnaire based study. Eur Arch Paediatr Dent. 2025 Apr;26(2):247-254. doi: 10.1007/s40368-024-00953-z. Epub 2024 Oct 30. Erratum in: Eur Arch Paediatr Dent. 2025 Aug;26(4):825. doi: 10.1007/s40368-025-01053-2. PMID: 39476170.	Excluded	Title/Abstract	
102	Hyam DM. The contemporary management of third molars. Aust Dent J. 2018 Mar;63 Suppl 1:S19-S26. doi: 10.1111/adj.12587. PMID: 29574809.	Excluded	Title/Abstract	
103	Isfeld D, Lagravere M, Leon-Salazar V, Flores-Mir C. Novel methodologies and technologies to assess mid-palatal suture maturation: a systematic review. Head Face Med. 2017 Jun 14;13(1):13. doi: 10.1186/s13005-017-0144-2. PMID: 28615034; PMCID: PMC5471738.	Excluded	Title/Abstract	
104	Jaffray DC, Eisenstein SM, Balain B, Trivedi JM, Newton Ede M. Early mobilisation of thoracolumbar burst fractures without neurology: a	Excluded	Title/Abstract	

	natural history observation. Bone Joint J. 2016 Jan;98-B(1):97-101. doi: 10.1302/0301-620X.98B1.36121. PMID: 26733521.			
105	Jasim ES, Alnuaimy NSM, Abid M, Dziedzic A. Orthodontic practice marketing: The orthodontist and laypeople's perspective. J Orthod Sci. 2024 Nov 25;13:47. doi: 10.4103/jos.jos_37_24. PMID: 39758113; PMCID: PMC11698251.	Excluded	Full text, irrelevant outcome	
106	Jedliński M, Mazur M, Schmeidl K, Grocholewicz K, Ardan R, Janiszewska-Olszowska J. Orthodontic Retention-Protocols and Materials-A Questionnaire Pilot Study among Polish Practitioners. Materials (Basel). 2022 Jan 16;15(2):666. doi: 10.3390/ma15020666. PMID: 35057382; PMCID: PMC8779968.	Excluded	Title/Abstract	
107	Joham SJ, Hadzic A, Urschler M. Implicit Is Not Enough: Explicitly Enforcing Anatomical Priors inside Landmark Localization Models. Bioengineering (Basel). 2024 Sep 17;11(9):932. doi: 10.3390/bioengineering11090932. PMID: 39329674; PMCID: PMC11428392.	Excluded	Title/Abstract	
108	Jones JW, McCullough LB. Ethics of treating postoperative pain. J Vasc Surg. 2012 Feb;55(2):583-4. doi: 10.1016/j.jvs.2011.12.036. PMID: 22264808.	Excluded	Title/Abstract	
109	Jorge OS, Leite JR, Lotto M, Cruvinel T. A study of Facebook comments regarding amber teething necklaces: insights into public perception. Eur Arch Paediatr Dent. 2025 Feb;26(1):71-82. doi: 10.1007/s40368-024-00956-w. Epub 2024 Nov 6. PMID: 39503825.	Excluded	Title/Abstract	
110	Jorge OS, Remiro MDS, Lotto M, Zakir Hussain I, Moreira MAA, Morita PP, Cruvinel T. Unveiling deception: Characterizing false amber necklace messages on Facebook. Int J Paediatr Dent. 2024 May;34(3):302-312. doi: 10.1111/ipd.13121. Epub 2023 Sep 13. PMID: 37705197.	Excluded	Title/Abstract	

111	Jorge OS, Remiro MODS, Lotto M, Cruvinel T. Exploring the factors driving higher interactions in false amber necklace posts on Facebook. Braz Oral Res. 2024 Aug 5;38:e067. doi: 10.1590/1807-3107bor-2024.vol38.0067. PMID: 39109764; PMCID: PMC11376611.	Excluded	Title/Abstract	
112	Jucá AM, Santana Jorge O, Moreira YR, Lotto M, Sá Menezes T, Cruvinel T. Uncovering a pseudoscience: an analysis of 'biological dentistry' Instagram posts. Acta Odontol Scand. 2024 Apr 24;83:180-189. doi: 10.2340/aos.v83.40486. PMID: 38656559; PMCID: PMC11302399.	Excluded	Title/Abstract	
113	Kamarudin Y, Mohd Nor NA, Libamin AC, Suriani ANH, Marhazlinda J, Bramantoro T, Ramadhani A, Neville P. Social media use, professional behaviors online, and perceptions toward e-professionalism among dental students. J Dent Educ. 2022 Aug;86(8):958-967. doi: 10.1002/jdd.12912. Epub 2022 Mar 5. PMID: 35247218.	Excluded	Title/Abstract	
114	Karkazi F, Antoniadou M, Demeterová K, Konstantonis D, Margaritis V, Lysy J. Orthodontic Risk Perspectives among Orthodontists during Treatment: A Descriptive Pilot Study in Greece and Slovakia. Healthcare (Basel). 2024 Feb 18;12(4):492. doi: 10.3390/healthcare12040492. PMID: 38391867; PMCID: PMC10887888.	Excluded	Title/Abstract	
115	Karkun R, Batra P, Singh AK. Influence of social media and corrected smile photographs in patients with malocclusion. Am J Orthod Dentofacial Orthop. 2023 Nov;164(5):712-727. doi: 10.1016/j.ajodo.2023.04.021. Epub 2023 Jul 4. PMID: 37409990.	Included		
116	Kenealy PM, Kingdon A, Richmond S, Shaw WC. The Cardiff dental study: a 20-year critical evaluation of the psychological health gain from orthodontic treatment. Br J Health Psychol. 2007 Feb;12(Pt 1):17-49. doi: 10.1348/135910706X96896. PMID: 17288664.	Excluded	Title/Abstract	

117	Kilinc ME, Sandefur EP, Zainab M, Peterman NJ, Yu-Shan AA, Apel PJ. Impact of the COVID-19 Pandemic on Buckle Fracture Treatment. J Pediatr Soc North Am. 2025 Mar 13;11:100162. doi: 10.1016/j.jposna.2025.100162. PMID: 40432869; PMCID: PMC12088341.	Excluded	Title/Abstract	
118	Kimura K, Langland OE, Biggerstaff RH. The evaluation of high-speed screen/film combinations in cephalometric radiography. Am J Orthod Dentofacial Orthop. 1987 Dec;92(6):484-91. doi: 10.1016/0889-5406(87)90230-7. PMID: 3479894.	Excluded	Title/Abstract	
119	Kim HW, Weinstein SL. Spine update. The management of scoliosis in neurofibromatosis. Spine (Phila Pa 1976). 1997 Dec 1;22(23):2770-6. doi: 10.1097/00007632-199712010-00014. PMID: 9431613.	Excluded	Title/Abstract	
120	Kirbschus A, Gesch D, Kaduk W, Gedrange T. The influence of craniofacial growth in a case of transverse facial cleft. J Orofac Orthop. 2006 May;67(3):215-24. English, German. doi: 10.1007/s00056-006-0602-0. PMID: 16736122.	Excluded	Title/Abstract	
121	Knorst JK, Brondani B, Vettore MV, Hesse D, Mendes FM, Ardenghi TM. Pathways between Social Capital and Oral Health from Childhood to Adolescence. J Dent Res. 2022 Sep;101(10):1155-1164. doi: 10.1177/00220345221094510. Epub 2022 May 20. PMID: 35593509.	Excluded	Title/Abstract	
122	Knorst JK, Vettore MV, Brondani B, Emmanuelli B, Tomazoni F, Ardenghi TM. Sense of coherence moderates the relationship between social capital and oral health-related quality of life in schoolchildren: a 10-year cohort study. Health Qual Life Outcomes. 2022 Apr 2;20(1):56. doi: 10.1186/s12955-022-01965-3. PMID: 35366896; PMCID: PMC8976264.	Excluded	Title/Abstract	
123	Knorst JK, Vettore MV, Brondani B, Emmanuelli B, Ardenghi TM. The Different Roles of Structural and Cognitive Social Capital on Oral Health-Related Quality of Life among Adolescents. Int J Environ Res Public	Excluded	Title/Abstract	

	Health. 2023 Apr 21;20(8):5603. doi: 10.3390/ijerph20085603. PMID: 37107885; PMCID: PMC10138599.			
124	Knösel M, Jung K. Informational value and bias of videos related to orthodontics screened on a video-sharing Web site. Angle Orthod. 2011 May;81(3):532-9. doi: 10.2319/091710-541.1. Epub 2011 Jan 24. PMID: 21261492; PMCID: PMC8923562.	Excluded	Title/Abstract	
125	Knösel M, Engelke W, Helms HJ, Bleckmann A. An appraisal of the current and potential value of web 2.0 contributions to continuing education in oral implantology. Eur J Dent Educ. 2012 Aug;16(3):131-7. doi: 10.1111/j.1600-0579.2011.00732.x. Epub 2012 Jan 12. PMID: 22783839.	Excluded	Title/Abstract	
126	Koppert E, Yi KH. Observational Study of Facial Rotation Patterns. J Craniofac Surg. 2025 Mar 28. doi: 10.1097/SCS.0000000000011278. Epub ahead of print. PMID: 40152590.	Excluded	Title/Abstract	
127	Kuroshima K, Miyazaki S, Hiranaka Y, Ryu M, Inoue S, Yurube T, Kakutani K, Tadokoro K. Risk Factors for Nonunion After Nonoperative Treatment for Pediatric Lumbar Spondylolysis: A Retrospective Case-Control Study. Am J Sports Med. 2024 Sep;52(11):2866-2873. doi: 10.1177/03635465241270293. Epub 2024 Sep 2. PMID: 39221535.	Excluded	Title/Abstract	
128	Kusaibati AM, Sultan K, Hajeer MY, Burhan AS, Alam MK. Adult patient expectations and satisfaction: Can they be influenced by viewing the three-dimensional predicted outcome before fixed orthodontic treatment of dental crowding? J World Fed Orthod. 2023 Dec;12(6):269-279. doi: 10.1016/j.ejwf.2023.08.005. Epub 2023 Sep 28. PMID: 37777351.	Excluded	Title/Abstract	
129	Lee KJC, Yong CW, Li H, Lai WMC, Chew MT, Ren Y. "Accuracy of bone-borne versus tooth-bone-borne orthognathic surgical guides: A	Excluded	Title/Abstract	

	systematic review and meta-analysis". J Dent. 2025 Oct;161:105940. doi: 10.1016/j.jdent.2025.105940. Epub 2025 Jul 4. PMID: 40618986.			
130	Lexner MO, Almer L. Case series: Treatment considerations in x-linked hypohidrotic ectodermal dysplasia. Eur Arch Paediatr Dent. 2009 Nov;10 Suppl 1:26-30. doi: 10.1007/BF03262696. PMID: 19863895.	Excluded	Title/Abstract	
131	Liamruk P, Onwong N, Amornrat K, Arayapipatkul A, Sipiyaruk K. Development and evaluation of an augmented reality serious game to enhance 21st century skills in cultural tourism. Sci Rep. 2025 Apr 18;15(1):13492. doi: 10.1038/s41598-025-95615-5. PMID: 40251186; PMCID: PMC12008235.	Excluded	Title/Abstract	
132	Liu C, Du S, Wang Z, Guo S, Cui M, Zhai Q, Zhang M, Fang B. Impact of orthodontic-induced facial morphology changes on aesthetic evaluation: a retrospective study. BMC Oral Health. 2024 Jan 5;24(1):24. doi: 10.1186/s12903-023-03776-4. PMID: 38183059; PMCID: PMC10768126.	Excluded	Title/Abstract	
133	Liu X, Chen S, Tsoi JKH, Matinlinna JP. Binary titanium alloys as dental implant materials-a review. Regen Biomater. 2017 Oct;4(5):315-323. doi: 10.1093/rb/rbx027. Epub 2017 Sep 23. PMID: 29026646; PMCID: PMC5633690.	Excluded	Title/Abstract	
134	Liu X, Luo Y, He X, Wang J, Li Z, Zhang Y, Hu X, Lu M, Tang F, Zhou Y, Min L, Tu C. [Three-dimensional-printed hemi-pelvic prosthesis for revision of aseptic loosening or screw fracture of modular hemi-pelvic prosthesis]. Zhongguo Xiu Fu Chong Jian Wai Ke Za Zhi. 2023 Oct 15;37(10):1183-1189. Chinese. doi: 10.7507/1002-1892.202306073. PMID: 37848311; PMCID: PMC10581870.	Excluded	Title/Abstract	

135	Longstaff S, Davies K, Benson P. Exploring 10-15-year-old patients' perspectives of fixed orthodontic treatment. J Orthod. 2021 Jun;48(2):110-117. doi: 10.1177/1465312520981077. Epub 2021 Feb 11. PMID: 33573439.	Excluded	Title/Abstract	
136	Lotto M, Aguirre PEA, Strieder AP, Cruvinel AFP, Cruvinel T. Levels of toothache-related interests of Google and YouTube users from developed and developing countries over time. PeerJ. 2019 Oct 10;7:e7706. doi: 10.7717/peerj.7706. PMID: 31616582; PMCID: PMC6790224.	Excluded	Title/Abstract	
137	Lotto M, Sá Menezes T, Zakir Hussain I, Tsao SF, Ahmad Butt Z, P Morita P, Cruvinel T. Characterization of False or Misleading Fluoride Content on Instagram: Infodemiology Study. J Med Internet Res. 2022 May 19;24(5):e37519. doi: 10.2196/37519. PMID: 35588055; PMCID: PMC9164089.	Excluded	Title/Abstract	
138	Lotto M, Jorge OS, Machado MAAM, Cruvinel T. Exploring online oral health misinformation: a content analysis. Braz Oral Res. 2023 May 29;37:e049. doi: 10.1590/1807-3107bor-2023.vol37.0049. PMID: 37255069.	Excluded	Title/Abstract	
139	Lotto M, Zakir Hussain I, Kaur J, Butt ZA, Cruvinel T, Morita PP. Analysis of Fluoride-Free Content on Twitter: Topic Modeling Study. J Med Internet Res. 2023 Jun 20;25:e44586. doi: 10.2196/44586. PMID: 37338975; PMCID: PMC10337345.	Excluded	Title/Abstract	
140	Luk JK, Tsang RC, Leung HB. Lateral epicondylalgia: midlife crisis of a tendon. Hong Kong Med J. 2014 Apr;20(2):145-51. doi: 10.12809/hkmj134110. Epub 2014 Feb 28. PMID: 24584568.	Excluded	Title/Abstract	
141	Luk KD, Saw LB, Grozman S, Cheung KM, Samartzis D. Assessment of skeletal maturity in scoliosis patients to determine clinical management: a new classification scheme using distal radius and ulna radiographs. Spine J. 2014 Feb 1;14(2):315-25. doi: 10.1016/j.spinee.2013.10.045. Epub 2013 Nov 12. PMID: 24239801.	Excluded	Title/Abstract	

142	Machado GM, Braga MM. Users' passivity in accessing digested scientific evidence through social media: cross-sectional insights. BMC Res Notes. 2022 Jun 23;15(1):218. doi: 10.1186/s13104-022-06089-x. PMID: 35739581; PMCID: PMC9229917.	Excluded	Title/Abstract	
143	Mani SA, Uma E, John J, Nieminen P. Perceptions of professional social media interaction with patients and faculty members - a comparative survey among dental students from Malaysia and Finland. BMC Med Educ. 2023 May 25;23(1):384. doi: 10.1186/s12909-023-04359-1. PMID: 37231460; PMCID: PMC10214545.	Excluded	Title/Abstract	
144	Manosudprasit A, Haghi A, Allareddy V, Masoud MI. Diagnosis and treatment planning of orthodontic patients with 3-dimensional dentofacial records. Am J Orthod Dentofacial Orthop. 2017 Jun;151(6):1083-1091. doi: 10.1016/j.ajodo.2016.10.037. PMID: 28554454.	Excluded	Title/Abstract	
145	Markusi M, Blagec T, Šimunović L, Lapter Varga M, Meštrović S. Awareness and usage of mobile applications as an orthodontic diagnostic tool among Croatian orthodontic clinicians. J Orthod. 2024 Jun;51(2):130-136. doi: 10.1177/14653125231217307. Epub 2023 Dec 28. PMID: 38153087.	Excluded	Title/Abstract	
146	Mattei TA, Hanovnikian J, H Dinh D. Progressive kyphotic deformity in comminuted burst fractures treated non-operatively: the Achilles tendon of the Thoracolumbar Injury Classification and Severity Score (TLICS). Eur Spine J. 2014 Nov;23(11):2255-62. doi: 10.1007/s00586-014-3312-0. Epub 2014 May 14. PMID: 24823845.	Excluded	Title/Abstract	
147	Ma Q, Kobayashi E, Jin S, Masamune K, Suenaga H. 3D evaluation model of facial aesthetics based on multi-input 3D convolution neural networks for orthognathic surgery. Int J Med Robot. 2024 Jun;20(3):e2651. doi: 10.1002/rcs.2651. PMID: 38872448.	Excluded	Title/Abstract	

148	Meira TM, Prestes J, Gasparello GG, Antelo OM, Pithon MM, Tanaka OM. The effects of images posted to social media by orthodontists on public perception of professional credibility and willingness to become a client. Prog Orthod. 2021 Mar 8;22(1):7. doi: 10.1186/s40510-021-00353-9. PMID: 33682012; PMCID: PMC7937582.	Included		
149	Menezes TDS, Martini MM, Lotto M, Jucá AM, Jorge OS, Cruvinel A, Cruvinel T. Untangling the truth: User engagement with misinformation in toothache-related Facebook posts. Community Dent Health. 2024 May 31;41(2):128-133. doi: 10.1922/CDH_00190Menezes06. PMID: 38682571.	Excluded	Title/Abstract	
150	Menezes TS, Martini MM, Lotto M, Jorge OS, Jucá AM, Aguirre PEA, Cruvinel T. Factors driving misinformation production and user engagement with toothache content on Facebook. Health Informatics J. 2024 Oct-Dec;30(4):14604582241274282. doi: 10.1177/14604582241274282. PMID: 39607870.	Excluded	Title/Abstract	
151	Menezes TS, Jucá AM, Jorge OS, Lotto M, Ayala Aguirre PE, Cruvinel T. Unraveling dental caries misinformation: Identifying predictive factors for engagement on Instagram. Digit Health. 2024 Dec 12;10:20552076241299642. doi: 10.1177/20552076241299642. PMID: 39669393; PMCID: PMC11635861.	Excluded	Title/Abstract	
152	Mengi A, Singh RP, Mengi N, Kalgotra S, Singh A. A questionnaire study regarding knowledge, attitude and usage of artificial intelligence and machine learning by the orthodontic fraternity of Northern India. J Oral Biol Craniofac Res. 2024 Sep-Oct;14(5):500-506. doi: 10.1016/j.jobcr.2024.06.004. Epub 2024 Jul 2. PMID: 39050525; PMCID: PMC11263740.	Excluded	Title/Abstract	

153	Montebugnoli F, Incerti Parenti S, D'Antò V, Alessandri-Bonetti G, Michelotti A. Effect of verbal and written information on pain perception in patients undergoing fixed orthodontic treatment: a randomized controlled trial. Eur J Orthod. 2020 Nov 3;42(5):494-499. doi: 10.1093/ejo/cjz068. PMID: 31504390.	Excluded	Title/Abstract	
154	Montes ABM, de Oliveira TM, Gavião MBD, de Souza Barbosa T. Occlusal, chewing, and tasting characteristics associated with orofacial dysfunctions in children with unilateral cleft lip and palate: a case-control study. Clin Oral Investig. 2018 Mar;22(2):941-950. doi: 10.1007/s00784-017-2173-4. Epub 2017 Jul 31. PMID: 28761982.	Excluded	Title/Abstract	
155	Nakano H, Satoh K, Norris R, Jin T, Kamegai T, Ishikawa F, Katsura H. Mechanical properties of several nickel-titanium alloy wires in three-point bending tests. Am J Orthod Dentofacial Orthop. 1999 Apr;115(4):390-5. doi: 10.1016/s0889-5406(99)70257-x. PMID: 10194282.	Excluded	Title/Abstract	
156	Nassani MZ, Alsalhani A, Alali FM, Rastam S, Alqhtani NR, Alqahtahni AS, Robaian A, Alhedyan FS, Bin Nabhan A, Alenazi A, Alqahtani KA, Alrafedah A, Abbas Alleft AA, Alnufaiy B, Alshenaiber R, Alghabban RO, Alagla M, Abuelqomsan MAS, Al-Joukhadar M, Al Zahed N, Darwish S, Sioufi A, Shamsy E, Kujan O, Noushad M, Al-Maweri SA, Binrayes A, Sabbagh B, Tarakji B. Public Awareness and Knowledge of Oral Cancer in 13 Middle Eastern and North African Countries. JAMA Netw Open. 2025 Mar 3;8(3):e250522. doi: 10.1001/jamanetworkopen.2025.0522. PMID: 40048163; PMCID: PMC11886726.	Excluded	Title/Abstract	
157	Nasseh I, Al-Rawi W. Cone Beam Computed Tomography. Dent Clin North Am. 2018 Jul;62(3):361-391. doi: 10.1016/j.cden.2018.03.002. PMID: 29903556.	Excluded	Title/Abstract	

158	Nawar NN, Elashiry MM, El Banna A, Saber SM, Schäfer E. Ex-vivo evaluation of clinically-set hydraulic sealers used with different canal dryness protocols and obturation techniques: a randomized clinical trial. Clin Oral Investig. 2024 Oct 27;28(11):612. doi: 10.1007/s00784-024-06006-5. PMID: 39463194; PMCID: PMC11513728.	Excluded	Title/Abstract	
159	Nehru A, Nagesh S. Smartphone-based 3D facial scanning applications in orthodontic diagnosis: A cross-sectional study. J Dent Res Dent Clin Dent Prospects. 2025 Jun 30;19(2):97-103. doi: 10.34172/joddd.025.42109. PMID: 40843122; PMCID: PMC12366668.	Excluded	Title/Abstract	
160	Nelson KL, Shroff B, Best AM, Lindauer SJ. Orthodontic marketing through social media networks: the patient and practitioner's perspective. Angle Orthod. 2015 Nov;85(6):1035-41. doi: 10.2319/110714-797.1. Epub 2015 Mar 4. PMID: 25738740; PMCID: PMC8612047.	Excluded	Full text, sample:orthodontists, ongoing tx in pts	
161	Nevins ML, Gartner-Sekler JL. Periodontal, implant, and prosthetic treatment for advanced periodontal disease. Compend Contin Educ Dent. 1997 May;18(5):469-74, 476, 478-9; quiz 480. PMID: 9533359.	Excluded	Title/Abstract	
162	Nieminen P, Uma E, Mani SA, John J, Laitala ML, Lappalainen OP. E-Professionalism among Dental Students from Malaysia and Finland. Int J Environ Res Public Health. 2022 Mar 9;19(6):3234. doi: 10.3390/ijerph19063234. PMID: 35328921; PMCID: PMC8949338.	Excluded	Title/Abstract	
163	Nouri M, Asefi S, Akbarzadeh Baghban A, Ahmadvand M, Shamsa M. Objective vs subjective analyses of arch form and preformed archwire selection. Am J Orthod Dentofacial Orthop. 2016 Apr;149(4):543-54. doi: 10.1016/j.ajodo.2015.09.023. PMID: 27021459.	Excluded	Title/Abstract	
164	Ocak I, Aksu M. Effects of curing lights on polymerization shrinkage of composite attachments in clear aligner treatment: A microcomputed	Excluded	Title/Abstract	

	tomography study. Am J Orthod Dentofacial Orthop. 2025 Jan;167(1):63-72. doi: 10.1016/j.ajodo.2024.08.012. Epub 2024 Oct 2. PMID: 39352331.			
165	Oliveira LM, Zanatta FB. Self-reported dental treatment needs during the COVID-19 outbreak in Brazil: an infodemiological study. Braz Oral Res. 2020 Sep 4;34:e114. doi: 10.1590/1807-3107bor-2020.vol34.0114. PMID: 32901729.	Excluded	Title/Abstract	
166	Ongkosuwito EM, Kuijpers MAR. HOW PRO'S CAN CONTRIBUTE TO WHAT MATTERS MOST TO PATIENTS WITH OROFACIAL CLEFTS. J Evid Based Dent Pract. 2023 Jan;23(1S):101792. doi: 10.1016/j.jebdp.2022.101792. Epub 2022 Oct 17. PMID: 36707166.	Excluded	Title/Abstract	
167	Oteri G, Panzarella V, Marcianò A, Di Fede O, Maniscalco L, Peditto M, Campisi G. Appropriateness in Dentistry: A Survey Discovers Improper Procedures in Oral Medicine and Surgery. Int J Dent. 2018 Apr 4;2018:3245324. doi: 10.1155/2018/3245324. PMID: 30123271; PMCID: PMC6079469.	Excluded	Title/Abstract	
168	Patil AS, Kshirsagar AR, Handa S. Is Social Media Influencing Patients to Choose Aligner Treatment? Cureus. 2025 Jun 22;17(6):e86523. doi: 10.7759/cureus.86523. PMID: 40698221; PMCID: PMC12282307.	Included		
169	Patural H, Nieto-Sánchez I, Rossi C, Templier L, Martin-Palomino-Sahagún P, Díaz-Renovales I. Esthetic comparison of two composites used for Invisalign's attachments bonding. J Clin Exp Dent. 2024 Aug 1;16(8):e940-e946. doi: 10.4317/jced.61853. PMID: 39281793; PMCID: PMC11392440.	Excluded	Title/Abstract	
170	Pei Y, Shi F, Chen H, Wei J, Zha H, Jiang R, Xu T. Personalized tooth shape estimation from radiograph and cast. IEEE Trans Biomed Eng.	Excluded	Title/Abstract	

	2012 Sep;59(9):2400-11. doi: 10.1109/TBME.2011.2174993. Epub 2011 Nov 7. PMID: 22084040.			
171	Pittet LF, Moore CL, McDonald E, Barry S, Bonten M, Campbell J, Croda J, Dalcolmo M, Davidson A, Douglas MW, Gardiner K, Gwee A, Jardim B, Lacerda MVG, Lucas M, Lynn DJ, Manning L, de Oliveira RD, Perrett KP, Prat-Aymerich C, Richmond PC, Rocha JL, Rodriguez-Baño J, Warris A, Wood NJ, Messina NL, Curtis N; BRACE Trial Consortium Group. Bacillus Calmette-Guérin vaccination for protection against recurrent herpes labialis: a nested randomised controlled trial. EClinicalMedicine. 2023 Sep 11;64:102203. doi: 10.1016/j.eclinm.2023.102203. PMID: 37719417; PMCID: PMC10500555.	Excluded	Title/Abstract	
172	Prithiviraj D, Siddiqui NR, Smyth RS, Hodges SJ, Sharif MO. The awareness and usage of orthodontic apps and social media by orthodontists in the UK: A questionnaire-based study. J Orthod. 2023 Mar;50(1):9-17. doi: 10.1177/14653125221094334. Epub 2022 May 1. PMID: 35491614.	Excluded	Title/Abstract	
173	Qabool H, Hamid F, Sukhia RH. Acceptance of SARS-CoV-2 vaccination and the associated factors among dental health care professionals: A cross-sectional survey. Dent Med Probl. 2022 Jan-Mar;59(1):21-26. doi: 10.17219/dmp/145491. PMID: 35138698.	Excluded	Title/Abstract	
174	Rachel Henzell M, Margaret Knight A, Morgaine KC, Antoun JS, Farella M. A qualitative analysis of orthodontic-related posts on Twitter. Angle Orthod. 2014 Mar;84(2):203-7. doi: 10.2319/051013-355.1. Epub 2013 Aug 28. PMID: 23984992; PMCID: PMC8673785.	Excluded	Title/Abstract	
175	Rajeh MT, Sembawa SN, Nassar AA, Al Hebshi SA, Aboalshamat KT, Badri MK. Social media as a learning tool: Dental students' perspectives.	Excluded	Title/Abstract	

	J Dent Educ. 2021 Apr;85(4):513-520. doi: 10.1002/jdd.12478. Epub 2020 Nov 20. PMID: 33219515.			
176	Rampon FB, Nóbrega C, Bretos JL, Arsati F, Jakob S, Jimenez-Pellegrin MC. Profile of the orthodontist practicing in the State of São Paulo--part 2. Dental Press J Orthod. 2013 Feb 15;18(1):32.e1-6. doi: 10.1590/s2176-94512013000100008. PMID: 23876966.	Excluded	Title/Abstract	
177	Rava A, Fusini F, Cinnella P, Massè A, Girardo M. Is cast an option in the treatment of thoracolumbar vertebral fractures? J Craniovertebr Junction Spine. 2019 Jan-Mar;10(1):51-56. doi: 10.4103/jcvjs.JCVJS_8_19. PMID: 31000982; PMCID: PMC6469317.	Excluded	Title/Abstract	
178	Rebaque Pistoni T, de la Cruz Pérez J, Nieto Sánchez I. Influence of social media on the esthetic perception of the lip profile of orthodontic patients. Heliyon. 2023 May 10;9(5):e15870. doi: 10.1016/j.heliyon.2023.e15870. PMID: 37251874; PMCID: PMC10209334.	Excluded	Title/Abstract	
179	Remiro MODS, Jorge OS, Lotto M, Lourenço Neto N, Machado MAAM, Cruvinel T. Reacting, Sharing, and Commenting: How Many Facebook Users Are Engaging with Posts Related to Dental Caries That Contain Misinformation? Caries Res. 2023;57(5-6):575-583. doi: 10.1159/000531014. Epub 2023 May 23. PMID: 37231798.	Excluded	Title/Abstract	
180	Ribeiro AA, Purger F, Rodrigues JA, Oliveira PR, Lussi A, Monteiro AH, Alves HD, Assis JT, Vasconcellos AB. Influence of contact points on the performance of caries detection methods in approximal surfaces of primary molars: an in vivo study. Caries Res. 2015;49(2):99-108. doi: 10.1159/000368562. PMID: 25572115.	Excluded	Title/Abstract	
181	Rizkalla JM, Lines T, Daoud Y, Zide J. Instagram and Pilon Fractures: An Analysis of Social Media and Its Relationship to Patient Injury	Excluded	Title/Abstract	

	Perception. Foot Ankle Spec. 2022 Feb;15(1):43-49. doi: 10.1177/1938640020940837. Epub 2020 Jul 20. PMID: 32686495.			
182	Rizk M, Häck S, Brenji S, Knaup I, Niederau C, Kiessling F, Marx N, Moellmann J, Kahles F, Pufe T, Apel C, Kern J, Seitz J, Käver L, Voelz C, Wolf M, Trinh S, Craveiro RB. Preventive effect of probiotics on periodontal ligament in a rat model of anorexia nervosa. Sci Rep. 2025 Jun 2;15(1):19287. doi: 10.1038/s41598-025-02610-x. PMID: 40456862; PMCID: PMC12130194.	Excluded	Title/Abstract	
183	Rodrigues GF, Carneiro ÍM, da Costa TC, Marques PAC, Barja-Fidalgo F, Fonseca-Gonçalves A. Influence of knowledge about silver diamine fluoride on the acceptance and perception of Brazilian children's guardians: a randomized controlled clinical trial. Eur Arch Paediatr Dent. 2025 Aug;26(4):769-777. doi: 10.1007/s40368-025-01040-7. Epub 2025 Apr 19. PMID: 40252190.	Excluded	Title/Abstract	
184	Rouas P, Bandon D, Nancy J, Delbos Y, Hauret L, Bar D. La tomographie volumétrique numérisée par le système NewTom: intérêt de ce nouvel examen d'imagerie médicale chez l'enfant [Digital volume tomography using the NewTom system: advantages of this new technique in children]. Arch Pediatr. 2006 Aug;13(8):1169-77. French. doi: 10.1016/j.arcped.2006.05.005. Epub 2006 Jul 21. PMID: 16860545.	Excluded	Title/Abstract	
185	Saatci P, Arli AO, Demir K, Saatci AO, Kavakçu S. Corneal involvement in Papillon-Lefèvre syndrome. J Pediatr Ophthalmol Strabismus. 2006 May-Jun;43(3):167-9. doi: 10.3928/01913913-20060301-05. PMID: 16761638.	Excluded	Title/Abstract	
186	Saccomanno S, Saran S, Guercio E, Mastrapasqua RF, Pirino A, Scoppa F. The Influence of the COVID-19 Pandemic on Orthodontic Treatments: A Survey Analysis. Dent J (Basel). 2022 Jan 20;10(2):15. doi: 10.3390/dj10020015. PMID: 35200241; PMCID: PMC8871191.	Excluded	Title/Abstract	

187	Salari F, Rafizadeh SM, Fakhredin H, Rajabi MT, Yaseri M, Hosseini F, Fekrazad R, Salari B. Prediction of substantial closed-globe injuries in orbital wall fractures. Int Ophthalmol. 2024 May 7;44(1):219. doi: 10.1007/s10792-024-03113-w. PMID: 38713333.	Excluded	Title/Abstract	
188	Salek F, El Idrissi I, El Alloussi M, Zaoui F, Azaroual MF. Corono-radicular dilaceration of a maxillary central incisor: A case report. Int Orthod. 2019 Sep;17(3):606-612. doi: 10.1016/j.ortho.2019.06.023. Epub 2019 Jun 25. PMID: 31253455.	Excluded	Title/Abstract	
189	Sampson A, Jeremiah HG, Andiappan M, Newton JT. The effect of viewing idealised smile images versus nature images via social media on immediate facial satisfaction in young adults: A randomised controlled trial. J Orthod. 2020 Mar;47(1):55-64. doi: 10.1177/1465312519899664. Epub 2020 Feb 7. PMID: 32031041.	Excluded	Title/Abstract	
190	Sampson A, Figueiredo DSF, Jeremiah HG, Oliveira DD, Freitas LRP, Chahoud M, Soares RV, Cobourne MT. The effect of social media on patient acceptance of temporary anchorage devices. Angle Orthod. 2021 May 1;91(3):363-370. doi: 10.2319/071020-618.1. PMID: 33461218; PMCID: PMC8084471.	Included		
191	Sandham HJ, Nadeau L, Phillips HI. The effect of chlorhexidine varnish treatment on salivary mutans streptococcal levels in child orthodontic patients. J Dent Res. 1992 Jan;71(1):32-5. doi: 10.1177/00220345920710010501. PMID: 1740553.	Excluded	Title/Abstract	
192	Sapata DM, Oliveira E Silva C, Pascotto RC, Poleti TMFF, Arai MSI, Ramos AL. Periodontal indexes of two types of 3 x 3 retainers: 0.032-in SS V-loop versus 0.0215-in SS coaxial - a randomized crossover trial. Dental Press J Orthod. 2024 Jan 8;28(6):e2323175. doi: 10.1590/2177-6709.28.6.e2323175.oar. PMID: 38198390; PMCID: PMC10773445.	Excluded	Title/Abstract	

193	Scheurer PA, Firestone AR, Bürgin WB. Perception of pain as a result of orthodontic treatment with fixed appliances. Eur J Orthod. 1996 Aug;18(4):349-57. doi: 10.1093/ejo/18.4.349. PMID: 8921656.	Excluded	Title/Abstract	
194	Schumacher HA, Bourauel C, Drescher D. Frictional forces when rectangular guiding arches with varying edge bevel are employed. J Orofac Orthop. 1998;59(3):139-49. English, German. doi: 10.1007/BF01317175. PMID: 9640000.	Excluded	Title/Abstract	
195	Sefton JM, Hicks-Little CA, Koceja DM, Cordova ML. Modulation of soleus H-reflex by presynaptic spinal mechanisms during varying surface and ankle brace conditions. Neurophysiol Clin. 2007 Jan-Mar;37(1):15-21. doi: 10.1016/j.neucli.2007.01.007. Epub 2007 Feb 9. PMID: 17418353.	Excluded	Title/Abstract	
196	Serrano-Velasco D, Martín-Vacas A, Paz-Cortés MM, Giovannini G, Cintora-López P, Aragoneses JM. Intraoral scanners in children: evaluation of the patient perception, reliability and reproducibility, and chairside time-A systematic review. Front Pediatr. 2023 Jun 26;11:1213072. doi: 10.3389/fped.2023.1213072. PMID: 37435173; PMCID: PMC10331299.	Excluded	Title/Abstract	
197	Serrano-Velasco D, Martín-Vacas A, Cintora-López P, Paz-Cortés MM, Aragoneses JM. Comparative Analysis of the Comfort of Children and Adolescents in Digital and Conventional Full-Arch Impression Methods: A Crossover Randomized Trial. Children (Basel). 2024 Feb 2;11(2):190. doi: 10.3390/children11020190. PMID: 38397302; PMCID: PMC10886968.	Excluded	Title/Abstract	
198	Shaffer SW, Uhl TL. Preventing and treating lower extremity stress reactions and fractures in adults. J Athl Train. 2006 Oct-Dec;41(4):466-9. PMID: 17273474; PMCID: PMC1748425.	Excluded	Title/Abstract	

199	Shahrul AI, Pauzzi ER, Abas NDA, Yahya NA, Khan KAM, Nor MM. To assess the knowledge, awareness, and perception of non-dentists offering orthodontic treatment among the Malaysian young adult population. BMC Public Health. 2024 Nov 20;24(1):3224. doi: 10.1186/s12889-024-20460-6. PMID: 39567923; PMCID: PMC11577906.	Excluded	Title/Abstract	
200	Sharif MO, Siddiqui NR, Hodges SJ. Patient awareness of orthodontic mobile phone apps. J Orthod. 2019 Mar;46(1):51-55. doi: 10.1177/1465312518821361. Epub 2019 Jan 29. PMID: 31056066.	Excluded	Title/Abstract	
201	Shaw T, Sergent A, Richardson M. Diagnosis and management of medial epicondyle avulsion fracture in a 27-year-old female: A case report. J Bodyw Mov Ther. 2022 Jan;29:23-25. doi: 10.1016/j.jbmt.2021.09.028. Epub 2021 Oct 9. PMID: 35248275.	Excluded	Title/Abstract	
202	Sheikh S, Pallagatti S, Singla I, Gupta R, Aggarwal A, Singh R, Gupta D. Survey of dental radiographical practice in states of Punjab and Haryana in India. J Investig Clin Dent. 2014 Feb;5(1):72-7. doi: 10.1111/jicd.12016. Epub 2012 Dec 11. PMID: 23233351.	Excluded	Title/Abstract	
203	Shinde S, Kumari S, Prakash M, M PG, Khajuria A, Modi N, Chhabra GK, Paul P. A Cross-Sectional Survey on the Use of Tobacco, Attempts on Cessation, and Locus of Control Among College Students. Cureus. 2023 Sep 29;15(9):e46195. doi: 10.7759/cureus.46195. PMID: 37905250; PMCID: PMC10613342.	Excluded	Title/Abstract	
204	Siddiqui N, Chia M, Sharif MO. Social media and orthodontics: Are our patients scrolling? J Orthod. 2022 Jun;49(2):179-184. doi: 10.1177/14653125211042025. Epub 2021 Sep 6. PMID: 34488483; PMCID: PMC9161434.	Excluded	Full text, irrelevant outcome	
205	Silveira GS, de Gauw JH, Mucha JN. Hypoplastic Canine: Would Treatment Decision be Different if the Diagnosis Were Made with	Excluded	Title/Abstract	

	Computed Tomography? Int J Orthod Milwaukee. 2016 Autumn;27(3):77-82. PMID: 30178948.			
206	Simpson S, Wallace CK, Vernazza CR. Paediatric dentistry provision in the North East of England: workforce confidence and attitudes. Br Dent J. 2022 Mar 18:1–7. doi: 10.1038/s41415-022-4045-9. Epub ahead of print. Erratum in: Br Dent J. 2022 Apr;232(7):474. doi: 10.1038/s41415-022-4148-3. PMID: 35304592; PMCID: PMC8932095.	Excluded	Title/Abstract	
207	Sin M, Butt S, Barber SK. Assessing dentist and dental student knowledge of and attitudes towards shared decision-making in the United Kingdom. Eur J Dent Educ. 2021 Nov;25(4):768-777. doi: 10.1111/eje.12656. Epub 2021 Jan 20. PMID: 33386681.	Excluded	Title/Abstract	
208	Sohrabi A, Babay Ahari S, Moslemzadeh H, Rafighi A, Aghazadeh Z. The reliability of clinical decisions based on the cervical vertebrae maturation staging method. Eur J Orthod. 2016 Feb;38(1):8-12. doi: 10.1093/ejo/cjv030. Epub 2015 Apr 28. PMID: 25925767.	Excluded	Title/Abstract	
209	Špiljak B, Šimunović L, Miličević AM, Granić M, Bergman L, Peršec J. Knowledge, Awareness, and Influence of the COVID-19 Pandemic on Students of Biomedical Faculties: A Cross-Sectional Study. Dent J (Basel). 2025 Jan 10;13(1):28. doi: 10.3390/dj13010028. PMID: 39851604; PMCID: PMC11763484.	Excluded	Title/Abstract	
210	Ståhl L, Sabel N, Naoumova J. Laypersons' esthetic assessment of teeth with de- or hypomineralization - a web-based survey. Acta Odontol Scand. 2025 Aug 19;84:446-456. doi: 10.2340/aos.v84.44231. PMID: 40827308.	Excluded	Title/Abstract	
211	Stupar I, Yetkiner E, Attin T, Attin R. Influence of Lateral Cephalometric Radiography on Treatment Planning and Preferences in Skeletal Open-Bite Patients: Do Lateral Cephalograms Influence Treatment Planning?	Excluded	Title/Abstract	

	Turk J Orthod. 2016 Dec;29(4):87-90. doi: 10.5152/TurkJOrthod.2016.1609. Epub 2016 Dec 1. PMID: 30112480; PMCID: PMC6007604.			
212	Subramaniam MH, Moirangthem V, Venkatesan M. Management of Spinal Langerhans Cell Histiocytosis in Children: A Systematic Review. Int J Spine Surg. 2024 Nov 19;18(6):769–80. doi: 10.14444/8662. Epub ahead of print. PMID: 39562044; PMCID: PMC11687042.	Excluded	Title/Abstract	
213	Sytek L, Inglehart MR, Ramaswamy V, Aronovich S, Edwards S, Kim-Berman H. Comparisons of orthodontic residents' performance and attitudes using 2D, 3D, and virtual reality surgical simulation methods. J Dent Educ. 2021 Aug;85(8):1415-1426. doi: 10.1002/jdd.12598. Epub 2021 Mar 31. PMID: 33792038.	Excluded	Title/Abstract	
214	Szczęsny G, Ratajczak K, Fijewski G, Małdyk P. Intentional Overscrewing of Humeral Head in Comminuted Fracture of Proximal Humerus and its Impact on Shoulder Function . A Case Study. Ortop Traumatol Rehabil. 2019 Aug 31;21(4):297-305. doi: 10.5604/01.3001.0013.5075. PMID: 32015204.	Excluded	Title/Abstract	
215	Tabor OB. Treatment of single compartment osteoarthritis of the knee using modular arthroplasty: a review of the literature and presentation of 29 cases. Orthopedics. 1984 Jun;7(6):979-83. doi: 10.3928/0147-7447-19840601-11. PMID: 24822729.	Excluded	Title/Abstract	
216	Taneja P, Kashyap P, Marya CM, Nagpal R, Kataria S, Mahapatra S, Marya A. Knowledge, Attitude, Practices, and Preparedness of Dental Professionals in Prescribing Nicotine Replacement Therapy. Biomed Res Int. 2022 Feb 17;2022:5782228. doi: 10.1155/2022/5782228. PMID: 35237688; PMCID: PMC8885202.	Excluded	Title/Abstract	

217	Tang L, Chen M, Li G, Luo Z, Ji X, Zhang X, Wu K, Zhu C, Shang X. [Effectiveness of proximal femur reconstruction combined with total hip arthroplasty for Crowe type IV developmental dysplasia of hip]. Zhongguo Xiu Fu Chong Jian Wai Ke Za Zhi. 2020 Jun 15;34(6):683-688. Chinese. doi: 10.7507/1002-1892.201911073. PMID: 32538556; PMCID: PMC8171527.	Excluded	Title/Abstract	
218	Thiruvenkatachari B, Sivakumar P, Ananth S, Sabbagh Y, Lewis BRK, Chadwick SM, Kaliyaperumal GS, Batra P. The impact of COVID-19 pandemic on orthodontic services and trainees' mental health in India. Front Med (Lausanne). 2023 Sep 1;10:1220505. doi: 10.3389/fmed.2023.1220505. PMID: 37724176; PMCID: PMC10505436.	Excluded	Title/Abstract	
219	Tiro A, Nakas E, Arslanagic A, Markovic N, Dzemidzic V. Perception of Dentofacial Aesthetics in School Children and Their Parents. Eur J Dent. 2021 Feb;15(1):13-19. doi: 10.1055/s-0040-1714040. Epub 2020 Jul 29. PMID: 32726855; PMCID: PMC7902104.	Excluded	Title/Abstract	
220	Tom K, Mancl L, Woloshyn H, Khosravi R, Bollen AM. Association between crowding estimation and extraction recommendations in orthodontics. Am J Orthod Dentofacial Orthop. 2024 Jan;165(1):64-72.e12. doi: 10.1016/j.ajodo.2023.07.012. Epub 2023 Sep 15. PMID: 37715755.	Excluded	Title/Abstract	
221	Turkalj M, Ghosh M, Čokić SM, Hoet PHM, Vanoirbeek J, Van Meerbeek B, Van Landuyt KL. Cytotoxicity assessment of eluates from vacuum-forming thermoplastics. Clin Oral Investig. 2024 Oct 4;28(10):570. doi: 10.1007/s00784-024-05952-4. PMID: 39365355.	Excluded	Title/Abstract	
222	Vasudavan S, Sullivan SR, Sonis AL. Comparison of intraoral 3D scanning and conventional impressions for fabrication of orthodontic retainers. J Clin Orthod. 2010 Aug;44(8):495-7. PMID: 21105587.	Excluded	Title/Abstract	

223	Verma SK, Kumar BD, Chandra S, Singh N, Kumari P, Verma A. Knowledge, Attitude, and Psychological Effect on Undergraduate/ Postgraduate Students in Lockdown COVID-19 Situation. J Pharm Bioallied Sci. 2021 Jun;13(Suppl 1):S696-S698. doi: 10.4103/jpbs.JPBS_829_20. Epub 2021 Jun 5. PMID: 34447184; PMCID: PMC8375943.	Excluded	Title/Abstract	
224	Wade N, Paul N, Nagar N, Rolland S, Germain S. Information-seeking behaviour in patients exploring orthognathic surgery: A qualitative study. J Orthod. 2025 Mar;52(1):63-71. doi: 10.1177/14653125241249494. Epub 2024 May 8. PMID: 38717064; PMCID: PMC11951351.	Excluded	Title/Abstract	
225	Wafaie K, Rizk MZ, Basyouni ME, Daniel B, Mohammed H. Tele-orthodontics and sensor-based technologies: a systematic review of interventions that monitor and improve compliance of orthodontic patients. Eur J Orthod. 2023 Jul 31;45(4):450-461. doi: 10.1093/ejo/cjad004. PMID: 37132630.	Excluded	Title/Abstract	
226	Wall A, Hillyard N, Ryan FS, Cunningham SJ. Adult orthodontic patients: What is important to them? J Orthod. 2024 Dec;51(4):366-374. doi: 10.1177/14653125241256975. Epub 2024 Jun 10. PMID: 38859622.	Excluded	Title/Abstract	
227	Wang X, Chen H, Ge S, Orhan K, van der Stelt P, Shi XQ. Comparison of side effects of different mandibular advancement devices for patients with obstructive sleep apnea: a systematic review and meta-analysis. J Clin Sleep Med. 2025 Aug 1;21(8):1477-1486. doi: 10.5664/jcsm.11712. PMID: 40160004; PMCID: PMC12320689.	Excluded	Title/Abstract	
228	Wang Y, Hofmann M, Ruf S, Zhang J, Huang Q. Intentional replantation and dental autotransplantation of mandibular posterior teeth: Two case reports. Medicine (Baltimore). 2023 Nov 17;102(46):e35822. doi: 10.1097/MD.0000000000035822. PMID: 37986277; PMCID: PMC10659724.	Excluded	Title/Abstract	

229	Wang Y, Xu P, Wang Y, Liu Y, Xu S, Zhao Z, Li H, Chen X. [Biomechanical study of lumbar vertebra during gait cycle in adolescent idiopathic scoliosis]. Sheng Wu Yi Xue Gong Cheng Xue Za Zhi. 2025 Jun 25;42(3):601-609. Chinese. doi: 10.7507/1001-5515.202410010. PMID: 40566784; PMCID: PMC12236210.	Excluded	Title/Abstract	
230	Watts GD, Christou P, Antonarakis GS. Experiences Of Individuals Concerning Combined Orthodontic and Orthognathic Surgical Treatment: A Qualitative Twitter Analysis. Med Princ Pract. 2018;27(3):227-235. doi: 10.1159/000487904. Epub 2018 Apr 11. PMID: 29642059; PMCID: PMC6062725.	Excluded	Title/Abstract	
231	Widmer CG, Morris-Wiman J. Mouse preferential incising force orientation changes during jaw closing muscle hyperalgesia and is sex dependent. Physiol Behav. 2016 Dec 1;167:132-144. doi: 10.1016/j.physbeh.2016.09.005. Epub 2016 Sep 9. PMID: 27619173; PMCID: PMC5159302.	Excluded	Title/Abstract	
232	Willershausen I, Necker F, Kloeckner R, Seidel CL, Paulsen F, Gölz L, Scholz M. Cinematic rendering to improve visualization of supplementary and ectopic teeth using CT datasets. Dentomaxillofac Radiol. 2023 Apr;52(4):20230058. doi: 10.1259/dmfr.20230058. Epub 2023 Apr 4. PMID: 37015249; PMCID: PMC10170174.	Excluded	Title/Abstract	
233	Willinger L, Imhoff AB, Schmitt A, Forkel P. Refixation tibialer knöcherner Avulsionsverletzungen des hinteren Kreuzbandes in Fadenankertechnik [Fixation of bony avulsions of the posterior cruciate ligament by a suture-bridge™ technique]. Oper Orthop Traumatol. 2019 Feb;31(1):3-11. German. doi: 10.1007/s00064-018-0582-4. Epub 2018 Dec 18. PMID: 30564842.	Excluded	Title/Abstract	

234	Wilson KE, Girdler NM, Welbury RR. Randomized, controlled, cross-over clinical trial comparing intravenous midazolam sedation with nitrous oxide sedation in children undergoing dental extractions. Br J Anaesth. 2003 Dec;91(6):850-6. doi: 10.1093/bja/aeg278. PMID: 14633757.	Excluded	Title/Abstract	
235	Wörtche R, Hassfeld S, Lux CJ, Müssig E, Hensley FW, Krempien R, Hofele C. Clinical application of cone beam digital volume tomography in children with cleft lip and palate. Dentomaxillofac Radiol. 2006 Mar;35(2):88-94. doi: 10.1259/dmfr/27536604. PMID: 16549435.	Excluded	Title/Abstract	
236	Wu J, Pan Q. [Semi-joint prosthesis replacement in treatment of malignant tumors around children's knees]. Zhongguo Xiu Fu Chong Jian Wai Ke Za Zhi. 2006 Oct;20(10):978-80. Chinese. PMID: 17140067.	Excluded	Title/Abstract	
237	Xu B, Yang K. Changes in alveolar bone structure during orthodontic tooth movement in adolescent and adult rats: A microcomputed tomography study. Orthod Craniofac Res. 2023 Nov;26(4):568-575. doi: 10.1111/ocr.12646. Epub 2023 Mar 10. PMID: 36866954.	Excluded	Title/Abstract	
238	Yagci G, Ayhan C, Yakut Y. Effectiveness of basic body awareness therapy in adolescents with idiopathic scoliosis: A randomized controlled study1. J Back Musculoskelet Rehabil. 2018;31(4):693-701. doi: 10.3233/BMR-170868. PMID: 29630516.	Excluded	Title/Abstract	
239	Yamada K, Hassan GS, Yamaki M, Hanada K. Use of X-rays in the computer-assisted patient information system. J Clin Orthod. 1996 Apr;30(4):206-7. PMID: 8975018.	Excluded	Title/Abstract	
240	Yuo TC, Cheng JH, De-Shing Chen D, Samson RS, Varghese E, Bayarsaikhan O. Patient considerations for orthodontists: A comparative study of university students in Malaysia and Taiwan. J Dent Sci. 2024 Oct;19(4):1991-1998. doi: 10.1016/j.jds.2024.07.003. Epub 2024 Jul 17. PMID: 39347045; PMCID: PMC11437273.	Excluded	Title/Abstract	

241	Yu J, Wang HM, Zhu XM, Fan ZL. [Clinical study on the treatment of Class II and ClassImalocclusion in adolescents with mandibular recession using invisible orthodontic appliances without brackets]. Shanghai Kou Qiang Yi Xue. 2024 Dec;33(6):661-666. Chinese. PMID: 40007301.	Excluded	Title/Abstract	
242	Zaheer U, Shahbaz M, Zaheer N, Israr J, Riaz A, Yaqub A, Alam MK. Knowledge, attitude, and behaviour of dentists working during the COVID-19 pandemic: A cross-sectional survey. Work. 2022;72(4):1153-1161. doi: 10.3233/WOR-211018. PMID: 35723151.	Excluded	Title/Abstract	
243	Zhang R, Chen H, Ma Y, Jin Z. Dental Artificial Intelligence Systems: A Review of Various Data Types. Discov Med. 2024 Mar;36(182):482-493. doi: 10.24976/Discov.Med.202436182.45. PMID: 38531789.	Excluded	Title/Abstract	
244	Zheng JL, Li Y, Hogue G, Johnson M, Anari JB, Regan MD, Baldwin KD. What imaging does my AIS patient need? A multi-group survey of provider preferences. Spine Deform. 2025 Mar;13(2):351-359. doi: 10.1007/s43390-024-00995-9. Epub 2024 Nov 4. PMID: 39495401; PMCID: PMC11893670.	Excluded	Title/Abstract	
245	Zhu Z, Ye Z, Wang Q, Li R, Li H, Guo W, Li Z, Xia L, Fang B. Evolutionary Trend of Dental Health Care Information on Chinese Social Media Platforms During 2018-2022: Retrospective Observational Study. JMIR Infodemiology. 2025 Apr 10;5:e55065. doi: 10.2196/55065. PMID: 40209216; PMCID: PMC12022532.	Excluded	Title/Abstract	
**2. Cochrane Library**				
**Search date**	25/8/2025			

**Results**	113			
**Search Query**	(Orthodontics OR "orthodontic treatment" OR braces OR "clear aligners" OR Invisalign) AND ("Social Media" OR "socialnetworks" OR Facebook OR Instagram OR TikTok OR YouTube OR Twitter OR X OR Snapchat) AND (willingnes OR motivation OR intent* OR perceptionOR attitude OR "decision making" OR preference* OR "patient acceptance")"			
**Nr.**	**Authors-Title**	**Eligibility**	**Reason for exclusion**	
1	Gao M, Yan X, Zhao R, Shan Y, Chen Y, Jian F, Long H, Lai W. Comparison of pain perception, anxiety, and impacts on oral health-related quality of life between patients receiving clear aligners and fixed appliances during the initial stage of orthodontic treatment. European Journal of Orthodontics 2021;43(3):353–359. DOI: 10.1093/ejo/cjaa056. Accessed 2 September 2025.	Excluded	Title/Abstract	
2	NCT06968325. AI-Designed Clear Aligners: a Randomized Clinical Trial. ClinicalTrials.gov 2025. Available from: https://clinicaltrials.gov/ct2/show/NCT06968325. Accessed 2 September 2025.	Excluded	Title/Abstract	
3	NCT06886984. Effects of Social Media on Oral Hygiene With Orthodontic Patients. ClinicalTrials.gov 2025. Available from: https://clinicaltrials.gov/ct2/show/NCT06886984. Accessed 2 September 2025	Excluded	Title/Abstract	
4	Dovgan JS, Walton RE, Bishara SE. Electrothermal debracketing: patient acceptance and effects on the dental pulp. American Journal of Orthodontics and Dentofacial Orthopedics 1995;108(3):249–255. PMID: 7572866. Accessed 2 September 2025.	Excluded	Title/Abstract	

5	Sjögren A, Arnrup K, Jensen C, Knutsson I, Huggare J. Pain and fear in connection to orthodontic extractions of deciduous canines. International Journal of Paediatric Dentistry 2010;20(3):193–200. DOI: 10.1111/j.1365-263X.2010.01034.x. Accessed 2 September 2025.	Excluded	Title/Abstract	
6	NCT02644811. Miniscrews as Anchorage Device for Orthodontic Treatment. ClinicalTrials.gov 2015. Available from: https://clinicaltrials.gov/show/NCT02644811. Accessed 2 September 2025.	Excluded	Title/Abstract	
7	Baumrind S, Korn EL, Boyd RL, Maxwell R. The decision to extract: part II. Analysis of clinicians' stated reasons for extraction. American Journal of Orthodontics and Dentofacial Orthopedics 1996;109(4):393–402. PMID: 8629423. Accessed 2 September 2025.	Excluded	Title/Abstract	
8	CTRI/2020/03/023970. Computer Model Designing for Taking Decision on Removal of Teeth for Orthodontic Tooth Correction. WHO ICTRP 2020. Available from: https://trialsearch.who.int/Trial2.aspx?TrialID=CTRI/2020/03/023970. Accessed 2 September 2025.	Excluded	Title/Abstract	
9	Karkun R, Batra P, Singh AK. Influence of social media and corrected smile photographs in patients with malocclusion. American Journal of Orthodontics and Dentofacial Orthopedics 2023;164(5):712–727. DOI: 10.1016/j.ajodo.2022.09.015. Accessed 2 September 2025.	DUPLICATE PUB 115		
10	Xu X, Zhang L, Jiang Y, Huang Y, Huang S, Yang S. Clinical research of music in relieving orthodontic pain. Hua Xi Kou Qiang Yi Xue Za Zhi [West China Journal of Stomatology] 2013;31(4):365–368. PMID: 24386755. Accessed 2 September 2025.	Excluded	Title/Abstract	
11	Montebugnoli F, Incerti Parenti S, D'Antò V, Alessandri-Bonetti G, Michelotti A. Effect of verbal and written information on pain perception in patients undergoing fixed orthodontic treatment: a randomized	DUPLICATE PUB 153	Title/Abstract	

	controlled trial. European Journal of Orthodontics 2020;42(5):494–499. DOI: 10.1093/ejo/cjz058. Accessed 2 September 2025.			
12	NCT05723094. Effect of Low-Level Laser Therapy on Stability During Retention Phase. ClinicalTrials.gov 2023. Available from: https://clinicaltrials.gov/show/NCT05723094. Accessed 2 September 2025.	Excluded	Title/Abstract	
13	Krug AY, Green C. Changes in patient evaluation of completed orthodontic esthetics after dental bleaching. Journal of Esthetic and Restorative Dentistry 2008;20(5):313–319. DOI: 10.1111/j.1708-8240.2008.00201.x. Accessed 2 September 2025	Excluded	Title/Abstract	
14	Gross RT, Ghaltakhchyan N, Nanney EM, Jackson TH, Wiesen CA, Mihas P, Persky AM, Frazier-Bowers SA, Jacox LA. Evaluating video-based lectures on YouTube for dental education. Orthodontics & Craniofacial Research 2023;26 Suppl 1:210–220. DOI: 10.1111/ocr.12681. Accessed 2 September 2025.	DUPLICATE PUB 85	Title/Abstract	
15	NCT03755622. Comparing Clinical Application of Innovatedly Made Transpalatal Arch (TPA) From 3D Reconstructed Model and Conventionally Made TPA. ClinicalTrials.gov 2018. Available from: https://clinicaltrials.gov/show/NCT03755622. Accessed 2 September 2025.	Excluded	Title/Abstract	
16	Robert-Lachaine X, Dessery Y, Belzile ÉL, Turmel S, Corbeil P. Three-month efficacy of three knee braces in the treatment of medial knee osteoarthritis in a randomized crossover trial. Journal of Orthopaedic Research 2020;38(10):2262–2271. DOI: 10.1002/jor.24683. Accessed 2 September 2025	Excluded	Title/Abstract	

17	RBR-9zytwf. Effects of orthodontic treatment with clear aligner and conventional fixed appliance. WHO ICTRP 2019. Available from: https://trialsearch.who.int/Trial2.aspx?TrialID=RBR-9zytwf. Accessed 2 September 2025.	Excluded	Title/Abstract	
18	Noori RM, Yassir YA. Effectiveness of tubular coaxial nickel-titanium and copper nickel-titanium orthodontic aligning archwires: a randomized clinical trial. International Orthodontics 2023;21(4):100812. DOI: 10.1016/j.ortho.2023.100812. Accessed 2 September 2025	Excluded	Title/Abstract	
19	Pinheiro FHSL, Frota CM, Garib DG, Sathler R, Ozawa TO, Lauris RCMC, Kato RM, Kurimori ÉT. A Cleft-Customized Occlusal Rating System to Assess Orthodontic Occlusal Improvement in Patients With Unilateral Cleft Lip and Palate. Cleft Palate-Craniofacial Journal 2022;59(1):54–65. DOI: 10.1177/10556656211005469. Accessed 2 September 2025.	Excluded	Title/Abstract	
20	NL-OMON41788. Effect of a soft brace on pain, knee stability and activity limitations in patients with knee osteoarthritis. WHO ICTRP 2015. Available from: https://trialsearch.who.int/Trial2.aspx?TrialID=NL-OMON41788. Accessed 2 September 2025.	Excluded	Title/Abstract	
21	Godfrey J, Little KJ, Cornwall R, Carr P, Samora J. Increasing brace treatment for distal radius buckle fractures: using quality improvement methodology to implement evidence-based medicine. Journal of Hand Surgery 2017;42(9):S25. DOI: 10.1016/j.jhsa.2017.06.050. Accessed 2 September 2025.	Excluded	Title/Abstract	
22	Ey-Chmielewska H. An attempt to use ultrasonic technique for confirming the diagnosis, planning and observation of long-term treatment results of painful temporo-mandibular joint dysfunction.	DUPLICATE PUB 68	Title/Abstract	

	Annales Academiae Medicae Stetinensis 1998;44:223–236. PMID: 10375927. Accessed 2 September 2025.			
23	Vasudavan S, Sullivan SR, Sonis AL. Comparison of intraoral 3D scanning and conventional impressions for fabrication of orthodontic retainers. Journal of Clinical Orthodontics 2010;44(8):495–497. PMID: 20829124. Accessed 2 September 2025.	DUPLICATE PUB 222	Title/Abstract	
24	Bullock L, Holden MA, Jinks C, Asamane EA, Herron D, Borrelli B, Callaghan MJ, Birrell F, Halliday N, Marshall M, Sowden G, Ingram C, McBeth J, Dziedzic K, Foster NE, Jowett S, Lawton S, Mallen CD, Peat G. Physiotherapists' Experiences and Perceived Acceptability of Delivering a Knee Bracing Intervention for People With Symptomatic Knee Osteoarthritis in a Randomised Trial (PROP OA): a Qualitative Study. Musculoskeletal Care 2024;22(4):e70021. DOI: 10.1002/msc.70021. Accessed 2 September 2025.	DUPLICATE PUB 43		
25	Phillips C, Kim SH, Tucker M, Turvey TA. Sensory retraining: burden in daily life related to altered sensation after orthognathic surgery, a randomized clinical trial. Orthodontics & Craniofacial Research 2010;13(3):169–178. DOI: 10.1111/j.1601-6343.2010.01494.x. Accessed 2 September 2025.	Excluded	Title/Abstract	
26	NCT04191070. Distalization Using Infrazygomatic Crest Miniscrews and Zygomatic Miniplates: a Randomized Clinical Trial. ClinicalTrials.gov 2019. Available from: https://clinicaltrials.gov/show/NCT04191070. Accessed 2 September 2025.	Excluded	Title/Abstract	
27	RBR-2fvknfd. Stability of printed versus conventional retainers: randomized clinical trial. WHO ICTRP 2023. Available from:	Excluded	Title/Abstract	

	https://trialsearch.who.int/Trial2.aspx?TrialID=RBR-2fvknfd. Accessed 2 September 2025.			
28	Sapata DM, Oliveira ES, Pascotto RC, Poleti TMFF, Arai MSI, Ramos AL. Periodontal indexes of two types of 3 x 3 retainers: 0.032-in SS V-loop versus 0.0215-in SS coaxial - a randomized crossover trial. Dental Press Journal of Orthodontics 2024;28(6):e2323175. DOI: 10.1590/2177-6709.28.6.e2323175.onl. Accessed 2 September 2025	DUPLICATE PUB 192		
29	Chen X, Chen W, Hu W, Huang K, Huang J, Zhou Y. Nurse-led intensive interventions improve adherence to continuous positive airway pressure therapy and quality of life in obstructive sleep apnea patients. Patient Preference and Adherence 2015;9:1707–1713. DOI: 10.2147/PPA.S90420. Accessed 2 September 2025.	Excluded	Title/Abstract	
30	NCT03601130. Assessment of HydroxyColl Bone Graft Substitute in High Tibial Osteotomy Wedge Grafting. ClinicalTrials.gov 2018. Available from: https://clinicaltrials.gov/show/NCT03601130. Accessed 2 September 2025.	Excluded	Title/Abstract	
31	Wilson KE, Welbury RR, Girdler NM. A randomised, controlled, crossover trial of oral midazolam and nitrous oxide for paediatric dental sedation. Anaesthesia 2002;57(9):860–867. DOI: 10.1046/j.1365-2044.2002.02716.x. Accessed 2 September 2025.	Excluded	Title/Abstract	
32	Diernberger S, Bernhardt O, Schwahn C, Kordass B. Self-reported chewing side preference and its associations with occlusal, temporomandibular and prosthodontic factors: results from the population-based Study of Health in Pomerania (SHIP-0). Journal of Oral Rehabilitation 2008;35(8):613–620. DOI: 10.1111/j.1365-2842.2007.01834.x. Accessed 2 September 2025.	Excluded	Title/Abstract	

33	Yip BHK, Li X, Leung CHY, Gao T, Chung VCH, Yu FWP, Lam TP, Cheng JCY, Wong SYS. Trial Protocol: the use of mindfulness-based intervention for improving bracing compliance for adolescent idiopathic scoliosis patients: protocol for a randomised, controlled trial. Journal of Physiotherapy 2018;64(3):193. DOI: 10.1016/j.jphys.2018.05.012. Accessed 2 September 2025.	Excluded	Title/Abstract	
34	El Riheem YMA, El-Barbary HM, Hegazy MM, Arafa AS. Comparative Study between Arthroereisis Versus Calcaneal Lengthening in Planovulgus Foot in Cerebral Palsy Children. Journal of Pharmaceutical Negative Results 2023;14:51–59. DOI: 10.47750/pnr.2023.14.01.07. Accessed 2 September 2025.	Excluded	Title/Abstract	
35	CTRI/2021/06/034174. Evaluation of pain perception and enamel loss by two different techniques: a randomized clinical trial. WHO ICTRP 2021. Available from: https://trialsearch.who.int/Trial2.aspx?TrialID=CTRI/2021/06/034174. Accessed 2 September 2025.	Excluded	Title/Abstract	
36	Zotti F, Zotti R, Albanese M, Nocini PF, Paganelli C. Implementing post-orthodontic compliance among adolescents wearing removable retainers through whatsapp: a pilot study. Patient Preference and Adherence 2019;13:609–615. DOI: 10.2147/PPA.S198675. Accessed 2 September 2025.	Excluded	Title/Abstract	
37	Zhang XG, Yang F, Wu TX, Shi ZD, Yi XZ. Evidence of Cochrane systematic reviews on the treatment of temporomandibular disorders. Chinese Journal of Evidence-Based Medicine 2008;8(12):1130–1132. Accessed 2 September 2025.	Excluded	Title/Abstract	
38	ISRCTN11717011. Comparison of the fixed versus the removable functional braces in the treatment of children with sticking out top front teeth. WHO ICTRP 2016. Available from:	Excluded	Title/Abstract	

	https://trialsearch.who.int/Trial2.aspx?TrialID=ISRCTN11717011. Accessed 2 September 2025.			
39	AlQabandi AK, Sadowsky C, BeGole EA. A comparison of the effects of rectangular and round arch wires in leveling the curve of Spee. American Journal of Orthodontics and Dentofacial Orthopedics 1999;116(5):522–529. DOI: 10.1016/S0889-5406(99)70278-3. Accessed 2 September 2025.	DUPLICATE PUB 23		
40	ISRCTN12197405. An investigation of treating patients with conventional and skeletal anchored protraction headgear. WHO ICTRP 2020. Available from: https://trialsearch.who.int/Trial2.aspx?TrialID=ISRCTN12197405. Accessed 2 September 2025.	Excluded	Title/Abstract	
41	Feistritzer-Grobl P, Nischelwitzer A, Saraph V. Game based physiotherapy for evidence based practice in children with juvenile idiopathic scoliosis. Scoliosis 2013;8:O21. DOI: 10.1186/1748-7161-8-S2-O21. Accessed 2 September 2025.	Excluded	Title/Abstract	
42	NCT04382638. Effectiveness of Three Dimensional Correction During in Bracing in Adolescent Idiopathic Scoliosis. ClinicalTrials.gov 2020. Available from: https://clinicaltrials.gov/show/NCT04382638. Accessed 2 September 2025.	Excluded	Title/Abstract	
43	RBR-7dydc4h. Effects of early treatment of anterior open bite with orthodontic aligners and removable palatal crib. WHO ICTRP 2024. Available from: https://trialsearch.who.int/Trial2.aspx?TrialID=RBR-7dydc4h. Accessed 2 September 2025.	Excluded	Title/Abstract	
44	ISRCTN68289972. A comparison of mandibular archwidth changes using two different bracket systems. WHO ICTRP 2012. Available from:	Excluded	Title/Abstract	

	https://trialsearch.who.int/Trial2.aspx?TrialID=ISRCTN68289972. Accessed 2 September 2025.			
45	Bains M, Reynolds PA, McDonald F, Sherriff M. Effectiveness and acceptability of face-to-face, blended and e-learning: a randomised trial of orthodontic undergraduates. European Journal of Dental Education 2011;15(2):110–117. DOI: 10.1111/j.1600-0579.2010.00653.x. Accessed 2 September 2025.	Excluded	Title/Abstract	
46	Chen D, Liu X, Liu Y, Wang X, Zheng J, Wu L. Virtual reality used in undergraduate orthodontic education. European Journal of Dental Education 2025;29(3):576–584. DOI: 10.1111/eje.12842. Accessed 2 September 2025	Excluded	Title/Abstract	
47	Paterson KL, Bennell KL, Campbell PK, Metcalf BR, Kasza J, Wrigley TW, Hinman RS. Footwear for self-managing knee osteoarthritis symptoms: the footstep randomized controlled trial. Osteoarthritis and Cartilage 2021;29(Suppl 1):S57–S58. DOI: 10.1016/j.joca.2021.02.095. Accessed 2 September 2025.	Excluded	Title/Abstract	
48	Al-Attar A, Nissan L, Almuzian M, Abid M. Effect of mini-implant facilitated micro-osteoperforations on the alignment of mandibular anterior crowding: a randomised controlled clinical trial. Journal of Orthodontics 2022;49(4):379–387. DOI: 10.1177/14653125221092043. Accessed 2 September 2025.	Excluded	Title/Abstract	
49	NCT07063186. Comparing Ligation Methods During Canine Retraction Stage Using Conventional Brackets. ClinicalTrials.gov 2025. Available from: https://clinicaltrials.gov/ct2/show/NCT07063186. Accessed 2 September 2025.	Excluded	Title/Abstract	
50	NCT06936033. Kinesiology Taping and Ankle Stability in Acute Injuries During Stair Descent. ClinicalTrials.gov 2025. Available from:	Excluded	Title/Abstract	

	https://clinicaltrials.gov/ct2/show/NCT06936033. Accessed 2 September 2025.			
51	NCT06807645. Evaluating the Impact of Prefabricated Acrylic Splints on Premaxillary Stability in Alveolar Cleft Bone Graft Repairs: a Randomized Controlled Trial.Available from: https://clinicaltrials.gov/ct2/show/NCT06807645, 2025ǀ added to CENTRAL: 28 February 2025 ǀ 2025 Issue 2	Excluded	Title/Abstract	
52	El-Angbawi A, McIntyre G, Fleming PS, Bearn D. Non-surgical adjunctive interventions for accelerating tooth movement in patients undergoing orthodontic treatment. Cochrane Database of Systematic Reviews 2023, Issue 6. Art. No.: CD010887. DOI: 10.1002/14651858.CD010887.pub3. Accessed 02 September 2025.	Excluded	Title/Abstract	
53	Fleming PS, Fedorowicz Z, Johal A, El-Angbawi A, Pandis N. Surgical adjunctive procedures for accelerating orthodontic treatment. Cochrane Database of Systematic Reviews 2015, Issue 6. Art. No.: CD010572. DOI: 10.1002/14651858.CD010572.pub2. Accessed 02 September 2025.	Excluded	Title/Abstract	
54	Hu H, Li C, Li F, Chen J, Sun J, Zou S, Sandham A, Xu Q, Riley P, Ye Q. Enamel etching for bonding fixed orthodontic braces. Cochrane Database of Systematic Reviews 2013, Issue 11. Art. No.: CD005516. DOI: 10.1002/14651858.CD005516.pub2. Accessed 02 September 2025.	Excluded	Title/Abstract	
55	Turner S, Harrison JE, Sharif FNJ, Owens D, Millett DT. Orthodontic treatment for crowded teeth in children. Cochrane Database of Systematic Reviews 2021, Issue 12. Art. No.: CD003453. DOI: 10.1002/14651858.CD003453.pub2. Accessed 02 September 2025.	Excluded	Title/Abstract	
56	Todhunter-Brown A, Sellers CE, Baer GD, Choo PL, Cowie J, Cheyne JD, Langhorne P, Brown J, Morris J, Campbell P. Physical rehabilitation approaches for the recovery of function and mobility following stroke.	Excluded	Title/Abstract	

	Cochrane Database of Systematic Reviews 2025, Issue 2. Art. No.: CD001920. DOI: 10.1002/14651858.CD001920.pub4. Accessed 02 September 2025.			
57	Liu C, Wei Z, Jian F, McIntyre G, Millett DT, Lai W, Wang Y. Initial arch wires used in orthodontic treatment with fixed appliances. Cochrane Database of Systematic Reviews 2024, Issue 2. Art. No.: CD007859. DOI: 10.1002/14651858.CD007859.pub5. Accessed 02 September 2025.	Excluded	Title/Abstract	
58	Handoll HHG, Elliott J, Iheozor-Ejiofor Z, Hunter J, Karantana A. Interventions for treating wrist fractures in children. Cochrane Database of Systematic Reviews 2018, Issue 12. Art. No.: CD012470. DOI: 10.1002/14651858.CD012470.pub2. Accessed 02 September 2025.	Excluded	Title/Abstract	
59	Kloukos D, Fudalej P, Sequeira-Byron P, Katsaros C. Maxillary distraction osteogenesis versus orthognathic surgery for cleft lip and palate patients. Cochrane Database of Systematic Reviews 2018, Issue 8. Art. No.: CD010403. DOI: 10.1002/14651858.CD010403.pub3. Accessed 02 September 2025.	Excluded	Title/Abstract	
60	Rizzo RRN, Cashin AG, Wand BM, Ferraro MC, Sharma S, Lee H, O'Hagan E, Maher CG, Furlan AD, van Tulder MW, McAuley JH. Non-pharmacological and non-surgical treatments for low back pain in adults: an overview of Cochrane reviews. Cochrane Database of Systematic Reviews 2025, Issue 3. Art. No.: CD014691. DOI: 10.1002/14651858.CD014691.pub2. Accessed 02 September 2025.	Excluded	Title/Abstract	
61	Kinnersley P, Phillips K, Savage K, Kelly MJ, Farrell E, Morgan B, Whistance R, Lewis V, Mann MK, Stephens BL, Blazeby J, Elwyn G, Edwards AGK. Interventions to promote informed consent for patients undergoing surgical and other invasive healthcare procedures. Cochrane	Excluded	Title/Abstract	

	Database of Systematic Reviews 2013, Issue 7. Art. No.: CD009445. DOI: 10.1002/14651858.CD009445.pub2. Accessed 02 September 2025.			
62	Chen DML, Han S, Summers A, Wang J, Rice M, Mallios J, Leslie L, Harb EN, Qureshi R. Interventions for improving adherence to amblyopia treatments in children. Cochrane Database of Systematic Reviews 2025, Issue 7. Art. No.: CD015820. DOI: 10.1002/14651858.CD015820.pub2. Accessed 02 September 2025.	Excluded	Title/Abstract	
63	Winfield NR, Barker NJ, Turner ER, Quin GL. Non-pharmaceutical management of respiratory morbidity in children with severe global developmental delay. Cochrane Database of Systematic Reviews 2014, Issue 10. Art. No.: CD010382. DOI: 10.1002/14651858.CD010382.pub2. Accessed 02 September 2025.	Excluded	Title/Abstract	
65	Negrini S, Minozzi S, Bettany-Saltikov J, Chockalingam N, Grivas TB, Kotwicki T, Maruyama T, Romano M, Zaina F. Braces for idiopathic scoliosis in adolescents. Cochrane Database of Systematic Reviews 2015, Issue 6. Art. No.: CD006850. DOI: 10.1002/14651858.CD006850.pub3. Accessed 02 September 2025.	Excluded	Title/Abstract	
66	Dias CGP, Godoy-Santos AL, Ferrari J, Ferretti M, Lenza M. Surgical interventions for treating hallux valgus and bunions. Cochrane Database of Systematic Reviews 2024, Issue 7. Art. No.: CD013726. DOI: 10.1002/14651858.CD013726.pub2. Accessed 02 September 2025.	Excluded	Title/Abstract	
67	Duivenvoorden T, Brouwer RW, van Raaij TM, Verhagen AP, Verhaar JAN, Bierma-Zeinstra SMA. Braces and orthoses for treating osteoarthritis of the knee. Cochrane Database of Systematic Reviews	Excluded	Title/Abstract	

	2015, Issue 3. Art. No.: CD004020. DOI: 10.1002/14651858.CD004020.pub3. Accessed 02 September 2025.			
68	Dyer TA, Brocklehurst P, Glenny AM, Davies L, Tickle M, Issac A, Robinson PG. Dental auxiliaries for dental care traditionally provided by dentists. Cochrane Database of Systematic Reviews 2014, Issue 8. Art. No.: CD010076. DOI: 10.1002/14651858.CD010076.pub2. Accessed 02 September 2025.	Excluded	Title/Abstract	
69	Bettany-Saltikov J, Weiss HR, Chockalingam N, Taranu R, Srinivas S, Hogg J, Whittaker V, Kalyan RV, Arnell T. Surgical versus non-surgical interventions in people with adolescent idiopathic scoliosis. Cochrane Database of Systematic Reviews 2015, Issue 4. Art. No.: CD010663. DOI: 10.1002/14651858.CD010663.pub2. Accessed 02 September 2025.	Excluded	Title/Abstract	
70	Romano M, Minozzi S, Bettany-Saltikov J, Zaina F, Chockalingam N, Kotwicki T, Maier-Hennes A, Arienti C, Negrini S. Therapeutic exercises for idiopathic scoliosis in adolescents. Cochrane Database of Systematic Reviews 2024, Issue 2. Art. No.: CD007837. DOI: 10.1002/14651858.CD007837.pub3. Accessed 02 September 2025.	Excluded	Title/Abstract	
71	Singh BP, Singh N, Jayaraman S, Kirubakaran R, Joseph S, Muthu MS, Jivnani H, Hua F. Occlusal interventions for managing temporomandibular disorders. Cochrane Database of Systematic Reviews 2024, Issue 9. Art. No.: CD012850. DOI: 10.1002/14651858.CD012850.pub2. Accessed 02 September 2025.	Excluded	Title/Abstract	
72	Zaina F, Tomkins-Lane C, Carragee E, Negrini S. Surgical versus non-surgical treatment for lumbar spinal stenosis. Cochrane Database of Systematic Reviews 2016, Issue 1. Art. No.: CD010264. DOI: 10.1002/14651858.CD010264.pub2. Accessed 02 September 2025.	Excluded	Title/Abstract	

73	Yeung DE, Jia X, Miller CA, Barker SL. Interventions for treating ankle fractures in children. Cochrane Database of Systematic Reviews 2016, Issue 4. Art. No.: CD010836. DOI: 10.1002/14651858.CD010836.pub2. Accessed 02 September 2025.	Excluded	Title/Abstract	
74	Handoll HHG, Pearce P. Interventions for treating isolated diaphyseal fractures of the ulna in adults. Cochrane Database of Systematic Reviews 2012, Issue 6. Art. No.: CD000523. DOI: 10.1002/14651858.CD000523.pub4. Accessed 02 September 2025.	Excluded	Title/Abstract	
75	Witteveen AGH, Hofstad CJ, Kerkhoffs GMMJ. Hyaluronic acid and other conservative treatment options for osteoarthritis of the ankle. Cochrane Database of Systematic Reviews 2015, Issue 10. Art. No.: CD010643. DOI: 10.1002/14651858.CD010643.pub2. Accessed 02 September 2025.	Excluded	Title/Abstract	
76	Handoll HHG, Madhok R. Conservative interventions for treating distal radial fractures in adults. Cochrane Database of Systematic Reviews 2003, Issue 2. Art. No.: CD000314. DOI: 10.1002/14651858.CD000314. Accessed 02 September 2025.	Excluded	Title/Abstract	
77	Matthews E, Brassington R, Kuntzer T, Jichi F, Manzur AY. Corticosteroids for the treatment of Duchenne muscular dystrophy. Cochrane Database of Systematic Reviews 2016, Issue 5. Art. No.: CD003725. DOI: 10.1002/14651858.CD003725.pub4. Accessed 02 September 2025.	Excluded	Title/Abstract	
78	Donken CCMA, Al-Khateeb H, Verhofstad MHJ, van Laarhoven CJHM. Surgical versus conservative interventions for treating ankle fractures in adults. Cochrane Database of Systematic Reviews 2012, Issue 8. Art. No.: CD008470. DOI: 10.1002/14651858.CD008470.pub2. Accessed 02 September 2025.	Excluded	Title/Abstract	

79	van der Heijden RA, Lankhorst NE, van Linschoten R, Bierma-Zeinstra SMA, van Middelkoop M. Exercise for treating patellofemoral pain syndrome. Cochrane Database of Systematic Reviews 2015, Issue 1. Art. No.: CD010387. DOI: 10.1002/14651858.CD010387.pub2. Accessed 02 September 2025.	Excluded	Title/Abstract	
80	Wallis JA, Bourne AM, Jessup RL, Johnston RV, Frydman A, Cyril S, Buchbinder R. Manual therapy and exercise for lateral elbow pain. Cochrane Database of Systematic Reviews 2024, Issue 5. Art. No.: CD013042. DOI: 10.1002/14651858.CD013042.pub2. Accessed 02 September 2025.	Excluded	Title/Abstract	
81	Fee PA, Macey R, Walsh T, Clarkson JE, Ricketts D. Tests to detect and inform the diagnosis of root caries. Cochrane Database of Systematic Reviews 2020, Issue 12. Art. No.: CD013806. DOI: 10.1002/14651858.CD013806. Accessed 02 September 2025.	Excluded	Title/Abstract	
82	Guo J, Li C, Zhang Q, Wu G, Deacon SA, Chen J, Hu H, Zou S, Ye Q. Secondary bone grafting for alveolar cleft in children with cleft lip or cleft lip and palate. Cochrane Database of Systematic Reviews 2011, Issue 6. Art. No.: CD008050. DOI: 10.1002/14651858.CD008050.pub2. Accessed 03 September 2025	Excluded	Title/Abstract	
83	Mehrholz J, Pohl M, Platz T, Kugler J, Elsner B. Electromechanical and robot-assisted arm training for improving activities of daily living, arm function, and arm muscle strength after stroke. Cochrane Database of Systematic Reviews 2018, Issue 9. Art. No.: CD006876. DOI: 10.1002/14651858.CD006876.pub5. Accessed 03 September 2025.	Excluded	Title/Abstract	
84	Amatya B, Young J, Khan F. Non-pharmacological interventions for chronic pain in multiple sclerosis. Cochrane Database of Systematic	Excluded	Title/Abstract	

	Reviews 2018, Issue 12. Art. No.: CD012622. DOI: 10.1002/14651858.CD012622.pub2. Accessed 03 September 2025.			
85	Showell MG, Cole S, Clarke MJ, DeVito NJ, Farquhar C, Jordan V. Time to publication for results of clinical trials. Cochrane Database of Systematic Reviews 2024, Issue 11. Art. No.: MR000011. DOI: 10.1002/14651858.MR000011.pub3. Accessed 03 September 2025.	Excluded	Title/Abstract	
86	Mohammed Meeran RA, Durairaj V, Sekaran P, Farmer SE, Pandyan AD. Assistive technologies, including orthotic devices, for the management of contractures in adults after a stroke. Cochrane Database of Systematic Reviews 2024, Issue 9. Art. No.: CD010779. DOI: 10.1002/14651858.CD010779.pub2. Accessed 03 September 2025.	Excluded	Title/Abstract	
87	Macey R, Walsh T, Riley P, Glenny A-M, Worthington HV, Clarkson JE, Ricketts D. Electrical conductance for the detection of dental caries. Cochrane Database of Systematic Reviews 2021, Issue 3. Art. No.: CD014547. DOI: 10.1002/14651858.CD014547. Accessed 03 September 2025.	Excluded	Title/Abstract	
88	Yeung SS, Yeung EW, Gillespie LD. Interventions for preventing lower limb soft-tissue running injuries. Cochrane Database of Systematic Reviews 2011, Issue 7. Art. No.: CD001256. DOI: 10.1002/14651858.CD001256.pub2. Accessed 03 September 2025.	Excluded	Title/Abstract	
89	Claireaux HA, Searle HKC, Parsons NR, Griffin XL. Interventions for treating fractures of the distal femur in adults. Cochrane Database of Systematic Reviews 2022, Issue 10. Art. No.: CD010606. DOI: 10.1002/14651858.CD010606.pub3. Accessed 03 September 2025.	Excluded	Title/Abstract	
90	French HP, Abbott JH, Galvin R. Adjunctive therapies in addition to land-based exercise therapy for osteoarthritis of the hip or knee. Cochrane Database of Systematic Reviews 2022, Issue 10. Art. No.:	Excluded	Title/Abstract	

	CD011915. DOI: 10.1002/14651858.CD011915.pub2. Accessed 03 September 2025.			
91	Arora A, Kumbargere Nagraj S, Khattri S, Ismail NM, Eachempati P. School dental screening programmes for oral health. Cochrane Database of Systematic Reviews 2022, Issue 7. Art. No.: CD012595. DOI: 10.1002/14651858.CD012595.pub4. Accessed 03 September 2025.	Excluded	Title/Abstract	
92	Braun C, McRobert CJ. Conservative management following closed reduction of traumatic anterior dislocation of the shoulder. Cochrane Database of Systematic Reviews 2019, Issue 5. Art. No.: CD004962. DOI: 10.1002/14651858.CD004962.pub4. Accessed 03 September 2025	Excluded	Title/Abstract	
93	Pedlow K, McDonough S, Lennon S, Kerr C, Bradbury I. Assisted standing for Duchenne muscular dystrophy. Cochrane Database of Systematic Reviews 2019, Issue 10. Art. No.: CD011550. DOI: 10.1002/14651858.CD011550.pub2. Accessed 03 September 2025.	Excluded	Title/Abstract	
94	Poolman RW, Goslings JC, Lee J, Statius Muller M, Steller EP, Struijs PAA. Conservative treatment for closed fifth (small finger) metacarpal neck fractures. Cochrane Database of Systematic Reviews 2005, Issue 3. Art. No.: CD003210. DOI: 10.1002/14651858.CD003210.pub3. Accessed 03 September 2025.	Excluded	Title/Abstract	
95	White CM, van Doorn PA, Garssen MPJ, Stockley RC. Interventions for fatigue in peripheral neuropathy. Cochrane Database of Systematic Reviews 2014, Issue 12. Art. No.: CD008146. DOI: 10.1002/14651858.CD008146.pub2. Accessed 03 September 2025	Excluded	Title/Abstract	
96	Chin B, Wee I, Syn NLX, O'Neill GK, Yap ES, Koh PL. Surgery for chronic arthropathy in people with haemophilia. Cochrane Database of	Excluded	Title/Abstract	

	Systematic Reviews 2022, Issue 11. Art. No.: CD013634. DOI: 10.1002/14651858.CD013634.pub2. Accessed 03 September 2025.			
97	Li S, Yu B, Zhou D, He C, Zhuo Q, Hulme JM. Electromagnetic fields for treating osteoarthritis. Cochrane Database of Systematic Reviews 2013, Issue 12. Art. No.: CD003523. DOI: 10.1002/14651858.CD003523.pub2. Accessed 03 September 2025.	Excluded	Title/Abstract	
98	Bina S, Pacey V, Barnes EH, Burns J, Gray K. Interventions for congenital talipes equinovarus (clubfoot). Cochrane Database of Systematic Reviews 2020, Issue 5. Art. No.: CD008602. DOI: 10.1002/14651858.CD008602.pub4. Accessed 03 September 2025.	Excluded	Title/Abstract	
99	Cooper AM, O'Malley LA, Elison SN, Armstrong R, Burnside G, Adair P, Dugdill L, Pine C. Primary school-based behavioural interventions for preventing caries. Cochrane Database of Systematic Reviews 2013, Issue 5. Art. No.: CD009378. DOI: 10.1002/14651858.CD009378.pub2. Accessed 03 September 2025.	Excluded	Title/Abstract	
100	Daly BJM, Sharif MO, Jones K, Worthington HV, Beattie A. Local interventions for the management of alveolar osteitis (dry socket). Cochrane Database of Systematic Reviews 2022, Issue 9. Art. No.: CD006968. DOI: 10.1002/14651858.CD006968.pub3. Accessed 03 September 2025.	Excluded	Title/Abstract	
101	Ada L, Foongchomcheay A, Canning CG. Supportive devices for preventing and treating subluxation of the shoulder after stroke. Cochrane Database of Systematic Reviews 2005, Issue 1. Art. No.: CD003863. DOI: 10.1002/14651858.CD003863.pub2. Accessed 03 September 2025.	Excluded	Title/Abstract	

102	Mehrholz J, Kugler J, Pohl M, Elsner B. Electromechanical-assisted training for walking after stroke. Cochrane Database of Systematic Reviews 2025, Issue 5. Art. No.: CD006185. DOI: 10.1002/14651858.CD006185.pub6. Accessed 03 September 2025	Excluded	Title/Abstract	
103	Heintjes EM, Berger M, Bierma-Zeinstra SMA, Bernsen RMD, Verhaar JAN, Koes BW. Pharmacotherapy for patellofemoral pain syndrome. Cochrane Database of Systematic Reviews 2004, Issue 3. Art. No.: CD003470. DOI: 10.1002/14651858.CD003470.pub2. Accessed 03 September 2025.	Excluded	Title/Abstract	
104	Cheuk DKL, Wong V, Wraige E, Baxter P, Cole A. Surgery for scoliosis in Duchenne muscular dystrophy. Cochrane Database of Systematic Reviews 2015, Issue 10. Art. No.: CD005375. DOI: 10.1002/14651858.CD005375.pub4. Accessed 03 September 2025.	Excluded	Title/Abstract	
105	Kerkhoffs GMMJ, Handoll HHG, de Bie R, Rowe BH, Struijs PAA. Surgical versus conservative treatment for acute injuries of the lateral ligament complex of the ankle in adults. Cochrane Database of Systematic Reviews 2007, Issue 2. Art. No.: CD000380. DOI: 10.1002/14651858.CD000380.pub2. Accessed 03 September 2025	Excluded	Title/Abstract	
106	Dwan K, Phillipi CA, Steiner RD, Basel D. Bisphosphonate therapy for osteogenesis imperfecta. Cochrane Database of Systematic Reviews 2016, Issue 10. Art. No.: CD005088. DOI: 10.1002/14651858.CD005088.pub4. Accessed 03 September 2025.	Excluded	Title/Abstract	
107	Karjalainen TV, Silagy M, O'Bryan E, Johnston RV, Cyril S, Buchbinder R. Autologous blood and platelet-rich plasma injection therapy for lateral elbow pain. Cochrane Database of Systematic Reviews 2021, Issue 9. Art. No.: CD010951. DOI: 10.1002/14651858.CD010951.pub2. Accessed 03 September 2025.	Excluded	Title/Abstract	

108	Wang C, Shang Y, Kanaan G, Chai L, Li H, Qi X. Silymarin for adults with metabolic dysfunction-associated steatotic liver disease. Cochrane Database of Systematic Reviews 2025, Issue 6. Art. No.: CD015524. DOI: 10.1002/14651858.CD015524.pub2. Accessed 03 September 2025.	Excluded	Title/Abstract	
109	Li H, Guo Z, Li C, Ma X, Wang Y, Zhou X, Johnson TM, Huang D. Materials for retrograde filling in root canal therapy. Cochrane Database of Systematic Reviews 2021, Issue 10. Art. No.: CD005517. DOI: 10.1002/14651858.CD005517.pub3. Accessed 03 September 2025.	Excluded	Title/Abstract	
110	Neubert A, Jaekel C, Metzendorf M-I, Richter B. Surgical versus non-surgical interventions for treating ankle fractures in adults. Cochrane Database of Systematic Reviews 2021, Issue 10. Art. No.: CD014930. DOI: 10.1002/14651858.CD014930. Accessed 03 September 2025.	Excluded	Title/Abstract	
111	Harris R, Raison H, Christian B, Bakare L, Okwundu CI, Burnside G. Interventions for improving adults' use of primary oral health care services. Cochrane Database of Systematic Reviews 2017, Issue 8. Art. No.: CD012771. DOI: 10.1002/14651858.CD012771. Accessed 03 September 2025.	Excluded	Title/Abstract	
112	Jen R, Almeida FR, Brasher P, Doyle-Waters MM, Salzman J, Fleetham J. Oral appliances for obstructive sleep apnoea. Cochrane Database of Systematic Reviews 2018, Issue 9. Art. No.: CD013131. DOI: 10.1002/14651858.CD013131. Accessed 03 September 2025.	Excluded	Title/Abstract	
113	Mao S, Liu L, Wang T, Cao Y. Community health worker-led interventions for hypertension. Cochrane Database of Systematic Reviews 2025, Issue 5. Art. No.: CD016048. DOI: 10.1002/14651858.CD016048. Accessed 03 September 2025.	Excluded	Title/Abstract	

**3. ScienceDirect**				
**Search date**	25/8/2025			
**Results**	289			
**Search Query**	TITLE-ABS-KEY((Orthodontic* OR "orthodontic treatment" OR braces OR "clear aligners" OR Invisalign) AND ("Social Media" OR "social network*" OR Facebook OR Instagram OR TikTok OR YouTube OR Twitter OR X OR Snapchat) AND (willing* OR motivat* OR intent* OR perception OR attitude* OR "decision making" OR preference* OR "patient acceptance"))			
**Nr.**	**Title**	**Authors**	**Eligibility**	**Reason for exclusion**
1	The quality of clear aligner therapy information on the internet: A scoping review	Maurice J Meade, Emi lija Jensen, Br ianna Poirier	Excluded	Title/Abstract
2	Instagram and clear aligner therapy: A content analysis of patient perspectives	Vincenzo Gra ssia , Fabrizia d'Apuzzo, R eem A. Alansari, Abdolreza Ja milian, Babak Sayahpour, S amar	Excluded	Title/Abstract

		M. Adel, Ludovica Nucci		
3	Exploring the pros and cons of social media in orthodontics: A critical analysis of the perspectives of patients and professionals	Dr Om Prakash Kharbanda , Dr Tabassum Qureshi , Dr Kaleem Fatima , Dr Mayank Khandelwal	Excluded	Title/Abstract
4	Orthodontic correction of Class II skeletal malocclusion complicated by deep bite using clear aligners: A case report	Rawah T. Eshky Diplomate of ABO	Excluded	Title/Abstract
5	The quality of orthognathic surgery information on social media: A scoping review	Mimi Ngo, Emilija Jensen, Maurice Meade	Excluded	Title/Abstract

6	World Federation of Orthodontists social media guidelines: Ensuring accuracy, reliability, and objectivity in online orthodontic information	Narayan H Gandedkar , Bryce PH Lee, Val my Pangrazio-Kulbersh, Reem Alansari, Jorge L Castillo, Ricardo M Cruz, Derya G Çakan , Kelvin WC Foong , Jae H Park , Young G Park, Aron Dellinger, Letizia Perillo, Allan R Thom , Nikhillesh Vaiid	DUPLICATE PUB 75	
7	Influence of social media and corrected smile photographs in patients with malocclusion	Rohit Karkun , Puneet Batr	DUPLICATE PUB 115	

		a, Ashish Kumar Singh		
8	Orthodontics in the era of social media: An interview with orthodontic journal editors	Dalya Al-Moghrabi , Martyn T. Cobourne , Padhraig S. Fleming , Neal D. Kravitz , Vinod Krishnan , Eric Jein-Wein Liou , Ambra Michelotti , Ravindra Nanda , Cagla Sar, Nikhilesh R. Vaid	Excluded	Title/Abstract
9	Sharing and assessing the impact of orthodontic research on social media: The case of altmetrics	Christos Livas , Konstantina Delli	Excluded	Title/Abstract
10	Evolutionary Trend of Dental Health Care Information on Chinese Social Media Platforms During 2018-2022: Retrospective Observational Study	Zhiyu Zhu, Zhiyun Ye, Qian Wang , Ruomei Li, Hairui Li, Weiming		Title/Abstract

		Guo, Zhenxia Li, Lunguo Xia, Bing Fang		
11	From an entertaining app to a search engine: A case of user-generated innovation in social media platforms	Mashael Almoqbel, Jana Alrassi, Sara Alzahrani, Hajar Alrashidi, Sabah Jarallah Aldoshan, Abdullah Alatiqi	Excluded	Title/Abstract
12	Social media use among orthodontic professionals: Present and future	Sarah AbuArqub, Dalya Al-Moghrabi, Aslam Alkadhimi, Padhraig S. Fleming		Title/Abstract
13	Viewing of clinical cases on social media by dentists: A cause of motivation or dissatisfaction?	Tazeen Zehra FCPS, Juzer Shabbir, Naheed Najmi, Arshad Hasan PhD, Arifa Haque, Waqas	Excluded	Title/Abstract

		A. Farooqui , Zohaib Khurshid Mres		
14	The role of orthodontic societies online and on social media: Insights from UK campaigns	Dalya Al-Moghrabi , Simon J Littlewood , Karen Juggins , Andrew Flett , Anshu Sood	Excluded	Title/Abstract
15	The Impact of Social Media Posts Concerning Esthetic Dentistry on Self-Esteem and Satisfaction Among Adults in Saudi Arabia	Ghadah Alhuzili, Asmaa Alghamdi, Rawan Alhazmy, Haneen Abdullah , Shahinaz Sembawa, Waad Khayat	Excluded	Title/Abstract
16	Social media and orthodontics: A mixed-methods analysis of orthodontic-related posts on Twitter and Instagram	Isabelle Graf, Hanna Gerwing, Karolin Hoefer, Daniel Ehlebracht, H	DUPLICATE PUB 83	

		ildegard Christ, Bert Braumann		
17	Generational perspectives of orthodontists in the U.S. and Canada: A survey study	Syed Rassal Hussain a, Shuying S. Jiang b, Jose A. Bosio a	DUPLICATE PUB 100	
18	Tele-orthodontics: Reliability of virtually obtained records for orthodontic decision-making — a pilot study	Ronald Lowe a, Gregory Wilding b, Ashish Gurav c, William Tanberg d, Thikriat Al-Jewair e	Excluded	Title/Abstract
19	Determining the impact of orthodontic patients' characteristics on their usage and preferences of social media	Talat H. Al-Gunaid, Afnan A. Aljohani, Khaled M. Alhazmi, Ahmed M. Ibrahim	DUPLICATE PUB 9	
20	Is social media the way to empower patients to share their experiences of dental care?	Sophy K. Barber MSc, Yung Lam BDS, Trevor	Excluded	Title/Abstract

		M. Hodge M Phil, Susan P avitt		
21	YouTube as a source of information on adult orthodontics: a video analysis study	Mehmet Ali Yavan, G ökçenur Gök çe	Excluded	Title/Abstract
22	Effect of social media in improving knowledge among patients having fixed appliance orthodontic treatment: A single-center randomized controlled trial	Fadi M. Al-Silwadi, Dalji t S. Gill, Aviva Petrie, Susan J. Cunningha m	Excluded	Title/Abstract
23	Adult orthodontics, motivations for treatment, choice, and impact of appliances: A qualitative study	Ama Johal, S hahad Hatam Dama nhuri, Fiorell a Colonio-Salazar	Excluded	Title/Abstract

24	Facebook marketing in contemporary orthodontic practice: A consumer report	Trent Cox , Ja e Hyun Park	Excluded	Full text, Outcome not relevant — the study assessed perceptions of Facebook marketing strategies rather than patients' willingness to initiate orthodontic treatment
25	Orthodontics in an online community: A computational analysis of r/Braces subreddit	Mehmet Ali Yavan, D erviş Emre Ercan	Excluded	Title/Abstract

26	Foundation models and intelligent decision-making: Progress, challenges, and perspectives	Jincai Huang, Yongjun Xu, Qi Wang, Qi (Cheems) Wang, Xingxing Liang , Fei Wang, Zhao Zhang, Wei Wei , Boxuan Zhang, Libo Huang, Jingru Chang , Liantao Ma, Ting Ma, Yuxuan Liang, Jie Zhang, Jian Guo, Xuhui Jiang, Xinxin Fan, Zhulin An , Tingting Li, Xuefei Li, Zezhi Shao, Tangwen Qian , Tao Sun, Boyu Diao, Chuanguang Yang, Chenqing Yu, Yi	Excluded	Title/Abstract

		qing Wu, Mengxian Li, Haifeng Zhang, Yongcheng Zeng, Zhicheng Zhang, Zhengqiu Zhu, Yiqin Lv, Aming Li, Xu Chen, Bo An , Wei Xiao , Chenguang Bai, Yuxing Mao, Zhigang Yin, Sheng Gui, Wentao Su, Yinghao Zhu, Junyi Gao, Xinyu He, Yizhou Li, Guangyin Jin , Xiang Ao, Xuehao Zhai, Haoran Tan, Lijun Yun , Hongquan Shi, Jun Li, Changjun Fan,		

		Kuihua Huang, Ewen Harrison, Victor C.M. Leung, Sihang Qiu, Yanjie Dong, Xiaolong Zheng, Gang Wang, Yu Zheng, Yuanzhuo Wang, Jiafeng Guo, Lizhe Wang, Xueqi Cheng, Yaonan Wang, Shanlin Yang, Mengyin Fu, Aiguo Fei		

27	Understanding orthodontists' decision making in relation to innovations from encounter to implementation: A qualitative study	Margarita Papakostopoulou, Josef Kučera, Hana Tycová	Excluded	Title/Abstract
28	A questionnaire study regarding knowledge, attitude and usage of artificial intelligence and machine learning by the orthodontic fraternity of Northern India	Arvind Mengi, Ravnitya Pal Singh, Nancy Mengi, Sneh Kalgotra, Abhishek Singh	DULPICATE PUB 152	
29	Adult patient preference for an orthodontic care provider in Canada	Matthew Brown, William Wiltshire, Fabio Henrique de Sa Leitao Pinheiro, Dieter J. Schönwetter	Excluded	Title/Abstract
30	The applications of digital technology in postgraduate orthodontic education	Divakar Karanth, Sarah Abu Arqub, Calogero Dolce	Excluded	Title/Abstract

31	Knowledge, attitude, and perception of orthodontic students, and orthodontists regarding role of artificial intelligence in field of orthodontics—An online cross-sectional survey	Seema Gupta, Santosh Verma, Arun K. Chauhan, Mainak Saha Roy, Wangonsana Rajkumari, Chirag Sahgal	Excluded	Title/Abstract
32	Exploring experiences of orthognathic surgery: A Reddit-based content analysis	Mimi Ngo, Emilija Jensen, Maurice Meade	Excluded	Title/Abstract
33	The last decade in orthodontics: A scoping review of the hits, misses and the near misses!	Narayan H. Gandedkar, Nikhilesh R. Vaid, M. Ali Darendeliler , Pratik Premjani , Donald J. Ferguson	Excluded	Title/Abstract
34	Evaluation of Korean-Language COVID-19–Related Medical Information on YouTube: Cross-Sectional Infodemiology Study	Hana Moon, Geon Ho Lee	Excluded	Title/Abstract
35	Decoding the nuances of scholarly output and publication metrics in orthodontics	Narayan H. Gandedkar, Veerasathp	Excluded	Title/Abstract

		urush Allare ddy, Nikhille sh Vaiid		
36	Main uses of Instagram in oral health research–A scoping review	Leandro Machado Oli veira, Kimber ly da Silva Pilecco, Daniel Fagundes de Souza, Cícero Anghinoni d e Oliveira, Fabr ício Batistin Zana tta	Excluded	Title/Abstract
37	Analysis of the information contained within TikTok videos regarding orthodontic retention	Maurice J. Meade, Cra ig W. Dreyer	Excluded	Title/Abstract
38	Dental Fear and Anxiety in Children and Adolescents: Qualitative Study Using YouTube	Xiaoli Gao, S H Hamzah , Cynthia Kar Yung Yiu, Colman McG rath, Nigel M King	Excluded	Title/Abstract

39	What is the current state of artificial intelligence applications in dentistry and orthodontics?	Paul Fawaz, Patrick El Sayegh, Ba rt Vande Vanne t	Excluded	Title/Abstract
40	Exploring the informal learning of zero waste lifestyle in Malaysia with big data analytics	Nur Suhaila Zulki fli, Latifah A bd Manaf	Excluded	Title/Abstract
41	Patient considerations for orthodontists: A comparative study of university students in Malaysia and Taiwan	Tee Chi Yuo , Joh nson Hsin-Chung Chen g, Daniel De-Shing Chen, Renu Sarah Samso n, Eby Vargh ese, Od Bayar saikhan	DUPLICATE PUB 240	
42	A comparison of patient testimonials on YouTube of the most common orthodontic treatment modalities: Braces, in-office aligners, and direct-to-consumer aligners	Riley J. Hunsaker, Bhavna Shrof f, Caroline Ca rrico, Brenna n Alford, Ste	Excluded	Title/Abstract

		ven<br>J. Lindauer		
43	An analysis of dental articles in predatory journals and associated online engagement	Dalya Al-Moghrabi, Rana S Albishri, Rahaf D Alshehri, Sarah Abu Arqub, Aslam Alkadhimi, Padhraig S Fleming	Excluded	Title/Abstract
44	Direct to consumer orthodontics: Exploring patient demographic trends and preferences	Brady C. Okuda, Sawsan Tabbaa, Michelle Edmonds, Youssef Toubouti, Humam Saltaji	Excluded	Title/Abstract
45	Virtual-First: A virtual workflow for new patient consultation, engagement and education in orthodontics	Vandana Katyal, Nikhilesh Vaid	Excluded	Title/Abstract

46	Factors Influencing the Decision Process within Seeking Orthodontic Care among the Saudi Population: A Cross-sectional Survey	Raghad Alharbi, Waleed Taju	Excluded	Full text, Does not refer to social media influece
47	Impact of COVID-19 Dentistry-Related Literature: An Altmetric Study	Konstantina Delli, Christos Livas, Nikolaos G. Nikitakis, Arjan Vissink	Excluded	Title/Abstract
48	Tweeting for Health Using Real-time Mining and Artificial Intelligence–Based Analytics: Design and Development of a Big Data Ecosystem for Detecting and Analyzing Misinformation on Twitter	Plinio Pelegrini Morita PEng, Irfhana Zakir Hussain BTech, Jasleen Kaur BTech, MTech, PhD, Matheus Lotto, Zahid Ahmad Butt MBBS,	Excluded	Title/Abstract
49	Association between crowding estimation and extraction recommendations in orthodontics	Kaitlyn Tom, Lloyd Mancl, Heather Woloshyn, Roozbeh Khosravi,	DUPLICATE PUB 220	

		Anne-<br>Marie Bollen		
50	Patients' perceptions matter: Risk communication and psychosocial factors in orthodontics	Yifan Zhao, Xinyi Shi, Junqi Liu, Rongrong Huo, Kai Xia, Yifan Wang, Guanyin Zhu, Wenxin Lu, Lan Zhang, Yajing Meng,<br>Zhihe Zhao	Excluded	Title/Abstract
51	The influence of women in shaping the specialty of orthodontics	Nellie Kim-Weroha, Su-Jung Kim	Excluded	Title/Abstract
52	Analysis of Fluoride-Free Content on Twitter: Topic Modeling Study	Matheus Lotto, Irfhana Zakir<br>Hussain BTech, Jasleen Kaur BTech,<br>MTech,<br>PhD, Zahid Ahmad Butt MBBS, Thiag	DUPLICATE PUB 139	

		o Cruvinel, P linio P Morita Ben g		
53	Factors Affecting the Decision-making of Direct Pulp Capping Procedures among Dental Practitioners: A Multinational Survey from 16 Countries with Meta-analysis	Ömer Hatipo ğlu , Fatma Pertek Hatip oğlu , Muha mmad Qasim Javed BDS, FCPS, Kacper Nijakowski, Nessrin Taha ,Christina El-saaidi BDS, MPH, Surend ar Sugumara n BDS, MDS, Yasmin e Elhamouly BDS,, Milan Drobac, Ricar do Machado, Thiyezen Abdullah Al	Excluded	Title/Abstract

		dhelai BDS, Hiro Kobayashi JD, Suha Alfirjani BDS, , Imran Zainal Abidin BDS, Benjamín Martin-Biedma PhD, Kopbayeva Maira DSc, Wen Yi Lim, Paulo J. Palma, João Filipe Brochado Martins		
54	Dentistry Students' Attitudes of PhD and Specialty Education	Özlem Çimen, Parla Meva Durmazpınar	Excluded	Title/Abstract
55	HOW PRO'S CAN CONTRIBUTE TO WHAT MATTERS MOST TO PATIENTS WITH OROFACIAL CLEFTS	Edwin M. Ongkosuwito, Mette A.R. Kuijpers	DUPLICATE 166	

56	“Airway Friendly” orthodontics! Are you serious?	Daniel J. Rinchuse	Excluded	Title/Abstract
57	Characterization of False or Misleading Fluoride Content on Instagram: Infodemiology Study	Matheus Lotto, Tamires Sá Menezes , Irfhana Zakir Hussain BTech, Shu-Feng Tsao , Zahid Ahmad Butt MBBS, Plinio P Morita BEng, Thiago Cruvinel	DUPLICATE 137	
58	Occlusal device therapy for sleep bruxism: Analysis of educational value, clarity, reliability, understandability, and actionability of information of content on YouTube	Tay Xiao Thong, Jennese Moo Wann Wai, Smita Nimbalkar, Pravinkumar G. Patil	Excluded	Title/Abstract

59	Machine learning and orthodontics, current trends and the future opportunities: A scoping review	Hossein Mohammad-Rahimi, Mohadeseh Nadimi, Mohammad Hossein Rohban, Erfan Shamsoddin, Victor Y. Lee, Saeed Reza Motamedian	Excluded	Title/Abstract
60	Perspectives of Dental Students and Faculty about Evidence-Based Dental Practice	Ahmad Abdelkarim, Donna Sullivan	DUPLICATE PUB 1	
61	When convenience trumps quality of care: A population-based survey on direct to consumer orthodontics	Rany M. Bous, Konstantinos Apostolopoulos, Manish Valiathan	Excluded	Title/Abstract

62	Psychophysiological Reactions of Internet Users Exposed to Fluoride Information and Disinformation: Protocol for a Randomized Controlled Trial	Matheus Lotto, MSc, Olivia Santana Jorge, Tamires Sá Menezes, Ana Maria Ramalho, Thais Marchini Oliveira, Fernando Bevilacqua, Thiago Cruvinel	Excluded	Title/Abstract
63	Predoctoral orthodontic education in the United States: Challenges and opportunities for generation Z learners	Maria Therese Galang-Boquiren, Negin Katebi, Christine Hong, Mitchell Lipp	Excluded	Title/Abstract
64	In search of the sample: Recent experiences of a trial team in Orthodontics	Susan Cunningham, David Bearn, Philip Benson, A	Excluded	Title/Abstract

		ma Johal, Declan Millett, Kevin O'Brien, Friedy Luther		
65	Performance in answering orthodontic patients' frequently asked questions: Conversational artificial intelligence versus orthodontists	Xinlianyi Zhou, Yao Chen, Ehab A. Abdulghani, Xu Zhang, Wei Zheng, Yu Li	Excluded	Title/Abstract
66	A clinician's perspective on indications and failures of bone-borne maxillary expanders	Biondi Guglielmo, Ludwig Björn, Mura Rossano, Di Leonardo Bruno, Adith Venugopal	Excluded	Title/Abstract
67	Intra- and inter-office communication: Important in achieving optimal treatment outcomes and patient satisfaction	Mary Eve Maestre , Robert Peracchia, George J. Cisneros	Excluded	Title/Abstract

68	The online attention analysis on orthognathic surgery research	Ricardo Grillo, Yunus Balel, Bruno Alvarez Quinta Reis, Dani Stanbouly, Sahand Samieirad, Fernando Melhem-Elias	Excluded	Title/Abstract
69	Urgencies and emergencies in orthodontics during the coronavirus disease 2019 pandemic: Brazilian orthodontists' experience	Paula Cotrin, Renan Morais Peloso, Nubia Inocencya Pavesi Pini, Renata Cristina Oliveira, Ricardo Cesar Gobbi de Oliveira, Fabricio Pinelli Valarelli, Karina Maria	Excluded	Title/Abstract

		Salvatore Frei tas		
70	Treatment motivation, psychosocial impact, and perfectionism in children and adult orthodontic patients: A cross-sectional study	Huanzhuo Z hao, Baraa D araqel, Man J iang, Tianci Z hang, Xiang Li, Jicheng Su n, Leilei Zhen g	Excluded	Title/Abstract
71	Insights into the hotspots and frontiers of research on design for sustainable behavior	Ouyang Lan, Lu Shizhu	Excluded	Title/Abstract
72	Compliance of orthodontic practice websites with ethical, legal and regulatory advertising obligations	Maurice J. Meade, Xiangqun Ju, David Hunter, Lisa Jamieson	Excluded	Title/Abstract
73	The knowledge, experience, and attitude on artificial intelligence-assisted cephalometric analysis: Survey of orthodontists and orthodontic students	Lizhuo Lin, B ojun Tang, Li ngyun Cao, Ji arong Yan, Ti ngting Zhao, Fang Hu, Ho ng He	Excluded	Title/Abstract

74	A cross-sectional analysis of the reliability, content and readability of orthodontic retention and retainer informed consent forms	Maurice J. Meade, Sven Jensen, Xiangqun Ju, David Hunter, Lisa Jamieson	Excluded	Title/Abstract
75	Mapping the scholarly landscape of TikTok (Douyin): A bibliometric exploration of research topics and trends	Abderahman Rejeb, Karim Rejeb, Andrea Appolloni, Horst Treiblmaier, Mohammad Iranmanesh	Excluded	Title/Abstract
76	A knowledge translation perspective on improving adherence to removable appliances in adolescents	Akriti Tiwari, Arnaldo Perez-Garcia, Carlos Flores-Mir	Excluded	Title/Abstract
77	Identifying factors that impact general dentists' referrals to orthodontists	Daniel S. Lee, Taylor Sulkowski, Clare Bocklage, Sylvia A. Frazier-Bowers, Chris Wiesen, Pa	Excluded	Title/Abstract

		ul Mihas, Laura A. Jacox		
78	Autonomy and consent in this era of unconscious priming	Adith Venugopal, Carlos Flores-Mir, Nikhilesh R. Vaid	Excluded	Title/Abstract
79	Dentists' Self-evaluated Ability in Diagnosing and Updating About Pulpotomy	Beatriz Della Terra Mouco Garrido, Luciana Lourenço Ribeiro Vitor, Thiago Cruvinel, Maria Aparecida Andrade Moreira Machado, Thais Marchini Oliveira, Natalino Lourenço Neto	Excluded	Title/Abstract
80	Orthondontic marketing. The issue of information vs. trust; supporting future patients in their choices when accessing online information	Neil Hillyard	Excluded	Title/Abstract

81	A Cross-Sectional Online Survey on Knowledge, Awareness, and Perceptions of Hollywood Smile Among the Saudi Arabia Population	Mohammed Abdulrahman Alsurayyi, Wafi Almutairi, Abdulrahman Ismail Binsaeed, Sami Aldhuwayhi, Saquib Ahmed Shaikh, Mohammed Ziauddeen Mustafa	Excluded	Title/Abstract
82	Antecedents of effectuation and causation in SMEs from emerging markets: the role of CEO temporal focus	Ekaterina Kozachenko, Galina Shirokova, Virginia Bodolica	Excluded	Title/Abstract
83	The quality of Web-based orthodontic information: A scoping review	Maurice J. Meade, Brianna Poirier, Emilija D. Jensen	Excluded	Title/Abstract

84	Assessing dentists' awareness of the orthodontic–restorative interface	Revathi Arunachalam, Neil Nathwani, Touraj Nejatian, Peter Fine, Albert Leung	DUPLICATE PUB 30	
85	World Federation of Orthodontists guidelines for postgraduate orthodontic education	Takashi Ono, Valmy Pangrazio-Kulbersh, Letizia Perillo, Flavia Artese, Ewa Czochrowska, M. Ali Darendeliler, Steven Dugoni, William E. Fayad Bajaire, Eric Liou, Jae Hyun Park, David P. Rice, Abbas Zaher, Athanasios E. Athanasio	Excluded	Title/Abstract

		u, Lee W. Graber, N ikhilesh R. Vaid		
86	Quality assessment of health science-related short videos on TikTok: A scoping review	Bingyan Li, Min Liu, Jia L iu, Yuxi Zhan g, Wenjuan Y ang, Lunfang Xie	Excluded	Title/Abstract
87	Decision on the management of teeth with advanced attachment loss: Effects of dentists' specialty and clinical experience	Mayara Boss ardi, Eliseu Aldrighi Mü nchow, Patríc ia Weidlich	Excluded	Title/Abstract
88	Marketing Directly to Patients	Benjamin Bur ris, Jeff Behan , Robert Pickr on, Bill Picka rd, Kelly Ferg	Excluded	Title/Abstract

		us, Mark L. Dake		
89	Educational debt and the gender gap: Understanding factors influencing orthodontists' career decisions	Catherine Campbell Wo rthington , Pa ul Mihas, Cla re Bocklage, Sylvia A. Frazier-Bowers, Feng - Chang Lin, C hing-Chang Ko, La ura Anne Jacox	Excluded	Title/Abstract
90	Impact of the coronavirus disease 2019 pandemic on orthodontic patients and their attitude to orthodontic treatment	Onyinye Dorothy Um eh , Ifeoma Linda Utomi, Ikenna Gerald Isiek we, Emmanuel T. Aladenika	Excluded	Title/Abstract

91	Can Large Language Models Serve as Reliable Tools for Information in Dentistry? A Systematic Review	Nora Alhazmi, Aram Alshehri, Fahad BaHammam b, Manju Philip, Muhammad Nadeem, Sanjeev Khanagar	Excluded	Title/Abstract
92	Trends and insights in animal and biomedical research: A 5-year scientometric analysis of orthodontic peer-reviewed journals	Hams H. Abdelrahman, Yomna M. Yacout, Dina G. Hassan, Mohamed G. Hassan, Gamal A. Hassan	Excluded	Title/Abstract
93	Practice profiles of orthodontists in Taiwan	Johnson Hsin-Chung Cheng, Chia-Hui Wu, Daniel De-Shing Chen	Excluded	Title/Abstract

94	Adolescent perceptions of orthodontic treatment risks and risk information: A qualitative study	John Perry, Ilona Johnson, Hashmat Popat, Maria Z. Morgan, Paul Gill	Excluded	Title/Abstract
95	Assessment of students' perspective on introduction of "digital dentistry" as self- directed learning module in undergraduate prosthodontics curriculum: A mixed-method study	Shuchi Tripathi, R.K. Dixit, Suyog Sindhu, R.D. Singh, Rameshwari Singhal , Richa Khanna	Excluded	Title/Abstract
96	CHAPTER 1 - The problem of compliance in orthodontics	Andrej Zentner	Excluded	Title/Abstract
97	The Use of Patient-Oriented Mobile Phone Apps in Oral Health: Scoping Review	Elina Väyrynen, Sanna Hakola, Anniina Keski-Salmi BDS, Hannaleena Jämsä, Raija Vainionpää, Saujanya Karki	Excluded	Title/Abstract

98	COVID-19 and Orthodontics: An Approach for Monitoring Patients at Home	Antonio Bianco, Domenico Dalessandri, Bruno Oliva, Ingrid Tonni, Gaetano Isola, Luca Visconti, Corrado Paganelli, Stefano Bonetti	Excluded	Title/Abstract
99	The home food environment for children in Northern China	R. Ma, X. Wang, Y.Y. Gong, H. Ensaff	Excluded	Title/Abstract
100	Considerations for orthodontists navigating career paths in Dental Support Organization	Jahnavi Rao, Richa Dutta	Excluded	Title/Abstract
101	Teledentistry and its applications in paediatric dentistry: A literature review	Harshita Sharma, Baranya Shrikrishna Suprabha, Arathi Rao	Excluded	Title/Abstract
102	A cost-effectiveness analysis of an endocrown versus complete crown	Nighat Naved, Asif R. Khowaja, Fahad Umer	Excluded	Title/Abstract
103	Barriers and risks of Mobility-as-a-Service (MaaS) adoption in cities: A systematic review of the literature	Luke Butler, Tan Yigitcanl	Excluded	Title/Abstract

		ar, Alexander Paz		
104	A scoping review of website-based orthognathic surgery information	Mimi K. Ngo, Nicholas Loo Yong Kee, Emilija D. Jensen, Maurice J. Meade, LLM	Excluded	Title/Abstract
105	Clinical Difficulties Related to Direct Composite Restorations: A Multinational Survey	Anna Lehmann , Kacper Nijakowski, Jakub Jankowski, David Donnermeyer, João Carlos Ramos, Milan Drobac, João Filipe Brochado Martins, Ömer Hatipoğlu, Bakhyt Omarova, Muhammad	Excluded	Title/Abstract

		Qasim Javed, Hamad Mohammad Alharkan, Olga Bekjanova, Sylvia Wyzga, Moataz-Bellah Ahmed Mohamed Alkhawas, Rutendo Kudenga, Anna Surdacka		
106	Essential factors in developing an efficient in-office aligner system	Rooz Khosravi, Ioanna Gidarakou, Tomas Salazar	Excluded	Title/Abstract
107	Information-seeking behavior of adolescent orthodontic patients	Rachel Stephens, Fiona S. Ryan, Susan J. Cunningham	Excluded	Title/Abstract

108	Quality assessment of online information on orthodontic Web sites in the United States	Nikolaos Ferlias, Katrine Smith, Agnes Straarup, Lorana Travancic, Kasper Dahl Kristensen, Peter Stoustrup	Excluded	Title/Abstract
109	AI Chatbots in Fintech Sector: A Study Towards Technological Convergence	Chandni Bansal , Ajay Kumar, Namrata Dogra, Gaydaa AlZohbi, Chand Prakash	Excluded	Title/Abstract
110	Where does evidence-based medicine sit with real world (clinical) medicine - IS IT TIME TO INVERT THE PYRAMID?	Kaval Patel Ute Schneider-Moser, Carmen Maria Costea, Lorenz Moser	Excluded	Title/Abstract
111	The key to successful online marketing for an orthodontic practice: Mastering the plan	Gaurav Gupta, Mary Kay Miller, Milind Darda	Excluded	Title/Abstract

112	Baseline Characteristics and Treatment Preferences of Oral Surgery Patients	Kathryn A. Atchison, Melanie W. Gironda, Edward E. Black, Stuart Schweitzer, Claudia Der-Martirosian, Alan Felsenfeld, MA, Richard Leathers DDS, Thomas R. Belin	Excluded	Title/Abstract
113	Health-Related Internet Use by Children and Adolescents: Systematic Review	Eunhee Park RN, PHNA-BC, PhD, Misol Kwon RN, BSN	Excluded	Title/Abstract
114	Artificial Intelligence: Applications in orthognathic surgery	P. Bouletreau, M. Makaremi, B. Ibrahim , A. Louvrier , N. Sigaux	Excluded	Title/Abstract

115	General dentists' treatment plans in response to cosmetic complains; a field study using unannounced-standardized-patient	Melika Hoseinzadeh, Afsoon Motallebi, Ali Kazemian	Excluded	Title/Abstract
116	26 - Esthetics and dental marketing	Edward Zuckerberg	Excluded	Title/Abstract
117	The reception of digital stories in dental education: Triggering thinking skills for empathy in undergraduate dentistry students	Burcu Şimşek, Çağla Karabağ, Şengül İnce, Evren Sertalp, Tolga F. Tözüm	Excluded	Title/Abstract
118	Comparison of patient factors influencing the selection of an orthodontist, general dentist, or direct-to-consumer aligners	Jeffrey C. Olson, Bhavna Shroff, Caroline Carrico, Joseph Boyle, Steven J. Lindauer	Excluded	Full text, No social media aspect
119	The quagmire of collegiality vs competitiveness	Adith Venugopal, Nikhilesh Vaid, S. Jay Bowman	Excluded	Title/Abstract
120	A qualitative evaluation of attitudes toward extractions among primary care orthodontists in Great Britain	Libby Richardson, Declan Millett, Philip	Excluded	Title/Abstract

		E. Benson, Susan J. Cunningham, Kara A. Gray-Burrows, Padhraig S. Fleming		
121	Evaluation of fear, anxiety, and knowledge among dental providers during the COVID 19 pandemic	Mayank Kakkar, Abdul Basir Barmak, Sangeeta Gajendra	Excluded	Title/Abstract
122	Growing production in dental practices	No authors available	Excluded	Title/Abstract
123	Negative online reviews of orthodontists: Content analysis of complaints posted by dissatisfied patients	Adam M. Skrypczak, William A. Tressel, Sara Ghayour, Roozbeh Khosravi, Douglas S. Ramsay	Excluded	Title/Abstract
124	WeChat as a Platform for Problem-Based Learning in a Dental Practical Clerkship: Feasibility Study	Wei Zhang DDS, MD, PhD, Zheng-Rong Li DDS,	Excluded	Title/Abstract

		Zhi Li DDS, MD, PhD		
125	Evaluation of orthodontists' experience with the surgery first protocol in orthodontic-surgical management	Victoria Damiano, Paul Fawaz, Bart Vande Vannet	Excluded	Title/Abstract
126	COVID-19 pandemic and pediatric dentistry: Fear, eating habits and parent’s oral health perceptions	Ricardo Campagnaro, Giulia de Oliveira Collet Mariana Podadeiro de Andrade, João Pedro da Silva Lopes Salles, Marina de Lourdes Calvo Fracasso, Debora Lopes Salles Scheffel, Karina Maria Salvatore Frei	Excluded	Title/Abstract

		tas, Gabriela Cristina Santin		
127	Online information on orthodontic treatment in Denmark: A population-based quality assessment	Nikolaos Ferlias, Michal Andrzej Ostapiuk, Troels Normann Diekema, Marcus Gaarde Kristensen, Kasper Dahl Kristensen, Peter Stoustrup	Excluded	Title/Abstract
128	Dental tourism: Examining tourist profiles, motivation and satisfaction	Mustaffa Jaapar, Ghazali Musa, Sedigheh Moghavvemi, Roslan Saub	Excluded	Title/Abstract

129	Repair versus replacement of defective direct dental restorations: A multinational cross-sectional study with meta-analysis	Ömer Hatipo ğlu, João Filipe Brochado Ma rtins, Mohmed Isaqali Karob ari, Nessrin T aha, Thiyeze n Abdullah Al dhelai, Daou d M. Ayyad, A hmed A. Madfa, Be njamin Marti n- Biedma, Rafa el Fernandez, Bakhyt A. Omarova, Lim Wen Yi , Suh a Alfirjani, A nna Lehman n , Surendar	Excluded	Title/Abstract

		Sugumaran , Xenos Petridis , Silvana Jukić Krmek , Dian Agustin Wahjuningrum , Azhar Iqbal x, Imran Zainal Abidin , Martha Gallegos Intriago …Fatma Pertek Hatipoğlu		
130	A phenomenological exploration of experience of Syrian dentists with online Course “Traumatic dental injuries”	Mayssoon Dashash, Rania Alkhadragy, Gillian M. Scanlan	Excluded	Title/Abstract
131	A surgeon’s perspective on the uncorrected skeletal deformity. Part I: Unintended consequences on facial structures and esthetic harmony	Saul M. Burk, Karina Charipova , Susan Orra, Patrick W. Harbour,	Excluded	Title/Abstract

		Mark D. Mishu, Stephen B. Baker		
132	Web-based information on oral dysplasia and precancer of the mouth – Quality and readability	Abdullah Alsoghier, Richeal Ni Riordain , Stefano Fedele, Stephen Porter	Excluded	Title/Abstract
133	Subject Index: 2017 Annual Subject Index	No author's name	Excluded	Title/Abstract
134	Never events in clinical orthodontic practice	Laurance Jerrold, Jaclyn Danoff-Rudick	Excluded	Title/Abstract
135	Patient perception of orthodontists with different stereotypes: development, psychometric properties, and application of an assessment instrument	Rafael Cunha Bittencourt, Sarah Braga Sayão de Paula, Líris Cristina Nepomuceno Pinto, Marcela Baraúna Mag	Excluded	Title/Abstract

		no, Lucas Alves Jural, Matheus Melo Pithon, Mônica Tirre de Souza Araújo		
136	Global survey to assess preferences for attending virtual orthodontic learning sessions	Mohammed Almuzian, Haris Khan, Samer Mheissen, Mark B. Wertheimer	Excluded	Title/Abstract
137	What you read is what you get: Are orthodontic randomized clinical trials correctly titled?	Georgina Kane, Martyn T. Cobourne, Nikolaos Pandis, Jadbinder Seehra	Excluded	Title/Abstract
138	Open Access: Concepts, findings, and recommendations for stakeholders in dentistry	Fang Hua, Cenyu Shen, Tanya Walsh, Anne-Marie Glenn	Excluded	Title/Abstract

		y, Helen Wor thington		
139	Patient Statistics: What You Really Need to Know	Ellen M. Grady	Excluded	Title/Abstract
140	16 - Adolescent patients	Lorne D. Koroluk	Excluded	Title/Abstract
141	“Ten years of war! You expect people to fear a ‘germ’?”: A qualitative study of initial perceptions and responses to the COVID-19 pandemic among displaced communities in opposition-controlled northwest Syria	Yazan Doued ari, Mervat A lhaffar, Muha mmed Al-Twaish, Hala Mkhallalati , Raheb Alwan y , Nafeesah Bte Mohamed Ibr ahim , Aysha th Zaseela, N our Horanieh , Aula Abbar a , Natasha H oward	Excluded	Title/Abstract

142	Evaluation of Dentists' Attitudes Towards Principles of Professional Ethics	Sayed Mohammad Razavi, Laleh Maleki, Amir Keshavarzian, Awat Feizi, Forooz Keshani, Sepehr Sobhani Kazemi	Excluded	Title/Abstract
143	Teledentistry Applied to Health and Education Outcomes: Evidence Gap Map	Júlia Nascimento da Silva Mulder, Marcelo Ramos Pinto, Isabelle Aníbal, Ana Paula Dornellas, Deise Garrido, Camila Huanca, Ana Estela Haddad, Carmen Verônica Mendes Abdala MPA, MLS	Excluded	Title/Abstract

144	Facial Transplantation	Krishna Vyas, Karim Bakri, Waleed Gibreel, Sebastian Cotofana, Hatem Amer, Samir Mardini	Excluded	Title/Abstract
145	Smile Analysis: Diagnosis and Treatment Planning	Ahmed Sabbah	Excluded	Title/Abstract
146	Extended reality technology for alleviating adult dental anxiety: A systematic review and meta-analysis of randomized controlled trials	Zeng Yang, Ping Liu, Yu Luo, Zhaowu Chai, Bing Yang	Excluded	Title/Abstract
147	Causation processes and innovation strategy in small- and medium-sized firms in emerging countries: The moderating role of founder-CEOs' human capital	Gordon Liu, Stephen X. Zhang, Wai Wai Ko, Asghar Afshar Jahanshahi, Yantai Chen, Joshin John	Excluded	Title/Abstract

148	Affect of Covid-19 on Restorative Dentistry Education in Türkiye	Sümeyye Tuncer Hancı , Lena Bal, Önder Hancı, Eda Güler, Nihan Gönülol	Excluded	Title/Abstract
149	Product advertisements in orthodontic journals: Are they evidence-based?	Othman Hameed, Nikolaos Pandis, Martyn T. Cobourne, Jadbinder Seehra	Excluded	Title/Abstract
150	Quality of Information Regarding Repair Restorations on Dentist Websites: Systematic Search and Analysis	Philipp Kanzow , Amelie Friederike Büttcher , Annette Wiega , Falk Schwendicke	Excluded	Title/Abstract
151	The use of ChatGPT and Google Gemini in responding to orthognathic surgery-related questions: A comparative study	Ahmed A. Abdel Aziz , Hams H. Abdelrahman , Mohamed G. Hassan	Excluded	Title/Abstract

152	Employment Opportunities, Latency, and Satisfaction among KAUFD Graduates	Arwa M. Farag, Hebah Al-Dehlawi, Nishan Hafiz, Wahiba Alsharif, Khalid Al-Johani, Ahoud Jazzar, Alaa F. Bukhari, Suad AlJohani, Ghassan Al-Turki	Excluded	Title/Abstract
153	Malocclusion traits as risk indicators for depression in young adults: A population-based cross-sectional study	Ahmed A. Alsulaiman	Excluded	Title/Abstract
154	Integrating artificial intelligence: A step forward in orthodontic education	Vinod Krishnan	Excluded	Title/Abstract
155	Publication trends and scientific profile of clinical trials on universal adhesives in dentistry: A metrics-based review	Aurélio de Oliveira Rocha, Lucas Menezes dos Anjos, Michael Willian Favoreto, Michely	Excluded	Title/Abstract

		Cristina Goebel, Bruno Henriques, Alessandra Reis, Alessandro D. Loguercio, Mariane Cardoso		
156	Perceived quality factors that discriminate parents of orthodontic patients according to their satisfaction	Gustavo Quiroga Souki , Juliana Meire Silva de Ávila , Luiz Rodrigo Cunha Moura , Bernardo Quiroga Souki	Excluded	Title/Abstract
157	Orthodontic pain: The use of non-pharmacological adjuncts and its effect on compliance	Padhraig S. Fleming, D. Al-Moghrabi, P. Fudalej, N. Pandis	Excluded	Title/Abstract
158	The impact of the COVID-19 pandemic on U.S. orthodontic practices in 2020	Hengameh Motevasel , Lana	Excluded	Title/Abstract

		R. Helms, George J. Eckert, Kelton T. Stewart, David A. Albright		
159	Reaching retirees	No authors available	Excluded	Title/Abstract
160	Performance of the ChatGPT-3.5, ChatGPT-4, and Google Gemini large language models in responding to dental implantology inquiries	Noha Taymour, Shaimaa M. Fouda, Hams H. Abdelrahaman, Mohamed G. Hassan	Excluded	Title/Abstract
161	Gap Analysis Need, effective demand of dental practice	Naveen Kumar Pera, Swapna Bettanapalya Venkatesh	Excluded	Title/Abstract
162	Stress and anxiety in orthodontic residents during the coronavirus disease 2019 pandemic	Edmund Khoo, Sophia G. Saeed , Hong-Yan Chiu, Vicky Quach,	Excluded	Title/Abstract

		Malvin Janal f, Kelton Ste wart		
163	Subject Index: Annual Index 2016	No authors available	Excluded	Title/Abstract
164	Motives for Surgical-Orthodontic Treatment and Effect of Treatment on Psychosocial Well-Being and Satisfaction: A Prospective Study of 118 Patients	Jesper Øland, John Jensen, Ask Elklit, Bi rte Melsen, Dr Odont	Excluded	Title/Abstract
165	Comparison of factors associated with the evidence-practice gap as perceived by Japanese and Brazilian dentists	Naoki Kakud ate, Yoko Yo koyama, Elai ne Pereira da Silva Tagliafe rro, Futoshi S umida, Yuki Matsumoto, Valeria V Gordan, Gr egg H Gilbert	Excluded	Title/Abstract

166	Current orthognathic surgery practices: A comprehensive survey from planning to discharge in Oral and Maxillofacial Surgery	Anne-Kathrin Bär, Andreas Pabst, Frederic Bouffleur, Daniel G.E. Thiem, Richard Werkmeister, Marco R. Kesting, Max Heiland, Bilal Al-Nawas, Philipp Becker	Excluded	Title/Abstract
167	Mobile health applications for children's oral health improvement: A systematic review	Niloofar Mohammadzadeh, Marsa Gholamzadeh, Sorour Zahednamazi, Seyed Mohammad Ayyoubzadeh	Excluded	Title/Abstract
168	Barriers in Implementing the Lean Startup Methodology in Indonesia – Case Study of B2B Startup	Michael Dwianto Nir	Excluded	Title/Abstract

		wan, Wawan Dhewanto		
169	Maternal and paternal anxiety levels through primary lip surgery	Hanife Nuray Yilmaz, Ece Abuhan	Excluded	Title/Abstract
170	Taxonomic discordance of immersive realities in dentistry: A systematic scoping review	Khaled Q. Al Hamad, Khalid N. Said, Marcus Engelschalk, Manal Matoug-Elwerfelli, Nidhi Gupta , Jelena Eric, Shaymaa A. Ali, Kamran Ali, Hanin Daas, Elham S. Abu Alhaija	Excluded	Title/Abstract
171	Should orthognathic surgery be performed in growing patients? A scoping review	Ricardo Grillo, Bruno Gomes, Tenyson Reis, Isabella	Excluded	Title/Abstract

		Romão Candido, Michael Miloro, Alexandre Meireles Borba		
172	Does age influence self-perception of the soft-tissue profile in children?	Vysnave Varatharaju, Marianne Caflisch, Cindy Soroken, Stavros Kiliaridis, Gregory S. Antonarakis	Excluded	Title/Abstract

173	Awareness of possible complications associated with direct composite restorations: A multinational survey among dentists from 13 countries with meta-analysis	Anna Lehmann, Kacper Nijakowski, Jakub Jankowski, David Donnermeyer, Paulo J. Palma, Milan Droba, João Filipe Brochado Martins, Fatma Pertek Hatipoğlu, Indira Tulegenova, Muhammad Qasim Javed, Hamad Mohammad Alharkan, Olga Bekjanova, Sylvia Wyzga, Moataz-Bellah Ahmed Mohamed Al	Excluded	Title/Abstract

		khawas, Rutendo Kudenga, Ömer Hatipoğlu, Anna Surdacka		
174	Index	No authors available	Excluded	Title/Abstract
175	Career Development: Multilevel Perspective	Yehuda Baruch	Excluded	Title/Abstract
176	Body Dysmorphic Disorder in Women	Katharine A. Phillips Leah C. Susser	Excluded	Title/Abstract
177	The long-term influence of orthodontic treatment on dental knowledge and behaviour: An Australian cohort study	Esma J. Doğramacı, Farhad B. Naini, David S. Brennan	Excluded	Title/Abstract

178	Starting a Cleft Team: A Primer	Randolph B. Capone M D, Sydney C. Butts MD, Lamont R. Jones	Excluded	Title/Abstract
179	Medical 4.0 technologies for healthcare: Features, capabilities, and applications	Abid Haleem , Mohd Javai d, Ravi Prata p Singh, Rajiv S uman	Excluded	Title/Abstract
180	Authors' response	Paula Cotrin, Karina Maria Salvatore Frei tas	Excluded	Title/Abstract
181	Care by video consultations: why or why not?	Jan Aidemar k	Excluded	Title/Abstract
182	Role of the Veterinary Technicians and Hygienists in Veterinary Dentistry and Oral Surgery	Mary L. Berg , Jeanette M. Eliason	Excluded	Title/Abstract
183	Handbooks or handcuffs?	panelLauranc e Jerrold	Excluded	Title/Abstract
184	I want to see everything	Laurance Jerr old	Excluded	Title/Abstract

185	Adult patient expectations and satisfaction: Can they be influenced by viewing the three-dimensional predicted outcome before fixed orthodontic treatment of dental crowding?	Abdalrahman Mohieddin Kusaibati, Kinda Sultan, Mohammad Y. Hajeer, Ahmad S. Burhan, Mohammad Khursheed Alam	DUPLICATE PUB 128	
186	Wednesday, 5 September 2018 - Free Communication Sessions 01–12 and Poster Sessions 01–15	FDI World Dental Federation	Excluded	Title/Abstract
187	Resting-State Functional Connectivity in a Community Sample of Children With a Range of Cognitive Disengagement Syndrome Symptoms	Stephen P. Becker, Adebayo Braimah, Jonathan A. Dudley, Leanne Tamm, Jeffery N. Epstein	Excluded	Title/Abstract
188	Chapter 31 - Practice Management	Julie Weir	Excluded	Title/Abstract
189	Where are we going?	Steven J. Lindauer	Excluded	Title/Abstract

190	Teledentistry for Improving Access To, and Quality of Oral Health Care: Overview of Systematic Reviews and Meta-Analyses	Pascaline Ke ngne Talla , Paul A llison, André Bussières DC ,, Anisha Rod rigues, Frédé ric Bergeron, Nicolas Gira udeau, Elha m Emami	Excluded	Title/Abstract
191	Tourism & animal suffering: Mapping the future	David A. Fennell, B astian Thoms en	Excluded	Title/Abstract
192	Effects of fixed vs removable orthodontic retainers on stability and periodontal health: 4-year follow-up of a randomized controlled trial	Dalya Al-Moghrabi, A ma Johal, Nia mh O'Rourk, Nikolaos Do nos, Nikolaos Pandis, Cecil ia Gonzales-Marin, Padhr aig S. Fleming	Excluded	Title/Abstract

193	A relationship well maintained	Vinod Krishn an	Excluded	Title/Abstract
194	ADA® 2014: America's Dental Meeting	No authors available	Excluded	Title/Abstract
195	Subject Index: Annual Index 2013	No authors available	Excluded	Title/Abstract
196	IS THERE A CORRELATION BETWEEN OBJECTIVE AND SUBJECTIVE METHODS TO ASSESS DENTAL ANXIETY? A SYSTEMATIC REVIEW AND META-ANALYSIS	CAMILA SILVA DE AMORIM , LARISSA SOARES LIMA DA SILVA M sC, GUIDO ARTEMIO M ARAÑÓN-VÁSQUEZ, MARCELA BARAÚNA MAGNO, A NDRÉA VAZ BRAGA PIN TOR, PEDRO PAULO PIRE S, LUCIANN E COPLE MAI	Excluded	Title/Abstract

		A, MATHEU S MELO PITH ON		
197	The staff’s perception of level of clinical competence of graduating dental students amid COVID-19: A qualitative research in one Saudi dental schoolLa percepción del personal sobre el nivel de competencia clínica de los estudiantes de odontología que se gradúan en medio de COVID-19: una investigación cualitativa en una escuela de odontología de Arabia Saudita	Rasha Salman A. Alafaleg, Reza Vahid Rouds ari, Riham To rk, Michaela Goodwin	Excluded	Title/Abstract
198	Malocclusion severity and smile features: Is there an association?	Hisham Moh ammed, Regi nald Kumar Jr, Hamza Be nnani, John P erry, Jamin B. Halberstad	Excluded	Title/Abstract

		t, Mauro Fare lla		
199	A Web-Based Survey Assessing the Attitudes of Health Care Professionals in Germany Toward the Use of Telemedicine in Pregnancy Monitoring: Cross-Sectional Study	Niklas Grassl MS, Juliane Nees, Kathari na Schramm, Julia Spratte, Christof Sohn , Timm Schot t, Sarah Schot t	Excluded	Title/Abstract
200	Subject Index	No authors available	Excluded	Title/Abstract
201	Effectiveness of an oral health promotion training program among school nurses in India	Bharathi M. Purohit, S neha Malhotr a, Manali De b Burma, Upen dra Singh Bhada uria, Deepali Agarwal, Sasi dharan Shiva kumar, Ritu	Excluded	Title/Abstract

		Duggal, Harsh Priya		
202	Curriculum content for Environmental Sustainability in Dentistry	Jonathan Dixon, James Field, Emma Gibson, Nicolas Martin	Excluded	Title/Abstract
203	An exploratory study of breakthrough innovations in digital businesses: The case of the Perfect Corporation	Feng-Shang Wu, Chia-Chang Tsai, Chien-Hsin Wu, Ting-Hsuan Lo	Excluded	Title/Abstract
204	The reallocation effects of COVID-19: Evidence from venture capital investments around the world	Andrea Bellucci, Alexander Borisov, Gianluca Gucciardi, Alberto Zazzaro	Excluded	Title/Abstract
205	Employee engagement: Communicating clear expectations	Gay Lowry	Excluded	Title/Abstract
206	Flipped Classroom Experiences in Clinical Dentistry – A Strategic Mini-Review	Abdullah Aljabr	Excluded	Title/Abstract

207	ChatGPT Conversations on Oral Cancer: Unveiling ChatGPT's Potential and Pitfalls	Nikunj Maniyar, Gargi S. Sarode, Sachin C. Sarode, Shruti Thakkar	Excluded	Title/Abstract
208	“I just want to be treated like a normal person”: Oral health care experiences of transgender adolescents and young adults	David W. Macdonald, Daniel H. Grossoehme DMin, Amanda Mazzola BS, Teresa Pestian, Scott B. Schwartz	Excluded	Title/Abstract
209	Sociobehavioural Factors Associated With Child Oral Health During COVID-19	Ravi Kumar Gudipaneni, Mohammed Farhan O. Alruwaili, Kiran Kumar Ganji, Mohmed Isaqali Karobari, Sachin K	Excluded	Title/Abstract

		ulkarni, Kira n Kumar Metta , Ali A. Assiry, Ni cholas Israels son, Omar A Bawazir		
210	ADA Meeting: ADA 2015—America's Dental Meeting	No authors available	Excluded	Title/Abstract
211	Orthodontic temporary anchorage devices: A qualitative evaluation of Internet information available to the general public	Maurice J. Meade, Cra ig W. Dreyer	Excluded	Title/Abstract
212	Adoption of dental innovations: The case of a standardized dental diagnostic terminology	Rachel B. Ramoni, Ji ni Etolue, MPH, Oluwa bunmi Toked e, Lyle McCle llan DDS, Kri sten Simmon s, Alfa Yansa ne, Joel M. White, M uhammad F. Walji, Elsb	Excluded	Title/Abstract

		eth Kalenderi an		
213	Effectiveness of a New App in Improving Oral Hygiene in Orthodontic Patients: A Pilot Study	Raquel Lopes dos Santos, Manu ela da Silva Spinola, Ellen Carval ho, Denis Clay Lopes dos Santos, Naile Dame-Teixeira, Deb ora Heller	Excluded	Title/Abstract
214	The validity of using profile predictions for class III patients planned for bimaxillary orthognathic surgery	Sarah L Franks, An ant Bakshi, B alvinder S Khambay	Excluded	Title/Abstract
215	Friday, 7 September 2018 - Free Communication Sessions 28–42 and Poster Sessions 31–45	FDI World Dental Federation	Excluded	Title/Abstract

216	Developing a Sustainable Program for Volunteer Surgical Care in Low-Income and Middle-Income Countries	Vennila Padmanaban MD, David Hoffman DDS, Shahid R. Aziz DMD, MD, FRCS(Ed), Ziad C. Sifri	Excluded	Title/Abstract
217	The psychological impact of orthognathic surgery: A 9-month follow-up	H.Asuman Kiyak, Roger A. West, Thomas Hohl, R. William McNeill	Excluded	Title/Abstract
218	7 - Psychosocial Considerations in the Evaluation and Treatment of Dentofacial Deformities	Jeffrey C. Posnick	Excluded	Title/Abstract
219	Online Patient Education Materials for Orthognathic Surgery Fail to Meet Readability and Quality Standards	Kevin C. Lee, Elizabeth T. Berg BA, Hossein E. Jazayeri BS, Sung-Kiang Chuang, Sidney B. Eisig	Excluded	Title/Abstract

220	A qualitative study of orthodontic patients' experiences in quarantine during the COVID-19 pandemic outbreak	Khaled Wafaie, Hisham Mohammed, Abdelrahman M.A. Mohamed, Jinshu Zhou, Ben Daniel, Qiao Yiqiang	Excluded	Title/Abstract
221	The Second-Opinion Consultation	No authors available	Excluded	Title/Abstract
222	Knowledge, perception, attitude, practice and awareness of dentists and dental students about molar-incisor hypomineralization: A systematic review	Lilibeth-Stephania Escoto-Vasquez, Mario Alberto Alarcón-Sánchez, Julieta Sarai Becerra-Ruiz, Ruth Rodríguez-Montaño, Sarah Monserrat Lomelí-	Excluded	Title/Abstract

		Martínez, Diana Elizabeth Aguirre-Cortés, Artak Heboyan		
223	Protocol for teen inflammation glutamate emotion research (TIGER): Toward predictors of treatment response and clinical course in depressed adolescents	Saché M. Coury, Vanessa López, Zia Bajwa, Jordan M. Garcia, Giana I. Teresi, Kate R. Kuhlman, Yan Li, Steve Cole, DaviJ. Miklowitz, Ioannis Pappas, Tiffany C. Ho	Excluded	Title/Abstract
224	Application of artificial intelligence in modern medicine	Nuo Xu, Dawei Yang, Kinji Arikawa, Chunxue Bai	Excluded	Title/Abstract

225	RETRACTED: Application of artificial intelligence (AI) to control COVID-19 pandemic: Current status and future prospects	Sumel Ashique, Neeraj Mishra, Sourav Mohanto, Ashish Garg, Farzad Taghizadeh-Hesary, B.H. Jaswanth Gowda, Dinesh Kumar Chellappan	Excluded	Title/Abstract
226	Doctors' Preview Program	No authors available	Excluded	Title/Abstract
227	Correlates of objective patient compliance with removable appliance wear	Dipl. Psych. Axel Bartsch Emil Witt, Habil. Gerd Sahm, Stefan Schneider	Excluded	Title/Abstract
228	Exceptional circumstances	Laurance Jerrold	Excluded	Title/Abstract
229	So long, farewell, auf wiederschein, goodbye	Laurance Jerrold	Excluded	Title/Abstract
230	What do the teeth say?	Peter C. Kesling, Ri	Excluded	Title/Abstract

		chard C. Parkhouse		
231	Chapter 1 - Current advancements of machine learning in healthcare	Sameer Desh mukh, Nikhil Kulkarni, Ay sswarya Man oharan, Benja min Vinarski, Apurva Ram anujam	Excluded	Title/Abstract
232	Introduction	Maxime Deri an	Excluded	Title/Abstract
233	Factors of life satisfaction and happiness among dentists: A cross sectional study	Feridun Aba y , S. Kutalmış Buy uk , Recep Tu rken	Excluded	Title/Abstract
234	8 - Photographic advances in facial imaging	Linping Zhao , Yu-Hui Huang	Excluded	Title/Abstract
235	Associate Registry	No authors available	Excluded	Title/Abstract
236	Table of Contents	No authors available	Excluded	Title/Abstract
237	Knowledge-intensive consumer services. Understanding KICS in the innovative global health-care sector	Cátia Miriam Costa	Excluded	Title/Abstract

		, Sandro Men donça		
238	Development of a core outcome set for use in routine orthodontic clinical trials	Aliki Tsichla ki, Kevin O'B rien, Philip E. Benson, Zo e Marshman, Ama Johal, Fi orella B. Colonio-Salazar, Nico la L. Harman, P adhraig S. Fleming	Excluded	Title/Abstract
239	Dentistry website analysis: An overview of the content of formulated questions and answers	Peivand Bast ani, Fatemeh Niknam, Ma hboobeh Rez azadeh, Gia mpiero Rossi - Fedele, Sisira Edirippulige, Mahnaz Sam adbeik	Excluded	Title/Abstract

240	Online Only Abstracts	No authors available	Excluded	Title/Abstract
241	Optimal gingival display on smiling based on different facial vertical patterns in Iranians	Mohammad Moslem Imani, Ehsan Mohamadi Nezhad, Amin Golshah, Roya Safari-Faramani	Excluded	Title/Abstract
242	Patient centred outcomes research: The new norm in health care system	Vinod Krishnan	Excluded	Title/Abstract
243	Public view of orthodontists	No authors available	Excluded	Title/Abstract
244	Foster parents between voluntarism and professionalisation: Unpacking the backpack	Lieselot De Wilde, Jochen Devlieghere, Michel Vandenbroeck, Bruno Vanobbergen	Excluded	Title/Abstract
245	Associate Registry	No authors available	Excluded	Title/Abstract

246	Annual review of selected scientific literature: A report of the Committee on Scientific Investigation of the American Academy of Restorative Dentistry	David R. Cagna , Terence E. Donovan , James R. McKee, Frederick Eichmiller, James E. Metz, Riccardo Marzola , Kevin G. Murphy g, Matthias Troeltzsch	Excluded	Title/Abstract
247	Work Hard … Get Lucky	LeeAnn Peniche	Excluded	Title/Abstract
248	Generation gap	No authors available	Excluded	Title/Abstract

249	Underrepresentation of female speakers at online and in-person dentistry conferences	María José Mendoza-Schettino, Daniel Arturo Navarrete-Limón, Josué Roberto Bermeo-Escalona, Edith Lilia Galindo-Reyes, José Luis Suárez-Franco, Mariné Ortiz-Magdaleno, Aída de los Angeles Cerda-Cristerna, Bernardino Isaac Cerda-Cristerna	Excluded	Title/Abstract

250	How well are dental qualitative studies involving interviews and focus groups reported?	Dalya Al-Moghrabi, Al iki Tsichlaki, Saleh Alkadi, Padhraig S. Fleming	Excluded	Title/Abstract
251	Parental Acceptance of Behavior Management Techniques for Pediatric Dental Visits in Qassim, Saudi Arabia: A Cross-sectional Study	Thiyezen Abdullah Al dhelai, Nimr an Shaman Almodhaibri, Zeyad Alsug hier, Saleh Abdullah Al harbi	Excluded	Title/Abstract
252	Family-centred care during midface advancement with a rigid external device: What do families need?	H. Bredero-Boelhouwer, K.F.M. Jooste n, M. van Veen-van der Hoek, I.M.J. Mathijssen	Excluded	Title/Abstract
253	ADA Meeting: ADA 2017–America's Dental Meeting	No authors available	Excluded	Title/Abstract
254	Large language models in periodontology: Assessing their performance in clinically relevant questions	Georgios S. Chatzopou los, Vasiliki	Excluded	Title/Abstract

		P. Koidou, Lazaros Tsalikis, Eleftherios G. Kaklamanos		
255	22 - Health Education and Health Literacy in Dental Public Health	Richie Kohli, Eli Schwarz	Excluded	Title/Abstract
256	Cephalometric landmark annotation using transfer learning: Detectron2 and YOLOv8 baselines on a diverse cephalometric image dataset	S. Rashmi, S. Srinath, Seema Deshmukh, S. Prashanth, Karthikeya Patil	Excluded	Title/Abstract
257	Saturday, September 13, 2014 - FREE COMMUNICATIONS SESSIONS 09-12	FDI World Dental Federation	Excluded	Title/Abstract
258	Thursday, 5 September 2019 - WDC 2019 Abstract Book FREE COMMUNICATION SESSIONS 01–12 and POSTER SESSIONS 01–06	FDI World Dental Federation	Excluded	Title/Abstract
259	Artificial Intelligence or Augmented Intelligence? Impact on our lives, rights and ethics	Fabio De Felice, Antonella Petrillo, Cristina De Luca, Ilaria Baffo	Excluded	Title/Abstract
260	Congenital Craniofacial and Maxillofacial Malformations	N.J.K. Rumsey	Excluded	Title/Abstract

261	The Development of Scientific Activity in Russian Universities	Marina Vasiljeva, Vadim Ponkratov, Tatyana Volkova, Saida Khairova, Natalya Nikitina, Olesya Dudnik, Maria Alimova, Nikolay Kuznetsov, Izabella Elyakova	Excluded	Title/Abstract
262	Beware of the herd	Peter M. Greco (Associate Editor for Ethics in Orthodontics)	Excluded	Title/Abstract
263	Training Satisfaction Versus Dissatisfaction Among Chief Residents in Oral and Maxillofacial Surgery—A Pilot Survey	Shahid R., Vincent B. Ziccardi , Sung-Kiang Chuang	Excluded	Title/Abstract
264	11 - Maintenance phase of care	Jennifer L. Brame, Ly	Excluded	Title/Abstract

		nne Carol Hunt, S amuel P. Nesbit		
265	P098AWDC 2015 – Abstract book - 23 September 2015 - FREE COMMUNICATION SESSIONS 21–40 and POSTER SESSIONS 17–32	FDI World Dental Federation	Excluded	Title/Abstract
266	The impact of interpersonal support on patient satisfaction with orthognathic surgery	H.Asuman K iyak	Excluded	Title/Abstract
267	Automated landmark identification for diagnosis of the deformity using a cascade convolutional neural network (FlatNet) on weight-bearing lateral radiographs of the foot	Seung Min Ryu, Kee won Shin, So o Wung Shin, S un Ho Lee, Su Min Seo, Seu ng- uk Cheon, Se ung- Ah Ryu, Jun- Sik Kim, Sun ghwan Ji, Na mkug Kim	Excluded	Title/Abstract
268	Sunday, September 14, 2014 - FREE COMMUNICATIONS SESSIONS 13-16	FDI World Dental Federation	Excluded	Title/Abstract

269	The decade ahead: Finding a better way	Arthur A. Dugoni	Excluded	Title/Abstract
270	Hanging the digital shingle: Dental ethics and search engine optimization	Eric S. Swirsky JD , MA, Chinami Michaels, Sara Stuefen, Michael Halasz	Excluded	Title/Abstract
271	Wednesday, 30 August 2017 - Free Communication Sessions 25–48 and Poster Sessions 25–47	FDI World Dental Federation	Excluded	Title/Abstract
272	Drivers, opportunities and best practice for sustainability in dentistry: A scoping review	Nicolas Martin, Madison Sheppard, GaneshParth Gorasia, Pranav Arora, Matthew Cooper, Steven Mulligan	Excluded	Title/Abstract
273	11 - Journals ranking and impact factors: how the performance of journals is measured	Iain D. Craig, Liz Ferguson, Adam T. Finch	Excluded	Title/Abstract

274	Brazilian adolescents' perception of the orthodontic appliance: A qualitative study	Anderson Ba rbosa de Almeida, Isa bel Cristina Gonçalves Le ite, Girlene A lves da Silva	Excluded	Title/Abstract
275	Beauty and the eye of the beholder: social consequences and personal adjustments for facial patients	Erika M. Arndt , Fe licia Travis), Arlette Lefeb vre , Ann Nie c , Ian R. Munro	Excluded	Title/Abstract
276	Temporomandibular disorders and chronic pain: Disease or illness?	Samuel F. Dworkin, Donna L. Massoth	Excluded	Title/Abstract
277	Subject Index: VOLUME 142, 2011	No authors available	Excluded	Title/Abstract
278	ADA Meeting: ADA 2019–America's Dental Meeting	No authors available	Excluded	Title/Abstract
279	Treatment outcomes and patient-reported quality of life after orthognathic surgery with computer-assisted 2- or 3-dimensional planning: A randomized double-blind active-controlled clinical trial	Martin Bengt sson, Gert W all, Pernilla L arsson, Jonas P. Becktor, La	Excluded	Title/Abstract

		rs Rasmusso n		
280	Maxillary arch width and buccal corridor changes with orthodontic treatment. Part 1: Differences between premolar extraction and nonextraction treatment outcomes	Anna H. Meyer, Mi chael G. Woods, D avid J. Manton	Excluded	Title/Abstract
281	AWDC 2016 – Abstract book - Friday, 9 September 2016 - FREE COMMUNICATION SESSIONS 36–53 POSTER SESSIONS 40–58	FDI World Dental Federation	Excluded	Title/Abstract
282	Associate Registry	No authors available	Excluded	Title/Abstract
283	Tuesday, 29 August 2017 – Free Communication Sessions 01–24 and Poster Sessions 01–24	FDI World Dental Federation	Excluded	Title/Abstract
284	Index	No authors available	Excluded	Title/Abstract
285	Current challenges and opportunities in global sleep medicine	No authors available	Excluded	Title/Abstract
286	Abstract Book	No authors available	Excluded	Title/Abstract
287	Saturday Abstracts	No authors available	Excluded	Title/Abstract
288	The Glossary of Prosthodontic Terms 2023: Tenth Edition	No authors available	Excluded	Title/Abstract

289	The 96th Annual Meeting of The Physiological Society of Japan	No authors available	Excluded	Title/Abstract
**4. Scopus**				
**Search date**	24/8/2025			
**Results**	250			
**Search Query**	(Orthodontic* OR "orthodontic treatment" OR braces OR "clear aligners" OR Invisalign) AND ("Social Media" OR "social network*" OR Facebook OR Instagram OR TikTok OR YouTube OR Twitter OR X OR Snapchat) AND (willing* OR motivat* OR intent* OR perception OR attitude* OR "decision making" OR preference* OR "patient acceptance")			
**Nr.**	**Title**	**Eligibility**	**Reason for exclusion**	
1	Kenis VM, Baindurashvili AG, Shpulev PS, Sapogovskiy AV, Melchenko EV, Rustamov GN, et al. Usefulness and accessibility of information on the treatment of children with congenital clubfoot: results of a survey of parents. Pediatric Traumatology, Orthopaedics and Reconstructive Surgery. 2024;12(3):327–34.	Excluded	Title/Abstract	
2	Bhamrah G, Ahmad S, NiMhurchadha S. Internet discussion forums, an information and support resource for orthognathic patients. American Journal of Orthodontics and Dentofacial Orthopedics. 2015;147(1):89–96.	DUPLICATE PUBMED #37		
3	Gutierrez-Marín N, Miranda-Garro I, López-Soto A. Instagram as a Pedagogical Tool in Pediatric Dentistry and Ortodontic Courses. Odovtos - International Journal of Dental Sciences. 2024;26(2):71–81.	Excluded	Title/Abstract	

4	Sampson A, Figueiredo DSF, Jeremiah HG, Oliveira DD, Freitas LRP, Chahoud M, et al. The effect of social media on patient acceptance of temporary anchorage devices: A cross-cultural study. Angle Orthodontist. 2021;91(3):363–70	DUPLICATE PUBMED #190		
5	Henzell M, Knight A, Antoun JS, Farella M. Social media use by orthodontie patients. New Zealand Dental Journal. 2013;109(4):130–3.	DUPLICATE PUBMED #96		
6	Sebbag R, Gebeile-Chauty S. Google Reviews: a descriptive study of 700 orthodontic practices. L'Orthodontie Française. 2021;92(1):167–76.	Excluded	Title/Abstract	
7	Alslakhi MHR, Oz U, Sin Ç. RETRACTED ARTICLE: The powerful effects of social media platforms on orthodontic patient knowledge's improving, attitude management and it is influence on financial income of the orthodontic clinic(Applied Nanoscience, (2022), 13, (2)). Applied Nanoscience (Switzerland). 2023;13(3):1803.	Excluded	Title/Abstract	
8	Grosvenor D, Kendall J, Sanders A, Chang-Tien CT. Kongress: A search and data mining application for U.S. congressional voting and Twitter data. In 2013. p. 550–3. Available from: https://www.scopus.com/inward/record.uri?eid=2-s2.0-84893456900&doi=10.1145%2F2525314.2525328&partnerID=40&md5=5ce35671bd5dd10403c75e5cb4781605	Excluded	Title/Abstract	
9	Bilal R. Preferences and attitudes of orthodontic patients on use of social media in Saudi Arabia. Medical Forum Monthly. 2021;32(8):37–41.	Excluded	Title/Abstract	
10	Soares LP, Scalioni FAR, Fiche GEC, Procopio SW, Carrada CF, Fernandes RB, et al. Knowledge of Brazilian Dentists about Dentoalveolar Trauma Care and their Experiences during the COVID-19 Pandemic. Pesquisa Brasileira em Odontopediatria e Clinica Integrada [Internet]. 2024;24. Available from: https://www.scopus.com/inward/record.uri?eid=2-s2.0-	Excluded	Title/Abstract	Full text, Not malignancy - Not only cancer

	85180184385&doi=10.1590%2Fpboci.2024.013&partnerID=40&md5=0b061 55700560c64c05af25e9d2d34ff			
11	Coêlho da Silveira TGS, Caracas HCPM. Perception of the relationship between TMD and orthodontic treatment among orthodontists. Dental Press Journal of Orthodontics. 2015;20(1):45–51.	DUPLICATE PUBMED #49		
12	Karkun R, Batra P, Singh AK. Influence of social media and corrected smile photographs in patients with malocclusion. American Journal of Orthodontics and Dentofacial Orthopedics. 2023;164(5):712–27.	DUPLICATE PUBMED #115		
14	Damasaru MS, Păcurar M, Mariș M, Dămășaru E, Mariş M, Tilinca CM. Implications of type 1 diabetes mellitus in the etiology and clinic of dento-maxillary anomalies - questionnaire-based evaluation of the dentists' opinion. Medicine and Pharmacy Reports. 2025;98(1):135–43.	Excluded	Title/Abstract	
14	Romero A, Ramírez-Rozzi FV, De-Juan-Herrero J, PÃ©rez-PÃ©rez A. Diet-related buccal dental microwear patterns in central african pygmy foragers and bantu-speaking farmer and pastoralist populations. PLOS ONE [Internet]. 2013;8(12).	Excluded	Title/Abstract	
15	Meira TM, Prestes J, Gasparello GG, Antelo OM, Pithon MM, Tanaka OM. The effects of images posted to social media by orthodontists on public perception of professional credibility and willingness to become a client. Progress in Orthodontics [Internet]. 2021;22(1). Available from: https://www.scopus.com/inward/record.uri?eid=2-s2.0-85102171960&doi=10.1186%2Fs40510-021-00353-9&partnerID=40&md5=79fa5eb0902f0193ac777bb254807543	DUPLICATE PUBMED #148		

16	Graf I, Kruse T, Braumann B, Hoefer K, Ehlebracht D. Looking good but tweeting bad? The social perception of orthodontic-related posts on Twitter and Instagram. Head and Face Medicine [Internet]. 2022;18(1). Available from: https://www.scopus.com/inward/record.uri?eid=2-s2.0-85124776200&doi=10.1186%2Fs13005-021-00302-1&partnerID=40&md5=d69e5cb80264a99fa7048c9a74a8e360	DUPLICATE PUBMED #84		
17	Doğrugören R, Demir GB, Topsakal KG, Duran GS, Görgülü S. Analysis of the patient's experiences on the related instagram posts about the orthodontic treatment. APOS Trends in Orthodontics. 2023;13(3):161–7.	Excluded	Title/Abstract	
18	Castilhos JS, Gasparello GG, Mota-Júnior SL, Hartmann GC, Miyagusuku LFI, Pithon MM, et al. Accessories in clear aligner therapy: Laypeople's expectations for comfort and satisfaction. Journal of Dental Research, Dental Clinics, Dental Prospects. 2024;18(2):102–9.	DUPLICATE PUBMED #47		
19	Almotairy N. Public perception of Invisalign® clear aligner treatment: A cross-sectional survey-based study. APOS Trends in Orthodontics. 2023;13(1):38–45.	Excluded	Title/Abstract	
20	Al-Gunaid TH, Aljohani AA, Ibrahim AM. Determining the impact of orthodontic patients' characteristics on their usage and preferences of social media. Journal of Taibah University Medical Sciences. 2021;16(1):16–21.	DUPLICATE PUBMED #9		
21	Graf I, Gerwing H, Hoefer K, Ehlebracht D, Christ H, Braumann B. Social media and orthodontics: A mixed-methods analysis of orthodontic-related posts on Twitter and Instagram. American Journal of Orthodontics and Dentofacial Orthopedics. 2020;158(2):221–8.	DUPLICATE PUBMED #83		
22	Grassia V, D'Apuzzo F, Alansari RA, Jamilian A, Sayahpour B, Adel SM, et al. Instagram and clear aligner therapy: A content analysis of patient perspectives. Seminars in Orthodontics [Internet]. 2024; Available from:	DUPLICATE SCIENCE DIRECT #2		

	https://www.scopus.com/inward/record.uri?eid=2-s2.0-85194933518&doi=10.1053%2Fj.sodo.2024.05.009&partnerID=40&md5=4396d1c2681fece6f9f36863d29968b4			
23	Adanan A, Kamarudin Y, Nor NAM, Bahar AD, Abd Rahman ANA. Perceptions towards orthodontic marketing through social media among young adults seeking orthodontic treatment: a qualitative study. Australasian Orthodontic Journal. 2024;40(1):73–84.	Included		
24	Alalola B, Alghanim L, Almoneef S, Aba Numay S, Aldghim A, Abu Wathlan J. The Impact of Orthodontists' Image-Based Social Media Posts on the Public's Willingness to Seek Treatment in the Central Region of Saudi Arabia. Patient Preference and Adherence. 2025;19:1255–61.	DUPLICATE PUBMED #14		
25	Mani S, Khurdal AS, Manerikar R, Arimbur A. Evaluation of augmented reality and social media on patient motivation to undergo fixed orthodontic treatment. APOS Trends in Orthodontics. 2024;14(2):124–9.	Excluded	Full text, does not refer to social media influence	
26	Shahrul AI, Pauzzi ER, Abas NDA, Yahya NA, Khan KAM, Marizan Nor MM. To assess the knowledge, awareness, and perception of non-dentists offering orthodontic treatment among the Malaysian young adult population. BMC Public Health [Internet]. 2024;24(1). Available from: https://www.scopus.com/inward/record.uri?eid=2-s2.0-85209727133&doi=10.1186%2Fs12889-024-20460-6&partnerID=40&md5=d3e7ece8f1c2b54a03756444d3dd93e2	DUPLICATE PUBMED #199		
27	Guo J, Yan X, Li S, van der Walt J, Guan G, Mei L. Quantitative and qualitative analyses of orthodontic-related videos on YouTube. Angle Orthodontist. 2020;90(3):411–8.	DUPLICATE PUBMED #89		
28	Allanqawi T, Alkadhimi A, Fleming PS. Postgraduate orthodontic education: An international perspective on content and satisfaction	DUPLICATE PUBMED #20		

	levels. Journal of the World Federation of Orthodontists. 2023;12(6):239–44.			
29	Siddiqui N, Chia M, Sharif MO. Social media and orthodontics: Are our patients scrolling? Journal of Orthodontics. 2022;49(2):179–84.	DUPLICATE PUBMED #204		
30	Wall A, Hillyard N, Ryan FS, Cunningham SJ. Adult orthodontic patients: What is important to them? Journal of Orthodontics. 2024;51(4):366–74.	DUPLICATE PUBMED #226		
31	Henzell MR, Knight AM, Morgaine KC, Antoun JS, Farella M. A qualitative analysis of orthodontic-related posts on Twitter. Angle Orthodontist. 2014;84(2):203–7.	DUPLICATE PUBMED #174		
32	Prithiviraj D, Siddiqui NR, Smyth RSD, Hodges SJ, Sharif MO. The awareness and usage of orthodontic apps and social media by orthodontists in the UK: A questionnaire-based study. Journal of Orthodontics. 2023;50(1):9–17.	DUPLICATE PUBMED #172		
33	Samman M, Bahanan L. The Perception and Usage of Fake Braces: Twitter Content Analysis. Open Dentistry Journal [Internet]. 2023;17(1). Available from: https://www.scopus.com/inward/record.uri?eid=2-s2.0-85164124869&doi=10.2174%2F18742106-v17-2304040-2022-137&partnerID=40&md5=93601a8f0567367040aac9d08a50943e	Excluded	Title/Abstract	
34	Guo F, Tang B, Qin D, Zhao T, Su YX, McGrath C, et al. The Impact of the COVID-19 Epidemic on Orthodontic Patients in China: An Analysis of Posts on Weibo. Frontiers in Medicine [Internet]. 2020;7. Available from: https://www.scopus.com/inward/record.uri?eid=2-s2.0-85098088498&doi=10.3389%2Ffmed.2020.577468&partnerID=40&md5=e3c2c33e2fa4808b7e51e89bff9160fd	DUPLICATE PUBMED #88		

35	Aflatooni JO, Loving R, Holderread BM, Liberman SR, Harris JD. #Scoliosis: an analysis of patient perception of scoliosis on TikTok. Baylor University Medical Center Proceedings. 2023;36(6):671–4.	DUPLICATE PUBMED #6		
36	Livas C, Delli K, Pandis N. “My Invisalign experience”: content, metrics and comment sentiment analysis of the most popular patient testimonials on YouTube. Progress in Orthodontics [Internet]. 2018;19(1). Available from: https://www.scopus.com/inward/record.uri?eid=2-s2.0-85040954272&doi=10.1186%2Fs40510-017-0201-1&partnerID=40&md5=eebe8da0a9b152fdf53a3585f59d4d25	Excluded	Title/Abstract	
37	Longstaff S, Davies K, Benson P. Exploring 10–15-year-old patients' perspectives of fixed orthodontic treatment. Journal of Orthodontics. 2021;48(2):110–7.	DUPLICATE PUBMED #135		
38	Adobes Martin M, Pérez Márquez A, Meuli S, Aguilera AC, Dioguardi M, Aiuto R, et al. User Experience, Satisfaction, and Complications of Direct-to-Consumer Orthodontics in Spain: A Cross-Sectional Study. Journal of Clinical Medicine [Internet]. 2025;14(7). Available from: https://www.scopus.com/inward/record.uri?eid=2-s2.0-105002396792&doi=10.3390%2Fjcm14072382&partnerID=40&md5=13be04d6a921650c6f7a452ea3f8ab20	DUPLICATE PUBMED #5		
39	Hussain SR, Jiang SS, Bosio JA. Generational perspectives of orthodontists in the U.S. and Canada: A survey study. American Journal of Orthodontics and Dentofacial Orthopedics. 2022;162(6):824–38	DUPLICATE PUBMED #100		
40	Sharif MO, Siddiqui NR, Hodges SJ. Patient awareness of orthodontic mobile phone apps. Journal of Orthodontics. 2019;46(1):51–5.	DUPLICATE PUBMED #200		

41	Jasim ES, Alnuaimy NSM, Abid M, Dziedzic A. Orthodontic practice marketing: The orthodontist and laypeople's perspective. Journal of Orthodontic Science [Internet]. 2024;13(1). Available from: https://www.scopus.com/inward/record.uri?eid=2-s2.0-85215381920&doi=10.4103%2Fjos.jos_37_24&partnerID=40&md5=a188f59d0566c83fd1dcbd92b904a418	DUPLICATE PUBMED #105		
42	Gasparello GG, Mota-Júnior SL, Hartmann GC, Berlesi AH, Acciaris F, Berretta LM, et al. Orthodontics social media, perceptions of science- and non-science-based posts among orthodontists, dentists, students and laypeople. PLOS ONE [Internet]. 2023;18(9 September). Available from: https://www.scopus.com/inward/record.uri?eid=2-s2.0-85173537733&doi=10.1371%2Fjournal.pone.0286927&partnerID=40&md5=6eb1c6ed1368468bee7df8d1ebe67153	DUPLICATE PUBMED #76		
43	Vishnupriya TR, Misra V, Yadav A, Attri S, Joshi D, Sharma P. Do-it-yourself aligners – safe, cheaper option or hazardous gamble? Journal of Contemporary Orthodontics. 2023;7(1):42–6.	Excluded	Title/Abstract	
44	Scribante A, Gallo S, Bertino K, Meles S, Gandini P, Sfondrini MF. The effect of chairside verbal instructions matched with instagram social media on oral hygiene of young orthodontic patients: A randomized clinical trial. Applied Sciences (Switzerland). 2021;11(2):1–14.	Excluded	Title/Abstract	
45	Ocak I, Aksu M. Effects of curing lights on polymerization shrinkage of composite attachments in clear aligner treatment: A microcomputed tomography study. American Journal of Orthodontics and Dentofacial Orthopedics. 2025;167(1):63–72.	DUPLICATE PUBMED #164		

46	Alharbi R, Taju W. Factors Influencing the Decision Process within Seeking Orthodontic Care among the Saudi Population: A Cross-sectional Survey. Open Dentistry Journal [Internet]. 2024;18.	DUPLICATE SCIENCE DIRECT #46		
47	Chen Q, Zhao Y, Liu Y, Sun Y, Yang C, Li P, et al. MSLPNet: multi-scale location perception network for dental panoramic X-ray image segmentation. Neural Computing and Applications. 2021;33(16):10277–91.	Excluded	Title/Abstract	
48	Schlichte MJ, Karimkhani C, Jones T, Trikha R, Dellavalle RP. Patient use of social media to evaluate cosmetic treatments and procedures. Dermatology Online Journal [Internet]. 2015;21(4). Available from: https://www.scopus.com/inward/record.uri?eid=2-s2.0-84927716032&partnerID=40&md5=5528ced774233ba266d1c4e553050531	Excluded	Title/Abstract	
49	Almotairy N. Public perception of the advertised claims of Damon® appliance system in Saudi Arabia: a cross-sectional survey-based study. BMC Oral Health [Internet]. 2025;25(1). Available from: https://www.scopus.com/inward/record.uri?eid=2-s2.0-105003444380&doi=10.1186%2Fs12903-025-06021-2&partnerID=40&md5=e75b7bb822b86be78be249677884582c	DUPLICATE PUBMED #21		
50	Cox T, Park JH. Facebook marketing in contemporary orthodontic practice: A consumer report. Journal of the World Federation of Orthodontists. 2014;3(2):e43–7.	DUPLICATE SCIENCE DIRECT #24		
51	Ahmed HMA, Obaid DH, Kadhum HI, Nahidh M, Russo D, Herford AS, et al. Awareness of orthodontic patients towards smartphone orthodontic apps. Minerva Dental and Oral Science. 2024;73(3):134–41.	DUPLICATE PUBMED #77		

52	Al-Silwadi FM, Gill DS, Petrie A, Cunningham SJ. Effect of social media in improving knowledge among patients having fixed appliance orthodontic treatment: A single-center randomized controlled trial. American Journal of Orthodontics and Dentofacial Orthopedics. 2015;148(2):231–7.	DUPLICATE PUBMED #13		
53	Nelson KL, Shroff B, Best AM, Lindauer SJ. Orthodontic marketing through social media networks: The patient and practitioner's perspective. Angle Orthodontist. 2015;85(6):1035–41.	DUPLICATE PUBMED #160		
54	Yavan MA, Eğlenen MN. 20-Year Change in the Perception of Orthodontic Treatment: A Cross-Sectional Study. Turkish Journal of Orthodontics. 2022;35(4):276–83.	Excluded	Title/Abstract	
55	Kamarozaman DMS, Kamarudin Y, Tengku Hamzah TNN, Nor NA. Self-perceived orthodontic need, informationseeking behaviour and knowledge on orthodontic treatment among young malaysian adults. Journal of Health and Translational Medicine. 2020;23(2):8–15.	Excluded	Title/Abstract	
56	Değirmencioğlu E, Kiliçoğlu H. Evaluating the impact of social media marketing from the perspective of orthodontists. BMC Oral Health [Internet]. 2024;24(1). Available from: https://www.scopus.com/inward/record.uri?eid=2-s2.0-85198074162&doi=10.1186%2Fs12903-024-04558-2&partnerID=40&md5=59e65cb3ecc0bab81e7f3c03809ca180	DUPLICATE PUBMED #61		
57	Dadgar S, Armin M, Ebrahimpour A, Moosazadeh M, Yousefi S, Sobouti F. Awareness of students in Mazandaran university of medical sciences about orthodontic treatment. Journal of Mazandaran University of Medical Sciences. 2019;29(175):98–106	Excluded		

58	Al-Moghrabi D, Johal A, Fleming PS. What are people tweeting about orthodontic retention? A cross-sectional content analysis. American Journal of Orthodontics and Dentofacial Orthopedics. 2017;152(4):516–22.	DUPLICATE PUBMED #11		
59	Mampieri G, Giancotti A. Invisalign technique in the treatment of adults with pre-restorative concerns. Progress in Orthodontics [Internet]. 2013;14(1). Available from: https://www.scopus.com/inward/record.uri?eid=2-s2.0-84891396255&doi=10.1186%2F2196-1042-14-40&partnerID=40&md5=f4c496000100ffa88dbffb3ca10f22a0	Excluded	Title/Abstract	
60	Wafaie K, Rizk MZ, Basyouni ME, Daniel B, Mohammed H. Tele-orthodontics and sensor-based technologies: a systematic review of interventions that monitor and improve compliance of orthodontic patients. European Journal of Orthodontics. 2023;45(4):450–61.	DUPLICATED PUBMED #225		
61	Hegarty E, Campbell C, Grammatopoulos E, DiBiase AT, Sherriff M, Cobourne MT. YouTubeTM as an information resource for orthognathic surgery. Journal of Orthodontics. 2017;44(2):90–6.	DUPLICATE PUBMED #93		
62	Cure RJ. Experiences of the challenges of undertaking an orthodontic needs assessment within the National Health Service in England. Journal of Orthodontics. 2019;46(2):143–7.	DUPLICATE PUBMED #54		
63	Crerand CE, Kapa HM, Litteral J, da Silveira AC, Markey MK. Adherence to Orthodontic Treatment in Youth With Craniofacial Conditions: A Survey of US Orthodontists. Cleft Palate Craniofacial Journal. 2019;56(10):1322–32.	DUPLICATE PUBMED #52		
64	Silveira GS, de Gauw JH, Mucha JN. Hypoplastic Canine: Would Treatment Decision be Different if the Diagnosis Were Made with Computed Tomography? International journal of orthodontics (Milwaukee, Wis). 2016;27(3):77–82	DUPLICATE PUBMED #205		

65	Knösel M, Jung K. Informational value and bias of videos related to orthodontics screened on a video-sharing Web site. Angle Orthodontist. 2011;81(3):532–9.	DUPLICATE PUBMED #124		
66	Almoammar S, Asiri E, Althogbi SI, Saad R, Al-Shahrani A, Hassan N, et al. Knowledge and attitude of general population towards orthodontic treatment in Aseer Region, Kingdom of Saudi Arabia. World Journal of Dentistry. 2017;8(6):483–9.	Excluded	Title/Abstract	
67	Krishnan V. Digital Opinion Leaders: The new age influencers! Journal of the World Federation of Orthodontists. 2025;14(3):123–4.	Excluded	Title/Abstract	
68	Knösel M, Engelke W, Helms HJ, Bleckmann A. An appraisal of the current and potential value of Web 2.0 contributions to continuing education in oral implantology. European Journal of Dental Education. 2012;16(3):131–7.	DUPLICATE PUBMED #125		
69	Magoi JS, Echezona RI. Academic Librarians' Activities in Creating Social-Media Authority in Three Selected Nigerian University Libraries. African Journal of Library Archives and Information Science. 2022;32(1):65–79.	Excluded	Title/Abstract	
70	Watts GD, Christou P, Antonarakis GS. Experiences of Individuals Concerning Combined Orthodontic and Orthognathic Surgical Treatment: A Qualitative Twitter Analysis. Medical Principles and Practice. 2018;27(3):227–35.	DUPLICATE PUBMED #230		
71	Al-Moghrabi D, Colonio-Salazar FB, Johal A, Fleming PS. Development of 'My Retainers' mobile application: Triangulation of two qualitative methods. Journal of Dentistry [Internet]. 2020;94. Available from: https://www.scopus.com/inward/record.uri?eid=2-s2.0-85079272390&doi=10.1016%2Fj.jdent.2020.103281&partnerID=40&md5=8ee10bf4607c7ba594c2c2c802fdc8e1	DUPLICATE PUBMED #12		

72	Rebaque Pistoni T, de la Cruz-Pérez J, Nieto-Sánchez I. Influence of social media on the esthetic perception of the lip profile of orthodontic patients. Heliyon [Internet]. 2023;9(5). Available from: https://www.scopus.com/inward/record.uri?eid=2-s2.0-85159455528&doi=10.1016%2Fj.heliyon.2023.e15870&partnerID=40&md5=667dae8effda233542b6009ce9f753e7	DUPLICATE PUBMED #178		
73	Smith BE, Hendrick P, Bateman M, Moffatt F, Rathleff MS, Selfe J, et al. Current management strategies for patellofemoral pain: An online survey of 99 practising UK physiotherapists. BMC Musculoskeletal Disorders [Internet]. 2017;18(1). Available from: https://www.scopus.com/inward/record.uri?eid=2-s2.0-85019125764&doi=10.1186%2Fs12891-017-1539-8&partnerID=40&md5=17889aedccce9f0c0d40829fe40350b6	Excluded	Title/Abstract	
74	Gandedkar NH, Lee BP, Pangrazio-Kulbersh V, Alansari R, Castillo JL, Cruz RM, et al. World Federation of Orthodontists social media guidelines: Ensuring accuracy, reliability, and objectivity in online orthodontic information. Journal of the World Federation of Orthodontists. 2025;14(4):187–93.	DUPLICATE PUBMED #75		
75	Kuah D, Burne S, Crichton K. Downhill skiing and snowboarding injuries: A guide for GPs. Medicine Today. 2005;6(8):61–6.	Excluded	Title/Abstract	
76	Arslan C, Aksahin EC, Nur Yilmaz RB, Cakan D. Does YouTubeTM Offer High-Quality Information About Nasoalveolar Molding? Cleft Palate Craniofacial Journal. 2024;61(1):5–11.	DUPLICATE PUBMED #29		
77	Oliveira LM, Zanatta FB. Self-reported dental treatment needs during the COVID-19 outbreak in Brazil: an infodemiological study. Brazilian Oral Research [Internet]. 2020;34. Available from: https://www.scopus.com/inward/record.uri?eid=2-s2.0-	DUPLICATE PUBMED #165		

	85090711719&doi=10.1590%2F1807-3107bor-2020.vol34.0114&partnerID=40&md5=5c9776a414011c6e4f6eea88d8aab76f			
78	Sweis O, Lomasney LM, Demos TC, Lebioda K, Hijaz T, Ghanayem A. The case: Diagnosis: Chance Fracture. Orthopedics. 2011;34(12):925–1014.	Excluded	Title/Abstract	
79	Berry L, Jones A, Barber S. Laypeople's interpretation of, and preference for, orthodontic images. Journal of Orthodontics. 2022;49(3):296–303.	DUPLICATE PUBMED #36		
80	Cho HE, Jang CW, Cho SR, Choi WA, Park JH. Mobile Apps to Improve Brace-Wearing Compliance in Patients with Idiopathic Scoliosis: A Quality Analysis, Functionality Review and Future Directions. Journal of Clinical Medicine [Internet]. 2023;12(5). Available from: https://www.scopus.com/inward/record.uri?eid=2-s2.0-85149951244&doi=10.3390%2Fjcm12051972&partnerID=40&md5=8348e879d7121b0e73618fa4c777e3f6	Excluded	Title/Abstract	
81	Guan J, Bisson EF. Treatment of Odontoid Fractures in the Aging Population. Neurosurgery Clinics of North America. 2017;28(1):115–23.	DUPLICATE PUBMED #89		
82	Cannatà D, Galdi M, Martina S, Rongo R, D'Antò V, Valletta R, et al. Preformed Elastodontic Appliances: Awareness and Attitude of Orthodontists and General Dental Practitioners. Children [Internet]. 2024;11(4). Available from: https://www.scopus.com/inward/record.uri?eid=2-s2.0-85191358555&doi=10.3390%2Fchildren11040418&partnerID=40&md5=3a4222aa896c6a7efa955abeb007b11e	DUPLICATE PUBMED #45		
83	Leigh-Smith S, Price R, Summers D. Atlas: Standard diagnostic tests for an unusual fracture. Emergency Medicine Journal. 2005;22(3):225–6.	Excluded	Title/Abstract	

84	Craven J, Davies I, Perry DC. The role of weaning in brace treatment for developmental dysplasia of the hip time to define best practice? Bone and Joint Open. 2025;6(6):685–90.	DUPLICATE PUBMED #51		
85	Lexner MO, Almer L. Case series: Treatment considerations in x-linked hypohidrotic ectodermal dysplasia. European archives of paediatric dentistry : official journal of the European Academy of Paediatric Dentistry. 2009;10 Suppl 1:26–30.	DUPLICATE PUBMED #130		
86	Montebugnoli F, Incerti-Parenti SI, D'Antò V, Alessandri-Bonetti G, Michelotti A. Effect of verbal and written information on pain perception in patients undergoing fixed orthodontic treatment: A randomized controlled trial. European Journal of Orthodontics. 2020;42(5):494–9	DUPLICATE PUB 153		
87	Nevins ML, Gartner-Sekler JL. Periodontal, implant, and prosthetic treatment for advanced periodontal disease. Compendium of continuing education in dentistry (Jamesburg, NJ : 1995). 1997;18(5):469–80.	DUPLICATE PUBMED #161		
88	Tang H, Xiao YF, Liu WJ, Meng JH, Wu YM, Xiong YL, et al. Preferences in anterior cruciate ligament reconstruction: A survey among orthopedic surgeons in China. Medicine (United States). 2024;103(7):E36482.	Excluded	Title/Abstract	
89	Kim HJ, Green DW. Spondylolysis in the adolescent athlete. Current Opinion in Pediatrics. 2011;23(1):68–72.	Excluded	Title/Abstract	
90	Bullock L, Holden MA, Jinks C, Atiah Asamane E, Herron D, Borrelli B, et al. Physiotherapists' Experiences and Perceived Acceptability of Delivering a Knee Bracing Intervention for People With Symptomatic Knee Osteoarthritis in a Randomised Trial (PROP OA): A Qualitative Study. Musculoskeletal Care [Internet]. 2024;22(4). Available from: https://www.scopus.com/inward/record.uri?eid=2-s2.0-85212399186&doi=10.1002%2Fmsc.70021&partnerID=40&md5=3c8b5e975a3bdfc2c3b9e2dc0a319415	DUPLICATE PUBMED #43		

91	Kenealy PM, Kingdon A, Richmond S, Shaw WC. The Cardiff dental study: A 20-year critical evaluation of the psychological health gain from orthodontic treatment. British Journal of Health Psychology. 2007;12(1):17–49.	DUPLICATE PUBMED #116		
92	Cheung T, Park J, Lee D, Kim C, Olson J, Javadi S, et al. Ability of mini-implant–facilitated micro-osteoperforations to accelerate tooth movement in rats. American Journal of Orthodontics and Dentofacial Orthopedics. 2016;150(6):958–67.	DUPLICATE PUB 48		
93	Elenko E, Speier A, Zohar D. A regulatory framework emerges for digital medicine. Nature Biotechnology. 2015;33(7):697–702.	Excluded	Title/Abstract	
94	Saccomanno S, Saran S, Guercio Monaco E, Mastrapasqua RF, Pirino A, Scoppa F. The Influence of the COVID-19 Pandemic on Orthodontic Treatments: A Survey Analysis. Dentistry Journal [Internet]. 2022;10(2). Available from: https://www.scopus.com/inward/record.uri?eid=2-s2.0-85123909718&doi=10.3390%2Fdj10020015&partnerID=40&md5=650fdaf62a11be56c34f8fa5bf689b6d	DUPLICATE PUBMED #86		
95	Zhu Z, Ye Z, Wang Q, Li R, Li H, Guo W, et al. Evolutionary Trend of Dental Health Care Information on Chinese Social Media Platforms During 2018-2022: Retrospective Observational Study. JMIR Infodemiology [Internet]. 2025;5.	DUPLICATE PUB 245		
96	Alrashid A, Burezq H. Parental perception of cleft lip in Kuwait. Current Pediatric Research. 2018;22(3):219–24.	Excluded	Title/Abstract	
97	Tanki H, Wani A, Ramzan A, Malik N, Chhibber S, Dar B, et al. Conservative management of craniovertebral junction injuries: Still a good option. Surgical Neurology International [Internet]. 2017;8(1). Available from: https://www.scopus.com/inward/record.uri?eid=2-s2.0-	Excluded	Title/Abstract	

	85020478075&doi=10.4103%2Fsni.sni_340_16&partnerID=40&md5=d7e5f5bef63e07607a8abc13ae8455ca			
98	Bogdanov V, Ilova D, Yordanova G. Adolescent Patient's Perceptions during Treatment with Class II Elastics. Case Reports in Dentistry [Internet]. 2024;2024.	DUPLICATE PUBMED #41		
99	Asgarian B, Shokrgozar HR. Evaluation of buckling-restrained braced frames overstrength factor. In 2007. p. 799–804.	Excluded	Title/Abstract	
100	Mahadware A, Saigiridhari A, Mishra A, Tupe A, Marathe N. Rainfall Prediction using Different Machine Learning and Deep Learning Algorithms. In 2022. Available from: https://www.scopus.com/inward/record.uri?eid=2-s2.0-85141565223&doi=10.1109%2FASIANCON55314.2022.9908857&partnerID=40&md5=7d8bd3dfddce3a7eeac7e4022400895a	Excluded	Title/Abstract	
101	Frawley T, Parkin N, Kettle J, Longstaff S, Benson P. Young people's experiences of orthodontic retainers: A qualitative study. Journal of Orthodontics. 2022;49(4):394–402.	DUPLICATE PUBMED #73		
102	Abdulhameed AZ, Ahmed MA, Almayah AA. The Impact of Bracing System Distribution and Location on Seismic Performance Enhancement of Multi-Story Steel Buildings. Mathematical Modelling of Engineering Problems. 2023;10(5):1833–41.	Excluded	Title/Abstract	
103	Heckmann K, Spitzer WJ, Lisson JA. Molar inclination in panoramic x-rays as an indicator for extraction decisions. Journal of Orofacial Orthopedics. 2007;68(6):491–500.	Excluded	Title/Abstract	
104	Bhattacharya S, Chatterjee P, Dutta S. Common orthopaedic problems in day to day general practice (in economically handicapped community). Journal of the Indian Medical Association. 2007;105(5):263–70.	DUPLICATE PUBMED #38		

105	Jedliński M, Mazur M, Schmeidl K, Grocholewicz K, Ardan R, Janiszewska-Olszowska J. Orthodontic Retention—Protocols and Materials—A Questionnaire Pilot Study among Polish Practitioners. Materials [Internet]. 2022;15(2). Available from: https://www.scopus.com/inward/record.uri?eid=2-s2.0-85122849082&doi=10.3390%2Fma15020666&partnerID=40&md5=58d3808f7e3ef40eb65acc92795eb3ea	DUPLICATE PUBMED #106		
106	Oliveira SC, Furquim RD, Ramos AL. Impact of brackets on smile esthetics: Laypersons and orthodontists perception. Dental Press Journal of Orthodontics. 2012;17(5):64–70.	Excluded	Title/Abstract	
107	Kuroshima K, Miyazaki S, Hiranaka Y, Ryu M, Inoue S, Yurube T, et al. Risk Factors for Nonunion After Nonoperative Treatment for Pediatric Lumbar Spondylolysis: A Retrospective Case-Control Study. American Journal of Sports Medicine. 2024;52(11):2866–73.	DUPLICATE PUBMED #127		
108	Bungău TC, Moca AE, Ciavoi G, Ignat-Romanul IM, Vaida LL, Buhas CL. Usage and Preferences of Orthodontic Mini-Implants Among Romanian Practitioners: A Survey Study. Dentistry Journal [Internet]. 2024;12(12). Available from: https://www.scopus.com/inward/record.uri?eid=2-s2.0-85213419656&doi=10.3390%2Fdj12120400&partnerID=40&md5=f1b99facc091f5fd0dcb5c6916c08145	DUPLICATE PUBMED #44		
109	Hashemi A, Clifton GC, Bagheri H, Zarnani P, Quenneville P. Proposed design procedure for steel self-centring tension-only braces with resilient connections. Structures. 2020;25:147–56.	Excluded	Title/Abstract	
110	Lee YJ, Wang WJ, Mohamad SM, Chandren JR, Gani SMA, Chung WH, et al. A comparison between Boston brace and European braces in the treatment of adolescent idiopathic scoliosis (AIS) patients: a systematic review based on the standardised Scoliosis Research Society (SRS)	Excluded	Title/Abstract	

	inclusion criteria for brace treatment. European Spine Journal. 2024;33(2):630–45.			
111	Aulisa AG, Giordano M, Falciglia F, Marzetti E, Poscia A, Guzzanti V. Correlation between compliance and brace treatment in juvenile and adolescent idiopathic scoliosis: SOSORT 2014 award winner. Scoliosis [Internet]. 2014;9(1). Available from: https://www.scopus.com/inward/record.uri?eid=2-s2.0-84902057658&doi=10.1186%2F1748-7161-9-6&partnerID=40&md5=76c718e6afd90833daa6a51a68ccf27f	Excluded	Title/Abstract	
112	Karkazi F, Antoniadou M, Demeterová K, Konstantonis D, Margaritis V, Lysy J. Orthodontic Risk Perspectives among Orthodontists during Treatment: A Descriptive Pilot Study in Greece and Slovakia. Healthcare (Switzerland) [Internet]. 2024;12(4). Available from: https://www.scopus.com/inward/record.uri?eid=2-s2.0-85185672456&doi=10.3390%2Fhealthcare12040492&partnerID=40&md5=278e5dea695c138fcfb3dc205f8369e2	DUPLICATE PUBMED #114		
113	Mattei TA, Hanovnikian J, Dinh DH. Progressive kyphotic deformity in comminuted burst fractures treated non-operatively: the Achilles tendon of the Thoracolumbar Injury Classification and Severity Score (TLICS). European Spine Journal. 2014;23(11):2255–62.	DUPLICATE PUBMED #146		
114	Turkalj M, Ghosh M, Čokić SM, Hoet PHM, Vanoirbeek J, van Meerbeek B, et al. Cytotoxicity assessment of eluates from vacuum-forming thermoplastics. Clinical Oral Investigations [Internet]. 2024;28(10). Available from: https://www.scopus.com/inward/record.uri?eid=2-s2.0-85205605529&doi=10.1007%2Fs00784-024-05952-4&partnerID=40&md5=32d006df1c19829ca11f66f4fb1db5ee	DUPLICATE PUBMED #221		

115	Condon SB, Veitch M. The experience of pregnancy associated osteoporosis: An international survey with implications for midwifery care. Midwifery [Internet]. 2022;115. Available from: https://www.scopus.com/inward/record.uri?eid=2-s2.0-85137693507&doi=10.1016%2Fj.midw.2022.103468&partnerID=40&md5=75bcbd0bd41475bf66f6f426e7ead591	DUPLICATE PUBMED #50		
116	Miller JD, Nader R. Treatment of combined osteoporotic compression fractures and spinal stenosis: Use of vertebral augumentation and interspinous process spacer. Spine. 2008;33(19):E717–20.	Excluded	Title/Abstract	
117	Gusella F, Orlando M, Peterman KD. Influence of mechanical and geometric uncertainty on the seismic performance of cold-formed steel braces with additional holes. Structures. 2021;29:494–506.	Excluded	Title/Abstract	
118	Markusi M, Blagec T, Šimunović L, Lapter-Varga M, Mestrovic S. Awareness and usage of mobile applications as an orthodontic diagnostic tool among Croatian orthodontic clinicians. Journal of Orthodontics. 2024;51(2):130–6.	DUPLICATE PUBMED #145		
119	Palmer K, Roeder C, Okazaki T, Shield C, Lehman D. Three-dimensional tests of two-story, one-bay by one-bay, steel concentric braced frames. In 2011. p. 3057–67. Available from: https://www.scopus.com/inward/record.uri?eid=2-s2.0-79958155931&doi=10.1061%2F41171%28401%29266&partnerID=40&md5=3889e5b2873c41d534ff283772d0b772	Excluded	Title/Abstract	
120	Kim HW, Weinstein SL. The management of scoliosis in neurofibromatosis. Spine. 1997;22(23):2770–6.	DUPLICATE PUBMED #119		

121	Radcliff KE, Babak Kalantar SB, Reitman CA. Surgical management of spondylolysis and spondylolisthesis in athletes: Indications and return to play. Current Sports Medicine Reports. 2009;8(1):35–40.	Excluded	Title/Abstract	
122	Hu J, Qiu Y, Wang X, Jiang L, Lu X, Li M, et al. Flexible six-dimensional force sensor inspired by the tenon-and-mortise structure of ancient Chinese architecture for orthodontics. Nano Energy [Internet]. 2022;96.	Excluded	Title/Abstract	
123	Khoury L, Chang E, Hill D, Shams S, Sim V, Panzo M, et al. Management of thoracic and lumbar spine fractures: Is MRI necessary in patients without neurological deficits? In 2019. p. 306–11. Available from: https://www.scopus.com/inward/record.uri?eid=2-s2.0-85064325255&partnerID=40&md5=38254a88c1c6d1de333c1771c9550b70	Excluded	Title/Abstract	
124	Donzelli S, Zaina F, Minnella S, Lusini M, Negrini S. Consistent and regular daily wearing improve bracing results: A case-control study. Scoliosis and Spinal Disorders [Internet]. 2018;13(1). Available from: https://www.scopus.com/inward/record.uri?eid=2-s2.0-85050663976&doi=10.1186%2Fs13013-018-0164-0&partnerID=40&md5=ae6de28f6ddcb9a97bf3f8422a6acba7	DUPLICATE PUBMED #62		
125	Kuba MHM, Izuka BH. One Brace: One Visit: Treatment of Pediatric Distal Radius Buckle Fractures with a Removable Wrist Brace and No Follow-up Visit. Journal of Pediatric Orthopaedics. 2018;38(6):e338–42.	Excluded	Title/Abstract	
126	Ertel AE, Robinson BRH, Eckman MH. Cost-effectiveness of cervical spine clearance interventions with litigation and long-term-care implications in obtunded adult patients following blunt injury. Journal of Trauma and Acute Care Surgery. 2016;81(5):897–904.	DUPLICATE PUBMED #66		
127	Mengi A, Singh RP, Mengi N, Kalgotra S, Singh A. A questionnaire study regarding knowledge, attitude and usage of artificial intelligence and	DUPLICATE PUBMED #152		

	machine learning by the orthodontic fraternity of Northern India. Journal of Oral Biology and Craniofacial Research. 2024;14(5):500–6.			
128	Luk JKH, Tsang RCC, Leung HB. Lateral epicondylalgia: Midlife crisis of a tendon. Hong Kong Medical Journal. 2014;20(2):145–51.	DUPLICATE PUBMED #140		
129	Abutayyem H. In-Vitro Investigation of the Shear Bond Strength of Different Orthodontic Adhesives to Enamel. Journal of Pharmacy and Bioallied Sciences. 2024;16:S2473–5.	DUPLICATE PUBMED #3		
130	Jaffray DC, Eisenstein SM, Balain B, Trivedi JM, Newton-Ede M. Early mobilisation of thoracolumbar burst fractures without neurology. Bone and Joint Journal. 2016;98B(1):97–101.	DUPLICATE PUBMED #104		
131	Yamada K, Hassan GS, Yamaki M, Hanada K. Use of X-rays in the computer-assisted patient information system. Journal of Clinical Orthodontics. 1996;30(4):206–7.	DUBLICATE PUBMED #239		
132	Espeland LV, Ivarsson K, Stenvik A. A new Norwegian index of orthodontic treatment need related to orthodontic concern among 11-year-olds and their parents. Community Dentistry and Oral Epidemiology. 1992;20(5):274–9.	DUPLICATE PUBMED #67		
133	Cheng H, Wen H, Ma Y, Liu Z, Wu H, Luowu L, et al. Development and Validation of a Nomogram for Predicting Adjacent Vertebral Fracture After Osteoporotic Vertebral Compression Fracture Surgery: A Multicenter Retrospective Cohort Study. Neurospine. 2025;22(2):592–602.	Excluded	Title/Abstract	
134	Thiruvenkatachari B, Sivakumar P, Ananth S, Sabbagh Y, Lewis BRK, Chadwick SM, et al. The impact of COVID-19 pandemic on orthodontic services and trainees' mental health in India. Frontiers in Medicine [Internet]. 2023;10. Available from:	DUPLICATE PUBMED #218		

	https://www.scopus.com/inward/record.uri?eid=2-s2.0-85171335064&doi=10.3389%2Ffmed.2023.1220505&partnerID=40&md5=2cd767d75e4520673f5f4781a06c756b			
135	Zheng JL, Li Y, Hogue G, Johnson M, Anari JB, Regan MD, et al. What imaging does my AIS patient need? A multi-group survey of provider preferences. Spine Deformity. 2025;13(2):351–9.	Excluded	Title/Abstract	
136	Koestner AJ, Hoak SJ. Spinal cord injury without radiographic abnormality (SCIWORA) in children. Journal of trauma nursing : the official journal of the Society of Trauma Nurses. 2001;8(4):101–8.	Excluded	Title/Abstract	
137	Xin YF, Rong SS, You AM, Hu YF. Brace materials for patients with post-stroke hemiplegia: Categories and biocompatibility. Chinese Journal of Tissue Engineering Research. 2015;19(30):4887–91.	Excluded	Title/Abstract	
138	Öziç MU, Gelincik B. Artificial Intelligence-Based Pose Estimation Model for Anatomical Landmark Detection in Lateral Cephalometric X-Rays. In 2024. Available from: https://www.scopus.com/inward/record.uri?eid=2-s2.0-85207898196&doi=10.1109%2FIDAP64064.2024.10710665&partnerID=40&md5=e74a8c5080656f790c802dca80dca71f	Excluded	Title/Abstract	
139	Wang Y, Xu P, Wang Y, Liu Y, Xu S, Zhao Z, et al. Biomechanical study of lumbar vertebra during gait cycle in adolescent idiopathic scoliosis. Shengwu Yixue Gongchengxue Zazhi/Journal of Biomedical Engineering. 2025;42(3):601–9.	DUPLICATE PUBMED #229		
140	Grimm BD, Leas DP, Glaser JA. The utility of routine postoperative radiographs after cervical spine fusion. Spine Journal. 2013;13(7):764–9.	Excluded	Title/Abstract	

141	Gütl C, Cheong C, Cheong F, Chang V, Nau SZ, Pirker J. Expectations of the generation NeXt in higher education: Learning engagement approaches in information sciences subjects. In 2015. p. 205–14. Available from: https://www.scopus.com/inward/record.uri?eid=2-s2.0-84964758597&doi=10.1109%2FICL.2015.7318027&partnerID=40&md5=3e6e7f357f98ad5146636be4fe7747fb	Excluded	Title/Abstract	
142	Hyam DM. The contemporary management of third molars. Australian Dental Journal. 2018;63:S19–26.	DUPLICATE PUBMED #102		
143	Presciutti SM, Karukanda T, Lee M. Management decisions for adolescent idiopathic scoliosis significantly affect patient radiation exposure. Spine Journal. 2014;14(9):1984–90.	Excluded	Title/Abstract	
144	Gülabi D, Sarı F, Sẹn C, Avci CC, Sağlam F, Erdem M, et al. Mid-term results of calcaneal plating for displaced intraarticular calcaneus fractures. Ulusal Travma ve Acil Cerrahi Dergisi. 2013;19(2):145–51.	Excluded	Title/Abstract	
145	Bjerklin K, Bondemark L. Ectopic maxillary canines and root resorption of adjacent incisors Does computed tomography (CT) influence decision-making by orthodontists? Swedish Dental Journal. 2008;32(4):179–85.	DUPLICATE PUBMED #40		
146	Kim SH, Lee CH. Chord Sidewall Failure of RHS X-Joints in Compression and Associated Design Recommendations. Journal of Structural Engineering [Internet]. 2021;147(8).	Excluded	Title/Abstract	
147	. Frankl P, Kupavskiĭ A. Non-trivial r-wise agreeing families. European Journal of Combinatorics [Internet]. 2025;126. Available from: https://www.scopus.com/inward/record.uri?eid=2-s2.0-85217257607&doi=10.1016%2Fj.ejc.2025.104129&partnerID=40&md5=6a9f90ae6cf77bccfcdaebc9df865fc4	Excluded	Title/Abstract	

148	Faggiano B, Fiorino L, Formisano A, Macillo V, Castaldo C, Mazzolani FM. Assessment of the design provisions for steel concentric X bracing frames with reference to Italian and European codes. Open Construction and Building Technology Journal. 2014;8:208–15.	Excluded	Title/Abstract	
149	Abe T, Shibao Y, Takeuchi Y, Mataki Y, Amano K, Hioki S, et al. Initial hospitalization with rigorous bed rest followed by bracing and rehabilitation as an option of conservative treatment for osteoporotic vertebral fractures in elderly patients: a pilot one arm safety and feasibility study. Archives of Osteoporosis [Internet]. 2018;13(1).	DUPLICATE PUBMED #2		
150	Maserati MB, Stephens B, Zohny Z, Lee JY, Kanter AS, Spiro RM, et al. Occipital condyle fractures: Clinical decision rule and surgical management. Clinical article. Journal of Neurosurgery: Spine. 2009;11(4):388–95.	Excluded	Title/Abstract	
151	Patano A, Cirulli N, Beretta M, Plantamura P, Inchingolo AD, Inchingolo AM, et al. Education technology in orthodontics and paediatric dentistry during the covid-19 pandemic: A systematic review. International Journal of Environmental Research and Public Health [Internet]. 2021;18(11).	Excluded	Title/Abstract	
152	Hua F, Qin D, Yan J, Zhao T, He H. COVID-19 Related Experience, Knowledge, Attitude, and Behaviors Among 2,669 Orthodontists, Orthodontic Residents, and Nurses in China: A Cross-Sectional Survey. Frontiers in Medicine [Internet]. 2020;7. Available from: https://www.scopus.com/inward/record.uri?eid=2-s2.0-85089848637&doi=10.3389%2Ffmed.2020.00481&partnerID=40&md5=98fb4912daccc3d54fdf0541c98d7c45	DUPLICATE PUBMED #98		

153	Schumacher HA, Bourauel C, Drescher D. Frictional forces when rectangular guiding arches with varying edge bevel are employed. Journal of Orofacial Orthopedics. 1998;59(3):139–49.	DUPLICATE PUBMED #194		
154	Harvey Z, Chamis M, Lin R. The Impact of Lateral Pads versus Posterolateral Pads in the Management of Idiopathic Scoliosis. Journal of Prosthetics and Orthotics. 2002;14(4):165–9.	Excluded	Title/Abstract	
155	Haney E, Gansky SA, Lee JS, Johnson E, Maki K, Miller AJ, et al. Comparative analysis of traditional radiographs and cone-beam computed tomography volumetric images in the diagnosis and treatment planning of maxillary impacted canines. American Journal of Orthodontics and Dentofacial Orthopedics. 2010;137(5):590–7.	DUPLICATE PUBMED #91		
156	Salek F, El Idrissi I, El Alloussi M, Zaoui F, Azaroual MF. Corono-radicular dilaceration of a maxillary central incisor: A case report. International Orthodontics. 2019;17(3):606–12.	DUPLICATE PUBMED #188		
157	Atchison KA, Luke LS, White SC. An algorithm for ordering pretreatment orthodontic radiographs. American Journal of Orthodontics and Dentofacial Orthopedics. 1992;102(1):29–44.	DUPLICATE PUBMED #31		
158	Rampon FB, Nóbrega C, Bretos JLG, Arsati F, Jakob S, Jimenez-Pellegrin MC. Profile of the orthodontist practicing in the state of São Paulo - Part 2. Dental Press Journal of Orthodontics. 2013;18(1):32-e6.	DUPLICATE PUBMED #176		
159	de Oliveira Silva YR, Zheng D, Peters SC, Fisher OS. Stabilization of a Cu-binding site by a highly conserved tryptophan residue. Journal of Inorganic Biochemistry [Internet]. 2024;253. Available from: https://www.scopus.com/inward/record.uri?eid=2-s2.0-85184608817&doi=10.1016%2Fj.jinorgbio.2024.112501&partnerID=40&md5=333cddb839b0872860432ba14195aeb3	DUPLICATE PUBMED #59		

160	Tom K, Mancl L, Woloshyn H, Khosravi R, Bollen AM. Association between crowding estimation and extraction recommendations in orthodontics. American Journal of Orthodontics and Dentofacial Orthopedics. 2024;165(1):64-72.e12.	DUPLICATE PUBMED #220		
161	Rava A, Fusini F, Cinnella P, Massè A, Girardo M. Is cast an option in the treatment of thoracolumbar vertebral fractures? Journal of Craniovertebral Junction and Spine. 2019;10(1):51–6.	DUPLICATE PUBMED #177		
162	Vogt L, Hübscher M, Brettmann K, Banzer W, Fink M. Postural correction by osteoporosis orthosis (Osteo-med): A randomized, placebo-controlled trial. Prosthetics and Orthotics International. 2008;32(1):103–10.	Excluded	Title/Abstract	
163	Akbay A, Bilginer B, Akalan N. Closed manual reduction maneuver of atlantoaxial rotatory dislocation in pediatric age. Child's Nervous System. 2014;30(6):1083–9.	Excluded	Title/Abstract	
164	Griffiths HJ, Wagner J, Anglen J, Bunn P, Metzler M. The use of forced flexion/extension views in the obtunded trauma patient. Skeletal Radiology. 2002;31(10):587–91.	Excluded	Title/Abstract	
165	Chen ZQ, Zhao YF, He SS, Wang CF, Zhang JT, Zhao YC, et al. Factors as predictors for thoracic and thoracolumbar/lumbar structural curves in adolescent idiopathic scoliosis. Chinese Medical Journal. 2012;125(8):1439–42.	Excluded	Title/Abstract	
166	Sapata DM, de Oliveira E Silva C, Pascotto RC, Poleti TMFF, Arai MSI, Ramos AL. Periodontal indexes of two types of 3 x 3 retainers: 0.032-in SS V-loop versus 0.0215-in SS coaxial — a randomized crossover trial. Dental Press Journal of Orthodontics [Internet]. 2023;28(6). Available from: https://www.scopus.com/inward/record.uri?eid=2-s2.0-85182271812&doi=10.1590%2F2177-	DUPLICATE PUBMED #192		

	6709.28.6.e2323175.oar&partnerID=40&md5=f46e1950566f0a022f095e3d2933b8db			
167	Halimi A, Zaoui F. [Comparative study of measurement of posterior tooth crowding between the dental panoramic xray and dental scanner]. Odonto-Stomatologie Tropicale. 2012;35(137):13–20	DUPLICATE PUBMED #90		
168	Drohomyretska M, Tkachenko Y. THE METHOD OF ASSESSING THE DEGREE OF GLOSSOPTOSIS ACCORDING TO CLINICAL AND X-RAY ANTHROPOMETRICAL PREDICTORS: CLINICAL GUIDELINES. Georgian Medical News. 2024;350(5):57–62.	DUPLICATE PUBMED #63		
169	Mehra P, Neeraja R, Anbarasi J, Ravi V, Al Mazroa A. VGG-16 based Deep Learning Approach for Cephalometric Landmark Detection. Open Public Health Journal [Internet]. 2024;17. Available from: https://www.scopus.com/inward/record.uri?eid=2-s2.0-105000820423&doi=10.2174%2F0118749445338097240923063441&partnerID=40&md5=98591556e418e01b87e95c582dd23c4c	Excluded	Title/Abstract	
170	Sohrabi A, Babay Ahari S, Moslemzadeh H, Rafighi A, Aghazadeh Z. The reliability of clinical decisions based on the cervical vertebrae maturation staging method. European Journal of Orthodontics. 2016;38(1):8–12.	DUPLICATE PUBMED #208		
171	Alqahtani H. Trends and Protocols in Orthodontic Finishing: A Cross-sectional Study. Journal of the International Clinical Dental Research Organization. 2025;17(1):69–77.	Excluded	Title/Abstract	
172	Simpson S, Wallace CK, Vernazza CR. Paediatric dentistry provision in the North East of England: workforce confidence and attitudes. British Dental Journal [Internet]. 2022; Available from: https://www.scopus.com/inward/record.uri?eid=2-s2.0-	DUPLICATE PUBMED #206		

	85126447866&doi=10.1038%2Fs41415-022-4045-9&partnerID=40&md5=662cf6f5cc38154501987895b48fa7ed			
173	Sabetian PW, Owens JS, Maldonado DR, Miecznikowski KB, Saks BR, Jimenez AE, et al. Circumferential and Segmental Arthroscopic Labral Reconstruction of the Hip Utilizing the Knotless Pull-Through Technique with All-Suture Anchors. Arthroscopy Techniques. 2021;10(10):e2245–51.	Excluded	Title/Abstract	
174	Al-Naoum F, Hajeer MY, Al-Jundi A. Does alveolar corticotomy accelerate orthodontic tooth movement when retracting upper canines? A split-mouth design randomized controlled trial. Journal of Oral and Maxillofacial Surgery. 2014;72(10):1880–9.	Excluded	Title/Abstract	
175	Xu B, Yang K. Changes in alveolar bone structure during orthodontic tooth movement in adolescent and adult rats: A microcomputed tomography study. Orthodontics and Craniofacial Research. 2023;26(4):568–75.	DUPLICATE PUBMED #237		
176	Srinath S. Treacher collins syndrome. Journal of Pharmaceutical Sciences and Research. 2014;6(6):247–50.	Excluded	Title/Abstract	
177	Nouri M, Asefi S, Akbarzade Baghban A, Ahmadvand M, Shamsa M. Objective vs subjective analyses of arch form and preformed archwire selection. American Journal of Orthodontics and Dentofacial Orthopedics. 2016;149(4):543–54.	DUPLICATE PUBMED #163		
178	Parreira PCS, Maher CG, Megale RZ, March L, Ferreira ML. An overview of clinical guidelines for the management of vertebral compression fracture: a systematic review. Spine Journal. 2017;17(12):1932–8.	Excluded	Title/Abstract	
179	Homem MA, Pereira TS, Martins-Júnior PA, Ramos-Jorge ML, Marques LS. Malocclusion Severity Index in primary dentition: development and validation. Arquivos em Odontologia. 2024;60:146–54.	Excluded	Title/Abstract	

180	Chatziioannou K, Karamanos SA, Huang Y. Ultra low-cycle fatigue performance of S420 and S700 steel welded tubular X-joints. International Journal of Fatigue [Internet]. 2019;129. Available from: https://www.scopus.com/inward/record.uri?eid=2-s2.0-85071122780&doi=10.1016%2Fj.ijfatigue.2019.105221&partnerID=40&md5=496899ceb2205598398e04bfcecb4e60	Excluded	Title/Abstract	
181	Craven M, Vajravelu ME, Shekdar KV, Levine MA, Mumm S, Whyte MP, et al. Early identification of a 12-bp tandem duplication in TNFRSF11A encoding receptor activator of nuclear factor-kappa B (RANK): Clinical characterization and response to bisphosphonate therapy. Bone [Internet]. 2023;170.	Excluded	Title/Abstract	
182	Wang L, Quiroz C, Velasco A, Morales R, Vicuña D, Concha G, et al. Cephalometric Characteristics in Chilean Latino Population with Normal Occlusion and Harmonic Profiles in Permanent Dentition. A Retrospective Study. International Journal of Morphology. 2023;41(4):1020–6.	Excluded	Title/Abstract	
183	Chauhan M, Chauhan MR, Agrawal A, Chawla AR. Gauging the knowledge, attitude and practice of interns and dentists about obstructive sleep apnoea—A cross-sectional study. Lung India. 2024;41(1):25–9	Excluded	Title/Abstract	
184	Helms JA, Speidel T, Denis KL. Effect of timing on long-term clinical success of alveolar cleft bone grafts. American Journal of Orthodontics and Dentofacial Orthopedics. 1987;92(3):232–40.	DUPLICATE PUBMED #94		
185	Kirbschus A, Gesch D, Kaduk W, Gedrange T. The influence of craniofacial growth in a case of transverse facial cleft. Journal of Orofacial Orthopedics. 2006;67(3):215–24.	DUPLICATE PUBMED #120		

186	Manosudprasit A, Haghi A, Allareddy V, Masoud MI. Diagnosis and treatment planning of orthodontic patients with 3-dimensional dentofacial records. American Journal of Orthodontics and Dentofacial Orthopedics. 2017;151(6):1083–91	DUPLICATE PUBMED #144		
187	Nakano H, Satoh K, Norris R, Jin T, Kamegai T, Ishikawa F, et al. Mechanical properties of several nickel-titanium alloy wires in three-point bending tests. American Journal of Orthodontics and Dentofacial Orthopedics. 1999;115(4):390–5.	DUPLICATE PUBMED #155		
188	Lin L, Zhao T, Ngan P, Zhuang Y, Qin D, Hua F, et al. Obstructive sleep apnea-related knowledge, attitude, experience, and behaviors among orthodontic professionals: a survey. Sleep and Breathing. 2023;27(6):2361–9.	Excluded	Title/Abstract	
189	Almalki SA, Gowdar IM, Asiri SN, Jodalli P, Alanazi BA, Alqahtani OA, et al. Awareness of deleterious oral habits and knowledge of habit breaking appliance among parents in Alkharj - A cross sectional study. F1000Research [Internet]. 2024;13. Available from: https://www.scopus.com/inward/record.uri?eid=2-s2.0-85210025408&doi=10.12688%2Ff1000research.157200.1&partnerID=40&md5=bbd32824e726139a7ca4364dd99d34f6	Excluded	Title/Abstract	
190	Ashwanthi K, Prabhavathy B, Reshma S, Jyosthna A. KNOWLEDGE, AWARENESS AND PRACTICE OF DIGITAL ORTHODONTICS AMONG ORTHODONTISTS AND ORTHODONTIC RESIDENTS – A CROSS SECTIONAL STUDY. International Journal Of Orthodontic Rehabilitation. 2023;14(4):45–57.	Excluded	Title/Abstract	
191	Nasseh I, Al-Rawi W. Cone Beam Computed Tomography. Dental Clinics of North America. 2018;62(3):361–91.	DUPLICATE PUBMED #157		

192	Alqahtani H. Management of maxillary impacted canines: A prospective study of orthodontists' preferences. Saudi Pharmaceutical Journal. 2021;29(5):384–90.	Excluded	Title/Abstract	
193	Hou S, Zhou T, Liu Y, Dang P, Lu H, Shi H. Teeth U-Net: A segmentation model of dental panoramic X-ray images for context semantics and contrast enhancement. Computers in Biology and Medicine [Internet]. 2023;152. Available from: https://www.scopus.com/inward/record.uri?eid=2-s2.0-85145492333&doi=10.1016%2Fj.compbiomed.2022.106296&partnerID=40&md5=e484f94b33fcd2222be2eace22200d2a	DUPLICATE PUBMED #97		
194	Adeyemi TE, Adekoya MN, Aikins EA. Assessment of the willingness of Nigerian Orthodontists to offer face-to-face orthodontic services to patients infected with Covid-19. African Health Sciences. 2023;23(1):51–8.	Excluded	Title/Abstract	
195	Liu X, Luo Y, He X, Wang J, Li Z, Zhang Y, et al. Three-dimensional-printed hemi-pelvic prosthesis for revision of aseptic loosening or screw fracture of modular hemi-pelvic prosthesis. Chinese Journal of Reparative and Reconstructive Surgery. 2023;37(10):1183–9.			
196	Iber-Díaz P, Senen-Carramolino R, Iglesias-Linares A, Fernández-Navarro P, Flores-Mir C, Yáñez-Vico RM. GWAS of post-orthodontic aggressive external apical root resorption identified multiple putative loci at X-Y chromosomes. Journal of Personalized Medicine. 2020;10(4):1–16.	Excluded	Title/Abstract	
197	Fallis DW, Kusy RP. Novel esthetic bonded retainers: a blend of art and science. Clinical orthodontics and research. 1999;2(4):200–8.	Excluded	Title/Abstract	
198	Ghani MI. Treatment of congenital kyphosis. In 2015. p. 91–100. Available from: https://www.scopus.com/inward/record.uri?eid=2-s2.0-84956704300&partnerID=40&md5=c0c36167f6d65a25407658565fefa2d2	Excluded	Title/Abstract	

199	Huang Q, Lu Y, Ma T, Wang Q, Wang C, Li Z, et al. Pedicled Double-Barrel Fibular Transplantation Versus Bone Transport in the Treatment of Upper Tibial Osteomyelitis with Bone Defects: A Retrospective Study. Orthopaedic Surgery. 2022;14(11):2888–96.	Excluded	Title/Abstract	
200	Alakbarov A. Patella Fracture. In 2022. p. 145–64. Available from: https://www.scopus.com/inward/record.uri?eid=2-s2.0-85152337511&partnerID=40&md5=007a38344cdef601443651e6fcfb845c	Excluded	Title/Abstract	
201	Lindauer SJ, Powell JA, Leypoldt BC, Tüfekçi E, Shroff B. Influence of patient financial account status on orthodontic appointment attendance. Angle Orthodontist. 2009;79(4):755–8.	Excluded	Title/Abstract	
202	Sandham HJ, Nadeau L, Phillips HI. The Effect of Chlorhexidine Varnish Treatment on Salivary Mutans Streptococcal Levels in Child Orthodontic Patients. Journal of Dental Research. 1992;71(1):32–5.	DUPLICATE PUBMED #151		
203	Glotzbecker MP, Wasser AM, Troy MJ, Proctor M, Emans JB. Neonatal C1 to C2 Osteomyelitis Leading to Instability and Neurological Decline: Novel Treatment with Occiput-C1-C2 Fusion and Occiput to Thorax Growing Rods. A Case Report. Journal of Pediatric Orthopaedics. 2015;35(4):379–84.	DUPLICATE PUBMED #78		
204	Hubáček M, Kripnerová T, Němčíková M, Křepelová A, Puchmajerová A, Malíková M, et al. Odontogenic keratocysts in the Basal Cell Nevus (Gorlin-Goltz) Syndrome associated with paresthesia of the lower jaw: Case report, retrospective analysis of a representative Czech cohort and recommendations for the early diagnosis of the disease. Neuroendocrinology Letters. 2016;37(4):269–76.	Excluded	Title/Abstract	
205	Wu J, Pan Q. Semi-joint prosthesis replacement in treatment of malignant tumors around children’s knees. Chinese Journal of Reparative and Reconstructive Surgery. 2006;20(10):978–80.	DUPLICATE PUBMED #236		

206	Chang JS, Ma CY, Ko EWC. Prediction of surgery-first approach orthognathic surgery using deep learning models. International Journal of Oral and Maxillofacial Surgery. 2024;53(11):942–9.	Excluded	Title/Abstract	
207	Stamm T, Böttcher D, Kleinheinz J. The University Münster model surgery system for orthognathic surgery - The digital update. Head and Face Medicine [Internet]. 2021;17(1). Available from: https://www.scopus.com/inward/record.uri?eid=2-s2.0-85111307455&doi=10.1186%2Fs13005-021-00278-y&partnerID=40&md5=d69aac1442c803ef4cfcd5ba021e5b88	Excluded	Title/Abstract	
208	Pittet LF, Moore CL, McDonald E, Barry S, Bonten M, Campbell J, et al. Bacillus Calmette-Guérin vaccination for protection against recurrent herpes labialis: a nested randomised controlled trial. eClinicalMedicine [Internet]. 2023;64	DUPLICATE PUBMED #171		
209	Fischer-Brandies H, Orthuber W, Ermert M, Hussmanns A. The force module for the bending art system. Preliminary results. Journal of Orofacial Orthopedics. 1998;59(5):301–11.	DUPLICATE PUBMED #70		
210	Wörtche R, Hassfeld S, Lux CJ, Müssig E, Hensley FW, Krempien R, et al. Clinical application of cone beam digital volume tomography in children with cleft lip and palate. Dentomaxillofacial Radiology. 2006;35(2):88–94.	DUPLICATE PUBMED #235		
211	Szczęsny G, Ratajczak K, Fijewski G, Małdyk P. Intentional overscrewing of humeral head in comminuted fracture of proximal humerus and its impact on shoulder function. A case study. Ortopedia Traumatologia Rehabilitacja. 2019;21(4):297–305	DUPLICATE PUBMED #214		
212	Dipalma G, Inchingolo AD, Inchingolo AM, Piras F, Carpentiere V, Garofoli G, et al. Artificial Intelligence and Its Clinical Applications in Orthodontics: A Systematic Review. Diagnostics [Internet]. 2023;13(24). Available from: https://www.scopus.com/inward/record.uri?eid=2-s2.0-	Excluded	Title/Abstract	

	85180675301&doi=10.3390%2Fdiagnostics13243677&partnerID=40&md5=dc715f0e5190a7df1b6abf16e60f54aa			
213	Ataş O, Yildirim TT. Evaluation of knowledge, attitudes, and clinical education of dental students about COVID-19 pandemic. PeerJ [Internet]. 2020;8. Available from: https://www.scopus.com/inward/record.uri?eid=2-s2.0-85091695227&doi=10.7717%2Fpeerj.9575&partnerID=40&md5=def3b662d830b39e7814189e832ed2f4	Excluded	Title/Abstract	
214	Tang L, Chen M, Li G, Luo Z, Ji X, Zhang X, et al. Effectiveness of proximal femur reconstruction combined with total hip arthroplasty for Crowe type IV developmental dysplasia of hip. Chinese Journal of Reparative and Reconstructive Surgery. 2020;34(6):683–8.	DUPLICATE PUBMED #217		
215	Ayub MA, Azni MS, Al-Jaf N. Tooth frame axes and centroid for dental occlusal system. Pertanika Journal of Science and Technology. 2017;25(S4):325–34.	Excluded	Title/Abstract	
216	Erratum: Bisphenol A and orthodontics: An update of evidence-based measures to minimize exposure for the orthodontic team and patients (American Journal of Orthodontics & Dentofacial Orthopedics (2017) 152(4) (435–441) (S0889540617306984)(10.1016/j.ajodo.2017.08.004)). American Journal of Orthodontics and Dentofacial Orthopedics. 2017;152(6):740	Excluded	Title/Abstract	
217	Dyrna F, Imhoff FB, Haller B, Braun S, Obopilwe E, Apostolakos JM, et al. Primary Stability of an Acromioclavicular Joint Repair Is Affected by the Type of Additional Reconstruction of the Acromioclavicular Capsule. American Journal of Sports Medicine. 2018;46(14):3471–9.	DUPLICATE PUBMED #64		

218	McKiernan FE. Integrated anti-remodeling and anabolic therapy for the osteoporosis of Hajdu-Cheney syndrome. Osteoporosis International. 2007;18(2):245–9.	Excluded	Title/Abstract	
219	Debelmas A, Ketoff S, Lanciaux S, Corre P, Friess M, Khonsari RH. Reproducibility assessment of Delaire cephalometric analysis using reconstructions from computed tomography. Journal of Stomatology, Oral and Maxillofacial Surgery. 2020;121(1):35–9.	DUPLICATE PUBMED #60		
220	Ribeiro AA, Purger F, Rodrigues JA, Oliveira PRA, Lussi A, Monteiro AH, et al. Influence of contact points on the performance of caries detection methods in approximal surfaces of primary molars: An in vivo study. Caries Research. 2015;49(2):99–108.	DUPLICATE PUBMED #180		
221	Ey-Chmielewska H. An attempt to use ultrasonic technique for confirming the diagnosis, planning and observation of long-term treatment results of painful temporo-mandibular joint dysfunction. Annales Academiae Medicae Stetinensis. 1998;44:223–36.	DUPLICATE PUBMED #68		
222	Sheikh S, Pallagatti S, Singla I, Gupta R, Aggarwal A, Singh R, et al. Survey of dental radiographical practice in states of Punjab and Haryana in India. Journal of Investigative and Clinical Dentistry. 2014;5(1):72–7.	DUPLICATE PUBMED #202		
223	Capelozza-Filho LC, Machado FMC, Ozawa TO, Cavassan AO, Cardoso MA. Bracket/wire play: What to expect from tipping prescription on pre-adjusted appliances. Dental Press Journal of Orthodontics. 2012;17(4):85–95.	Excluded	Title/Abstract	
224	Felizardo R, Thomas A, Foucart JM. Useful radiological techniques in orthodontics. L'Orthodontie Française. 2012;83(1):11–22	DUPLICATE PUBMED #69		
225	AlQabandi AK, Sadowsky C, BeGole EA. A comparison of the effects of rectangular and round arch wires in leveling the curve of Spee. American Journal of Orthodontics and Dentofacial Orthopedics. 1999;116(5):522–9.	DUPLICATE PUBMED #23		

226	Rouas P, Bandon D, Nancy J, Delbos Y, Hauret L, Bar D. Digital volume tomography using the NewTom system: advantages of this new technique in children. Archives de Pediatrie. 2006;13(8):1169–77.	DUPLICATE PUBMED #184		
227	[No authors found]. 7 Book Reviews. Heythrop Journal 1987, 28(4):441–501	Excluded	Title/Abstract	
228	Yu J, Wang HM, Zhu XM, Fan ZL. Clinical study on the treatment of Class II and ClassI malocclusion in adolescents with mandibular recession using invisible orthodontic appliances without brackets. Shanghai Kou Qiang Yi Xue / Shanghai Journal of Stomatology. 2024;33(6):661–6.	DUPLICATE PUBMED #241		
229	Ricketts RM. The evolution of diagnosis to computerized cephalometrics. American Journal of Orthodontics. 1969;55(6):795–803.	Excluded	Title/Abstract	
230	Vasudavan S, Sullivan SR, Sonis AL. Comparison of intraoral 3D scanning and conventional impressions for fabrication of orthodontic retainers. Journal of Clinical Orthodontics. 2010;44(8):495–7.	DUPLICATE PUBMED #222		
231	Brandão NMCB, Palomares NB, Lima T, Quintão CCA, Lopes KB, Miguel JAM. Facial soft tissue changes in adolescent patients treated with three different functional appliances: a randomized clinical trial. Dental Press Journal of Orthodontics [Internet]. 2024;29(5).	Excluded	Title/Abstract	
232	Koppert E, Yi KH. Observational Study of Facial Rotation Patterns. Journal of Craniofacial Surgery [Internet]. 2025; Available from: https://www.scopus.com/inward/record.uri?eid=2-s2.0-105001409992&doi=10.1097%2FSCS.0000000000011278&partnerID=40&md5=4a0de5919d00e90ea9186d8c4aaef98a	DUPLICATE PUBMED #146		
233	Kimura K, Langland OE, Biggerstaff RH. The evaluation of high-speed screen/film combinations in cephalometric radiography. American Journal of Orthodontics and Dentofacial Orthopedics. 1987;92(6):484–91.	DUPLICATE PUBMED #118		

234	Grünheid T, Sudit GN, Larson BE. Debonding and adhesive remnant cleanup: An in vitro comparison of bond quality, adhesive remnant cleanup, and orthodontic acceptance of a flash-free product. European Journal of Orthodontics. 2015;37(5):497–502.	DUPLICATE PUBMED #86		
235	Rajpal G, Patni L, Dhariwal A, Kumar A, Visvarma S, Kumar A. Antioxidant and dentistry. In 2022. p. 669–80. Available from: https://www.scopus.com/inward/record.uri?eid=2-s2.0-85138377343&doi=10.1016%2FB978-0-12-819096-8.00055-0&partnerID=40&md5=58edee68fc9ff658327843fe2b503a61	Excluded	Title/Abstract	
236	Upadhyay M, Upadhyay V, Tandan A, Choukse V, Kumar S. Intentional replantation of oblique crown-root fracture - A case report. Indian Journal of Public Health Research and Development. 2012;3(1):16–8.	Excluded	Title/Abstract	
237	Nawar NN, Elashiry MM, El-Banna A, Saber SM, Schäfer E. Ex-vivo evaluation of clinically-set hydraulic sealers used with different canal dryness protocols and obturation techniques: a randomized clinical trial. Clinical Oral Investigations [Internet]. 2024;28(11).	DUPLICATE PUBMED #158		
238	Bimler HP, Bimler AB. Die Überwachung der kieferorthopädischen Behandlung durch individuelle Reaktionskurven. Fortschritte der Kieferorthopadie. 1987;48(5):371–8.	Excluded	Title/Abstract	
239	Giordano D, Leonardi R. Web-trace and the learning of visual discrimination skills. In 2007. p. 80–5. Available from: https://www.scopus.com/inward/record.uri?eid=2-s2.0-65549083415&doi=10.1109%2FPLT.2007.34&partnerID=40&md5=b93fe645f9105040c0f7124e468e800d	Excluded	Title/Abstract	

240	Oteri G, Panzarella V, Marcianò A, Di Fede O, Maniscalco L, Peditto M, et al. Appropriateness in Dentistry: A Survey Discovers Improper Procedures in Oral Medicine and Surgery. International Journal of Dentistry [Internet]. 2018;2018. Available from: https://www.scopus.com/inward/record.uri?eid=2-s2.0-85051050763&doi=10.1155%2F2018%2F3245324&partnerID=40&md5=ba16b856d33defcf911afb4369cc9a26	DUPLICATE PUBMED #167		
241	Azizi F, Zaseh MMS, Golshah A, Imani MM, Safari-Faramani R. Comparative efficacy of pharmaceutical (Ibuprofen) and non-pharmaceutical (photobiomodulation, and chewing gum) interventions for pain reduction after elastomeric separator placement in orthodontic patients: a randomized clinical trial. Lasers in Medical Science [Internet]. 2024;39(1). Available from: https://www.scopus.com/inward/record.uri?eid=2-s2.0-85204874257&doi=10.1007%2Fs10103-024-04186-w&partnerID=40&md5=7fb175510851eba55af6d6187d940de0	DUPLICATE PUBMED #32		
242	De Souza SLS, Novaes Junior AB, Pontes CC, Taba Junior M, Grisi MFM, Silveira e Souza AMM. Guided bone regeneration with intentionally exposed membranes and its implications for implant dentistry. A 6 months re-entry randomized clinical trial. Journal of Osseointegration. 2010;2(2):45–51.	Excluded	Title/Abstract	
243	Nehru A, Nagesh S. Smartphone-based 3D facial scanning applications in orthodontic diagnosis: A cross-sectional study. Journal of Dental Research, Dental Clinics, Dental Prospects. 2025;19(2):97–103.	DUPLICATE PUBMED #159		
244	Joham SJ, Hadzic A, Urschler M. Implicit Is Not Enough: Explicitly Enforcing Anatomical Priors inside Landmark Localization Models. Bioengineering [Internet]. 2024;11(9). Available from:	DUPLICATE PUBMED #107		

	https://www.scopus.com/inward/record.uri?eid=2-s2.0-85205061150&doi=10.3390%2Fbioengineering11090932&partnerID=40&md5=c461dd7b4aa9a1b35de2cb98e26d6959			
245	Rajesh Murugan R, Yezdani A. Skeletal maxillary bilateral posterior crossbite correction with decortication and bone autograft augmentation. Indian Journal of Public Health Research and Development. 2019;10(12):2392–8.	Excluded	Title/Abstract	
246	Gizani S, Seremidi K, Gkourtsogianni S, Mitsea A. Awareness and practice of dentomaxillofacial imaging among paediatric dentists: a questionnaire survey of members of the European Academy of Paediatric Dentistry. Oral Radiology. 2023;39(3):576–87.	DUPLICATE PUBMED #77		
247	Hembree M, Buschang PH, Carrillo R, Spears R, Rossouw P. Effects of intentional damage of the roots and surrounding structures with miniscrew implants. American Journal of Orthodontics and Dentofacial Orthopedics. 2009;135(3):280-280.e9.	DUPLICATE PUBMED #95		
248	Anderson JD, Szalai JP. The Toronto outcome measure for craniofacial prosthetics: A condition-specific quality-of-life instrument. International Journal of Oral and Maxillofacial Implants. 2003;18(4):531–8	Excluded	Title/Abstract	
249	Wood CV. Teaching and Learning Guide for: 'The Sociologies of Knowledge, Science, and Intellectuals: Distinctive Traditions and Overlapping Perspectives'. Sociology Compass. 2011;5(1):128–33.	Excluded	Title/Abstract	
250	Mainjot AKJ. No-Prep Zirconia Cantilever Resin-Bonded Fixed Dental Prostheses: A Noninvasive, Simple Approach to Replacing a Single Missing Tooth. Journal of Esthetic and Restorative Dentistry. 2025;37(1):68–84.	Excluded	Title/Abstract	

6. ProQuest				
**Search date**	25/8/2025			
**Results**	346			
**Search Query**	(Orthodontic* OR "orthodontic treatment" OR braces OR "clear aligners" OR Invisalign) AND ("Social Media" OR "social network*" OR Facebook OR Instagram OR TikTok OR YouTube OR Twitter OR X OR Snapchat) AND (willing* OR motivat* OR intent* OR perception OR attitude* OR "decision making" OR preference* OR "patient acceptance")			
**Nr.**	**Authors-Title**	**Eligibility**	**Reason for exclusion**	
1	Hongyang, Z. (2019). Growth and transformation of private dental clinics in china: A case study of deron dental (Order No. 31367380). Available from ProQuest Dissertations & Theses Global. (3110418383). Retrieved from https://www.proquest.com/dissertations-theses/growth-transformation-private-dental-clinics/docview/3110418383/se-2	Excluded	Title/Abstract	
2	Quesada, M. L. T. (2021). Fatores que influenciam a necessidade de tratamento ortodôntico - perceção de estudantes universitários (Order No. 31748581). Available from ProQuest Dissertations & Theses Global. (3143978823). Retrieved from https://www.proquest.com/dissertations-theses/fatores-que-influenciam-necessidade-de-tratamento/docview/3143978823/se-2	Excluded	Title/Abstract	

3	Tamošiūnaitė, I. (2022). Orthodontic pain in social media, youtube platform (Order No. 31264844). Available from ProQuest Dissertations & Theses Global. (3098805011). Retrieved from https://www.proquest.com/dissertations-theses/orthodontic-pain-social-media-youtube-platform/docview/3098805011/se-2	Excluded	Title/Abstract	
4	Iluz, G. M. (2024). The outcomes of malocclusion treatment with clear aligners and bracket system: Systematic literature review and meta analysis (Order No. 31660178). Available from ProQuest Dissertations & Theses Global. (3132852449). Retrieved from https://www.proquest.com/dissertations-theses/outcomes-malocclusion-treatment-with-clear/docview/3132852449/se-2	Excluded	Title/Abstract	
5	Baneshi, M. (2023). Effectiveness of clear orthodontic aligners in correcting malocclusion- systematic review (Order No. 31227603). Available from ProQuest Central; ProQuest Dissertations & Theses Global. (3098795571). Retrieved from https://www.proquest.com/dissertations-theses/effectiveness-clear-orthodontic-aligners/docview/3098795571/se-2	Excluded	Title/Abstract	
6	Francela, J. M. V. (2024). The correlation between orthodontic aligners and masticatory muscle activity: A scoping review (Order No. 32160126). Available from ProQuest Dissertations & Theses Global. (3235006269). Retrieved from https://www.proquest.com/dissertations-theses/correlation-between-orthodontic-aligners/docview/3235006269/se-2	Excluded	Title/Abstract	
7	Petrušytė, G. (2020). Pacientų gyvenimo kokybė skirtingomis ortodontinio gydymo breketų sistema stadijomis (Order No. 31263762). Available from ProQuest Dissertations & Theses Global. (3098801878).	Excluded	Title/Abstract	

	Retrieved from https://www.proquest.com/dissertations-theses/pacientų-gyvenimo-kokybė-skirtingomis/docview/3098801878/se-2			
8	Wanczewska, N. (2018). Oral health attitudes and knowledge among pediatric patients receiving orthodontic treatment. A cross-sectional study (Order No. 31270513). Available from ProQuest Dissertations & Theses Global. (3098809838). Retrieved from https://www.proquest.com/dissertations-theses/oral-health-attitudes-knowledge-among-pediatric/docview/3098809838/se-2	Excluded	Title/Abstract	
9	Bompard, R. (2020). Periodontal impact of orthodontic treatment with clear aligners (Order No. 30210897). Available from ProQuest Dissertations & Theses Global. (2763451927). Retrieved from https://www.proquest.com/dissertations-theses/periodontal-impact-orthodontic-treatment-with/docview/2763451927/se-2	Excluded	Title/Abstract	
10	Partouche, A. J. D. (2021). Effects of conventional orthodontic treatment versus transparent aligners in periodontal health: An integrative review (Order No. 30206354). Available from ProQuest Dissertations & Theses Global. (2748384569). Retrieved from https://www.proquest.com/dissertations-theses/effects-conventional-orthodontic-treatment-versus/docview/2748384569/se-2	Excluded	Title/Abstract	
11	Erotokritou, N. (2018). Comparison of periodontal health between patients with fixed orthodontic appliances and invisalign®: A systematic review (Order No. 31270515). Available from ProQuest Dissertations & Theses Global. (3098807958). Retrieved from https://www.proquest.com/dissertations-theses/comparison-periodontal-health-between-patients/docview/3098807958/se-2	Excluded	Title/Abstract	

12	Hourani, A. G. A. (2019). The effect of orthodontic treatment on traumatised teeth: A systematic review and vignette study (Order No. 29082007). Available from ProQuest Dissertations & Theses Global. (2665128144). Retrieved from https://www.proquest.com/dissertations-theses/effect-orthodontic-treatment-on-traumatised-teeth/docview/2665128144/se-2	Excluded	Title/Abstract	
13	Schenk, M. (2024). Treatment and long-term stability of maxillary diastema: A systematic review (Order No. 31660197). Available from ProQuest Dissertations & Theses Global. (3132852342). Retrieved from https://www.proquest.com/dissertations-theses/treatment-long-term-stability-maxillary-diastema/docview/3132852342/se-2	Excluded	Title/Abstract	
14	Monk, A. (2016). Pharmacological interventions for pain relief during orthodontic treatment (Order No. 10754857). Available from ProQuest Dissertations & Theses Global. (1985540774). Retrieved from https://www.proquest.com/dissertations-theses/pharmacological-interventions-pain-relief-during/docview/1985540774/se-2	Excluded	Title/Abstract	
15	Wu, T. (. (2022). Diagnosis and treatment evaluation in patient with short face (Order No. 31574091). Available from ProQuest Dissertations & Theses Global. (3095742857). Retrieved from https://www.proquest.com/dissertations-theses/diagnosis-treatment-evaluation-patient-with-short/docview/3095742857/se-2	Excluded	Title/Abstract	
16	Susarchick, L. (2021). Staining and cleaning effects of copolyester and copolymer retainer materials (Order No. 28722804). Available from ProQuest Dissertations & Theses Global. (2557226838). Retrieved from https://www.proquest.com/dissertations-theses/staining-cleaning-effects-copolyester-copolymer/docview/2557226838/se-2	Excluded	Title/Abstract	

17	V., S. (2019). Biomechanical effects of torquing on upper central incisor with thermoplastic aligner – A comparative three dimensional finite element study with and without auxillaries (Order No. 30588267). Available from ProQuest Dissertations & Theses Global. (2866351525). Retrieved from https://www.proquest.com/dissertations-theses/biomechanical-effects-torquing-on-upper-central/docview/2866351525/se-2	Excluded	Title/Abstract	
18	Harrison, J. E. (2002). Evidence based orthodontics: The way forward or an unrealistic dream?(Order No. 30832233). Available from ProQuest Dissertations & Theses Global. (2925385857). Retrieved from https://www.proquest.com/dissertations-theses/evidence-based-orthodontics-way-forward/docview/2925385857/se-2	Excluded	Title/Abstract	
19	DeLuke, M. K. (2005). Orthodontic treatment provided by general dentists and pedodontists: A national survey (Order No. 31076648). Available from ProQuest Dissertations & Theses Global. (3073204985). Retrieved from https://www.proquest.com/dissertations-theses/orthodontic-treatment-provided-general-dentists/docview/3073204985/se-2	Excluded	Title/Abstract	
20	K., M. S. (2018). A questionnaire based comparative comfort analysis of lingual and labial fixed orthodontic appliances (Order No. 30586694). Available from ProQuest Dissertations & Theses Global. (2866349721). Retrieved from https://www.proquest.com/dissertations-theses/questionnaire-based-comparative-comfort-analysis/docview/2866349721/se-2	Excluded	Title/Abstract	

21	Ahmed, S. (2019). Efficacy of single dose low level laser therapy on pain perception caused by placement of elastomeric separators and kesling separators – a comparative clinical study (Order No. 30584131). Available from ProQuest Dissertations & Theses Global. (2866350113). Retrieved from https://www.proquest.com/dissertations-theses/efficacy-single-dose-low-level-laser-therapy-on/docview/2866350113/se-2	Excluded	Title/Abstract	
22	Reiss, S. M. (2018). Biomarkers of orthodontic tooth movement with fixed appliances and vibration appliance therapy: A randomized clinical trial (Order No. 31076629). Available from ProQuest Dissertations & Theses Global. (3073204316). Retrieved from https://www.proquest.com/dissertations-theses/biomarkers-orthodontic-tooth-movement-with-fixed/docview/3073204316/se-2	Excluded	Title/Abstract	
23	Golwara, A. K. R. (2006). A comparison of the index of complexity outcome and need (icon) with the peer assessment rating (par) and the index of orthodontic treatment need (iotn) - an in vitro study (Order No. 30283180). Available from ProQuest Dissertations & Theses Global. (2786886440). Retrieved from https://www.proquest.com/dissertations-theses/comparison-index-complexity-outcome-need-icon/docview/2786886440/se-2	Excluded	Title/Abstract	
24	Siddiqui, M. A. (2018). Comparison of piezocision assisted orthodontics with conventional orthodontic treatment (Order No. 30586980). Available from ProQuest Dissertations & Theses Global. (2866350082). Retrieved from https://www.proquest.com/dissertations-theses/comparison-piezocision-assisted-orthodontics-with/docview/2866350082/se-2	Excluded	Title/Abstract	
25	Bayat, J. T. (2016). Malocclusion among adolescents: Qualitative and quantitative studies of the impact on oral health and daily life (Order No. 28426098). Available from ProQuest Dissertations & Theses Global.	Excluded	Title/Abstract	

	(2560033756). Retrieved from https://www.proquest.com/dissertations-theses/malocclusion-among-adolescents-qualitative/docview/2560033756/se-2			
26	Rao, L. R. (2018). Comparison of the rate of retraction into healed and recent extraction sites – A prospective clinical study (Order No. 30583637). Available from ProQuest Dissertations & Theses Global. (2866086509). Retrieved from https://www.proquest.com/dissertations-theses/comparison-rate-retraction-into-healed-recent/docview/2866086509/se-2	Excluded	Title/Abstract	
27	C., S. H. (2006). Morphological study of temporomandibular joint in orthodontically treated patients by using pre-treatment and post-treatment orthopantomographs (Order No. 30283297). Available from ProQuest Dissertations & Theses Global. (2787196201). Retrieved from https://www.proquest.com/dissertations-theses/morphological-study-temporomandibular-joint/docview/2787196201/se-2	Excluded	Title/Abstract	
28	I, L.,Pushpa Femina. (2019). Effects of carbonated drinks on mechanical properties of three types of thermoplastic aligner materials- an in vitro study (Order No. 30586410). Available from ProQuest Dissertations & Theses Global. (2866353404). Retrieved from https://www.proquest.com/dissertations-theses/effects-carbonated-drinks-on-mechanical/docview/2866353404/se-2	Excluded	Title/Abstract	
29	Sukhia, H. R. (2000). Oral hygiene evaluation in orthodontic practice (Order No. 28525736). Available from ProQuest Dissertations & Theses Global. (2571088264). Retrieved from https://www.proquest.com/dissertations-theses/oral-hygiene-evaluation-orthodontic-practice/docview/2571088264/se-2	Excluded	Title/Abstract	

30	Chao, H. (2019). Comparison the effectiveness of common topical fluoride application and CPP- ACP application on preventing white spot lesions in orthodontic: A systematic review (Order No. 31261359). Available from ProQuest Dissertations & Theses Global. (3098798177). Retrieved from https://www.proquest.com/dissertations-theses/comparison-effectiveness-common-topical-fluoride/docview/3098798177/se-2	Excluded	Title/Abstract	
31	Stoyanova, J. (2022). The effect of build angle on compressive strength of 3-D printed orthodontic aligners (Order No. 31628748). Available from ProQuest Dissertations & Theses Global. (3132853101). Retrieved from https://www.proquest.com/dissertations-theses/effect-build-angle-on-compressive-strength-3-d/docview/3132853101/se-2	Excluded	Title/Abstract	
32	Price, J. C. (2016). Socioeconomic position and the national health service orthodontic service (Order No. 10174799). Available from ProQuest Dissertations & Theses Global. (1827601923). Retrieved from https://www.proquest.com/dissertations-theses/socioeconomic-position-national-health-service/docview/1827601923/se-2	Excluded	Title/Abstract	
33	Touloumi, F. (2014). Differences in lip projection with and without labial denture flanges in a maxillary edentulous population – A patient centered analysis (Order No. 31081679). Available from ProQuest Dissertations & Theses Global. (3073207523). Retrieved from https://www.proquest.com/dissertations-theses/differences-lip-projection-with-without-labial/docview/3073207523/se-2	Excluded	Title/Abstract	
34	Hattarki, R. S. (2006). Effect of low dose doxycycline on the periodontal status of patients undergoing orthodontic treatment with fixed mechanotherapy (Order No. 30283162). Available from ProQuest Dissertations & Theses Global. (2786886337). Retrieved from	Excluded	Title/Abstract	

	https://www.proquest.com/dissertations-theses/effect-low-dose-doxycycline-on-periodontal-status/docview/2786886337/se-2			
35	Jose, A. (2019). Comparative evaluation of antiadherent and antibacterial properties of titanium oxide and silver coated stainless steel, niti and tma wires against lactobacillus acidophilus – an in vitro study (Order No. 30587470). Available from ProQuest Dissertations & Theses Global. (2866353540). Retrieved from https://www.proquest.com/dissertations-theses/comparative-evaluation-antiadherent-antibacterial/docview/2866353540/se-2	Excluded	Title/Abstract	
36	Gaurav, P. (2018). Efficacy of canine retraction in corticotomy with and without laser therapy - A split mouth clinical trial (Order No. 30587404). Available from ProQuest Dissertations & Theses Global. (2866353649). Retrieved from https://www.proquest.com/dissertations-theses/efficacy-canine-retraction-corticotomy-with/docview/2866353649/se-2	Excluded	Title/Abstract	
37	Raphy, P. (2018). The use of qlf-DTM (quantitative light-induced fluorescence-DigitalTM) as an oral hygiene evaluation tool to assess plaque accumulation and enamel demineralisation in pre orthodontic patients with suboptimal oral hygiene (Order No. 30676834). Available from ProQuest Dissertations & Theses Global. (2890693612). Retrieved from https://www.proquest.com/dissertations-theses/use-qlf-d-sup-tm-quantitative-light-induced/docview/2890693612/se-2	Excluded	Title/Abstract	
38	Glynn, E. J. (1996). Changes in vertical dimension in growing orthodontic patients (Order No. 31077305). Available from ProQuest Dissertations & Theses Global. (3073206673). Retrieved from https://www.proquest.com/dissertations-theses/changes-vertical-dimension-growing-orthodontic/docview/3073206673/se-2	Excluded	Title/Abstract	

39	T.P., D. (2019). Low-level laser therapy for treatment of pain associated with orthodontic elastomeric separator placement:A clinical study (Order No. 30587670). Available from ProQuest Dissertations & Theses Global. (2866350747). Retrieved from https://www.proquest.com/dissertations-theses/low-level-laser-therapy-treatment-pain-associated/docview/2866350747/se-2	Excluded	Title/Abstract	
40	Obalapura, S. K. (2019). An effective way of reducing traumatic ulcer in early phase of orthodontic treatment - A single blind randomized controlled trial (Order No. 30584892). Available from ProQuest Dissertations & Theses Global. (2866352111). Retrieved from https://www.proquest.com/dissertations-theses/effective-way-reducing-traumatic-ulcer-early/docview/2866352111/se-2	Excluded	Title/Abstract	
41	Niruban, V. (2021). The effect of smartphone motivational techniques on oral hygiene of patients undergoing orthodontic treatment with fixed orthodontic appliances (Order No. 31269218). Available from ProQuest Dissertations & Theses Global. (3098805686). Retrieved from https://www.proquest.com/dissertations-theses/effect-smartphone-motivational-techniques-on-oral/docview/3098805686/se-2	Excluded	Title/Abstract	
42	M.K, J. (2019). Comparative evaluation of antiadherent and antibacterial properties of titanium oxide and silver coated stainless steel brackets against lactobacillus acidophilus – an in vitro study (Order No. 30583697). Available from ProQuest Dissertations & Theses Global. (2866086924). Retrieved from https://www.proquest.com/dissertations-theses/comparative-evaluation-antiadherent-antibacterial/docview/2866086924/se-2	Excluded	Title/Abstract	

43	Marzban, R. B. (1999). Tnf-α levels in the gingival crevicular fluid of teeth with external apical root resorption (Order No. 31076876). Available from ProQuest Dissertations & Theses Global. (3073204342). Retrieved from https://www.proquest.com/dissertations-theses/tnf-α-levels-gingival-crevicular-fluid-teeth-with/docview/3073204342/se-2	Excluded	Title/Abstract	
44	Seth, V. (2016). Comparative evaluation of the effects of premolar extraction on vermilion height and LIP area in bimaxillary protrusion and crowding cases- A photographic study (Order No. 30276997). Available from ProQuest Dissertations & Theses Global. (2786886931). Retrieved from https://www.proquest.com/dissertations-theses/comparative-evaluation-effects-premolar/docview/2786886931/se-2	Excluded	Title/Abstract	
45	Antony, G. (2018). Correlation between objective and subjective evaluation of profile in class II div.1 patients after orthodontic treatment (Order No. 30585279). Available from ProQuest Dissertations & Theses Global. (2866351745). Retrieved from https://www.proquest.com/dissertations-theses/correlation-between-objective-subjective/docview/2866351745/se-2	Excluded	Title/Abstract	
46	Glaue, E. C. (2024). Comparative analysis of classification systems in hemifacial microsomia: Toward a standardized diagnostic and treatment framework (Order No. 31659762). Available from ProQuest Dissertations & Theses Global. (3132852214). Retrieved from https://www.proquest.com/dissertations-theses/comparative-analysis-classification-systems/docview/3132852214/se-2	Excluded	Title/Abstract	
47	P., K. K. (2018). Esthetic perception of the smile by varying mesio-distal angulation of maxillary central incisors in frontal smile (Order No. 30583802). Available from ProQuest Dissertations & Theses Global.	Excluded	Title/Abstract	

	(2866350044). Retrieved from https://www.proquest.com/dissertations-theses/esthetic-perception-smile-varying-mesio-distal/docview/2866350044/se-2			
48	Mohd.Arif Fadzillah, A. (2019). Comparing the clinical acceptability of innovatedly made transpalatal arch (TPA) from 3D reconstructed model and conventionally-made TPA (Order No. 30597970). Available from ProQuest Dissertations & Theses Global. (2877960540). Retrieved from https://www.proquest.com/dissertations-theses/comparing-clinical-acceptability-innovatedly-made/docview/2877960540/se-2	Excluded	Title/Abstract	
49	Davoody, L. R. (2015). Efficiency of piezotome-corticision assisted orthodontics in alleviating mandibular anterior crowding - A randomized controlled clinical trial (Order No. 31079773). Available from ProQuest Dissertations & Theses Global. (3073206928). Retrieved from https://www.proquest.com/dissertations-theses/efficiency-piezotome-corticision-assisted/docview/3073206928/se-2	Excluded	Title/Abstract	
50	Yaiphaba, K. (2017). A retroprospective study of long-term radiographic assessment of secondary alveolar bone grafting outcomes performed before and after canine eruption in complete unilateral and bilateral cleft lip and cleft palate involving cleft alveolus (Order No. 30585131). Available from ProQuest Dissertations & Theses Global. (2866352188). Retrieved from https://www.proquest.com/dissertations-theses/retroprospective-study-long-term-radiographic/docview/2866352188/se-2	Excluded	Title/Abstract	
51	Mohiuddin, M. I. (2009). Evaluation of the lingual orthodontic appliance: A clinical study (Order No. 30580020). Available from ProQuest Dissertations & Theses Global. (2866082650). Retrieved from	Excluded	Title/Abstract	

	https://www.proquest.com/dissertations-theses/evaluation-lingual-orthodontic-appliance-clinical/docview/2866082650/se-2			
52	, P. G.,S.V. (2019). Effect of micro-osteoperforation on the rate of tooth movement (Order No. 30587671). Available from ProQuest Dissertations & Theses Global. (2866352711). Retrieved from https://www.proquest.com/dissertations-theses/effect-micro-osteoperforation-on-rate-tooth/docview/2866352711/se-2	Excluded	Title/Abstract	
53	Rasid, S. (2018). Evaluation of modified microosteoperforation on the rate of en masse retraction. A prospective clinical study (Order No. 30584572). Available from ProQuest Dissertations & Theses Global. (2866353489). Retrieved from https://www.proquest.com/dissertations-theses/evaluation-modified-microosteoperforation-on-rate/docview/2866353489/se-2	Excluded	Title/Abstract	
54	Singh, B. (2009). Assessment of objective and subjective measures as indicators for facial esthetics -A comparative study (Order No. 30560496). Available from ProQuest Dissertations & Theses Global. (2848396792). Retrieved from https://www.proquest.com/dissertations-theses/assessment-objective-subjective-measures-as/docview/2848396792/se-2	Excluded	Title/Abstract	
55	Pretorius, N. E. (2003). The effect of arnica montana D6 on the level of discomfort in patients undergoing orthodontic treatment (Order No. 28331801). Available from ProQuest Dissertations & Theses Global. (2571088383). Retrieved from https://www.proquest.com/dissertations-theses/effect-i-arnica-montana-d6-on-level-discomfort/docview/2571088383/se-2	Excluded	Title/Abstract	

56	Maan, A. S. (2019). Assessment of interleukin-1β (IL-1β) and prostaglandin E2 (PGE2) levels in children and adults during initial orthodontic treatment (Order No. 30587743). Available from ProQuest Dissertations & Theses Global. (2866350712). Retrieved from https://www.proquest.com/dissertations-theses/assessment-interleukin-1β-il-prostaglandin-e-sub/docview/2866350712/se-2	Excluded	Title/Abstract	
57	Pathan, S. (2018). Evaluation of efficacy of low intensity diode laser and rise in GCF levels of TNF-α and IL-6 to accelerate orthodontic tooth movement (Order No. 30588090). Available from ProQuest Dissertations & Theses Global. (2866353831). Retrieved from https://www.proquest.com/dissertations-theses/evaluation-efficacy-low-intensity-diode-laser/docview/2866353831/se-2	Excluded	Title/Abstract	
58	V., A. R. (2017). The ability of panoramic radiography to predict neurosensory disturbances of inferior alveolar nerve following surgical removal of mandibular third molar (Order No. 30282437). Available from ProQuest Dissertations & Theses Global. (2786883554). Retrieved from https://www.proquest.com/dissertations-theses/ability-panoramic-radiography-predict/docview/2786883554/se-2	Excluded	Title/Abstract	
59	K., S. K. (2016). An evaluation and comparison of adherenceoof micro-organisms on different types of orthodontic brackets – an ex vivo study (Order No. 30579685). Available from ProQuest Dissertations & Theses Global. (2866082365). Retrieved from https://www.proquest.com/dissertations-theses/evaluation-comparison-adherenceoof-micro/docview/2866082365/se-2	Excluded	Title/Abstract	

60	Ramitha. (2018). Comparative evaluation of the rate and type of tooth movement in periodontally accelerated osteogenic orthodontics (paoo) and conventional orthodontic technique- a clinical study (Order No. 30584293). Available from ProQuest Dissertations & Theses Global. (2866352109). Retrieved from https://www.proquest.com/dissertations-theses/comparative-evaluation-rate-type-tooth-movement/docview/2866352109/se-2	Excluded	Title/Abstract	
61	Mzizana, N. Y. H. (2009). Soft tissue facial profile assessment of 15-20 years old tswana subjects (Order No. 30715484). Available from ProQuest Dissertations & Theses Global. (2901811076). Retrieved from https://www.proquest.com/dissertations-theses/soft-tissue-facial-profile-assessment-15-20-years/docview/2901811076/se-2	Excluded	Title/Abstract	
62	Akbari, A. (2020). The quantification of force distribution of a vibrational device for accelerating tooth movement (Order No. 30504336). Available from ProQuest Dissertations & Theses Global. (2838330920). Retrieved from https://www.proquest.com/dissertations-theses/quantification-force-distribution-vibrational/docview/2838330920/se-2	Excluded	Title/Abstract	
63	Varikkodan, M. E. (2019). Comparative evaluation of prevalence of white spot lesions between labial and lingual fixed orthodontic appliances (Order No. 30587827). Available from ProQuest Dissertations & Theses Global. (2866349945). Retrieved from https://www.proquest.com/dissertations-theses/comparative-evaluation-prevalence-white-spot/docview/2866349945/se-2	Excluded	Title/Abstract	
64	Humphreys, S. J. (2021). Management of children with molar-incisor-hypomineralisation (Order No. 28942808). Available from ProQuest Dissertations & Theses Global. (2616979990). Retrieved from	Excluded	Title/Abstract	

	https://www.proquest.com/dissertations-theses/management-children-with-molar-incisor/docview/2616979990/se-2			
65	Mohd, M. U. M. (2018). To compare the efficacy of dentrifice and mouthwash in reducing the white spot lesion which occurs during orthodontic treatment. -an invivo study (Order No. 30586391). Available from ProQuest Dissertations & Theses Global. (2866350030). Retrieved from https://www.proquest.com/dissertations-theses/compare-efficacy-dentrifice-mouthwash-reducing/docview/2866350030/se-2	Excluded	Title/Abstract	
66	Reis, S. I. M. (2020). Midpalatal suture maturation and cervical vertebrae maturation: A CBCT comparative study (Order No. 31045742). Available from ProQuest Dissertations & Theses Global. (3085971063). Retrieved from https://www.proquest.com/dissertations-theses/midpalatal-suture-maturation-cervical-vertebrae/docview/3085971063/se-2	Excluded	Title/Abstract	
67	Chanda, C. (2016). Evaluation and comparison of probiotic and fluoride mouth rinse in reduction of streptococcus mutans colonization around orthodontic brackets: An in-vivo study (Order No. 30579740). Available from ProQuest Dissertations & Theses Global. (2866084372). Retrieved from https://www.proquest.com/dissertations-theses/evaluation-comparison-probiotic-fluoride-mouth/docview/2866084372/se-2	Excluded	Title/Abstract	
68	Romana, U. (2018). Comparision of rate of en masse retraction with nickel titanium coil springs, elastomeric chains & active tiebacks and variation in root parallelism between canine and second premolar using 3 different force systems : A clinical study (Order No. 30584249). Available from ProQuest Dissertations & Theses Global. (2866351872). Retrieved from https://www.proquest.com/dissertations-	Excluded	Title/Abstract	

	theses/comparision-rate-en-masse-retraction-with-nickel/docview/2866351872/se-2			
69	C., N. K. (2009). Evaluation of the treatment effects with labial and lingual fixed appliances in patients with bimaxillary protrusion-A prospective study (Order No. 30560023). Available from ProQuest Dissertations & Theses Global. (2848392511). Retrieved from https://www.proquest.com/dissertations-theses/evaluation-treatment-effects-with-labial-lingual/docview/2848392511/se-2	Excluded	Title/Abstract	
70	Satravaha, Y. (2020). Accuracy of single tooth positions on 3D intraoral scans of patients with orthodontic multi-bracket appliances (Order No. 32217357). Available from ProQuest Dissertations & Theses Global. (3236295839). Retrieved from https://www.proquest.com/dissertations-theses/accuracy-single-tooth-positions-on-3d-intraoral/docview/3236295839/se-2	Excluded	Title/Abstract	
71	Sztabinski, B. E. (2019). 3D cone beam computed tomography as a main diagnostic tool in everyday work of an odontologist (Order No. 31264191). Available from ProQuest Dissertations & Theses Global. (3098802470). Retrieved from https://www.proquest.com/dissertations-theses/3d-cone-beam-computed-tomography-as-main/docview/3098802470/se-2	Excluded	Title/Abstract	
72	Pernet, P. (2021). Replacement of incisors bonded cantilever bridge during orthodontic treatment: A case report (Order No. 29137705). Available from ProQuest Dissertations & Theses Global. (2689294702). Retrieved from https://www.proquest.com/dissertations-	Excluded	Title/Abstract	

	theses/replacement-incisors-bonded-cantilever-bridge/docview/2689294702/se-2			
73	Vyas, I. (2018). Bite force and centric slide evaluation over 6 months post orthodontic treatment in extraction and non extraction treated patients using T-scan: A prospective study (Order No. 30587632). Available from ProQuest Dissertations & Theses Global. (2866352886). Retrieved from https://www.proquest.com/dissertations-theses/bite-force-centric-slide-evaluation-over-6-months/docview/2866352886/se-2	Excluded	Title/Abstract	
74	Brahmachari, P. (2018). Colour stability of different esthetic brackets under the influence of in vitro dietary media- A spectrophotometric analysis (Order No. 30586809). Available from ProQuest Dissertations & Theses Global. (2866352654). Retrieved from https://www.proquest.com/dissertations-theses/colour-stability-different-esthetic-brackets/docview/2866352654/se-2	Excluded	Title/Abstract	
75	Varghese, A. (2006). Comparison of dimensional changes in the dental arch after extraction and non-extraction treatment (Order No. 30282919). Available from ProQuest Dissertations & Theses Global. (2786883451). Retrieved from https://www.proquest.com/dissertations-theses/comparison-dimensional-changes-dental-arch-after/docview/2786883451/se-2	Excluded	Title/Abstract	
76	Barmpoutis, P. (2006). Evaluation of soft tissue in class III cases treated by different types of face masks (Order No. 28593368). Available from ProQuest Dissertations & Theses Global. (2561545018). Retrieved from https://www.proquest.com/dissertations-theses/evaluation-soft-tissue-class-iii-cases-treated/docview/2561545018/se-2	Excluded	Title/Abstract	

77	Kalinaitė, M. (2020). Angle II klasės anomalijų gydymo metodo įtaka pacientų veido pokyčiams (Order No. 31263722). Available from ProQuest Dissertations & Theses Global. (3098801499). Retrieved from https://www.proquest.com/dissertations-theses/angle-ii-klasės-anomalijų-gydymo-metodo-įtaka/docview/3098801499/se-2	Excluded	Title/Abstract	
78	Dzulkhaini, A. (2012). High le fort I surgically assisted rapid maxillary expansion (Order No. 28593008). Available from ProQuest Dissertations & Theses Global. (2561521683). Retrieved from https://www.proquest.com/dissertations-theses/high-le-fort-i-surgically-assisted-rapid/docview/2561521683/se-2	Excluded	Title/Abstract	
79	Merhi, A. M. (1997). Cephalometric evaluation of stability of jasper jumper treatment in class II cases (Order No. 28539698). Available from ProQuest Dissertations & Theses Global. (2561521673). Retrieved from https://www.proquest.com/dissertations-theses/cephalometric-evaluation-stability-jasper-jumper/docview/2561521673/se-2	Excluded	Title/Abstract	
80	Kishor, S. (2005). A comparative study of chlorhexidine and phenolic essential oil mouth rinse on the periodontal and gingival health of patients undergoing fixed orthodontic treatment (Order No. 30282912). Available from ProQuest Dissertations & Theses Global. (2786886293). Retrieved from https://www.proquest.com/dissertations-theses/comparative-study-chlorhexidine-phenolic/docview/2786886293/se-2	Excluded	Title/Abstract	
81	Rathi, K.,L. (2018). The effect of platelet rich fibrin and selective alveolar decortication on orthodontic tooth movement – A prospective clinical study (Order No. 30587793). Available from ProQuest Dissertations & Theses Global. (2866351850). Retrieved from	Excluded	Title/Abstract	

	https://www.proquest.com/dissertations-theses/effect-platelet-rich-fibrin-selective-alveolar/docview/2866351850/se-2			
82	Lee, Y. (2021). Association between mandibular incisor to mandibular plane angle (IMPA) and periodontal health status based on loss of clinical attachment level (CAL): A cross-sectional study (Order No. 30669915). Available from ProQuest Dissertations & Theses Global. (2890693526). Retrieved from https://www.proquest.com/dissertations-theses/association-between-mandibular-incisor-plane/docview/2890693526/se-2	Excluded	Title/Abstract	
83	Brehove, J. M. (2021). Effects of cleaning agents on the properties of two different thermoplastic retainer materials (Order No. 28648174). Available from ProQuest Dissertations & Theses Global. (2566019972). Retrieved from https://www.proquest.com/dissertations-theses/effects-cleaning-agents-on-properties-two/docview/2566019972/se-2	Excluded	Title/Abstract	
84	V, M.,Abdul Rasheed. (2018). Evaluation of the rate of individual canine retraction and gingival crevicular fluid levels of interleaukin-1β with micro-osteo perforations – A split mouth study (Order No. 30588028). Available from ProQuest Dissertations & Theses Global. (2866351658). Retrieved from https://www.proquest.com/dissertations-theses/evaluation-rate-individual-canine-retraction/docview/2866351658/se-2	Excluded	Title/Abstract	
85	Goonewardene, R. (2015). Stability of a surgery first approach on mandibular prognathism (Order No. 31079995). Available from ProQuest Dissertations & Theses Global. (3073205776). Retrieved from https://www.proquest.com/dissertations-theses/stability-surgery-first-approach-on-mandibular/docview/3073205776/se-2	Excluded	Title/Abstract	

86	Yuming, F. (2021). The influence of extraction and non-extraction treatment on class II patients' soft tissue profile: A literature review (Order No. 31271419). Available from ProQuest Dissertations & Theses Global. (3098808318). Retrieved from https://www.proquest.com/dissertations-theses/influence-extraction-non-treatment-on-class-ii/docview/3098808318/se-2	Excluded	Title/Abstract	
87	Ramesh, N. (2018). External apical root resorption following orthodontic treatment in maxillary and mandibular incisors-A study of contributing factors (Order No. 30585245). Available from ProQuest Dissertations & Theses Global. (2866350368). Retrieved from https://www.proquest.com/dissertations-theses/external-apical-root-resorption-following/docview/2866350368/se-2	Excluded	Title/Abstract	
88	Abelak, D. D. (2017). Treatment provision by general dentists in mauritius, with particular reference to prosthodontic treatment (Order No. 31796538). Available from ProQuest Dissertations & Theses Global. (3161868617). Retrieved from https://www.proquest.com/dissertations-theses/treatment-provision-general-dentists-mauritius/docview/3161868617/se-2	Excluded	Title/Abstract	
89	Sunny, J. (2019). Comparative analysis of bone remodelling markers in anterior and posterior segment after micro-osteoperforations : GCF study (Order No. 30584427). Available from ProQuest Dissertations & Theses Global. (2866350442). Retrieved from https://www.proquest.com/dissertations-theses/comparative-analysis-bone-remodelling-markers/docview/2866350442/se-2	Excluded	Title/Abstract	
90	Laher, A. (2000). Effectiveness of manual toothbrushes in patients with fixed orthodontic appliances (Order No. 30715390). Available from ProQuest Dissertations & Theses Global. (2901811869). Retrieved from	Excluded	Title/Abstract	

	https://www.proquest.com/dissertations-theses/effectiveness-manual-toothbrushes-patients-with/docview/2901811869/se-2			
91	Kaler, J. (2022). Comparison of vacuum-formed retainer protocols using 3 shape orthoanalyzer (Order No. 31628731). Available from ProQuest Dissertations & Theses Global. (3132851309). Retrieved from https://www.proquest.com/dissertations-theses/comparison-vacuum-formed-retainer-protocols-using/docview/3132851309/se-2	Excluded	Title/Abstract	
92	Selamet, B. U. (2009). Clinical comparison of self-ligating brackets versus conventional brackets (Order No. 28558549). Available from ProQuest Dissertations & Theses Global. (2561545986). Retrieved from https://www.proquest.com/dissertations-theses/clinical-comparison-self-ligating-brackets-versus/docview/2561545986/se-2	Excluded	Title/Abstract	
93	O'Hea, C. M. (1999). A prospective investigation of maxillary central incisor root resorption incident to orthodontic therapy (Order No. 31077838). Available from ProQuest Dissertations & Theses Global. (3073205645). Retrieved from https://www.proquest.com/dissertations-theses/prospective-investigation-maxillary-central/docview/3073205645/se-2	Excluded	Title/Abstract	
94	Dzingle, J. N. (2017). Facial morphology as a determinant of anchorage control (Order No. 31079492). Available from ProQuest Dissertations & Theses Global. (3073205602). Retrieved from https://www.proquest.com/dissertations-theses/facial-morphology-as-determinant-anchorage/docview/3073205602/se-2	Excluded	Title/Abstract	

95	Yarlagadda, S. K. B. (2009). A survey of observable deviation of facial and anterior tooth midline and comparison between men and women subjects in bangalore east population (Order No. 30560492). Available from ProQuest Dissertations & Theses Global. (2848396426). Retrieved from https://www.proquest.com/dissertations-theses/survey-observable-deviation-facial-anterior-tooth/docview/2848396426/se-2	Excluded	Title/Abstract	
96	Ansari, T. A. (2009). Clinical evaluation of retraction with ostectomy in the extraction site using the MBT appliance (Order No. 30560481). Available from ProQuest Dissertations & Theses Global. (2848394457). Retrieved from https://www.proquest.com/dissertations-theses/clinical-evaluation-retraction-with-ostectomy/docview/2848394457/se-2	Excluded	Title/Abstract	
97	Maulik, C. (2005). A dynamic smile analysis in young adults (Order No. 31077946). Available from ProQuest Dissertations & Theses Global. (3073205438). Retrieved from https://www.proquest.com/dissertations-theses/dynamic-smile-analysis-young-adults/docview/3073205438/se-2	Excluded	Title/Abstract	
98	George, D. E. (2018). A comparative evaluation of static and dynamic lip-tooth characteristics during speech and smile (Order No. 30586038). Available from ProQuest Dissertations & Theses Global. (2866350974). Retrieved from https://www.proquest.com/dissertations-theses/comparative-evaluation-static-dynamic-lip-tooth/docview/2866350974/se-2	Excluded	Title/Abstract	
99	Vagger, R.,M. (2016). Maxillary incisal inclination and its association with pre maxillary vertical height, depth and maxillary jaw base rotation – A cephalometric study (Order No. 30577235). Available from ProQuest Dissertations & Theses Global. (2848781535). Retrieved from https://www.proquest.com/dissertations-theses/maxillary-incisal-inclination-association-with/docview/2848781535/se-2	Excluded	Title/Abstract	

100	Kumar, N. (2016). Comparing the treatment of white spot lesions using cpp - acp cream V/S microabrasion - A quantative assessment (Order No. 30276852). Available from ProQuest Dissertations & Theses Global. (2786886699). Retrieved from https://www.proquest.com/dissertations-theses/comparing-treatment-white-spot-lesions-using-cpp/docview/2786886699/se-2	Excluded	Title/Abstract	
101	Ahad, M. (2009). Evaluation of micro implants for absolute anchorage: A clinical study (Order No. 30559290). Available from ProQuest Dissertations & Theses Global. (2848394958). Retrieved from https://www.proquest.com/dissertations-theses/evaluation-micro-implants-absolute-anchorage/docview/2848394958/se-2	Excluded	Title/Abstract	
102	Batra, K. (2009). Clinical evaluation of efficiency and anchorage loss during canine retraction using micro-implants as compared to conventional first molar anchorage (Order No. 30283181). Available from ProQuest Dissertations & Theses Global. (2786882953). Retrieved from https://www.proquest.com/dissertations-theses/clinical-evaluation-efficiency-anchorage-loss/docview/2786882953/se-2	Excluded	Title/Abstract	
103	Seres, L. (2011). Modern therapeutic approaches in the correction of dentofacial and maxillofacial deformities (Order No. 30756415). Available from ProQuest Dissertations & Theses Global. (2901816826). Retrieved from https://www.proquest.com/dissertations-theses/modern-therapeutic-approaches-correction/docview/2901816826/se-2	Excluded	Title/Abstract	
104	Peterson, P. (2022). A multidisciplinary long-term evaluation of patients born with unilateral cleft lip and palate treated with different surgical protocols (Order No. 30254622). Available from ProQuest Dissertations & Theses Global. (2778644566). Retrieved from	Excluded	Title/Abstract	

	https://www.proquest.com/dissertations-theses/multidisciplinary-long-term-evaluation-patients/docview/2778644566/se-2			
105	A., S. R. (2018). Comparing the rate of relapse with damon Q brackets and conventional brackets in extraction cases (Order No. 30586913). Available from ProQuest Dissertations & Theses Global. (2866350284). Retrieved from https://www.proquest.com/dissertations-theses/comparing-rate-relapse-with-damon-q-brackets/docview/2866350284/se-2	Excluded	Title/Abstract	
106	Parthdev, R. (2016). Identification of an ideal site for placement of mini screw implants in paramedian palate: A computed tomographic study (Order No. 30579285). Available from ProQuest Dissertations & Theses Global. (2866082130). Retrieved from https://www.proquest.com/dissertations-theses/identification-ideal-site-placement-mini-screw/docview/2866082130/se-2	Excluded	Title/Abstract	
107	Afreen, S. (2018). A prospective invivo study to evaluate the effect of local application of PGE1 patches on the rate of canine retraction (Order No. 30587974). Available from ProQuest Dissertations & Theses Global. (2866352660). Retrieved from https://www.proquest.com/dissertations-theses/prospective-invivo-study-evaluate-effect-local/docview/2866352660/se-2	Excluded	Title/Abstract	
108	Sağır, S. (2010). Skeletal and dentoal veolar effects of forsus™ fatigue resistant device in class II division 1 patients (Order No. 28523467). Available from ProQuest Dissertations & Theses Global. (2568605561). Retrieved from https://www.proquest.com/dissertations-theses/skeletal-dentoal-veolar-effects-forsus™-fatigue/docview/2568605561/se-2	Excluded	Title/Abstract	

109	de Sequeira Fernandes, Natascha Lília Margarida. (2021). The influence of le fort 1 maxillary advancement osteotomy on nasal morphology and aesthetics (Order No. 31617984). Available from ProQuest Dissertations & Theses Global. (3122672012). Retrieved from https://www.proquest.com/dissertations-theses/influence-le-fort-1-maxillary-advancement/docview/3122672012/se-2	Excluded	Title/Abstract	
110	Mohammed, W. Z. P. (2016). Comparison of cephalometric norms between iranian population in mysore and caucasian adults using antero-posterior and vertical dimensions (Order No. 30276782). Available from ProQuest Dissertations & Theses Global. (2786897865). Retrieved from https://www.proquest.com/dissertations-theses/comparison-cephalometric-norms-between-iranian/docview/2786897865/se-2	Excluded	Title/Abstract	
111	Drummond, R. J. (2003). Orthodontic status and treatment need of 12-year-old children in south africa : An epidemiological study using the dental aesthetic index (Order No. 30700280). Available from ProQuest Dissertations & Theses Global. (2890697825). Retrieved from https://www.proquest.com/dissertations-theses/orthodontic-status-treatment-need-12-year-old/docview/2890697825/se-2	Excluded	Title/Abstract	
112	N., H. K. (2019). Evaluation of the shear bond strength of three types of retainer wires bonded with a composite adhesive- an in vitro study (Order No. 30584117). Available from ProQuest Dissertations & Theses Global. (2866349220). Retrieved from https://www.proquest.com/dissertations-theses/evaluation-shear-bond-strength-three-types/docview/2866349220/se-2	Excluded	Title/Abstract	
113	Tom, A. (2019). Comparative evaluation of arch dimensional changes between damon Q and MBT brackets: An in-vitro study (Order No. 30587500). Available from ProQuest Dissertations & Theses Global.	Excluded	Title/Abstract	

	(2866352512). Retrieved from https://www.proquest.com/dissertations-theses/comparative-evaluation-arch-dimensional-changes/docview/2866352512/se-2			
114	Yoshang, J. (2020). Biomonitoring of genotoxic effect in children subjucted to dental radiographs during pulpectomy procedure - BMCyt assay (Order No. 30578797). Available from ProQuest Dissertations & Theses Global. (2866084349). Retrieved from https://www.proquest.com/dissertations-theses/biomonitoring-genotoxic-effect-children-subjucted/docview/2866084349/se-2	Excluded	Title/Abstract	
115	Kachroo, Y. M. (2006). Establishing soft tissue profile norms for lingayat college going students of belgaum city using angular photogrammetric analysis (Order No. 30282883). Available from ProQuest Dissertations & Theses Global. (2786886603). Retrieved from https://www.proquest.com/dissertations-theses/establishing-soft-tissue-profile-norms-lingayat/docview/2786886603/se-2	Excluded	Title/Abstract	
116	Arumughan, S. (2018). A comparison of the rate of retraction with low level laser therapy and conventional retraction technique – A clinical study (Order No. 30587330). Available from ProQuest Dissertations & Theses Global. (2866354049). Retrieved from https://www.proquest.com/dissertations-theses/comparison-rate-retraction-with-low-level-laser/docview/2866354049/se-2	Excluded	Title/Abstract	
117	Ganeiber, T. (2013). Cbct evaluation of the relationship between lower central incisor inclination and mandibular symphysis among different subjects with normal and long face pattern (Order No. 28528577). Available from ProQuest Dissertations & Theses Global. (2570011625). Retrieved from https://www.proquest.com/dissertations-theses/cbct-evaluation-relationship-between-lower/docview/2570011625/se-2	Excluded	Title/Abstract	

118	Narayan, A. (2007). Three piece intrusion arch (Order No. 30276561). Available from ProQuest Dissertations & Theses Global. (2786882902). Retrieved from https://www.proquest.com/dissertations-theses/three-piece-intrusion-arch/docview/2786882902/se-2	Excluded	Title/Abstract	
119	Rengarajan, S. (2018). A comparative evaluation of morphological patterns of lip prints between class II growing individuals and adults and its use as a predictor of the malocclusion – A cross sectional study (Order No. 30583673). Available from ProQuest Dissertations & Theses Global. (2866087145). Retrieved from https://www.proquest.com/dissertations-theses/comparative-evaluation-morphological-patterns-lip/docview/2866087145/se-2	Excluded	Title/Abstract	
120	Sjögren, A. (2011). Influence of deciduous canine extractions on incisor alignment, dental arch dimensions and dental fear (Order No. 28422238). Available from ProQuest Dissertations & Theses Global. (2570012591). Retrieved from https://www.proquest.com/dissertations-theses/influence-deciduous-canine-extractions-on-incisor/docview/2570012591/se-2	Excluded	Title/Abstract	
121	Gaikwad, S. (2009). Influence of smile ARC and buccal corridors on facial attractiveness (Order No. 30560503). Available from ProQuest Dissertations & Theses Global. (2848396891). Retrieved from https://www.proquest.com/dissertations-theses/influence-smile-arc-buccal-corridors-on-facial/docview/2848396891/se-2	Excluded	Title/Abstract	
122	Chakravarthy, K. (2006). Awareness and attitudes concerning mouthguard use in 7-12 years old children at davangere skating rink (Order No. 30559562). Available from ProQuest Dissertations & Theses Global. (2848396607). Retrieved from	Excluded	Title/Abstract	

	https://www.proquest.com/dissertations-theses/awareness-attitudes-concerning-mouthguard-use-7/docview/2848396607/se-2			
123	Jose, A. R. (2019). Quantitative assessment of root resorption in tad aided anchorage with and without rap- A CBCT study on enmasse retraction cases (Order No. 30583788). Available from ProQuest Dissertations & Theses Global. (2866352961). Retrieved from https://www.proquest.com/dissertations-theses/quantitative-assessment-root-resorption-tad-aided/docview/2866352961/se-2	Excluded	Title/Abstract	
124	Joseph, R. V. (2016). A comparative study to determine the effect of micro-osteoperforations on the rate of canine retraction". an in vivo study (Order No. 30578583). Available from ProQuest Dissertations & Theses Global. (2866084126). Retrieved from https://www.proquest.com/dissertations-theses/comparative-study-determine-effect-micro/docview/2866084126/se-2	Excluded	Title/Abstract	
125	Kalsi, S. (2018). Assessment of plaque retention on different orthodontic brackets using real time polymerase chain reaction- A comparitive microbiological study (Order No. 30585258). Available from ProQuest Dissertations & Theses Global. (2866350589). Retrieved from https://www.proquest.com/dissertations-theses/assessment-plaque-retention-on-different/docview/2866350589/se-2	Excluded	Title/Abstract	
126	Umesh, K. W. A. (2018). Morphometric analyzation of the interproximal embrasure area": A clinico-digital study (Order No. 30587896). Available from ProQuest Dissertations & Theses Global. (2866350102). Retrieved from https://www.proquest.com/dissertations-theses/morphometric-analyzation-interproximal-embrasure/docview/2866350102/se-2	Excluded	Title/Abstract	

127	Panou, E. (2011). Investigation of the dimensional changes of maxillary sinuses and pharyngeal airway in class III patients undergoing bimaxillary orthognathic surgery (Order No. 28594633). Available from ProQuest Dissertations & Theses Global. (2561521677). Retrieved from https://www.proquest.com/dissertations-theses/investigation-dimensional-changes-maxillary/docview/2561521677/se-2	Excluded	Title/Abstract	
128	Jeedi, A.,B. (2016). Assessment of root topography, alveolar bone level and thickness of maxillary incisors following intrusion and retraction with three piece base arch using cone beam computed tomography - A prospective clinical study (Order No. 30579161). Available from ProQuest Dissertations & Theses Global. (2866082085). Retrieved from https://www.proquest.com/dissertations-theses/assessment-root-topography-alveolar-bone-level/docview/2866082085/se-2	Excluded	Title/Abstract	
129	Kaka, M. (2016). Longitudinal assessment of the Z-angle (Order No. 31795869). Available from ProQuest Dissertations & Theses Global. (3161868567). Retrieved from https://www.proquest.com/dissertations-theses/longitudinal-assessment-z-angle/docview/3161868567/se-2	Excluded	Title/Abstract	
130	Jesheen, N. (2019). A comparison of two non -surgical treatment modalities for the treatment of vertical dysplasias – A CBCT study (Order No. 30585103). Available from ProQuest Dissertations & Theses Global. (2866350227). Retrieved from https://www.proquest.com/dissertations-theses/comparison-two-non-surgical-treatment-modalities/docview/2866350227/se-2	Excluded	Title/Abstract	
131	Librizzi, Z. T. (2015). Comparison of the effects of three surgical techniques on the rate of orthodontic tooth movement in a rat model (Order No. 31079881). Available from ProQuest Dissertations & Theses Global. (3073205577). Retrieved from	Excluded	Title/Abstract	

	https://www.proquest.com/dissertations-theses/comparison-effects-three-surgical-techniques-on/docview/3073205577/se-2			
132	Kalbası, P. (2006). Radiographic evaluation of apical root resorption of maxillary first molars due to the intrusion effect of jasper jumper (Order No. 28563509). Available from ProQuest Dissertations & Theses Global. (2561471416). Retrieved from https://www.proquest.com/dissertations-theses/radiographic-evaluation-apical-root-resorption/docview/2561471416/se-2	Excluded	Title/Abstract	
133	Sreekumar, C.,A. (2019). Changes in skeletal and soft tissues following treatment in class I bimaxillary protrusion- A cephalomertic study (Order No. 30587809). Available from ProQuest Dissertations & Theses Global. (2866350387). Retrieved from https://www.proquest.com/dissertations-theses/changes-skeletal-soft-tissues-following-treatment/docview/2866350387/se-2	Excluded	Title/Abstract	
134	M., P. K. I. (2016). Assessment of root topography alveolar bone level and thickness of maxillary incisors following Rickett's intrusion and retraction arch by using cone beam computed tomography "-A prospective clinical study (Order No. 30277041). Available from ProQuest Dissertations & Theses Global. (2786887852). Retrieved from https://www.proquest.com/dissertations-theses/assessment-root-topography-alveolar-bone-level/docview/2786887852/se-2	Excluded	Title/Abstract	
135	Velentza, M. (2014). Three-dimensional assessment of condyle and glenoid fossa changes after treatment with the twin block and herbst appliances (Order No. 28538477). Available from ProQuest Dissertations & Theses Global. (2561545989). Retrieved from	Excluded	Title/Abstract	

	https://www.proquest.com/dissertations-theses/three-dimensional-assessment-condyle-glenoid/docview/2561545989/se-2			
136	Chaitanya, P. V. K. (2018). Evaluation of the effects of deep bite correction with class II elastics, reverse curve of spee and anterior bite plane on the skeletal and dental parameters -A prospective in-vivo study (Order No. 30587770). Available from ProQuest Dissertations & Theses Global. (2866350719). Retrieved from https://www.proquest.com/dissertations-theses/evaluation-effects-deep-bite-correction-with/docview/2866350719/se-2	Excluded	Title/Abstract	
137	Mohamed, N. A. (2007). The soft tissue changes after class ii treatment with bionator and jasper jumper appliances (Order No. 28513275). Available from ProQuest Dissertations & Theses Global. (2570999776). Retrieved from https://www.proquest.com/dissertations-theses/soft-tissue-changes-after-class-ii-treatment-with/docview/2570999776/se-2	Excluded	Title/Abstract	
138	Pharande, A. J. (2006). Establishing hard and soft tissue norms for orthognathic surgery patients in indianpopulation: A cephalometric and photographic study (Order No. 30282805). Available from ProQuest Dissertations & Theses Global. (2786883184). Retrieved from https://www.proquest.com/dissertations-theses/establishing-hard-soft-tissue-norms-orthognathic/docview/2786883184/se-2	Excluded	Title/Abstract	
139	Swoboda, N. A. (2013). An evaluation of the frankfort mandibular plane angle bisector (FMAB) wits appraisal in the assessment of anteroposterior jaw relationships (Order No. 29241909). Available from ProQuest Dissertations & Theses Global. (2701130682). Retrieved from https://www.proquest.com/dissertations-theses/evaluation-frankfort-mandibular-plane-angle/docview/2701130682/se-2	Excluded	Title/Abstract	

140	Suliman, M. F. (2014). Can an orthopantomograph be used as an indicator of vertical jaw relations?(Order No. 30707365). Available from ProQuest Dissertations & Theses Global. (2901483984). Retrieved from https://www.proquest.com/dissertations-theses/can-orthopantomograph-be-used-as-indicator/docview/2901483984/se-2	Excluded	Title/Abstract	
141	Amitha, K. (2017). Clinical assessment of the effects of low level laser therapy on coronally advanced flap procedure in the management of isolated gingival recession (Order No. 30278539). Available from ProQuest Dissertations & Theses Global. (2786886511). Retrieved from https://www.proquest.com/dissertations-theses/clinical-assessment-effects-low-level-laser/docview/2786886511/se-2	Excluded	Title/Abstract	
142	S., A. (2019). Evaluation of enamel remineralizing potential of CPP ACPF, amine fluoride and bioactive glass toothpastes with nanoindentation (Order No. 30588332). Available from ProQuest Dissertations & Theses Global. (2866350857). Retrieved from https://www.proquest.com/dissertations-theses/evaluation-enamel-remineralizing-potential-cpp/docview/2866350857/se-2	Excluded	Title/Abstract	
143	McElroy, S. D. (1998). Bjork predictors of mandibular rotation and their relationship to anterior open bite treatment (Order No. 31077130). Available from ProQuest Dissertations & Theses Global. (3073206353). Retrieved from https://www.proquest.com/dissertations-theses/bjork-predictors-mandibular-rotation-their/docview/3073206353/se-2	Excluded	Title/Abstract	
144	Nayeem, M. (2018). Comparison of hard and soft tissue changes in skeletal class ii individuals treated with one phase and two phase treatment protocols: A retrospective cephalometric study (Order No. 30586725). Available from ProQuest Dissertations & Theses Global. (2866353004). Retrieved from https://www.proquest.com/dissertations-	Excluded	Title/Abstract	

	theses/comparison-hard-soft-tissue-changes-skeletal/docview/2866353004/se-2			
145	Al-Masry, M. (2004). Radiographic evaluation of apical root resorption of maxillary first molars after intrusion using zygomatic skeletal anchorage (Order No. 28519416). Available from ProQuest Dissertations & Theses Global. (2571088421). Retrieved from https://www.proquest.com/dissertations-theses/radiographic-evaluation-apical-root-resorption/docview/2571088421/se-2	Excluded	Title/Abstract	
146	Kuriakose, B. M. (2016). Comparison of corrosion resistance of nickel titanium and beta titanium wires in chloride and fluoride environments – an invitro study (Order No. 30576317). Available from ProQuest Dissertations & Theses Global. (2848767210). Retrieved from https://www.proquest.com/dissertations-theses/comparison-corrosion-resistance-nickel-titanium/docview/2848767210/se-2	Excluded	Title/Abstract	
147	Savant, S. (2016). Evaluation of short term effect on periodontal health by fixed orthodontic appliance (Order No. 30277049). Available from ProQuest Dissertations & Theses Global. (2786887500). Retrieved from https://www.proquest.com/dissertations-theses/evaluation-short-term-effect-on-periodontal/docview/2786887500/se-2	Excluded	Title/Abstract	
148	M., V. (2018). Photographic soft tissue profile analysis of adolescents at 12-15 years of age with dental class I and class II in davangere population – A comparative study (Order No. 30585234). Available from ProQuest Dissertations & Theses Global. (2866352430). Retrieved from https://www.proquest.com/dissertations-theses/photographic-soft-tissue-profile-analysis/docview/2866352430/se-2	Excluded	Title/Abstract	

149	Hussain, K. S. (2019). Photocatalytic effect of titanium oxide against lactobacillus acidophilus and streptococcus mutans for its antiadherent and antibacterial properties on stainless steel bracket, an in vitro study (Order No. 30586240). Available from ProQuest Dissertations & Theses Global. (2866352056). Retrieved from https://www.proquest.com/dissertations-theses/photocatalytic-effect-titanium-oxide-against/docview/2866352056/se-2	Excluded	Title/Abstract	
150	Röhr, J.,Roman Maximilian. (2014). Häufigkeit und ausmaß von wurzelspitzenresorptionen nach festsitzender therapie mit multibracketapparatur - eine retrospektive studie (Order No. 28470314). Available from ProQuest Dissertations & Theses Global. (2572634169). Retrieved from https://www.proquest.com/dissertations-theses/häufigkeit-und-ausmaß-von/docview/2572634169/se-2	Excluded	Title/Abstract	
151	S., V. (2019). Assessment of fracture resistance of commonly used self-drilling orthodontic mini-implants of various diameters: An in-vitro study (Order No. 30584954). Available from ProQuest Dissertations & Theses Global. (2866349846). Retrieved from https://www.proquest.com/dissertations-theses/assessment-fracture-resistance-commonly-used-self/docview/2866349846/se-2	Excluded	Title/Abstract	
152	Röhr, J.,Roman Maximilian. (2014). Prevalence and degree of root resorption after treatment with fixed orthodontic appliance - A retrospective study (Order No. 31202653). Available from ProQuest Dissertations & Theses Global. (3073247826). Retrieved from https://www.proquest.com/dissertations-theses/prevalence-degree-root-resorption-after-treatment/docview/3073247826/se-2	Excluded	Title/Abstract	

153	Malagan, M. A. (2010). A comparision of the efficacy of four different types of orthodontic separators (Order No. 30276766). Available from ProQuest Dissertations & Theses Global. (2786882967). Retrieved from https://www.proquest.com/dissertations-theses/comparision-efficacy-four-different-types/docview/2786882967/se-2	Excluded	Title/Abstract	
154	M.P., S. (2019). A tomographic assessment of alveolar bone after en masse retraction- an in-vivo study (Order No. 30584014). Available from ProQuest Dissertations & Theses Global. (2866352958). Retrieved from https://www.proquest.com/dissertations-theses/tomographic-assessment-alveolar-bone-after-en/docview/2866352958/se-2	Excluded	Title/Abstract	
155	Nileshkumar, M. D. (2019). Enamel deproteinization and its effect on ETCH pattern and shear bond strength (Order No. 30588323). Available from ProQuest Dissertations & Theses Global. (2866349747). Retrieved from https://www.proquest.com/dissertations-theses/enamel-deproteinization-effect-on-etch-pattern/docview/2866349747/se-2	Excluded	Title/Abstract	
156	T., L. (2018). A comparative cephalometric study of changes in chin position in class II division 1 patients treated with twin block and fixed functional appliances (Order No. 30585767). Available from ProQuest Dissertations & Theses Global. (2866352614). Retrieved from https://www.proquest.com/dissertations-theses/comparative-cephalometric-study-changes-chin/docview/2866352614/se-2	Excluded	Title/Abstract	
157	Papadopoulos, C. (2016). Nasal morphology as a predictor of craniofacial growth direction (Order No. 29244778). Available from ProQuest Dissertations & Theses Global. (2714866945). Retrieved from https://www.proquest.com/dissertations-theses/nasal-morphology-as-predictor-craniofacial-growth/docview/2714866945/se-2	Excluded	Title/Abstract	

158	Mangan, J. T. (2018). Reliability of cervical vertebrae maturation (CVM) staging method using full versus cropped lateral cephalograms (Order No. 13831689). Available from ProQuest Dissertations & Theses Global. (2164135359). Retrieved from https://www.proquest.com/dissertations-theses/reliability-cervical-vertebrae-maturation-cvm/docview/2164135359/se-2	Excluded	Title/Abstract	
159	, C.,K.R. (2013). Evaluation and comparsion of accuracy and precision of root length measurement using computed tomography and periapical radiograph in sliding and loop mechanics (Order No. 30583504). Available from ProQuest Dissertations & Theses Global. (2866086629). Retrieved from https://www.proquest.com/dissertations-theses/evaluation-comparsion-accuracy-precision-root/docview/2866086629/se-2	Excluded	Title/Abstract	
160	Palacios, J. (2015). Impact of bracket design and oral hygiene maintenance on halitosis in the orthodontic patient (Order No. 31081614). Available from ProQuest Dissertations & Theses Global. (3073208488). Retrieved from https://www.proquest.com/dissertations-theses/impact-bracket-design-oral-hygiene-maintenance-on/docview/3073208488/se-2	Excluded	Title/Abstract	
161	Yasas, B.,V. (2006). The effectiveness of canine lacebacks in roth vs. MBT systems: A randomised clinical trial (Order No. 30582291). Available from ProQuest Dissertations & Theses Global. (2866084751). Retrieved from https://www.proquest.com/dissertations-theses/effectiveness-canine-lacebacks-roth-vs-mbt/docview/2866084751/se-2	Excluded	Title/Abstract	
162	K., J. (2018). A prospective clinical study to evaluate the amount of mandibular autorotation and change in condylar position, after leveling aligning of maxillary anteriors in cases with class II DIV2 malocclusion (Order No. 30586009). Available from ProQuest	Excluded	Title/Abstract	

	Dissertations & Theses Global. (2866349373). Retrieved from https://www.proquest.com/dissertations-theses/prospective-clinical-study-evaluate-amount/docview/2866349373/se-2			
163	Y., K. (2018). Comparison of the rate of canine retraction and anchorage loss between esthetic, semiesthetic and metal self-ligating brackets using sliding mechanics – A clinical study (Order No. 30584836). Available from ProQuest Dissertations & Theses Global. (2866352146). Retrieved from https://www.proquest.com/dissertations-theses/comparison-rate-canine-retraction-anchorage-loss/docview/2866352146/se-2	Excluded	Title/Abstract	
164	Upadhyay, M. (2006). Evaluation of titanium mini implants as a rigid source of intra oral anchorage for orthodontic space closure a clinical study (Order No. 30282937). Available from ProQuest Dissertations & Theses Global. (2786887130). Retrieved from https://www.proquest.com/dissertations-theses/evaluation-titanium-mini-implants-as-rigid-source/docview/2786887130/se-2	Excluded	Title/Abstract	
165	Kumari, S. (2016). Correlation of the SNP's of FGFR1 and FGF10 with non-syndromic cleft lip with or without cleft palate (Order No. 30584567). Available from ProQuest Dissertations & Theses Global. (2866351590). Retrieved from https://www.proquest.com/dissertations-theses/correlation-snp-s-fgfr1-fgf10-with-non-syndromic/docview/2866351590/se-2	Excluded	Title/Abstract	
166	AlRushaid, S. S. (2014). Three-dimensional evaluation of root and alveolar ridge width of maxillary lateral incisors in patients with unilateral agenesis (Order No. 31080919). Available from ProQuest Dissertations & Theses Global. (3073208377). Retrieved from	Excluded	Title/Abstract	

	https://www.proquest.com/dissertations-theses/three-dimensional-evaluation-root-alveolar-ridge/docview/3073208377/se-2			
167	P., F. (2009). Evaluation of efficacy of MBT versatile +Appliance during space closure (Order No. 30579403). Available from ProQuest Dissertations & Theses Global. (2866082233). Retrieved from https://www.proquest.com/dissertations-theses/evaluation-efficacy-mbt-versatile-sup-appliance/docview/2866082233/se-2	Excluded	Title/Abstract	
168	Rangan, M. (2020). Evaluation of treatment outcomes in the graduate orthodontic clinic at sefako makgatho university using the american board of orthodontics cast and radiographic examination (Order No. 31799841). Available from ProQuest Dissertations & Theses Global. (3161872070). Retrieved from https://www.proquest.com/dissertations-theses/evaluation-treatment-outcomes-graduate/docview/3161872070/se-2	Excluded	Title/Abstract	
169	Chouinard, M. (2016). Biomarkers of orthodontic tooth movement with fixed appliances and vibration device: A randomized clinical trial (Order No. 31081108). Available from ProQuest Dissertations & Theses Global. (3073208103). Retrieved from https://www.proquest.com/dissertations-theses/biomarkers-orthodontic-tooth-movement-with-fixed/docview/3073208103/se-2	Excluded	Title/Abstract	
170	Machaka, M. (2018). Outcomes of secondary alveolar bone grafts in cleft patients (Order No. 31796999). Available from ProQuest Dissertations & Theses Global. (3161869799). Retrieved from https://www.proquest.com/dissertations-theses/outcomes-secondary-alveolar-bone-grafts-cleft/docview/3161869799/se-2	Excluded	Title/Abstract	

171	S., V. (2019). Effect of transverse dimensional changes on the health of the buccal bone in the maxillary arch of using passive self-ligating brackets using CBCT: An in vivo study (Order No. 30586389). Available from ProQuest Dissertations & Theses Global. (2866351912). Retrieved from https://www.proquest.com/dissertations-theses/effect-transverse-dimensional-changes-on-health/docview/2866351912/se-2	Excluded	Title/Abstract	
172	Devi, T. T. (2018). Perioral soft tissue evaluation of different skeletal malocclusions: A cephalometric study (Order No. 30587397). Available from ProQuest Dissertations & Theses Global. (2866351393). Retrieved from https://www.proquest.com/dissertations-theses/perioral-soft-tissue-evaluation-different/docview/2866351393/se-2	Excluded	Title/Abstract	
173	Bomvana, V. (2020). Treatment difficulty assessment of postgraduate orthodontic cases at a university clinic (Order No. 31799846). Available from ProQuest Dissertations & Theses Global. (3161868039). Retrieved from https://www.proquest.com/dissertations-theses/treatment-difficulty-assessment-postgraduate/docview/3161868039/se-2	Excluded	Title/Abstract	
174	R., P. G. (2006). Efficiency of newer generation pre-adjusted edgewise applications- A randomised controlled trail (Order No. 30282820). Available from ProQuest Dissertations & Theses Global. (2786886279). Retrieved from https://www.proquest.com/dissertations-theses/efficiency-newer-generation-pre-adjusted-edgewise/docview/2786886279/se-2	Excluded	Title/Abstract	
175	Biradar, A. K. (2010). To establish soft tissue norms for males and females of south indian population by profile angle and aesthetic analysis (Order No. 30277422). Available from ProQuest Dissertations & Theses Global. (2786887810). Retrieved from https://www.proquest.com/dissertations-	Excluded	Title/Abstract	

	theses/establish-soft-tissue-norms-males-females-south/docview/2786887810/se-2			
176	Mowafi, M. (2002). Cephalometric evaluation of molar distalization by hyrax screw used in conjunction with lip bumper (Order No. 28513400). Available from ProQuest Dissertations & Theses Global. (2567984147). Retrieved from https://www.proquest.com/dissertations-theses/cephalometric-evaluation-molar-distalization/docview/2567984147/se-2	Excluded	Title/Abstract	
177	Nesaragi, M. M. (2018). Assessing the effects of submucosal injection of platelet rich plasma (PRP) in accelerating orthodontic tooth movement – an in vivo study (Order No. 30586317). Available from ProQuest Dissertations & Theses Global. (2866352664). Retrieved from https://www.proquest.com/dissertations-theses/assessing-effects-submucosal-injection-platelet/docview/2866352664/se-2	Excluded	Title/Abstract	
178	de Carvalho, T. P. (2023). White spot lesions : Diagnosis and treatment : A systematic review (Order No. 30862173). Available from ProQuest Dissertations & Theses Global. (2957139715). Retrieved from https://www.proquest.com/dissertations-theses/white-spot-lesions-diagnosis-treatment-systematic/docview/2957139715/se-2	Excluded	Title/Abstract	
179	Dawood, M. (2019). Prevalence of tooth size discrepancy (bolton index) among raichur population": An in vitro study (Order No. 30585289). Available from ProQuest Dissertations & Theses Global. (2866352335). Retrieved from https://www.proquest.com/dissertations-theses/prevalence-tooth-size-discrepancy-bolton-index/docview/2866352335/se-2	Excluded	Title/Abstract	

180	Carraro, M. (2018). Smile aesthetics: An interdisciplinary approach (Order No. 30211177). Available from ProQuest Dissertations & Theses Global. (2763454065). Retrieved from https://www.proquest.com/dissertations-theses/smile-aesthetics-interdisciplinary-approach/docview/2763454065/se-2	Excluded	Title/Abstract	
181	Dhanashetti, R. (2016). Osteocalcin levels in peri-miniscrew implant crevicular fluid during orthodontic tooth movement: An in-vivo study (Order No. 30276957). Available from ProQuest Dissertations & Theses Global. (2786883317). Retrieved from https://www.proquest.com/dissertations-theses/osteocalcin-levels-peri-miniscrew-implant/docview/2786883317/se-2	Excluded	Title/Abstract	
182	Kunal, K. (2017). Evaluation and comparison of diode laser, nano carbonate apatite dentifrice, and their combination over dentinal tubules occlusion by scanning electron microscopy – an in vitro study (Order No. 30276536). Available from ProQuest Dissertations & Theses Global. (2786886571). Retrieved from https://www.proquest.com/dissertations-theses/evaluation-comparison-diode-laser-nano-carbonate/docview/2786886571/se-2	Excluded	Title/Abstract	
183	Singla, L. (2006). Rapid orthodontic tooth movement into newly distracted bone - A clinical study (Order No. 30282875). Available from ProQuest Dissertations & Theses Global. (2786888820). Retrieved from https://www.proquest.com/dissertations-theses/rapid-orthodontic-tooth-movement-into-newly/docview/2786888820/se-2	Excluded	Title/Abstract	
184	Devasia, N. (2019). A study of hard tissue cephalometric norms for kerala population using burstone COGS analysis (Order No. 30587177). Available from ProQuest Dissertations & Theses Global. (2866351224).	Excluded	Title/Abstract	

	Retrieved from https://www.proquest.com/dissertations-theses/study-hard-tissue-cephalometric-norms-kerala/docview/2866351224/se-2			
185	Ananda, R. (2018). Evaluation of laser aided papilla-split procedure to prevent rotational relapse-A randomized controlled trial (Order No. 30586417). Available from ProQuest Dissertations & Theses Global. (2866353934). Retrieved from https://www.proquest.com/dissertations-theses/evaluation-laser-aided-papilla-split-procedure/docview/2866353934/se-2	Excluded	Title/Abstract	
186	Priebe, D. N. (2000). The effectiveness of differential moment strategies in anchorage control during space closure (Order No. 31077257). Available from ProQuest Dissertations & Theses Global. (3073204341). Retrieved from https://www.proquest.com/dissertations-theses/effectiveness-differential-moment-strategies/docview/3073204341/se-2	Excluded	Title/Abstract	
187	Singh, A. (2016). A comparative evaluation of EN masse retraction in class II malocclusion cases treated with mini-implants and conventional orthodontic mechanics - A clinical study (Order No. 30587899). Available from ProQuest Dissertations & Theses Global. (2866352355). Retrieved from https://www.proquest.com/dissertations-theses/comparative-evaluation-en-masse-retraction-class/docview/2866352355/se-2	Excluded	Title/Abstract	
188	Waremani, A. S. (2019). Effects of mandibular canine intrusion obtained using cantilever vs bone anchorage: A comparative finite element study (Order No. 30584780). Available from ProQuest Dissertations & Theses Global. (2866349724). Retrieved from https://www.proquest.com/dissertations-theses/effects-mandibular-canine-intrusion-obtained/docview/2866349724/se-2	Excluded	Title/Abstract	

189	Sharp, J. A. (2021). On the relationship between condylar cortication and mandibular growth activity (Order No. 28648740). Available from ProQuest Dissertations & Theses Global. (2572604917). Retrieved from https://www.proquest.com/dissertations-theses/on-relationship-between-condylar-cortication/docview/2572604917/se-2	Excluded	Title/Abstract	
190	Frick, C. J. (2021). Clinical feasibility evaluation of digital dental articulation for 3-piece maxillary orthognathic surgery (Order No. 28652675). Available from ProQuest Dissertations & Theses Global. (2567993314). Retrieved from https://www.proquest.com/dissertations-theses/clinical-feasibility-evaluation-digital-dental/docview/2567993314/se-2	Excluded	Title/Abstract	
191	Litsas, G. (2005). Comparison of cervical vertebral maturation and hand wrist skeletal maturation growth indicators in orthodontic patients (Order No. 28523859). Available from ProQuest Dissertations & Theses Global. (2570011609). Retrieved from https://www.proquest.com/dissertations-theses/comparison-cervical-vertebral-maturation-hand/docview/2570011609/se-2	Excluded	Title/Abstract	
192	A., S. (2019). A comparative study of colour stability, load deflection, modulus of elasticity and tensile strength of coated and uncoated NI-TI arch wires-an invitro study (Order No. 30583962). Available from ProQuest Dissertations & Theses Global. (2866352692). Retrieved from https://www.proquest.com/dissertations-theses/comparative-study-colour-stability-load/docview/2866352692/se-2	Excluded	Title/Abstract	
193	Nair, S.,S. (2018). A comparison of cervical vertebrae and modified MP3 stages using periapical radiograph for assessment of skeletal maturity (Order No. 30586508). Available from ProQuest Dissertations & Theses Global. (2866349842). Retrieved from	Excluded	Title/Abstract	

	https://www.proquest.com/dissertations-theses/comparison-cervical-vertebrae-modified-mp3-stages/docview/2866349842/se-2			
194	Monehi, S. E. (2014). Comparing two orthodontic brackets' bond to fluorosed and non-fluorosed enamel - an in vitro study (Order No. 30707754). Available from ProQuest Dissertations & Theses Global. (2901484028). Retrieved from https://www.proquest.com/dissertations-theses/comparing-two-orthodontic-brackets-bond-fluorosed/docview/2901484028/se-2	Excluded	Title/Abstract	
195	Han, S. M. (2022). Cbct use in orthodontic residency programs in usa (Order No. 29283530). Available from ProQuest Dissertations & Theses Global. (2714865103). Retrieved from https://www.proquest.com/dissertations-theses/cbct-use-orthodontic-residency-programs-usa/docview/2714865103/se-2	Excluded	Title/Abstract	
196	Saidalavi, S.,K. (2018). Cephalometric evaluation of patients treated by maxillary anterior segmental distraction (Order No. 30584378). Available from ProQuest Dissertations & Theses Global. (2866356127). Retrieved from https://www.proquest.com/dissertations-theses/cephalometric-evaluation-patients-treated/docview/2866356127/se-2	Excluded	Title/Abstract	
197	Datta, A. G. (2008). To determine the average canine inclination values in different types of arch forms in karnataka population (Order No. 30559552). Available from ProQuest Dissertations & Theses Global. (2848392057). Retrieved from https://www.proquest.com/dissertations-theses/determine-average-canine-inclination-values/docview/2848392057/se-2	Excluded	Title/Abstract	

198	Sarkar, S. R. (2016). Changes in the force degradation of orthodontic elastomeric chains when subjected to different formulations of chlorhexidine mouthrinse - an in vitro study (Order No. 30581174). Available from ProQuest Dissertations & Theses Global. (2866084610). Retrieved from https://www.proquest.com/dissertations-theses/changes-force-degradation-orthodontic-elastomeric/docview/2866084610/se-2	Excluded	Title/Abstract	
199	Varghese, V. (2016). Comparison of streptococcus mutans count in plaque around the orthodontic brackets before and after using different forms of probiotics (Order No. 30580351). Available from ProQuest Dissertations & Theses Global. (2866083376). Retrieved from https://www.proquest.com/dissertations-theses/comparison-streptococcus-mutans-count-plaque/docview/2866083376/se-2	Excluded	Title/Abstract	
200	M., S. (2018). An evaluation of the bond strength using 3 different primer (transbond XT primer, moisture insensitive primer and new liquid polish sealer) in presence of saliva contamination, an in-vivo study (Order No. 30584590). Available from ProQuest Dissertations & Theses Global. (2866357097). Retrieved from https://www.proquest.com/dissertations-theses/evaluation-bond-strength-using-3-different-primer/docview/2866357097/se-2	Excluded	Title/Abstract	
201	Nilosha, C. C. M. (2019). Evaluation and comparision of bracket slot dimensions in lingual bracket systems – an in vitro study (Order No. 30584493). Available from ProQuest Dissertations & Theses Global. (2866352669). Retrieved from https://www.proquest.com/dissertations-theses/evaluation-comparision-bracket-slot-dimensions/docview/2866352669/se-2	Excluded	Title/Abstract	

202	Al Jawad, Feras H. Abedal. (2006). Comparison between cervical headgear and teuscher in the treatment of class II division I malocclusion (Order No. 28552528). Available from ProQuest Dissertations & Theses Global. (2561545055). Retrieved from https://www.proquest.com/dissertations-theses/comparison-between-cervical-headgear-teuscher/docview/2561545055/se-2	Excluded	Title/Abstract	
203	Thulaseedharan, S. (2019). A CBCT study to evaluate the changes in the alveolar bone thickness, vertical and sagittal positioning of the maxillary and mandibular anteriors after en-masse retraction (Order No. 30588089). Available from ProQuest Dissertations & Theses Global. (2866352775). Retrieved from https://www.proquest.com/dissertations-theses/cbct-study-evaluate-changes-alveolar-bone/docview/2866352775/se-2	Excluded	Title/Abstract	
204	Singh, A. (2016). Evaluation of efficiency of two nanohydroxyapatite remineralising agents with a hydroxyapatite and a conventional dentifrice - A comparative in vitro study (Order No. 30576422). Available from ProQuest Dissertations & Theses Global. (2848773753). Retrieved from https://www.proquest.com/dissertations-theses/evaluation-efficiency-two-nanohydroxyapatite/docview/2848773753/se-2	Excluded	Title/Abstract	
205	Zapata, G. (2013). Differences in lip projection with and without labial denture flanges in a maxillary edentulous population - an objective analysis (Order No. 31079648). Available from ProQuest Dissertations & Theses Global. (3073206397). Retrieved from https://www.proquest.com/dissertations-theses/differences-lip-projection-with-without-labial/docview/3073206397/se-2	Excluded	Title/Abstract	
206	Taimour, A. (2018). Permanent tooth auto-transplantation in pediatrics as a treatment alternative – A systematic review (Order No. 31261213). Available from ProQuest Dissertations & Theses Global. (3098798157).	Excluded	Title/Abstract	

	Retrieved from https://www.proquest.com/dissertations-theses/permanent-tooth-auto-transplantation-pediatrics/docview/3098798157/se-2			
207	Lundgren, G. P. (2015). Early restorative rehabilitation of children and adolescents with amelogenesis imperfecta (Order No. 28425305). Available from ProQuest Dissertations & Theses Global. (2567990463). Retrieved from https://www.proquest.com/dissertations-theses/early-restorative-rehabilitation-children/docview/2567990463/se-2	Excluded	Title/Abstract	
208	Halapanavar, B. (2019). "Comparison of skeletal changes in glenoid fossa, condylar head and articular space between twin block appliance and fixed functional appliance using cone beam computed tomography" – A clinical prospective study (Order No. 30586519). Available from ProQuest Dissertations & Theses Global. (2866352078). Retrieved from https://www.proquest.com/dissertations-theses/comparison-skeletal-changes-glenoid-fossa/docview/2866352078/se-2	Excluded	Title/Abstract	
209	Rahman, O. U. R. (2005). Surgically assisted retraction in MBT technique-A clinical study (Order No. 30283058). Available from ProQuest Dissertations & Theses Global. (2786887507). Retrieved from https://www.proquest.com/dissertations-theses/surgically-assisted-retraction-mbt-technique/docview/2786887507/se-2	Excluded	Title/Abstract	
210	Chaturvedi, B. K. (2018). Assessment of lower third molar eruption in different anteroposterior skeletal patterns and age-related groups: A radiographic study (Order No. 30586564). Available from ProQuest Dissertations & Theses Global. (2866350701). Retrieved from https://www.proquest.com/dissertations-theses/assessment-lower-third-molar-eruption-different/docview/2866350701/se-2	Excluded	Title/Abstract	

211	Mishra, A. (2016). A comparative assessment of frictional resistance between lingual brackets and archwires in wet and dry condition by a pin on disk type friction testing machine - an in-vitro study (Order No. 30576466). Available from ProQuest Dissertations & Theses Global. (2848774417). Retrieved from https://www.proquest.com/dissertations-theses/comparative-assessment-frictional-resistance/docview/2848774417/se-2	Excluded	Title/Abstract	
212	Gupta, C. (2018). A cone beam computed tomography study to correlate the oropharyngeal airway dimensions in Angle's class II division I patients treated with functional appliance- an invitro study (Order No. 30585067). Available from ProQuest Dissertations & Theses Global. (2866352465). Retrieved from https://www.proquest.com/dissertations-theses/cone-beam-computed-tomography-study-correlate/docview/2866352465/se-2	Excluded	Title/Abstract	
213	Tabassum, J. (2019). Biomechanical effects of maxillary expansion on a patient with bilateral cleft lip and palate"-A finite element study (Order No. 30587149). Available from ProQuest Dissertations & Theses Global. (2866351372). Retrieved from https://www.proquest.com/dissertations-theses/biomechanical-effects-maxillary-expansion-on/docview/2866351372/se-2	Excluded	Title/Abstract	
214	Tippashetty, S.,K. (2019). To check the reliability of hamular notch incisive papilla plane evaluator for orientation of occlusal plane in dentulous subjects: An in-vivo study (Order No. 30583985). Available from ProQuest Dissertations & Theses Global. (2866351067). Retrieved from https://www.proquest.com/dissertations-theses/check-reliability-hamular-notch-incisive-papilla/docview/2866351067/se-2	Excluded	Title/Abstract	

215	Taslan, S. (2008). Evaluation of changes in tongue pressure before during and after crib appliance therapy in open bite cases (Order No. 28594335). Available from ProQuest Dissertations & Theses Global. (2561545990). Retrieved from https://www.proquest.com/dissertations-theses/evaluation-changes-tongue-pressure-before-during/docview/2561545990/se-2	Excluded	Title/Abstract	
216	John, P. C. (2016). A comparative evaluation of the periodontal parameters in various dentofacial growth patterns (Order No. 30276911). Available from ProQuest Dissertations & Theses Global. (2786887238). Retrieved from https://www.proquest.com/dissertations-theses/comparative-evaluation-periodontal-parameters/docview/2786887238/se-2	Excluded	Title/Abstract	
217	P, J.,Pamila Ross. (2018). Stress distribution in different root morphologies on orthodontic loading- 3D finite element study (Order No. 30584918). Available from ProQuest Dissertations & Theses Global. (2866351275). Retrieved from https://www.proquest.com/dissertations-theses/stress-distribution-different-root-morphologies/docview/2866351275/se-2	Excluded	Title/Abstract	
218	Narain, A. (2016). Determining the mesio distal width of maxillary central incisor among davangere population by correlating the inner canthal distance and interpupillary distance to geometric proportion and biometric width respectively an- in vivo study (Order No. 30585129). Available from ProQuest Dissertations & Theses Global. (2866350820). Retrieved from https://www.proquest.com/dissertations-theses/determining-mesio-distal-width-maxillary-central/docview/2866350820/se-2	Excluded	Title/Abstract	

219	Potgieter, N. (2017). A comparison between the band-and-loop space maintainer with a loop-design fibre-reinforced composite space maintainer (Order No. 30709637). Available from ProQuest Dissertations & Theses Global. (2901493210). Retrieved from https://www.proquest.com/dissertations-theses/comparison-between-band-loop-space-maintainer/docview/2901493210/se-2	Excluded	Title/Abstract	
220	Shaban, H. M. (2003). Treatment effects of the forsus appliance: A lateral cephalometric and magnetic resonance imaging study (Order No. 28532034). Available from ProQuest Dissertations & Theses Global. (2561514448). Retrieved from https://www.proquest.com/dissertations-theses/treatment-effects-forsus-appliance-lateral/docview/2561514448/se-2	Excluded	Title/Abstract	
221	Kumar, K. (2018). Alveolar and symphysis region characteristics of patients with skeletal class III malocclusion –A cephalometric study (Order No. 30583615). Available from ProQuest Dissertations & Theses Global. (2866086469). Retrieved from https://www.proquest.com/dissertations-theses/alveolar-symphysis-region-characteristics/docview/2866086469/se-2	Excluded	Title/Abstract	
222	Haralambidis, A. (2007). Morphological changes of the nasal cavity induced by rapid maxillary expansion. a study on 3D computed tomography models (Order No. 28551416). Available from ProQuest Dissertations & Theses Global. (2561545971). Retrieved from https://www.proquest.com/dissertations-theses/morphological-changes-nasal-cavity-induced-rapid/docview/2561545971/se-2	Excluded	Title/Abstract	

223	Jose, J. A. (2018). A comparative evaluation of interleukin 1β and prostaglandin E2 levels with and without low level laser therapy during en masse retraction – A biochemical study (Order No. 30585790). Available from ProQuest Dissertations & Theses Global. (2866352172). Retrieved from https://www.proquest.com/dissertations-theses/comparative-evaluation-interleukin-1β/docview/2866352172/se-2	Excluded	Title/Abstract	
224	Sygouros, A. (2013). Surgically assisted rapid maxillary expansion. an evaluation of two different surgical techniques and their effect on maxillary dentoskeletal complex based on cone-beam computed tomography : A retrospective clinical study (Order No. 28534174). Available from ProQuest Dissertations & Theses Global. (2561521618). Retrieved from https://www.proquest.com/dissertations-theses/surgically-assisted-rapid-maxillary-expansion/docview/2561521618/se-2	Excluded	Title/Abstract	
225	P., S. (2018). Biological evaluation of patterns of orthodontic forces on the crevicular alkaline phosphatase activity: An in-vivo study (Order No. 30585608). Available from ProQuest Dissertations & Theses Global. (2866350730). Retrieved from https://www.proquest.com/dissertations-theses/biological-evaluation-patterns-orthodontic-forces/docview/2866350730/se-2	Excluded	Title/Abstract	
226	K., R. K. (2018). A radiographic study of relationship between maxillary canine impaction and sella turcica bridging in bangalore urban orthodontic patients (Order No. 30583652). Available from ProQuest Dissertations & Theses Global. (2866087129). Retrieved from https://www.proquest.com/dissertations-theses/radiographic-study-relationship-between-maxillary/docview/2866087129/se-2	Excluded	Title/Abstract	

227	Vijesh. (2016). Comparison of W angle, pi angle and yen angle as an indicator of assessing sagittal dysplasia- A cephalometric study (Order No. 30582481). Available from ProQuest Dissertations & Theses Global. (2866082477). Retrieved from https://www.proquest.com/dissertations-theses/comparison-w-angle-pi-yen-as-indicator-assessing/docview/2866082477/se-2	Excluded	Title/Abstract	
228	Palo, L. (2020). Retrospective evaluation of the survival rate of buccal mini-implants and the factors associated with their survival (Order No. 31080898). Available from ProQuest Dissertations & Theses Global. (3073208553). Retrieved from https://www.proquest.com/dissertations-theses/retrospective-evaluation-survival-rate-buccal/docview/3073208553/se-2	Excluded	Title/Abstract	
229	John, N. (2018). Effect of lubricants on friction in self-ligating ceramic brackets with stainless steel and esthetic arch-wire (Order No. 30584256). Available from ProQuest Dissertations & Theses Global. (2866352773). Retrieved from https://www.proquest.com/dissertations-theses/effect-lubricants-on-friction-self-ligating/docview/2866352773/se-2	Excluded	Title/Abstract	
230	de Matos, M.,Tourais Nunes. (2022). Composite veneers: The direct-indirect technique – clinical case report (Order No. 30879611). Available from ProQuest Dissertations & Theses Global. (3039373256). Retrieved from https://www.proquest.com/dissertations-theses/composite-veneers-direct-indirect-technique/docview/3039373256/se-2	Excluded	Title/Abstract	
231	Efthimiadou, D. (2006). Evaluation of dental and skeletal changes due to surgically assisted rapid maxillary expansion (Order No. 28536929). Available from ProQuest Dissertations & Theses Global. (2561521684). Retrieved from https://www.proquest.com/dissertations-	Excluded	Title/Abstract	

	theses/evaluation-dental-skeletal-changes-due-surgically/docview/2561521684/se-2			
232	R., R. (2019). Chemical and biological evaluation of different commercially available metal orthodontic bracketsan invitro study (Order No. 30584447). Available from ProQuest Dissertations & Theses Global. (2866353382). Retrieved from https://www.proquest.com/dissertations-theses/chemical-biological-evaluation-different/docview/2866353382/se-2	Excluded	Title/Abstract	
233	Ziadeh, S. K. (2006). Cephalometric evaluation of skeletal and dental effects of bionator vd. teuscher appliance: A comparative study (Order No. 28516296). Available from ProQuest Dissertations & Theses Global. (2567984027). Retrieved from https://www.proquest.com/dissertations-theses/cephalometric-evaluation-skeletal-dental-effects/docview/2567984027/se-2	Excluded	Title/Abstract	
234	, S.,B.V. (2016). Temporomandibular disorders- A clinico-radiologic study (Order No. 30557556). Available from ProQuest Dissertations & Theses Global. (2848396787). Retrieved from https://www.proquest.com/dissertations-theses/temporomandibular-disorders-clinico-radiologic/docview/2848396787/se-2	Excluded	Title/Abstract	
235	R., A. (2019). Comparison of recession coverage using periosteal pedicle graft alone and in combination with low level laser therapy - A randomised controlled clinical trial (Order No. 30586097). Available from ProQuest Dissertations & Theses Global. (2866349616). Retrieved from https://www.proquest.com/dissertations-theses/comparison-recession-coverage-using-periosteal/docview/2866349616/se-2	Excluded	Title/Abstract	

236	Dippenaar, A. M. (2005). Utilising radiographic incisor crown markers to determine incisor inclination on lateral headfilms - and experimental study on extracted teeth (Order No. 30701389). Available from ProQuest Dissertations & Theses Global. (2890697888). Retrieved from https://www.proquest.com/dissertations-theses/utilising-radiographic-incisor-crown-markers/docview/2890697888/se-2	Excluded	Title/Abstract	
237	Agarwal, A. (2016). Estimation of N-telopeptide levels in peri miniscrew implant crevicular fluid during orthodontic tooth movement: An invivo study (Order No. 30579975). Available from ProQuest Dissertations & Theses Global. (2866084655). Retrieved from https://www.proquest.com/dissertations-theses/estimation-n-telopeptide-levels-peri-miniscrew/docview/2866084655/se-2	Excluded	Title/Abstract	
238	R., K. B. (2019). Biomechanical simulation of mandibular first molar protraction into second premolar extraction space with temporary anchorage devices- A finite element study (Order No. 30584127). Available from ProQuest Dissertations & Theses Global. (2866353846). Retrieved from https://www.proquest.com/dissertations-theses/biomechanical-simulation-mandibular-first-molar/docview/2866353846/se-2	Excluded	Title/Abstract	
239	B., S. (2016). Assessment of skeletal and dental maturation in different facial types of south indian population – A comparative study (Order No. 30583992). Available from ProQuest Dissertations & Theses Global. (2866353613). Retrieved from https://www.proquest.com/dissertations-theses/assessment-skeletal-dental-maturation-different/docview/2866353613/se-2	Excluded	Title/Abstract	

240	Pamporakis, P. (2012). Three dimensional evaluation of changes in maxillary sinuses and pharyngeal airway in class iii maxillary deficiency cases undergoing orthopedic facemask treatment (Order No. 28521206). Available from ProQuest Dissertations & Theses Global. (2571013276). Retrieved from https://www.proquest.com/dissertations-theses/three-dimensional-evaluation-changes-maxillary/docview/2571013276/se-2	Excluded	Title/Abstract	
241	Andreopoulos, İ. N. (2013). Three-dimensional analysis of morphological changes in the face and maxilla of patients with unilateral complete cleft lip and palate after presurgical nasoalveolar molding treatment (Order No. 28532042). Available from ProQuest Dissertations & Theses Global. (2561513886). Retrieved from https://www.proquest.com/dissertations-theses/three-dimensional-analysis-morphological-changes/docview/2561513886/se-2	Excluded	Title/Abstract	
242	Moger, G. L. (2006). Transcutaneous electrical nerve stimulation therapy in temporomandibular disorder – A clinical study (Order No. 30283264). Available from ProQuest Dissertations & Theses Global. (2787194543). Retrieved from https://www.proquest.com/dissertations-theses/transcutaneous-electrical-nerve-stimulation/docview/2787194543/se-2	Excluded	Title/Abstract	
243	Dubey, P. (2017). Assessment of mandibular foramen location using CBCT (Order No. 30278133). Available from ProQuest Dissertations & Theses Global. (2786886908). Retrieved from https://www.proquest.com/dissertations-theses/assessment-mandibular-foramen-location-using-cbct/docview/2786886908/se-2	Excluded	Title/Abstract	
244	Bagga, V. (2016). Efficacy of articaine and lignocaine on palatal anesthesia on bilateral maxillary premolar extractions – A comparative study (Order No. 30583296). Available from ProQuest Dissertations &	Excluded	Title/Abstract	

	Theses Global. (2866086879). Retrieved from https://www.proquest.com/dissertations-theses/efficacy-articaine-lignocaine-on-palatal/docview/2866086879/se-2			
245	V., S. K. (2009). Determination of the relative parallelism of occlusal plane to three ala-tragal lines in various skeletal malocclusions-A cephalometric study (Order No. 30283288). Available from ProQuest Dissertations & Theses Global; ProQuest One Literature. (2787193510). Retrieved from https://www.proquest.com/dissertations-theses/determination-relative-parallelism-occlusal-plane/docview/2787193510/se-2	Excluded	Title/Abstract	
246	Naidu, P. (2009). Correlation between skeletal maturation and mandibular growth changes in children and adolescents: A cephalometric study (Order No. 30582206). Available from ProQuest Dissertations & Theses Global. (2866084947). Retrieved from https://www.proquest.com/dissertations-theses/correlation-between-skeletal-maturation/docview/2866084947/se-2	Excluded	Title/Abstract	
247	Haris, E. (2019). Evaluation of realationship between cervical vertebral maturity and maxillary canine, mandibular second molar, mandibular premolar calcification stages (Order No. 30583832). Available from ProQuest Dissertations & Theses Global. (2866350507). Retrieved from https://www.proquest.com/dissertations-theses/evaluation-realationship-between-cervical/docview/2866350507/se-2	Excluded	Title/Abstract	
248	Hanson, M. S. (2021). Effect of pad abrasion on shear bond strength of 3D-printed orthodontic brackets (Order No. 28650314). Available from ProQuest Dissertations & Theses Global. (2562790237). Retrieved from https://www.proquest.com/dissertations-theses/effect-pad-abrasion-on-shear-bond-strength-3d/docview/2562790237/se-2	Excluded	Title/Abstract	

249	Vaz, A. C. R. (2019). Midpalatal suture maturation assessment on panoramic X-rays and its correlation with cervical vertebrae maturation (Order No. 31057828). Available from ProQuest Dissertations & Theses Global. (3086165686). Retrieved from https://www.proquest.com/dissertations-theses/midpalatal-suture-maturation-assessment-on/docview/3086165686/se-2	Excluded	Title/Abstract	
250	Ulrich, M. S. (2011). Can Bell's palsy synkinesis be improved by PNF?(Order No. 30277559). Available from ProQuest Dissertations & Theses Global. (2786887546). Retrieved from https://www.proquest.com/dissertations-theses/can-bell-s-palsy-synkinesis-be-improved-pnf/docview/2786887546/se-2	Excluded	Title/Abstract	
251	Salameh, F. H. (2005). Reliability of measurements done on hand-tracings cephalometric radiographs, compared to measurements done by two different computerized analysis software (Order No. 28538562). Available from ProQuest Dissertations & Theses Global. (2561544993). Retrieved from https://www.proquest.com/dissertations-theses/reliability-measurements-done-on-hand-tracings/docview/2561544993/se-2	Excluded	Title/Abstract	
252	D., A. (2017). Comparative evaluation of cone-beam computed tomography versus direct surgical measurements in the diagnosis of mandibular molar furcation involvement (Order No. 30278525). Available from ProQuest Dissertations & Theses Global. (2786887254). Retrieved from https://www.proquest.com/dissertations-theses/comparative-evaluation-cone-beam-computed/docview/2786887254/se-2	Excluded	Title/Abstract	

253	C., D. S. (2018). Variation in tooth size measurement measured at occlusal level and contact points and its correlation with arch size – A CBCT study (Order No. 30586563). Available from ProQuest Dissertations & Theses Global. (2866352067). Retrieved from https://www.proquest.com/dissertations-theses/variation-tooth-size-measurement-measured-at/docview/2866352067/se-2	Excluded	Title/Abstract	
254	Mullen, S. R. (2004). Accuracy of performing space analysis using EmodelsTM and plaster models (Order No. 30397398). Available from ProQuest Dissertations & Theses Global. (2812065806). Retrieved from https://www.proquest.com/dissertations-theses/accuracy-performing-space-analysis-using-emodels/docview/2812065806/se-2	Excluded	Title/Abstract	
255	Mohammed, N. A. (2003). The effects of compound palatal arch se* (super elastic) in children (Order No. 28524790). Available from ProQuest Dissertations & Theses Global. (2567984108). Retrieved from https://www.proquest.com/dissertations-theses/effects-compound-palatal-arch-se-super-elastic/docview/2567984108/se-2	Excluded	Title/Abstract	
256	Heigrujam, J. (2019). Assessment of developmental dental anomalies and root development in bilateral and unilateral cleft lip and palate children (Order No. 30586937). Available from ProQuest Dissertations & Theses Global. (2866353009). Retrieved from https://www.proquest.com/dissertations-theses/assessment-developmental-dental-anomalies-root/docview/2866353009/se-2	Excluded	Title/Abstract	
257	B., L. K. (2006). Long term bony and soft tissue stability following genioplasty-A cephalometric study (Order No. 30560399). Available from ProQuest Dissertations & Theses Global. (2848394158). Retrieved from https://www.proquest.com/dissertations-theses/long-term-bony-soft-tissue-stability-following/docview/2848394158/se-2	Excluded	Title/Abstract	

258	Sarin, S. (2018). Study of stress distribution in craniofacial structures following implant assisted maxillary protraction - A three dimensional fem study (Order No. 30586936). Available from ProQuest Dissertations & Theses Global. (2866350599). Retrieved from https://www.proquest.com/dissertations-theses/study-stress-distribution-craniofacial-structures/docview/2866350599/se-2	Excluded	Title/Abstract	
259	da Silva, Carolina Pacheco,Veiga Dias. (2020). Dentoalveolar and skeletal effects on bone born marpe vs hybrid marpe appliances : A three dimensional retrospective comparative study (Order No. 30966245). Available from ProQuest Dissertations & Theses Global. (3059336080). Retrieved from https://www.proquest.com/dissertations-theses/dentoalveolar-skeletal-effects-on-bone-born-marpe/docview/3059336080/se-2	Excluded	Title/Abstract	
260	Basha, M. Q. (2018). Corelation of skeletal maturation of cervical vertebrae with dental maturation of lower canine- A clinical radiologic study (Order No. 30587886). Available from ProQuest Dissertations & Theses Global. (2866352140). Retrieved from https://www.proquest.com/dissertations-theses/corelation-skeletal-maturation-cervical-vertebrae/docview/2866352140/se-2	Excluded	Title/Abstract	
261	Iyengar, S.,S. (2016). A cephalometric evaluation of airway space in skeletal class II subjects with normal maxilla (Order No. 30581236). Available from ProQuest Dissertations & Theses Global. (2866081923). Retrieved from https://www.proquest.com/dissertations-theses/cephalometric-evaluation-airway-space-skeletal/docview/2866081923/se-2	Excluded	Title/Abstract	

262	Hamed, T. (2006). A meta analysis of the skeletal and dental effects of fixed functional appliances treatment (Order No. 28534315). Available from ProQuest Dissertations & Theses Global. (2561545044). Retrieved from https://www.proquest.com/dissertations-theses/meta-analysis-skeletal-dental-effects-fixed/docview/2561545044/se-2	Excluded	Title/Abstract	
263	Chambers, D. R. (2014). The effective dose of different scanning protocols using the sirona galileos® comfort CBCT scanner (Order No. 29242014). Available from ProQuest Dissertations & Theses Global. (2701132780). Retrieved from https://www.proquest.com/dissertations-theses/effective-dose-different-scanning-protocols-using/docview/2701132780/se-2	Excluded	Title/Abstract	
264	Kanwal, B. (2018). Comparison of soft tissue and airway changes following bimaxillary surgery versus mandibular setback surgery in correction of skeletal class III malocclusion -A cephalometric study (Order No. 30584802). Available from ProQuest Dissertations & Theses Global. (2866350532). Retrieved from https://www.proquest.com/dissertations-theses/comparison-soft-tissue-airway-changes-following/docview/2866350532/se-2	Excluded	Title/Abstract	
265	Hammoude, S. (2005). Ortodontic and orthoredic effects of activator and cervical headgear: A comparative study (Order No. 28519140). Available from ProQuest Dissertations & Theses Global. (2567983935). Retrieved from https://www.proquest.com/dissertations-theses/ortodontic-orthoredic-effects-activator-cervical/docview/2567983935/se-2	Excluded	Title/Abstract	
266	Agrwal, S. K. (2018). Determination of skeeletal growth pattern using panoramic radiographs (Order No. 30584116). Available from ProQuest Dissertations & Theses Global. (2866350540). Retrieved from https://www.proquest.com/dissertations-theses/determination-skeeletal-growth-pattern-using/docview/2866350540/se-2	Excluded	Title/Abstract	

267	Tank, K. (2016). A cbct analysis of buccal bone thickness in maxilla and mandible and its clinical significance in monocortical screws placement (Order No. 30278139). Available from ProQuest Dissertations & Theses Global. (2786883341). Retrieved from https://www.proquest.com/dissertations-theses/cbct-analysis-buccal-bone-thickness-maxilla/docview/2786883341/se-2	Excluded	Title/Abstract	
268	M.S.P.S., S. (2009). Bite force evaluation in patients treated with titanium conventional plating system and titanium locking plating system for mandibular fractures (Order No. 30560461). Available from ProQuest Dissertations & Theses Global. (2848393838). Retrieved from https://www.proquest.com/dissertations-theses/bite-force-evaluation-patients-treated-with/docview/2848393838/se-2	Excluded	Title/Abstract	
269	Devendra, P. S. (2009). Treatment of localized gingival recession using the free rotated papilla autograft combined with coronally advanced flap by conventional and microsurgical technique - A comparative clinical study (Order No. 30560417). Available from ProQuest Dissertations & Theses Global. (2848395174). Retrieved from https://www.proquest.com/dissertations-theses/treatment-localized-gingival-recession-using-free/docview/2848395174/se-2	Excluded	Title/Abstract	
270	Raso, G. (2022). The influence of root prominence in the onset of gingival recession and its significance as a risk factor for root coverage treatment : A systematic review (Order No. 30852198). Available from ProQuest Dissertations & Theses Global. (2956852819). Retrieved from https://www.proquest.com/dissertations-theses/influence-root-prominence-onset-gingival/docview/2956852819/se-2	Excluded	Title/Abstract	

271	Özkaya, S. (2009). One year follow-up of the changes of the nasal cavity morphology induced by rapid maxillary expansion. a study on 3-D computed tomography models (Order No. 28519620). Available from ProQuest Dissertations & Theses Global. (2570999104). Retrieved from https://www.proquest.com/dissertations-theses/one-year-follow-up-changes-nasal-cavity/docview/2570999104/se-2	Excluded	Title/Abstract	
272	Igdayir, K. M. A. S. (2020). Investigation of inflammatory responses following the use of novel anisotropic hydrogel tissue expander in the skin of maxillofacial region (Order No. 30598469). Available from ProQuest Dissertations & Theses Global. (2877956630). Retrieved from https://www.proquest.com/dissertations-theses/investigation-inflammatory-responses-following/docview/2877956630/se-2	Excluded	Title/Abstract	
273	Belludi, A. C. (2017). A correlative study of dermatoglyphic patterns with class I and III skeletal malocclusions (Order No. 30278554). Available from ProQuest Dissertations & Theses Global. (2786883854). Retrieved from https://www.proquest.com/dissertations-theses/correlative-study-dermatoglyphic-patterns-with/docview/2786883854/se-2	Excluded	Title/Abstract	
274	Marti, S. J. (2021). Maxillary transverse expansion- A study of dentoalveolar effects with TAD-supported RMES versus traditional RMES (Order No. 28412272). Available from ProQuest Dissertations & Theses Global. (2560876256). Retrieved from https://www.proquest.com/dissertations-theses/maxillary-transverse-expansion-study/docview/2560876256/se-2	Excluded	Title/Abstract	
275	Garza, J. J. (2021). Investigation of bacterial adhesion to 3D-printed nasoalveolar molding appliance acrylic (Order No. 28652686). Available from ProQuest Dissertations & Theses Global. (2564491230). Retrieved	Excluded	Title/Abstract	

	from https://www.proquest.com/dissertations-theses/investigation-bacterial-adhesion-3d-printed/docview/2564491230/se-2			
276	G., C. D. (2017). Analysis of condyle - fossa relationship in completely edentulous patients before and after prosthetic rehabilitation with complete dentures (Order No. 30278468). Available from ProQuest Dissertations & Theses Global. (2786883572). Retrieved from https://www.proquest.com/dissertations-theses/analysis-condyle-fossa-relationship-completely/docview/2786883572/se-2	Excluded	Title/Abstract	
277	Majhi, S. S. (2019). Expression of HIF-1α in human gingival connective tissue graft, with and without laser therapy in the treatment of gingival root coverage procedure - A clinico-biomolecular study (Order No. 30583991). Available from ProQuest Dissertations & Theses Global. (2866356148). Retrieved from https://www.proquest.com/dissertations-theses/expression-hif-1α-human-gingival-connective/docview/2866356148/se-2	Excluded	Title/Abstract	
278	Castanheira, C. V. (2020). Management of pulp canal obliteration: Systematic review of case reports (Order No. 31181278). Available from ProQuest Dissertations & Theses Global. (3098767570). Retrieved from https://www.proquest.com/dissertations-theses/management-pulp-canal-obliteration-systematic/docview/3098767570/se-2	Excluded	Title/Abstract	
279	Welling, A. (2021). A longitudinal assessment of the effects of breastfeeding on the development of arch morphology and malocclusion (Order No. 28319386). Available from ProQuest Dissertations & Theses Global. (2560824252). Retrieved from https://www.proquest.com/dissertations-theses/longitudinal-assessment-effects-breastfeeding-on/docview/2560824252/se-2	Excluded	Title/Abstract	

280	Shaan-E-Kareemi. (2019). Application of botulinum toxin type-A in the management of trigeminal neuralgia (Order No. 30584148). Available from ProQuest Dissertations & Theses Global. (2866350745). Retrieved from https://www.proquest.com/dissertations-theses/application-botulinum-toxin-type-management/docview/2866350745/se-2	Excluded	Title/Abstract	
281	Chopra, A. (2006). Cephalometeric evaluation of midface distraction osteogenesis in cleft lip and palate patients - A longitudinal study (Order No. 30282876). Available from ProQuest Dissertations & Theses Global. (2786882928). Retrieved from https://www.proquest.com/dissertations-theses/cephalometeric-evaluation-midface-distraction/docview/2786882928/se-2	Excluded	Title/Abstract	
282	Willis, N. G. (2021). The effect of post-print processing protocol on adhesive strength of soft acrylic to 3D-printed hard acrylic (Order No. 28651227). Available from ProQuest Dissertations & Theses Global. (2564171549). Retrieved from https://www.proquest.com/dissertations-theses/effect-post-print-processing-protocol-on-adhesive/docview/2564171549/se-2	Excluded	Title/Abstract	
283	N., D. R. (2018). A comparative study of condyle and glenoid fossa positions in dentulous and edentulous patients on lateral cephalogram (Order No. 30586691). Available from ProQuest Dissertations & Theses Global. (2866349645). Retrieved from https://www.proquest.com/dissertations-theses/comparative-study-condyle-glenoid-fossa-positions/docview/2866349645/se-2	Excluded	Title/Abstract	
284	Gupta, A. (2019). Efficacy of autogenously bone ring augmentation with simultaneous implant placement in inadequate socket (Order No. 30586656). Available from ProQuest Dissertations & Theses Global. (2866351183). Retrieved from https://www.proquest.com/dissertations-	Excluded	Title/Abstract	

	theses/efficacy-autogenously-bone-ring-augmentation-with/docview/2866351183/se-2			
285	Pandit, H. R. (2017). Comparison of platelet rich fibrin (PRF) and connective tissue graft (CTG) utilizing vestibular incision subperiosteal tunnel access (VISTA) technique in the management of multiple gingival recession: A clinical study (Order No. 30278444). Available from ProQuest Dissertations & Theses Global. (2786887259). Retrieved from https://www.proquest.com/dissertations-theses/comparison-platelet-rich-fibrin-prf-connective/docview/2786887259/se-2	Excluded	Title/Abstract	
286	Sukumaran, P. (2016). Effects of non-alcoholic and alcoholic mouthwashes on force degradation of orthodontic elastomeric chains – an in-vitro comparative study (Order No. 30579437). Available from ProQuest Dissertations & Theses Global. (2866083488). Retrieved from https://www.proquest.com/dissertations-theses/effects-non-alcoholic-mouthwashes-on-force/docview/2866083488/se-2	Excluded	Title/Abstract	
287	Thakur, I. (2017). Accuracy of oro-cervical radiographic indexes in determining the chronological age- a digital radiographic study (Order No. 30282523). Available from ProQuest Dissertations & Theses Global. (2786887894). Retrieved from https://www.proquest.com/dissertations-theses/accuracy-oro-cervical-radiographic-indexes/docview/2786887894/se-2	Excluded	Title/Abstract	
288	Thomas, A. (2006). Comparison of accuracy of bracket placement between direct and indirect bonding techniques- an invitro study (Order No. 30282931). Available from ProQuest Dissertations & Theses Global. (2786883369). Retrieved from https://www.proquest.com/dissertations-	Excluded	Title/Abstract	

	theses/comparison-accuracy-bracket-placement-between/docview/2786883369/se-2			
289	C., G. (2018). Comparative evaluation of modified coronally advanced tunnel with collagen matrix versus connective tissue graft for root coverage in multiple gingival recessionsa randomised controlled clinical trial (Order No. 30586623). Available from ProQuest Dissertations & Theses Global. (2866353395). Retrieved from https://www.proquest.com/dissertations-theses/comparative-evaluation-modified-coronally/docview/2866353395/se-2	Excluded	Title/Abstract	
290	Naqvi, Z. A. (2010). Effect of cleft lip and palate repair on craniofacial growth - A comparative study between operated and unoperated cleft lip and palate patients in southern karnataka population (Order No. 30277535). Available from ProQuest Dissertations & Theses Global. (2786886358). Retrieved from https://www.proquest.com/dissertations-theses/effect-cleft-lip-palate-repair-on-craniofacial/docview/2786886358/se-2	Excluded	Title/Abstract	
291	Hassan, S. (2009). Efficacy of 4% articaine HCL and 2% lignocaine HCL in extraction of maxillary premolars for orthodontic reason a comparative study (Order No. 30560336). Available from ProQuest Dissertations & Theses Global. (2848395246). Retrieved from https://www.proquest.com/dissertations-theses/efficacy-4-articaine-hcl-2-lignocaine-extraction/docview/2848395246/se-2	Excluded	Title/Abstract	
292	Khairi, J. (2017). The antero-posterior location of the mental foramen in subjects with different skeletal pattern (Order No. 30600696). Available from ProQuest Dissertations & Theses Global. (2877960108). Retrieved	Excluded	Title/Abstract	

	from https://www.proquest.com/dissertations-theses/antero-posterior-location-mental-foramen-subjects/docview/2877960108/se-2			
293	N., D. K. (2008). Long-term comparison of treatment outcome and stability of classii patients treated with fixed bite jumping appliances verus bilateral sagital split osteotomy - A cephalometric study (Order No. 30559452). Available from ProQuest Dissertations & Theses Global. (2848392415). Retrieved from https://www.proquest.com/dissertations-theses/long-term-comparison-treatment-outcome-stability/docview/2848392415/se-2	Excluded	Title/Abstract	
294	Meky, M. (2023). The effect of bite turbos on mandibular anterior bracket failure rate: A retrospective study (Order No. 30669874). Available from ProQuest Dissertations & Theses Global. (2890692710). Retrieved from https://www.proquest.com/dissertations-theses/effect-bite-turbos-on-mandibular-anterior-bracket/docview/2890692710/se-2	Excluded	Title/Abstract	
295	Jalali, E. (2015). A morphological and volumetric airway analysis of patients with mandibulofacial abnormalities using cone beam computed tomography (Order No. 31079888). Available from ProQuest Dissertations & Theses Global. (3073207032). Retrieved from https://www.proquest.com/dissertations-theses/morphological-volumetric-airway-analysis-patients/docview/3073207032/se-2	Excluded	Title/Abstract	
296	Pauly, N. G. (2018). Morphometric analysis of the mental foramen and mandibular canal using OPG and CBCT: A comparative radiographic study (Order No. 30586841). Available from ProQuest Dissertations & Theses Global. (2866350563). Retrieved from https://www.proquest.com/dissertations-theses/morphometric-analysis-mental-foramen-mandibular/docview/2866350563/se-2	Excluded	Title/Abstract	

297	Catalano, M. (2021). Aesthetic surgical treatment in anterior maxilla – comparison between lip repositioning versus crown lengthening: Literature review (Order No. 30205993). Available from ProQuest Dissertations & Theses Global. (2748384803). Retrieved from https://www.proquest.com/dissertations-theses/aesthetic-surgical-treatment-anterior-maxilla/docview/2748384803/se-2	Excluded	Title/Abstract	
298	Madhukant, A. (2005). Clinical efficiancy of collagen as a guided tissue regeneration membrane (healguide) in the treatment of localized gingival recession (Order No. 30282813). Available from ProQuest Dissertations & Theses Global. (2786886653). Retrieved from https://www.proquest.com/dissertations-theses/clinical-efficiancy-collagen-as-guided-tissue/docview/2786886653/se-2	Excluded	Title/Abstract	
299	Basheer, S. M. (2019). Physics forceps versus conventional forceps in maxillary molar extraction: A comparative study (Order No. 30588392). Available from ProQuest Dissertations & Theses Global. (2866351834). Retrieved from https://www.proquest.com/dissertations-theses/physics-forceps-versus-conventional-maxillary/docview/2866351834/se-2	Excluded	Title/Abstract	
300	Maniyar, A. K. (2019). Establishing of norms for vertical maxillary excess in north karnataka population- A cephalometric study (Order No. 30585912). Available from ProQuest Dissertations & Theses Global. (2866352414). Retrieved from https://www.proquest.com/dissertations-theses/establishing-norms-vertical-maxillary-excess/docview/2866352414/se-2	Excluded	Title/Abstract	
301	Suresh, S. (2018). Comperision of mucoderm® and connective tissue graft(CTG) utilizing nodified pinhole technique in the management of multiple gingival recession: A clinical study (Order No. 30588457). Available from ProQuest Dissertations & Theses Global. (2866349269).	Excluded	Title/Abstract	

	Retrieved from https://www.proquest.com/dissertations-theses/comperision-mucoderm®-connective-tissue-graft-ctg/docview/2866349269/se-2			
302	Ihwigat, M. A. (2006). The effect of using mouthwashes to prevent bacteremia during initial therapy of periondontal treatment (Order No. 28550202). Available from ProQuest Dissertations & Theses Global. (2561545001). Retrieved from https://www.proquest.com/dissertations-theses/effect-using-mouthwashes-prevent-bacteremia/docview/2561545001/se-2	Excluded	Title/Abstract	
303	Mansabdar, P. (2017). A comparison between conventional arch bars and modified arch bars (WITH SCREWS) in maxillofacial fractures (Order No. 30278580). Available from ProQuest Dissertations & Theses Global. (2786883253). Retrieved from https://www.proquest.com/dissertations-theses/comparison-between-conventional-arch-bars/docview/2786883253/se-2	Excluded	Title/Abstract	
304	D., V. (2017). To assess the effectiveness of sugar free chewing gum with CPP-ACP on streptococcus mutans count" – A double blind randomized controlled study (Order No. 30282465). Available from ProQuest Dissertations & Theses Global. (2786883270). Retrieved from https://www.proquest.com/dissertations-theses/assess-effectiveness-sugar-free-chewing-gum-with/docview/2786883270/se-2	Excluded	Title/Abstract	
305	D'Silva, J. (2016). A prospective comparitive clinical study of conventional erich arch bar, intermaxillary fixation screws and sullia modified erich arch bar in the management of maxillofacial trauma (Order No. 30278137). Available from ProQuest Dissertations & Theses Global. (2786883393). Retrieved from	Excluded	Title/Abstract	

	https://www.proquest.com/dissertations-theses/prospective-comparitive-clinical-study/docview/2786883393/se-2			
306	Lerner, H. (2021). Experimental and clinical results to support digital workflows in implant dentistry (Order No. 30763272). Available from ProQuest Dissertations & Theses Global. (2901818952). Retrieved from https://www.proquest.com/dissertations-theses/experimental-clinical-results-support-digital/docview/2901818952/se-2	Excluded	Title/Abstract	
307	Barbedo, A. G. (2021). Maxillary disjunction: The effectiveness of marpe method in the palatine suture - A narrative review (Order No. 30206456). Available from ProQuest Dissertations & Theses Global. (2748386605). Retrieved from https://www.proquest.com/dissertations-theses/maxillary-disjunction-effectiveness-marpe-method/docview/2748386605/se-2	Excluded	Title/Abstract	
308	Madaíl, A.,Carolina Sarrico. (2020). Upper airways volume and craniofacial morphology: A CBCT retrospective study (Order No. 29139750). Available from ProQuest Dissertations & Theses Global. (2689289406). Retrieved from https://www.proquest.com/dissertations-theses/upper-airways-volume-craniofacial-morphology-cbct/docview/2689289406/se-2	Excluded	Title/Abstract	
309	Modi, N. (2010). Effect of disinfecting solutions on the mechanical properties of orthodontic elastomeric ligatures - in vitro study (Order No. 30282222). Available from ProQuest Dissertations & Theses Global. (2786883767). Retrieved from https://www.proquest.com/dissertations-theses/effect-disinfecting-solutions-on-mechanical/docview/2786883767/se-2	Excluded	Title/Abstract	

310	Saba, S. (2019). Comparative evaluation of periogen™ and platelet rich fibrin in the treatment of Miller's class I and ii gingival recession defects (Order No. 30586570). Available from ProQuest Dissertations & Theses Global. (2866349416). Retrieved from https://www.proquest.com/dissertations-theses/comparative-evaluation-periogen™-platelet-rich/docview/2866349416/se-2	Excluded	Title/Abstract	
311	Cavalcanti, L. B. (2013). Comparative study of the biomechanical behaviour of extraoral force modules (Order No. 30204553). Available from ProQuest Dissertations & Theses Global. (2748386548). Retrieved from https://www.proquest.com/dissertations-theses/comparative-study-biomechanical-behaviour/docview/2748386548/se-2	Excluded	Title/Abstract	
312	Sengupta, G. (2019). Assess the morphology of soft palate by using CBCT in mangalore population (Order No. 30583899). Available from ProQuest Dissertations & Theses Global. (2866351876). Retrieved from https://www.proquest.com/dissertations-theses/assess-morphology-soft-palate-using-cbct/docview/2866351876/se-2	Excluded	Title/Abstract	
313	Shahapur, S. (2018). Analysis of airway dimensions in normodivergent, hypodivergent and hyperdivergent individuals in relation to cranio-cervical angulation -- in davanagere population (Order No. 30586897). Available from ProQuest Dissertations & Theses Global. (2866350991). Retrieved from https://www.proquest.com/dissertations-theses/analysis-airway-dimensions-normodivergent/docview/2866350991/se-2	Excluded	Title/Abstract	
314	Singh, M. (2017). Evaluation of bone level, gingival esthetics, stability and success rate in immediately provisionalized dental implant replacing single missing maxillary or mandibular tooth - A prospective study (Order No. 30587822). Available from ProQuest Dissertations & Theses Global. (2866350923). Retrieved from	Excluded	Title/Abstract	

	https://www.proquest.com/dissertations-theses/evaluation-bone-level-gingival-esthetics/docview/2866350923/se-2			
315	Shukla, A. (2019). Evaluation of efficacy of direct bonded orthodontic bracket with E-chain for intermaxillary fixation (Order No. 30585282). Available from ProQuest Dissertations & Theses Global. (2866351427). Retrieved from https://www.proquest.com/dissertations-theses/evaluation-efficacy-direct-bonded-orthodontic/docview/2866351427/se-2	Excluded	Title/Abstract	
316	Yamany, I. (2011). A comparative analysis of different fields of view in cone beam computed tomography imaging to identify the inferior alveolar nerve canal (Order No. 31079979). Available from ProQuest Dissertations & Theses Global. (3073206392). Retrieved from https://www.proquest.com/dissertations-theses/comparative-analysis-different-fields-view-cone/docview/3073206392/se-2	Excluded	Title/Abstract	
317	H.V., A. (2018). The correlation between certain facial and dental measurements that influence dental aesthetics" – an in vivo study (Order No. 30586231). Available from ProQuest Dissertations & Theses Global. (2866351245). Retrieved from https://www.proquest.com/dissertations-theses/correlation-between-certain-facial-dental/docview/2866351245/se-2	Excluded	Title/Abstract	
318	Yeddula, K. (2019). Comparision of conventional and cone beam computed tomography generated cephalograms for reliability and reproducibility of landmarks (Order No. 30583810). Available from ProQuest Dissertations & Theses Global. (2866350095). Retrieved from	Excluded	Title/Abstract	

	https://www.proquest.com/dissertations-theses/comparision-conventional-cone-beam-computed/docview/2866350095/se-2			
319	Ambica. (2016). Evaluation of expression and distribution of fibronectin on the root surface of teeth in chronic and aggressive periodontitis: An immunohistochemical study (Order No. 30279467). Available from ProQuest Dissertations & Theses Global. (2786887680). Retrieved from https://www.proquest.com/dissertations-theses/evaluation-expression-distribution-fibronectin-on/docview/2786887680/se-2	Excluded	Title/Abstract	
320	Raghav, K. (2006). Evaluation and comparison of composite posts orthodontic "y" wire posts and glass fiber posts in restoric badly decayed primary incisors-an in vitro and in vivo study (Order No. 30283176). Available from ProQuest Dissertations & Theses Global. (2786886783). Retrieved from https://www.proquest.com/dissertations-theses/evaluation-comparison-composite-posts-orthodontic/docview/2786886783/se-2	Excluded	Title/Abstract	
321	Ibragim, S. A. (2017). Oral status among down syndrome patients (Order No. 31261150). Available from ProQuest Dissertations & Theses Global. (3098798076). Retrieved from https://www.proquest.com/dissertations-theses/oral-status-among-down-syndrome-patients/docview/3098798076/se-2	Excluded	Title/Abstract	
322	Nawi, N. A. M. (2018). Anatomical variations of maxillary sinus septa and their relationship to the antral volume in patients with different skeletal profiles (Order No. 30598003). Available from ProQuest Dissertations & Theses Global. (2877960849). Retrieved from	Excluded	Title/Abstract	

	https://www.proquest.com/dissertations-theses/anatomical-variations-maxillary-sinus-septa-their/docview/2877960849/se-2			
323	S., J. D. (2005). Comparison of treatment with acupuncture and conventional methods in the management of frozen shoulder (Order No. 30580817). Available from ProQuest Dissertations & Theses Global. (2866084416). Retrieved from https://www.proquest.com/dissertations-theses/comparison-treatment-with-acupuncture/docview/2866084416/se-2	Excluded	Title/Abstract	
324	Bharat, G. R. (2016). Comparative study on the efficacy of physics forceps versus conventional forceps in dental extractions (Order No. 30278121). Available from ProQuest Dissertations & Theses Global. (2786887658). Retrieved from https://www.proquest.com/dissertations-theses/comparative-study-on-efficacy-physics-forceps/docview/2786887658/se-2	Excluded	Title/Abstract	
325	N., D. A. (2016). Comparison of the fracture resistance of reattached incisor tooth fragments with circumferential bevel, notches, grooves and palatal cavity- an in vitro study (Order No. 30278146). Available from ProQuest Dissertations & Theses Global. (2786883722). Retrieved from https://www.proquest.com/dissertations-theses/comparison-fracture-resistance-reattached-incisor/docview/2786883722/se-2	Excluded	Title/Abstract	
326	Bhavsar, A. K. (2019). A comparative clinical and optical evaluation of self-filling osmotic tissue expander in augmenting keratinized tissue around dentulous region (Order No. 30584637). Available from ProQuest Dissertations & Theses Global. (2866350057). Retrieved from https://www.proquest.com/dissertations-theses/comparative-clinical-optical-evaluation-self/docview/2866350057/se-2	Excluded	Title/Abstract	

327	Nagaral, S. (2009). Isolation of candida species from the oral cavity and fingertips of complete denture wearers (Order No. 30560506). Available from ProQuest Dissertations & Theses Global. (2848396815). Retrieved from https://www.proquest.com/dissertations-theses/isolation-candida-species-oral-cavity-fingertips/docview/2848396815/se-2	Excluded	Title/Abstract	
328	K.P., S. (2018). A photographic assessment of nose after cleft rhinoplasty in a patients with complete unilateral cleft lip and palate (Order No. 30586520). Available from ProQuest Dissertations & Theses Global. (2866351987). Retrieved from https://www.proquest.com/dissertations-theses/photographic-assessment-nose-after-cleft/docview/2866351987/se-2	Excluded	Title/Abstract	
329	Mukhia, N. (2019). A comparison of geometric accuracy of three dimensional bone surface modelling on cone beam computed tomography and white light scanner (Order No. 30588506). Available from ProQuest Dissertations & Theses Global. (2866351768). Retrieved from https://www.proquest.com/dissertations-theses/comparison-geometric-accuracy-three-dimensional/docview/2866351768/se-2	Excluded	Title/Abstract	
330	Malkani, S. (2019). Comparative study of bonded polyethylene fiber reinforced ribbon and stainless steel wire composite resin splint in treatment of dentoalveolar fracture (Order No. 30584666). Available from ProQuest Dissertations & Theses Global. (2866349512). Retrieved from https://www.proquest.com/dissertations-theses/comparative-study-bonded-polyethylene-fiber/docview/2866349512/se-2	Excluded	Title/Abstract	
331	Muralidharan, N. (2017). Assessment of relationship of maxillary sinus floor topography and maxillary sinus mucosal thickening with root position of maxillary posterior teeth with periapical infections using limited volume cone beam computed tomography (Order No. 30582706).	Excluded	Title/Abstract	

	Available from ProQuest Dissertations & Theses Global. (2866086644). Retrieved from https://www.proquest.com/dissertations-theses/assessment-relationship-maxillary-sinus-floor/docview/2866086644/se-2			
332	Nargotra, R. (2005). Use of distraction osteogenesis as a treatment modality in repaired cleft lip-palate patient (Order No. 30282901). Available from ProQuest Dissertations & Theses Global. (2786886762). Retrieved from https://www.proquest.com/dissertations-theses/use-distraction-osteogenesis-as-treatment/docview/2786886762/se-2	Excluded	Title/Abstract	
333	Bamzahim, M. S. (2005). Evaluation of the DIAGNOdent method for detection and quantification of carious lesions : In vitro and in vivo studies (Order No. 28426728). Available from ProQuest Dissertations & Theses Global. (2571087831). Retrieved from https://www.proquest.com/dissertations-theses/evaluation-diagnodent-method-detection/docview/2571087831/se-2	Excluded	Title/Abstract	
334	S., V. (2008). Evaluation of the efficacy of plaque removal and maintenance of gingival health using manual and powered toothbrushes with and without toothpaste (Order No. 30560066). Available from ProQuest Dissertations & Theses Global. (2848394124). Retrieved from https://www.proquest.com/dissertations-theses/evaluation-efficacy-plaque-removal-maintenance/docview/2848394124/se-2	Excluded	Title/Abstract	
335	Malhotra, D. (2006). Self tapping imf screws, A simplified method of intermaxillary fixation-A clinical study (Order No. 30282861). Available from ProQuest Dissertations & Theses Global. (2786886725). Retrieved from https://www.proquest.com/dissertations-theses/self-tapping-imf-screws-simplified-method/docview/2786886725/se-2	Excluded	Title/Abstract	

336	Szabó, B. (2020). Factors influencing the success of root-amputated and restored maxillary molar teeth (Order No. 30763369). Available from ProQuest Dissertations & Theses Global. (2901816226). Retrieved from https://www.proquest.com/dissertations-theses/factors-influencing-success-root-amputated/docview/2901816226/se-2	Excluded	Title/Abstract	
337	Pychkina-Grandjean, A. (2018). Early caries detection: A systematic review (Order No. 31261183). Available from ProQuest Dissertations & Theses Global. (3098799671). Retrieved from https://www.proquest.com/dissertations-theses/early-caries-detection-systematic-review/docview/3098799671/se-2	Excluded	Title/Abstract	
338	Hosseinzadehfard, P. (2023). Pulpos kraujo PH vertinimas pulpito atvejais (Order No. 31269856). Available from ProQuest Dissertations & Theses Global. (3098805851). Retrieved from https://www.proquest.com/dissertations-theses/pulpos-kraujo-ph-vertinimas-pulpito-atvejais/docview/3098805851/se-2	Excluded	Title/Abstract	
339	Ulery, G. (2022). Principal component analysis observing different parameters of medical factors influencing dental health conditions of pediatric patients (Order No. 29280956). Available from ProQuest Dissertations & Theses Global. (2714864613). Retrieved from https://www.proquest.com/dissertations-theses/principal-component-analysis-observing-different/docview/2714864613/se-2	Excluded	Title/Abstract	
340	Shetty, A. (2005). The association between dental arch dimensions and dental abnormalities of the maxillary arch with the occurance of consonant misarticulations in cleft-palate children:A comparative study (Order No. 30282846). Available from ProQuest Dissertations & Theses Global; ProQuest One Literature. (2786888890). Retrieved from	Excluded	Title/Abstract	

	https://www.proquest.com/dissertations-theses/association-between-dental-arch-dimensions/docview/2786888890/se-2			
341	V., S. B. V. (2006). Comparative evaluation of subpithelial connective tissue graft either with autologois platelet rich plasma(PRP) or fibrin-fibronectin sealing system (tissuecol) in the treatment of localized gingival recession-A clinical study (Order No. 30283067). Available from ProQuest Dissertations & Theses Global. (2786883210). Retrieved from https://www.proquest.com/dissertations-theses/comparative-evaluation-subpithelial-connective/docview/2786883210/se-2	Excluded	Title/Abstract	
342	R., S. D. (2006). Effect of surgical tube drain with primary closure technique after the removal of impacted mandibular third molars-A clinical study (Order No. 30282853). Available from ProQuest Dissertations & Theses Global. (2786886817). Retrieved from https://www.proquest.com/dissertations-theses/effect-surgical-tube-drain-with-primary-closure/docview/2786886817/se-2	Excluded	Title/Abstract	
343	S.K., V. (2019). CBCT guided endodontics : Accuracy evaluation of access cavity preparation combined with optical surface scans - an invitro study (Order No. 30584026). Available from ProQuest Dissertations & Theses Global. (2866354060). Retrieved from https://www.proquest.com/dissertations-theses/cbct-guided-endodontics-accuracy-evaluation/docview/2866354060/se-2	Excluded	Title/Abstract	
344	Roy, B. (2019). Evaluation of two different forceps systems in extraction of maxillary molars - A prospective comparative study (Order No. 30584003). Available from ProQuest Dissertations & Theses Global. (2866350401). Retrieved from https://www.proquest.com/dissertations-	Excluded	Title/Abstract	

	theses/evaluation-two-different-forceps-systems/docview/2866350401/se-2			
345	Raghotham, K. (2006). Alveolar ridge augmentation in mandible using distraction osteogenesis (Order No. 30282899). Available from ProQuest Dissertations & Theses Global. (2786887736). Retrieved from https://www.proquest.com/dissertations-theses/alveolar-ridge-augmentation-mandible-using/docview/2786887736/se-2	Excluded	Title/Abstract	
346	Jacobucci, J. B. (2021). Measuring differences of interpretation among dentists on antibiotic prophylaxis guidelines (Order No. 28722844). Available from ProQuest Dissertations & Theses Global. (2557465032). Retrieved from https://www.proquest.com/dissertations-theses/measuring-differences-interpretation-among/docview/2557465032/se-2	Excluded	Title/Abstract	
